# Genome‐wide association coupled gene to gene interaction studies unveil novel epistatic targets among major effect loci impacting rice grain chalkiness

**DOI:** 10.1111/pbi.13516

**Published:** 2020-12-09

**Authors:** Gopal Misra, Saurabh Badoni, Sabiha Parween, Rakesh Kumar Singh, Hei Leung, OluFunmilayo Ladejobi, Richard Mott, Nese Sreenivasulu

**Affiliations:** ^1^ International Rice Research Institute Los Baños Philippines; ^2^ University College London London UK; ^3^ Present address: International Center for Biosaline Agriculture Academic City Dubai United Arab Emirates

**Keywords:** epistatic interactions, genome‐wide association studies, gene regulatory networks, grain quality, percent grain chalkiness, quantitative trait loci

## Abstract

Rice varieties whose quality is graded as excellent have a lower percent grain chalkiness (PGC) of two per cent and below with higher whole grain yields upon milling, leading to higher economic returns for farmers. We have conducted a genome‐wide association study (GWAS) using a combined population panel of *indica* and *japonica* rice varieties, and identified a total of 746 single nucleotide polymorphisms (SNPs) that were strongly associated with the chalk phenotype, covered 78 Quantitative Trait Loci (QTL) regions. Among them, 21 were high‐value QTLs, as they explained at least 10 % of the phenotypic variance for PGC. A combined epistasis and GWAS was applied to dissect the genetics of the complex chalkiness trait, and its regulatory cascades were validated using gene regulatory networks. Promising novel epistatic interactions were found between the loci of chromosomes 6 (*PGC6.1*) and 7 (*PGC7.8*) that contributed to lower PGC. Based on haplotype mining only a few modern rice varieties confounded with a lower chalkiness, and they possess several PGC QTLs. The importance of *PGC6.1* was validated through multi‐parent advanced generation intercrosses and several low‐chalk lines possessing superior haplotypes were identified. The results of this investigation have deciphered the underlying genetic networks that can reduce PGC to 2%, and will thus support future breeding programs to improve the grain quality of elite genetic material with high‐yielding potentials.

## Introduction

Rice (*Oryza sativa* L.) is one of the oldest domesticated crops in Asia, originating from the wild rice species *O. rufipogon*. It currently contributes to the daily energy needs of half of the world’s population (Butardo and Sreenivasulu, 2019). The two major subspecies of rice are *japonica*, which has short, bold grains with either a sticky or soft texture, and *indica* which has medium or long slender non‐sticky grains with a fluffy texture (Misra *et al*., 2018). Varieties that have a higher whole grain yield and a higher degree of translucency without the appearance of chalkiness (opaque discoloration) fetches higher premium due to an increased demand in the export market (Misra *et al*., 2019; Sreenivasulu *et al*., 2015). Rice traders consider milled seed lots with less than 2% chalky grains to be premium, 5% chalkiness is considered grade 1, 10% chalkiness as grade 2, and seed lots with more than 15% chalkiness are grade 3 and have the lowest market value (Laborte *et al*., 2015). Chalkiness is considered undesirable in part, because it has a negative impact on the cooking quality of the rice (Butardo and Sreenivasulu, 2019). Overall, this undesirable trait has a negative influence on consumer acceptability and crop market value (Laborte *et al*., 2015; Siebenmorgen *et al*., 2013). This trait is thus vital across the rice value chain, and it is crucial that natural variations in rice to reduce PGC are identified and utilized to improve elite high‐yielding lines, through breeding approaches.

PGC is a complex polygenic quantitative trait (Sreenivasulu *et al*., 2015). The chalkiness quality, however, is also affected by the increased incidence of high temperatures and humidity levels during the seed filling period (Ishimaru *et al*., 2018). PGC is triggered by lower starch granule densities, as irregularly shaped starch granules are loosely arranged, which creates air spaces between them, rendering their appearance quality poor and causing the grains to be prone to breakage during milling (Fitzgerald *et al*., 2009; Juliano, 1992; Siebenmorgen *et al*., 2013). Grain size‐related traits, such as a higher grain width with low or no amylose content, are known to confer unfavourable pleiotropic effects that increase grain chalkiness in *japonica* subspecies (Gong *et al*., 2017; Quero *et al*., 2018).

High‐yielding breeding populations often lack superior haplotypes that confer low levels of chalkiness, and as a result, many varieties released from 2006 to 2015 possess a median PGC of 10% (Misra *et al*., 2019). The available resequencing resources that were generated from diversity panels for the sub‐species *japonica* and *indica*, possess huge variation for grain quality traits, and thus amenable to conduct GWAS in rice (Misra *et al*., 2017). To date, several association peaks from chromosomes 1, 2, 3, 5, 6, 7, 8 and 12 have been identified that influence PGC and have been reproduced in multiple environments, but many have either low or moderate effect QTLs, explaining the low levels of phenotypic variance (Gong *et al*., 2017; Misra *et al*., 2019; Quero *et al*., 2018; Wang *et al*., 2017). Among the fine‐mapped moderate effect QTLs identified from the *indica* germplasm, the gene *chalk5* (encoding H^+^‐based pyrophosphatase) was found to affect cellular endomembrane trafficking, which impacts on PGC (Li *et al*., 2014). Recently, the fine‐mapped *chalk5.1* candidate gene with unknown function was located next to *GW5* known to influence the chalkiness (Misra *et al*., 2019).

Understanding epistasis‐based genetic interactions is crucial for capturing additional genetic information. Epistasis is defined as interactions between SNPs or loci across the genome, impacting target traits either additively to mutually increase a phenotypic effect or non‐additively to counter the effects of the major QTL, via other loci that interact epistatically (Mackay, 2014). In addition, epistasis has been documented in diversity panels subjected to selection and domestication in self‐pollinating crops (Carter *et al*., 2005; Doust *et al*., 2014). It has been established that genetic interactions can reveal the functional relationships between genes and thus point to the important regulators influencing quantitative traits in crops (Bocianowski, 2012; Eshed and Zamir, 1996; Li *et al*., 2014; Zhao *et al*., 2016). Recently, epistatic analysis has been successfully combined with GWAS as a complementary approach to dissect the underlying genetics of agronomic characteristics, such as flowering in maize (Assefa *et al*., 2019; Kim *et al*., 2020). Such systematic studies, however, have not yet been applied to the genetic interactions influencing grain chalkiness in rice.

Given that the heritable component of grain chalk is governed in a polygenic manner, understanding the epistatic interactions between the loci of the minor/major effect QTLs could unravel complex genetic interactions that impact on grain chalkiness. The present study combined whole‐genome resequencing‐based GWAS with epistatic interactions, to discover the genomic regions from a combined panel of *japonica* and *indica*, conferring low chalkiness in rice. We also identified a few low‐chalk breeding lines that possessed either individual chalk QTLs or QTL combinations through haplotype mining. We identified the causal effect genes within the key QTL regions that were distinguished from the pleiotropic effect, between chalkiness and other grain quality traits (amylose and grain size). In addition, the importance of the genetic regions was validated through multi‐parent advanced generation intercross (MAGIC) populations, specifically made from the intercrosses of sub‐species and lines identified as low chalk with low levels of amylose.

## Results

### Identification of genetic regions strongly associated with chalkiness using genome‐wide association studies in combined *japonica and indica* panels and MAGIC populations

**Figure 1:**
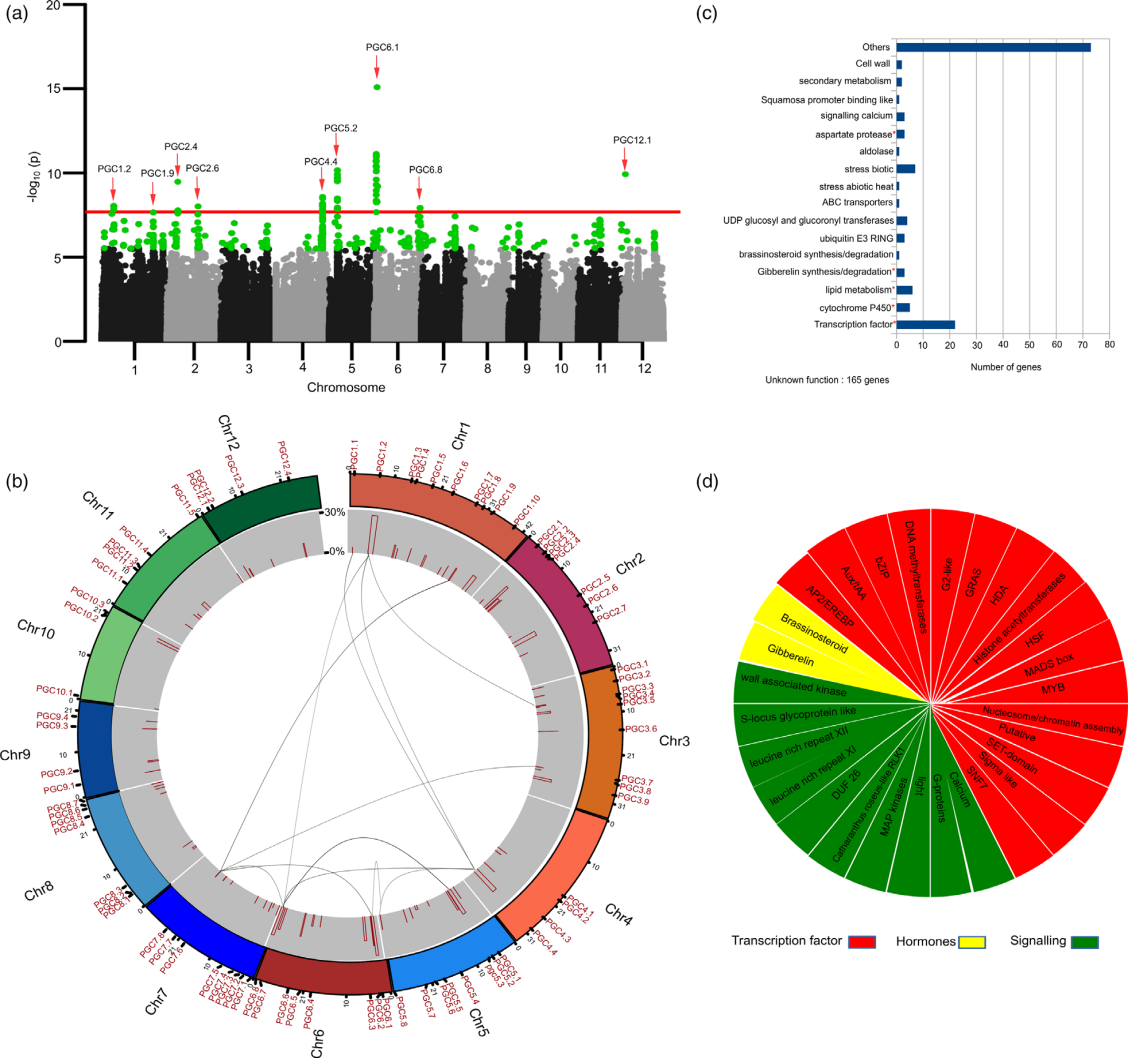
Single‐locus genome‐wide association study (GWAS) for per cent grain chalkiness (PGC) in combined diversity panel and genome‐wide distribution of identified chalk‐regulating QTLs with their functional categories. (a) Manhattan plot for PGC was plotted using combined panel of 583 accessions, showing the significant loci surpassing the Bonferroni threshold (‐log_10_ *P* > 7.68), indicated by red line, and FDR threshold by green dots. A significant number of loci influencing chalk were termed as PGCxy, based on their chromosome (x), and order of their physical position on chromosome (y). (b) Circos depicts the genome‐wide distribution of chalk‐regulating QTL on different chromosome positions, and along with phenotypic variation explained (PVE) showing their total effect on phenotype. The internal area of the circos shows the significant epistatic interactions between QTLs affecting PGC; (c) Functional categories of the genes identified underlying the chalk‐regulating QTLs and enriched functional categories depicted by the superscripted red asterisk. (d) Classification of sub‐functional categories of transcription factors, hormone and signalling molecule.

To identify the prominent genomic loci associated with the sources of the PGC variations in rice, we utilized a set of 583 lines of diverse germplasm comprising 299 *indica* and 284 *japonica* accessions from 71 countries. They also covered a wide range of PGC levels, ranging from 0 to 100, for both the *indica* and *japonica* populations (Figure S1). A total of 2.4 million quality‐assured SNPs were obtained from the total panel by aligning the sequences to the Nipponbare reference genome (MSU7). A principal component analysis (PCA) was conducted with the diversity panel data to assess the underlying genetic variation (Figure S2). The first two principal components (PC) explicitly explained 42.64% of the genetic variation, as the first PC separated the *indica* and *japonica* subspecies (38.28% of the variation), and the second PC distinguished the tropical *japonica* from the temperate *japonica* (4.36% of the variation) (Figure S2). By conducting the GWAS using ultra‐dense SNPs that accounted for the population structure, 746 significant SNPs associated with the PGC, belonging to 77 different QTLs, were identified from the different chromosomes, surpassing the Bonferroni threshold mark (−log_10_ (*P*) ≥ 7.68; red colour horizontal line) and the false discovery rate (FDR)‐threshold criterion (*P* < 0.05) (Figure 1a, b, Table 1, Table S1). The SNPs signified associations with chalk trait co‐located in the same genomic regions were considered as QTL (Table 1). The names of the QTLs significantly associated with the PGC were labelled using the format of PGCx.y, where x indicates the chromosome number and y signifies its order on the chromosome, based on its physical position (Figure 1a, b). Interestingly, when the GWAS was conducted only for the *japonica* panel, only chromosomes 2 (*PGC2.1*) and 6 (*PGC6.1*) were identified as having significant loci regulating the PGC (for further details, refer to Appendix S1). Furthermore, out of the 746 SNPs influencing grain chalkiness in the combined population, 590 SNPs negatively influenced the PGC and 138 positively affected the trait (Table S1). Out of a total of 77 QTLs, a set of 56 QTLs showed loci with minor to moderate effects across the genome, and their phenotypic variation explained (PVE) was low, and ranged between 2% and 9.8% (Figure 1b, Table S1). Importantly, there were 21 major QTLs conferring higher PVE values were shown in Table 1. These QTLs accounted for the shared variation and explained 72.18% of the total PVE, when all the identified PGC QTLs were considered. We mined the 30 × sequencing data available for the 92 breeding lines, for the presence of favourable allele under PGC QTLs and found that IRRI142 possessed 10 pyramided QTLs, and that IRRI143, IRRI133 and IRRI176 had 8, 3 and 2 QTLs, respectively, conferring lower chalkiness levels (Table 2). In addition, QTL *PGC5.2* was identified in two lines (IRRI176 and IRRI177) and QTL *PGC5.3* in multiple lines (IR42, IR64, IRRI134, IRRI135, IRRI139, IRRI143 and IRRI149), and both conferred a low‐chalk phenotype (Table 2).

**Table 1:** Significant QTLs (showing PVE ≥ 10) identified for regulating PGC along with their physical position on chromosomes, underlying topmost SNP, and further details

Chalk‐regulating QTLs							Top SNP details								
QTL ID	Chr	Start	End	*h* ^2^ ^§^	PVE^‡^	Length (Kb)	Top SNP	Allele Source^†^	Beta^§^	*P*‐value	Ref	Alt	Overlapping Gene	SNP effect	Functional Annotation
PGC1.2	1	6520765	8059695	0.27	26.8	1538.9	snp_01_7754524	J	−0.82	9.17E‐09	C	T	LOC_Os01g13820	upstream gene variant	Unclassified
PGC1.10	1	39443793	40645958	0.08	12.2	1202.2	snp_01_40488152	J	0.45	5.03E‐07	G	A	LOC_Os01g69990	synonymous variant	Unclassified
PGC2.2	2	5081459	5765468	0.19	21	684	snp_02_5765468	J	−0.74	1.06E‐07	G	C	LOC_Os02g10860	upstream gene variant	b‐ZIP transcription factor
PGC2.3	2	6019127	6764968	0.6	10.9	745.8	snp_02_6130172	J	−0.99	2.55E‐07	C	T	LOC_Os02g11830	upstream gene variant	Cell vesicle transport
PGC2.4	2	7100959	7136961	0.2	12.2	36	snp_02_7100959	J	−1.19	3.34E‐10	G	C	LOC_Os02g13320	upstream gene variant	Unclassified
PGC2.6	2	20302120	21603338	0.09	16.8	1301.2	snp_02_20575016	J	0.47	9.74E‐09	G	A	LOC_Os02g34380	synonymous variant	Unclassified
PGC3.6	3	15024656	15024656	0.1	13.1	0.001	snp_03_15024656	J	−0.8	2.22E‐06	G	C	LOC_Os03g26260	downstream gene variant	Stress biotic
PGC3.9	3	30084300	30791743	0.23	12.3	707.4	snp_03_30791743	J	−0.49	1.31E‐07	G	A	LOC_Os03g53700	5’‐UTR variant	Putative transcription regulator
PGC4.4	4	29923839	31212801	0.85	18.7	1289	snp_04_31024760	J	0.44	2.67E‐09	T	C	LOC_Os04g52220	synonymous variant	Unclassified
PGC5.2	5	5023459	5986536	0.55	19.4	963.1	snp_05_5371716	I	0.33	7.00E‐11	G	A	LOC_Os05g09520‐LOC_Os05g09530	intergenic region	NA
PGC5.3	5	6146864	6664004	0.27	15.1	517.1	snp_05_6146864	I	−0.30	1.51E‐07	T	C	LOC_Os05g10990	downstream gene variant	Unclassified
PGC5.8	5	29309709	29341831	0.39	10.3	32.1	snp_05_29337175	J	0.43	3.43E‐07	C	T	LOC_Os05g51140	synonymous variant	Unclassified
PGC6.1	6	1225608	1874664	0.51	27.5	649.1	snp_06_1824110	J	−1.07	8.13E‐16	C	T	LOC_Os06g04290	upstream gene variant	Protein synthesis
PGC6.2	6	2032400	2232920	0.19	20.6	200.5	snp_06_2232920	J	−0.95	6.29E‐10	G	A	LOC_Os06g05050	missense variant (Ala‐>Thr)	Wall‐associated kinase
PGC6.3	6	4005919	4647272	0.16	16.6	641.4	snp_06_4005919	J	−0.74	2.51E‐07	C	T	LOC_Os06g08270	upstream gene variant	Unclassified
PGC6.6	6	22794466	23104134	0.56	16.5	309.7	snp_06_22796392	J	−0.63	6.99E‐07	G	T	LOC_Os06g38460	upstream gene variant	Unclassified
PGC6.7	6	29158719	29708892	0.4	15.6	550.2	snp_06_29160497	J	−0.81	3.55E‐08	T	C	LOC_Os06g48200	upstream gene variant	Cell wall modification
PGC6.8	6	30184764	30974876	0.28	20.5	790.1	snp_06_30468379	J	−0.72	1.19E‐08	A	G	LOC_Os06g50330	upstream gene variant	Development unspecified
PGC9.1	9	3432724	3432724	0.09	10.3	0.001	snp_09_3432724	J	−0.82	2.98E‐06	G	A	LOC_Os09g07070	downstream gene variant	Unclassified
PGC10.2	10	20874591	20874591	0.15	15.4	0.001	snp_10_20874591	J	−0.55	6.05E‐07	C	T	LOC_Os10g39130	downstream gene variant	MADS‐box transcription factor
PGC10.3	10	21798319	21798319	0.1	15.9	0.001	snp_10_21798319	J	−0.86	1.72E‐06	T	C	LOC_Os10g40640	upstream gene variant	Unclassified

^†^ represent the source subspecies showing the major occurrence of alternate allele, where J and I indicate *Japonica*, and *Indica* subspecies, respectively.

^‡^ per cent of phenotypic variation explained by the respective QTL.

^§^ *h* ^2^ refers to narrow‐sense heritability, and beta signify the effect size of respective allele (refer method for details).

**Table 2:** QTL regions harboured by selected key entries from breeding panel and MAGIC population conferring low percent grain chalkiness

Selected Entries	QTLs present (Physical position in Mb)^‡^	Percent Grain Chalkiness	
		Season 1^†^	Season 2^†^
*Breeding panel*			
IRRI142	*PGC1.1 (1.55), PGC1.10 (39.44‐40.65), PGC2.5 (18.03), PGC2.6 (20.3‐21.6), PGC3.3 (6.46‐6.73), PGC3.8 (27.65), PGC7.6 (17.27‐17.28), PGC8.1 (3.65), PGC8.2 (4.17), PGC8.4 (24.53)*	2.2	8.3
IRRI152	*PGC1.1 (1.55),PGC2.5 (18.03), PGC2.7 (25.25), PGC3.3 (6.46‐6.73), PGC3.8 (27.65), PGC5.5 (17.20‐17.85), PGC5.8 (29.31‐29.34), PGC6.5 (20.02‐20.41)*	3	5.9
IRRI173	*PGC2.1 (3.16)*	3.2	6.1
IRRI133	*PGC4.4 (29.92‐31.21), PGC5.2 (5.02‐5.99), PGC5.3 (6.15‐6.66)*	1.8	3.1
IRRI177	*PGC5.2 (5.02‐5.99)*	3.4	2
IRRI176	*PGC5.2 (5.02‐5.99), PGC6.1 (1.23‐1.87)*	3.2	1.6
IRRI101	*PGC6.1 (1.23‐1.87)*	1.2	1.6
IR42	*PGC5.3 (6.15‐6.66)*	0.2	1.7
IR64	*PGC5.3 (6.15‐6.66)*	3.9	1
IRRI134	*PGC5.3 (6.15‐6.66)*	3.4	3
IRRI135	*PGC5.3 (6.15‐6.66)*	4.5	2.7
IRRI139	*PGC5.3 (6.15‐6.66)*	1.8	2.7
IRRI143	*PGC5.3 (6.15‐6.66)*	2	2.3
IRRI149	*PGC5.3 (6.15‐6.66)*	1	1.4
IR40	*PGC8.7 (28.22‐28.26)*	7	7.2
*MAGIC population*			
IR_99853‐B‐B‐B‐583	*PGC4.4 (29.92‐31.21)*	1.4	4.2
IR_99853‐B‐B‐B‐543	*PGC4.4 (29.92‐31.21)*	1.5	3.1
IR_99853‐B‐B‐B‐296	*PGC6.1 (1.23‐1.87)*	2.4	9.4
IR_99853‐B‐B‐B‐160	*PGC6.1 (1.23‐1.87)*	6	8.7
IR_99853‐B‐B‐B‐621	*PGC8.1 (3.65)*	1.5	1.2
IR_99853‐B‐B‐B‐470	*PGC8.1 (3.65), PGC8.2 (4.17)*	3.8	0.8
IR_99853‐B‐B‐B‐919	*PGC8.1 (3.65), PGC8.2 (4.17)*	7.4	4.6
IR_99853‐B‐B‐B‐830	*PGC8.1 (3.65), PGC8.2 (4.17)*	9.2	2.7
IR_99853‐B‐B‐B‐962	*PGC8.1 (3.65), PGC8.2 (4.17)*	0.4	0.9
IR_99853‐B‐B‐B‐989	*PGC8.1 (3.65), PGC8.2 (4.17)*	0.8	1.1
IR_99853‐B‐B‐B‐603	*PGC8.1 (3.65), PGC8.2 (4.17)*	1	3.3
IR_99853‐B‐B‐B‐38	*PGC8.1 (3.65), PGC8.2 (4.17)*	5.7	6.5
IR_99853‐B‐B‐B‐927	*PGC8.1 (3.65), PGC8.2 (4.17)*	0.3	4.9
IR_99853‐B‐B‐B‐291	*PGC8.1 (3.65), PGC8.2 (4.17)*	7	4.7

^†^ Breeding panel was evaluated for grain chalkiness phenotype in 2015 wet season (season 1) and 2016 dry season (season 2), while MAGIC population was evaluated in 2017 dry season (season 1) and 2018 dry season (season 2).

^‡^ If two corresponding physical positions within the QTL interval remain same up to two decimal places, only one physical position is mentioned for respective QTL.

A total of 65 genes were identified from the 21 QTL regions with higher PVE, and they were found to belong to the 16 functionally classified categories, using GOMapMan (http://www.gomapman.org) (Figure 1c). The functional categories that were enriched belonged to aspartate protease, gibberellin synthesis, lipid metabolism, cytochrome P450 and ~ 22 transcription factor genes (TFs) (Figure 1c, d). Among the TFs, the genes belonging to the GRAS TF family, DNA methyl transferase, several histone deacetylases and RNA polymerase sigma factors, possessed non‐synonymous mutations (Figure 1d, Table S2). In addition, the genes related to biotic stress, lipid degradation, nitrilases, proteases, G‐protein signalling genes, and the gibberellin (GA) biosynthesis genes, also had non‐synonymous mutations with amino acid alterations and are known to be responsive to oxidative stress (Table S2).

### Targeted‐gene association studies resulted in the discovery of novel allelic variations that reduced chalkiness

**Figure 2:**
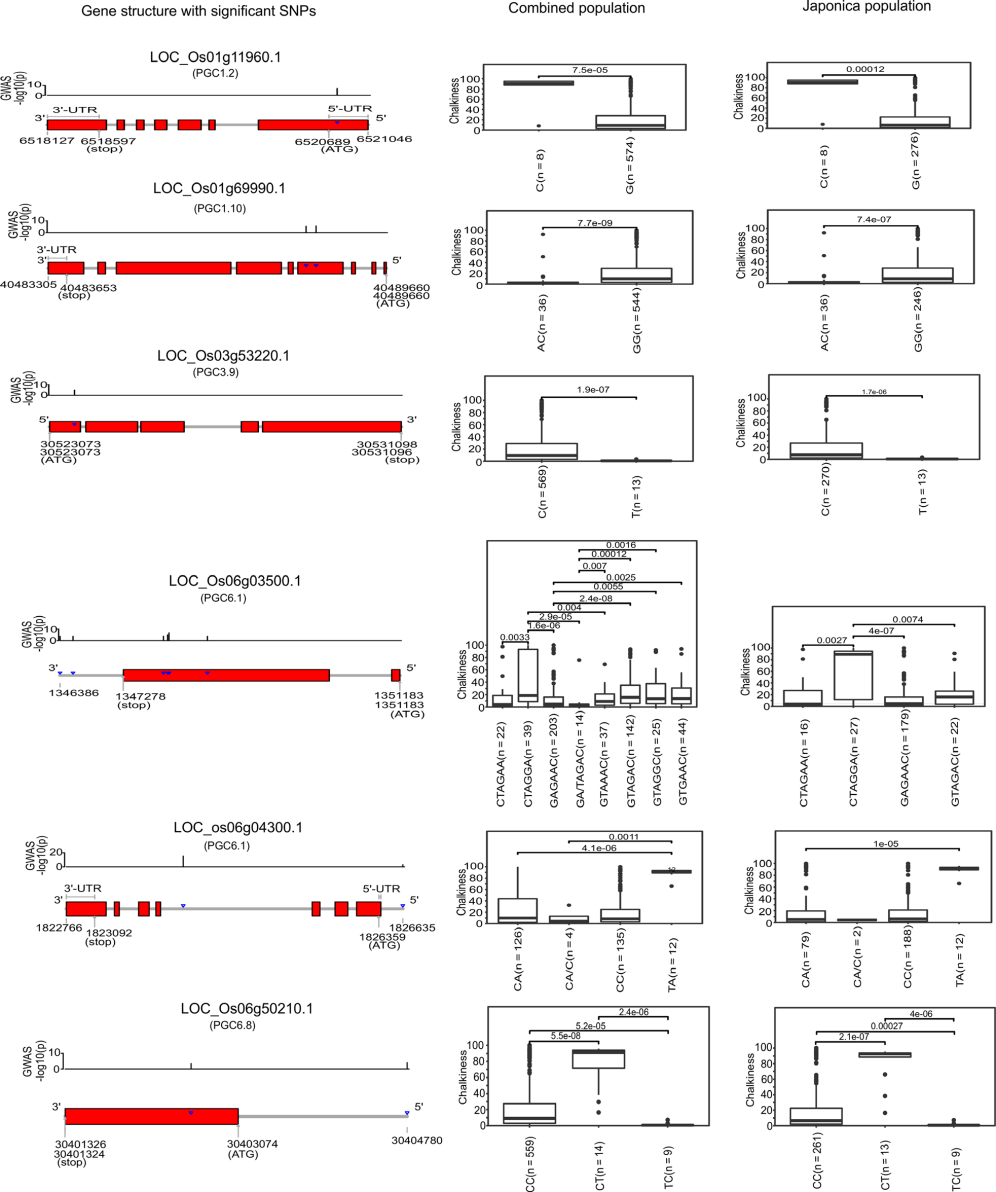
Targeted‐gene association analysis (TGAS) of chalk‐regulating QTLs identifies causal candidate genes from chalk QTLs exhibit moderate to high effect on the trait (phenotypic variance explained, PVE ≥ 10). Left section of the figure represents the respective gene structure model with ‐log_10_ *P* scale showing corresponding significance level of underlying allelic variants, just above the gene model. In the middle and right panel, boxplots represent the phenotypic distribution of haplotypes constructed from the allelic variants present in combined germplasm panel (left), and *japonica* panel (right), with number (n) of respective haplotype bearing accessions. Within boxplots, the pairwise significance level was tested using the Wilcoxon test and level of significance is specified.

**Figure 3:**
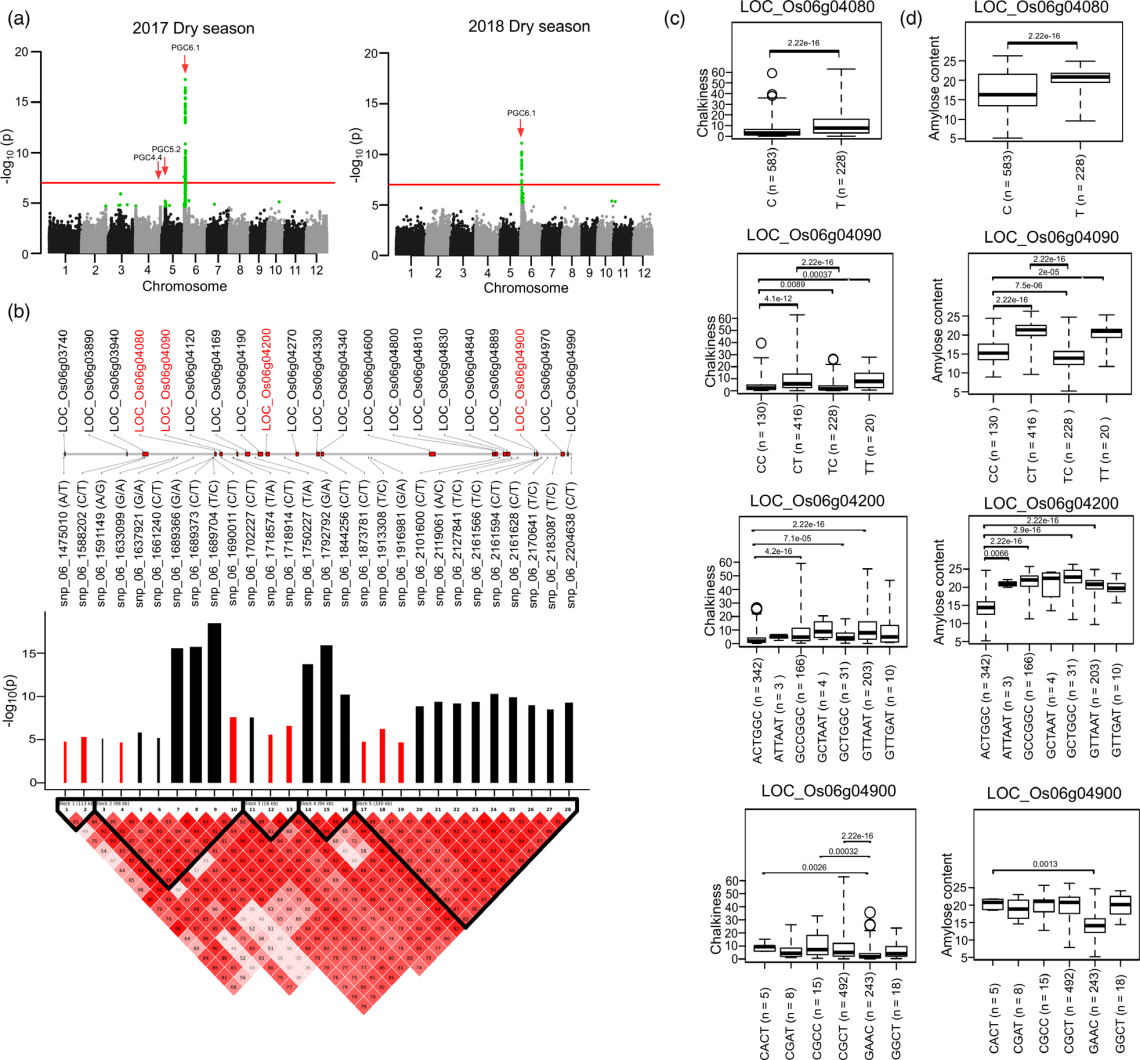
GWAS for PGC conducted in the MAGIC population. (a) Manhattan plot for per cent grain chalkiness (PGC) using MAGIC population grown in 2017 dry season and 2018 dry season identified genomic loci on chromosome 6. (b) Linkage Disequilibrium (LD)‐based plot showing 28 tagged SNPs distribute across 5 LD blocks with their positive (black bar) and negative (red bar) allele effect, where width of bar shows the allele effect size; candidates genes highlighted in red were selected for further study. Targeted‐gene association studies (TGAS) of LOC_Os06g04080, LOC_Os06g04090, LOC_Os06g04200 and LOC_Os06g04900, showing respective boxplots with phenotypic variation for grain chalkiness (c), and amylose content (d); within boxplots, the pairwise significance level was tested using the Wilcoxon test.

Targeted‐gene association studies (TGAS) were employed to identify the target genes located within the major QTL regions conferring lower PGC with higher PVE values in the diversity panel (Figure 2). The genomic region of *PGC1.2*, with a PVE of 26.7, negatively influenced the PGC. Within this region, the candidate gene LOC_Os01g11960 (histone lysine methyl transferase) was identified with snp_01_6520765 in the 5ʹ‐UTR region encoding the G allele, which confers a lower chalk level with a PGC of ~8.9 (Table 1, Table S2). The alternative allele SNP (carrying C; PGC ~ 90.6) notably distinguished the 8 entries found in the *japonica* from the remaining 574 entries, with the G allele (Figure 2). The genomic region of *PGC1.10* (PVE12.2) increases chalkiness. Within this genetic region, the GG haplotype that was predominantly represented in *japonica*, increased the chalkiness, while its alternative AC haplotype constructed from the genic region of LOC_Os01g69990 (expressed protein), caused synonymous variations and had a very low median PGC of ~1.5 (Figure 2). Within *PGC3.9* (PVE12.9), a synonymous SNP (C > T; snp_03_30523640 bp) in the genic region of the LOC_Os03g53220 (encodes ribonucleoprotein helicase) was identified. Lines with the C allele, which were abundant in *japonica*, had a PGC variance of ~9.5 and were clearly distinguished from the lines for the T allele, that conferred a very low PGC of ~0.64 (Figure 2). A high‐effect rare allele (C > T; allele frequency = 1.5%) in the upstream region of another candidate gene LOC_Os06g50210 (expressed protein) identified from the *PGC6.8* region, with a PVE of 20.5. Its derived haplotype (TC) was found to reduce the PGC to a very low level of ~0.64 in the diversity lines (Figure 2). Most importantly, within *PGC6.1*, that had the highest PVE of 27.53, a key haplotype G(A/T)AGAC derived including SNP causing a missense mutation (A > G; snp_06_1347924 bp) within the candidate gene LOC_Os06g03500 (encoding the NBS‐LRR disease resistance protein) had a median PGC of 2.9 and was abundantly represented in *indica* (Figure 2). Another candidate, 5 Mb apart within the *PGC6.1* region, LOC_O06g04300, harboured a set of SNPs in the upstream and intronic region, forming a haplotype (CA/C) that resulted in a low‐chalk phenotype with a median of ~4.4 (Figure 2). Within this haplotype, the key SNP (C > T; snp_06_1824110) converted the phenotype from a low to an extremely high‐chalk level and was found to be abundant in *japonica* (Table S2). The superior haplotype (GAAGACA) was identified in combination with both candidates (LOC_Os06g03500 and LOC_O06g04300) within *PGC6.1* and conferred lower levels of chalkiness of ~1.6%, in the IRRI176 and IRRI101 breeding lines (Figure S3). Notably, the *PGC6.1* genomic regions influencing the PGC were validated using the MAGIC population developed by intercrossing 8 diverse parents, covering the genetic variability from the *indica*, *japonica* and *aus* genomes (Figure 2a, b, Figure S4). The superior MAGIC lines with low chalkiness were found to possess the *PGC6.1*, *PGC4.4* and *PGC8.1* QTLs. Using the MAGIC population, we identified the importance of 4 genes (LOC_Os06g04080, LOC_Os06g04090, LOC_Os06g04200 and LOC_Os06g04900) in influencing the lower PGC levels, to help achieve premium grade rice production. Interestingly, these haplotypes were also found to have amylose content levels of >5% (Figure 3a‐d). These novel candidate genes with rare alleles conferring the lower PGC levels in the rice grains that were discovered from the diversity panels and MAGIC population will be useful resources and targets in breeding programs to lower chalkiness.

### Epistatic interactions identified the key regulators contributing to increased grain chalkiness

**Figure 4:**
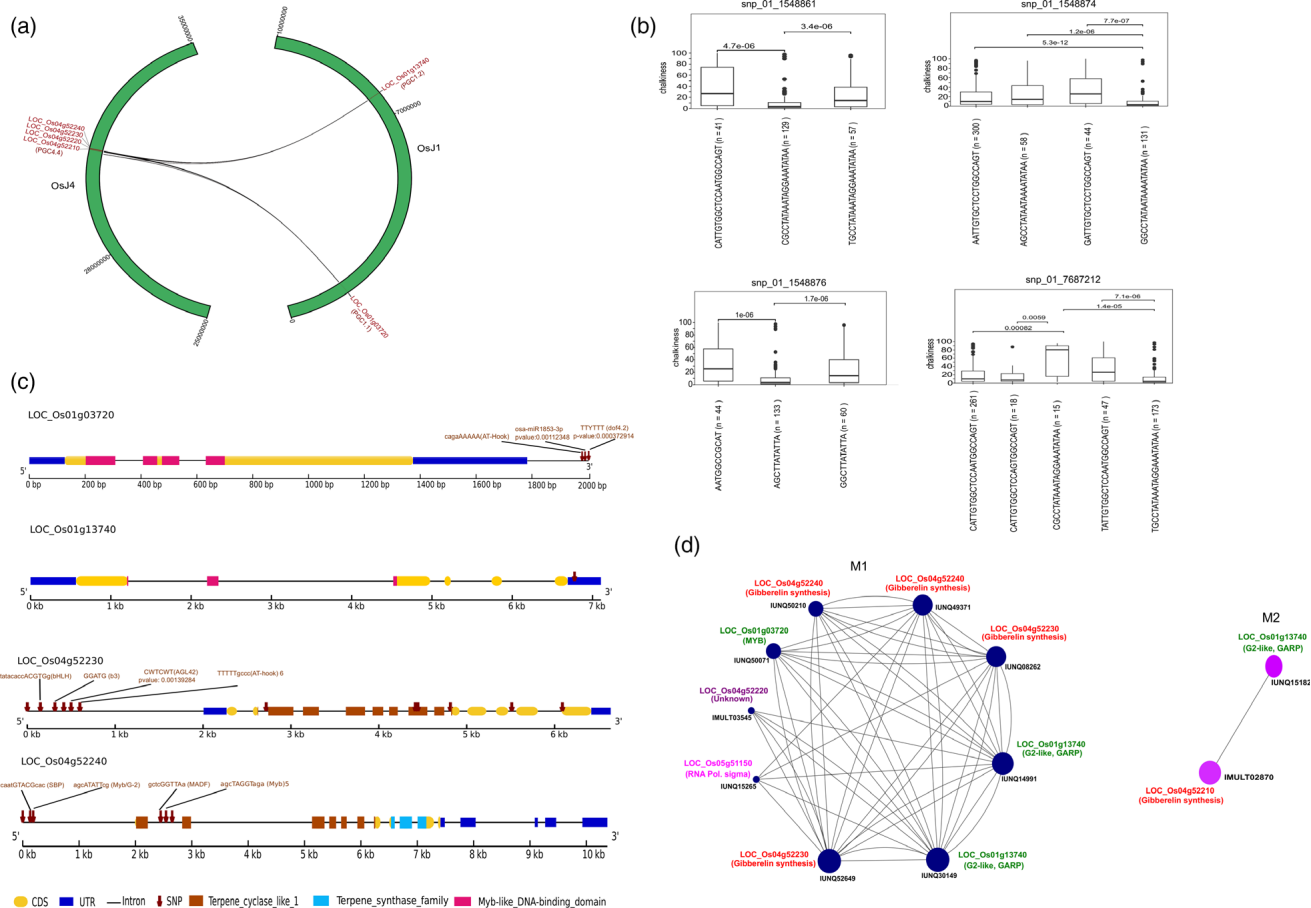
Epistatic interactions between chromosomes 1 and 4. (a) Two half circles (green) depicted the chromosomes 1 and 4 with scale of physical positions in base pairs (bps), and black lines signify the epistatic interactions between allelic variants lying on genes from chromosome 1 (LOC_Os01g03720; LOC_Os01g13740), and SNPs present in adjacent candidate genes from chromosome 4 (PGC4.4). (b) Haplotypes derived from a set of three SNPs (snp_01_1548861, snp_01_1548874, snp_01_1548876) lying within candidate LOC_Os01g03720 which exhibit epistatic interactions with SNPs from candidate genes of chromosome 4, its phenotypic variation for chalkiness was shown in box plots and pairwise significance values tested using the Wilcoxon test specified therein. (c) Gene structure models of candidate genes LOC_Os01g03720, LOC_Os01g13740 from chromosome 1, and LOC_Os04g52230 and LOC_Os04g52240 from chromosome 4 show the positions of SNPs along with underlying key functional domains. (d) Gene regulatory network derived from transcriptome data generated from contrasting chalk haplotype containing lines along with visualization of subnetwork of modules M1 (darskslate blue) and M2 (purple) with probes coding for target gene LOC_Os01g13740 (IUNQ30149, IUNQ14991); LOC_Os01g03720 (IUNQ50071); LOC_Os04g52240 (IUNQ49371, IUNQ50210); LOC_Os04g52230 (IUNQ52649, IUNQ08262); LOC_Os04g52220 (IMULT03545); LOC_Os05g51150 (IUNQ15265), LOC_Os04g52210 (IMULT02870), LOC_Os01g13740 (IUNQ15182). Interaction among the targets inside the module, each of the coloured solid circles represents node (oligo probes), the different size of nodes is based on its degree of connectivity and connecting lines represent weighted edges.

We conducted an epistatic interaction analysis using a linear regression model and identified 245 SNPs showing genome‐wide epistatic interactions, influencing the PGC (Figure 4, Figure S5, Table S3). For further details refer Appendix S2. The results revealed a substantial number of highly significant epistatic interactions between chromosomes 1 (*PGC1.1*, *PGC1.2*) and 4 (*PGC4.4*). Within *PGC1.1*, three moderate effect loci (snp_01_1548861, snp_01_1548874 and snp_01_1548876) were mapped to LOC_Os01g03720 (encodes for *MYB62* TF) and showed intensive epistatic interactions with the gene family members of ent‐kaurene synthase genomic loci (LOC_Os04g52210, LOC_Os04g52230 and LOC_Os04g52240), and LOC_Os04g52220 (unknown expressed) of chromosome 4 (*PGC4.4*) (Figure 4a‐b, Figure S5). Intriguingly, 3 SNPs in the 3ʹ‐regulatory region of the MYB62 TF gene (LOC_Os01g03720) were downstream gene variants that overlapped with the binding site for the AT‐hook TFs/proteins (cagaAAAA; snp_01_1548861), miRNA (miR1853; snp_01_1548874; *P*‐value = 0.00118) and Dof TF (dof 4.2; snp_01_1548876; *P*‐value = 0.00037) (Figure 4c). By combining the SNPs contributing to the epistatic interactions between *PGC1.1* and *PGC4.4*, the functional haplotypes were derived, showing significant phenotypic variations for PGC and suggesting that their shared contributions resulted in the increased chalkiness (Figure 4b). In addition, the key interacting candidate genes from *PGC4.4*, including LOC_Os04g52230 and LOC_Os04g52240, had binding sites for the key TFs, including the bHLH, At‐hook, B3‐domain TF, MYB TF/G2‐like and MYB5, suggesting that the underlying interactions that regulate PGC may belong to the complex TF‐based regulations (Figure 4c). Candidate genes LOC_Os04g52230 and LOC_Os04g52240 also appeared as top genes when PGC4.4 is subjected to TGAS (Figure S6). In addition, a distant genomic locus (snp_01_7687212) in the 3ʹ‐UTR variant of the candidate LOC_Os01g13740 (encoding a G2‐like MYB TF) from chromosome 1, represented the *PGC1.2* region, was shown to have epistatic interactions with the same loci of *PGC4.4* (LOC_Os04g52210, LOC_Os04g52220, LOC_Os04g52230 and LOC_Os04g52240) from chromosome 4, with a collective PVE of 17.4 (Figure 4a, c, Figure S5, Table S3). The variants found in the GA biosynthesis genes within *PGC4.4*, are likely to be mediated by two different MYB TFs (*MYB62* and *G2‐like MYB*). The MAGIC population (Figure S4) confirmed the contributions of the haplotypes derived from LOC_Os04g52230 and LOC_Os04g52240 on chromosome 4 in conferring lower chalkiness (Figure S7). However, the epistatic contributing alleles from *PGC1.1* and *PGC1.2*, that were detected in the diversity panels, were absent in the MAGIC population. The RNA polymerase sigma factor (LOC_Os05g51150) from the *PGC5.8* QTL showed epistatic interactions with the two gene family members of the ent‐kaurene synthases (Figure S5, Table S3), likely contributes to intermolecular epistasis regulations.

Since epistasis can represent a component of the gene interactions that drive transcription through TF‐based regulations, we tested the genome‐wide expression profiles obtained from the microarrays of 76 lines that were identified to possess superior and inferior functional haplotypes, derived from the GWAS‐coupled epistasis interactions [MYB62 (*PGC1.1)*, G2‐like MYB TF (*PGC1.2)* with GA biosynthesis genes located in *PGC4.4*]. The underlying dynamic relationships between the genes were identified using the derived gene regulatory networks influencing the PGC. The genome‐scale co‐expressed network construction resulted in a total of 47 modules (Figure S8). Of these, the M1 module encompassed most of the epistasis target genes identified from the GWAS‐based QTLs (*PGC1.1*, *PGC1.2* and *PGC4.4*), with significant edge weight and a higher degree of connectivity (Figure 4d, Figure S8, Table S4). In module M1, the target gene LOC_Os01g13740 *(G2‐like MYB* TF, *GARP*) was found to have multiple interacting partners with: (a) the key regulators of LOC_Os01g03720 *(MYB62)* from the *PGC1.2* QTL, LOC_Os05g51150 (RNA polymerase sigma factor) from the *PGC5.8* QTL, and (b) other tandemly duplicated gene family members encoding ent‐kaurene synthases (*LOC_Os04g52230*, *LOC_Os04g52240)* and the *LOC_Os04g52220* (unknown) target genes from the *PGC4.4* QTL (Figure 4d, Table S4). Likewise, the *MYB62* TF (*LOC_Os01g03720)* interacts with the G2‐like MYB TF (*LOC_Os01g13740*) and gibberellin synthesis genes (*LOC_Os04g52230*, *LOC_Os04g52240*). Module M2 contains one of the target epistasis genes, LOC_Os04g52210, encoding different gene family members of the ent‐kaurene synthase from the *PGC4.4* QTL (Figure 4d, Figure S8, Table S4). Taken together, the genome‐wide perturbations identified from the GWAS that impacted on the PGC trait were independently validated through gene regulatory networks, based on the co‐expressed transcriptome data. These results confirmed the importance of MYB62, G2‐like MYB TFs, and the RNA polymerase sigma factor, in regulating the tandemly duplicated ent‐kaurene synthases from *PGC4.4*, either as complexes or individually (Figure S5).

### Genetic regions conferring low chalkiness identified through GWAS‐epistatic interactions

As a result of the GWAS, the *PGC6.1* region, which had the highest PVE of 27.53 for conferring lower chalkiness, was mapped to the interval between 1.23–1.87 Mb (Table 1), with 6 LD blocks showing a negative effect on PGC (Figure S9a‐b). This region was co‐located with the granule‐bound starch synthase I (*GBSS I*) gene. Markedly, several SNPs (snp_06_1227791, snp_06_1258271, snp_06_1347924, snp_06_1261311, snp_06_1246170, snp_06_1259666, snp_06_1259614 and snp_06_1262389) within the *PGC6.1* region exhibited epistatic interactions (Figure S9c). There are 4 SNPs harboured on LOC_Os06g03310 (expressed unclassified), including two prominent SNPs (snp_06_1259614, Beta −0.65; snp_06_1259666, Beta −0.66) with missense variants (snp_06_1259614 Ile > Asn; snp_06_1347924, Tyr > His) that regulate the chalkiness phenotype (Figure S9a, c, Table S2).

**Figure 5:**
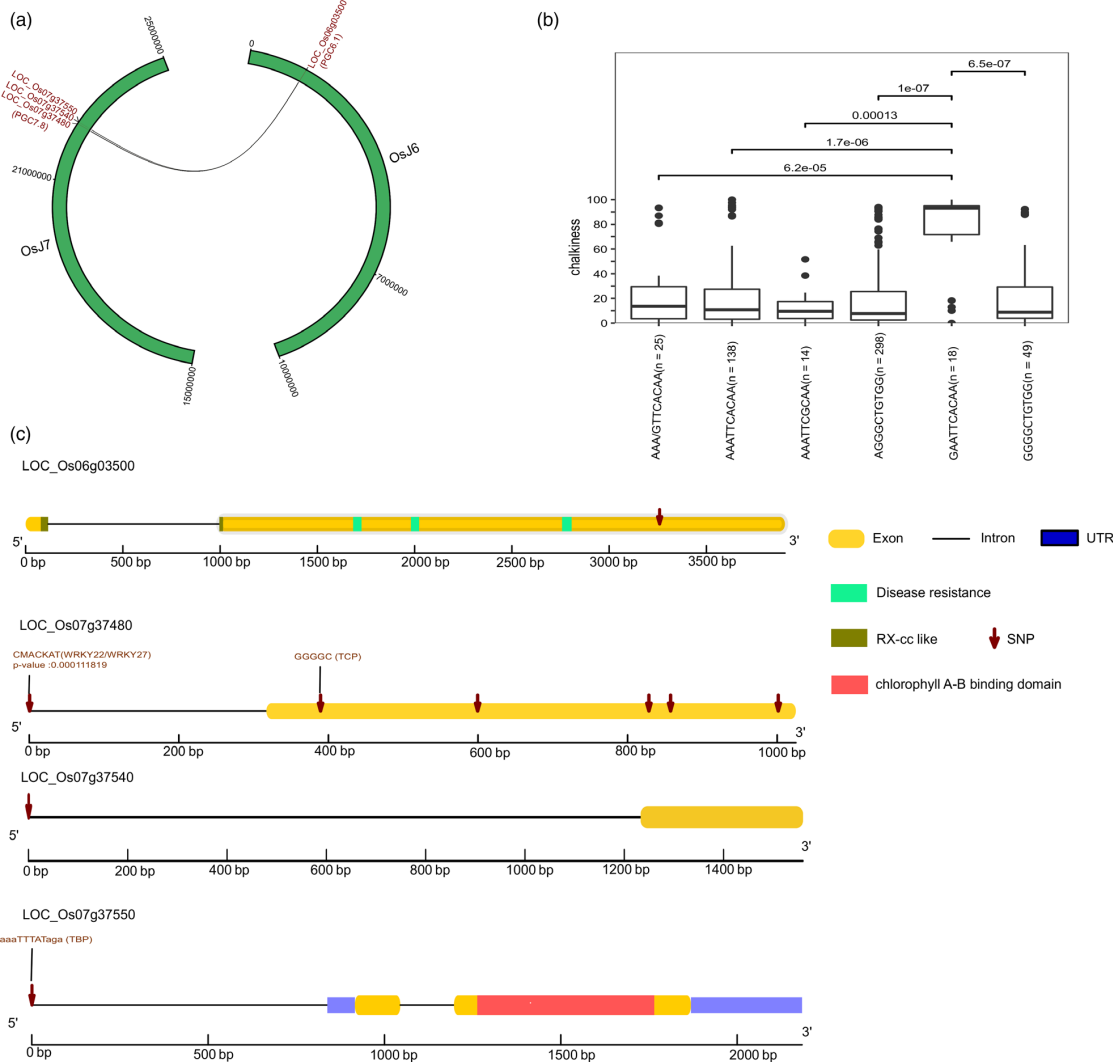
Epistatic interactions exist between chromosomes 6 and 7. (a) Chromosomes 6 and 7 are shown using the semicircular structure with scale of physical positions in base pairs (bps), which indicate the epistatic interaction of a locus from LOC_Os06g03500 on chromosome 6 (*PGC6.1*) with three loci from adjacent candidate genes from chromosome 7 (*PGC7.8*). (b) Combination of SNPs from candidate genes from *PGC6.1* and *PGC7.8* utilized to construct haplotypes, which show the phenotypic variation for PGC in the combined panel. Pairwise comparison was highlighted with the significance level as assessed by Wilcoxon test. (c) Gene structure models of candidate genes LOC_Os06g03500, LOC_Os07g37480 and LOC_Os07g37550 showing the positions of SNPs along with its key functional domains. Candidate gene lying on chromosome 7, that is LOC_Os07g37480 harbours binding sites for WRKY22/WRKY27 (*P* = 0.00011) in 5′‐upstream regulatory region, whereas, LOC_Os07g37550 exhibits binding site for the TATA‐binding protein (TBP) in 5′‐upstream region.

A significant SNP, snp_06_1347924, created a missense variant in the candidate gene LOC_Os06g03500 (encoded NBS‐LRR domain) from *PGC6.1* of chromosome 6 (PVE = 27.53) and exhibited a number of prominent epistatic interactions with 9 SNPs on chromosome 7. The epistatic interactions were established using 9 key SNPs falling on LOC_Os07g37480 (expressed unclassified), LOC_Os07g37540 (expressed unclassified) and LOC_Os07g37550 (photosystem II protein) from *PGC7.8*, showing a collective PVE of 13.6 (Figure 5a‐c, Table S2). Of the 9 interactive SNPs, notably, 7 SNPs (including 5 SNPs creating missense variants, and 1 in the 5ʹ‐UTR region) were detected alone in LOC_Os07g37480, while the remaining two SNPs covered two different neighbouring genes, LOC_Os07g37540 (unclassified) and LOC_Os07g37550 (photosystem II protein) (Figure 5a‐c, Table S2). On LOC_Os07g37480, an SNP overlapped with the binding site of a WRKY TF, while on LOC_Os07g37550, an interacting SNP was observed in the binding site for the TATA‐binding protein (TBP), suggesting that there was a regulatory importance to these interactions (Figure 5c). The candidate genes LOC_Os07g37540 and LOC_Os07g37550 were further validated to confer lower chalkiness in IRRI172 and IRRI174 (Figure S4). SNPs from the *PGC6.1* and *PGC7.8* regions were combined to derive the functional haplotype AGGGCTGTGG, which had the lowest median PGC of 7.65 (Figure 5a, b).

The second genomic region of *PGC6.8* was mapped to a 0.8 Mb region (30.2–31 Mb) of chromosome 6, exhibiting a PVE of 20.6, and contained a total of 107 SNPs, that showed interactions primarily with *PGC7.8* and minor interactions with *PGC1.2* (Figure S5). In addition, the single SNPs from *PGC1.9* (snp_01_34329540) and *PGC3.7* (snp_03_26840782) showed epistatic interactions with multiple SNPs in the *PGC7.8* genetic region (Figure S5, Table S2).

## Discussion

### Unravelling the novel genetic variants influencing low chalkiness in rice that were identified using a GWAS and epistatic interactions

Farmers sell their harvested paddies to millers, who avoid harvests with high levels of chalky rice grains, as they are predisposed to breakage during the milling and polishing processes (Butardo and Sreenivasulu, 2019). Thus, any undesirable traits, such as an increase in chalkiness that directly affects the premium, need to be selected against by the breeders in developing varieties. The present study identified 21 major QTLs and 5 significant epistatic interactions that explain the high PVE impacts on grain chalkiness. Several of the QTLs/genes that were detected in this investigation (*PGC5.2*, *PGC6.1* and *PGC7.8*) have previously been reported (Gong *et al*., 2017; Misra *et al*., 2019; Shomura *et al*., 2008; Sun *et al*., 2013; Weng *et al*., 2008). Notably, however, 18 additional novel and high impact QTLs were identified on different chromosomes that regulate PGC (Table 1, Table S1). Their potential epistatic interactions contributed to the identification of gene‐gene interactions (Zuk *et al*., 2012). This strategy effectively allowed for the capture of high‐effect genome‐wide multi‐locus epistatic interactions, either within the QTL region or across the QTLs, that influence chalk at the subspecies level, and are evolutionarily diverged in distinct population structures. Functional categories associated with PGC QTLs of high PVE observed using GOMapman reinforced the previously identified roles key metabolic pathways including Myb transcription factors (Liu *et al*., 2010) and lipid metabolism (Kaneko *et al*., 2016) in regulating grain chalkiness. Aside from identifying, the major QTLs that explain the higher PVE through combined GWAS and epistasis, novel functional haplotypes were defined from the interacting QTLs of *PGC6.1* (PVE = 27.53) and *PGC7.8* (22.4–22.5 Mb region), and these were found to contribute to reducing the chalkiness to less than 2%. Such novel regions need to be utilized in breeding programs to lower chalk levels, and in particularly when exploring the heterosis created from the inter‐subspecies crosses of *japonica* and *indica*.

### Dissecting the causal effects of lowering chalkiness from the pleiotropic effect of amylose content (AC) within *PGC6.1*

In the *japonica* subspecies, waxy and low amylose content are correlated with increased chalkiness, and thus mutations in *gbssI* were correlated with increased chalkiness in the *japonica* population (Quero *et al*., 2018; Zhao *et al*., 2015). In congruence, the white core expression (*qWCE6*) chalk QTL was mapped to chromosome 6, and co‐localized with the waxy loci of *GBSSI* in a combined BC_2_F_6_ mapping population, made from *japonica* and *indica* crosses (Okada and Yamasaki, 2019). In addition, this chalk genetic region was confirmed in the GWAS conducted with the hybrid crosses of the F_2_ population (Gong *et al*., 2017). Interestingly, in this investigation of QTL *PGC6.1*, we identified two important genes, LOC_Os06g03500 and LOC_os06g04300 (Figure 2), that are 415 kb upstream and 52 kb downstream of *GBSSI*, respectively, shown to confer lower chalkiness levels and thus premium quality characteristics. The combined haplotype derived from the genic regions of LOC_Os06g03500 and LOC_Os06g04300 validated their ability to confer low chalkiness in the IRRI101 and IRRI176 breeding lines (Figure S3). Interestingly, none of the key alleles reported previously to influence amylose content at the *GBSSI* (*Wx*) locus (Anacleto *et al*., 2019; Butardo *et al*., 2017; Misra *et al*., 2018) were associated with the chalk phenotype in the diversity panel (Table S2). In the MAGIC populations, we identified haplotypes that lowered the chalk in the *GBSSI* gene as well as in the neighbouring loci LOC_Os06g04080, LOC_Os06g04090 and LOC_Os06g04900, which also affects the amylose content. The MAGIC lines that exhibit low chalkiness in the low amylose backgrounds are a valuable resource. These results reinforce the perspective that genes present near to *GBSSI* in *PCG6.1* could be targeted in *japonica* breeding programs to achieve lower chalk levels in a low amylose background.

### Impact of grain‐size traits on grain chalkiness

Grain size‐related traits, such as grain width, are known to confer unfavourable pleiotropic effects on grain chalkiness; thus, grain width was found to be positively correlated with increases in PGC (Zhao *et al*., 2015). Previous studies reported that some QTLs governing grain width (*qGW5*) and PGC (*qPGWC5*) were overlapped (Wang *et al*., 2017). Additional reports have highlighted that chalk QTLs were overlapped with grain width‐related genes. Interestingly, in the present study, of the reported 78 chalk QTLs, only the *PGC5.2*, *PGC6.1* and *PGC7.8* QTL regions overlapped with the known grain width governing genes (Table S2) (Gong *et al*., 2017; Misra *et al*., 2019; Shomura *et al*., 2008; Sun *et al*., 2013; Weng *et al*., 2008). Although the *PGC5.2* region interval possesses a known candidate gene for grain width, *GW5*, it did not directly signify association with the chalk phenotype. Strikingly, genes present upstream of *GW5* and 4 SNPs‐mapped downstream of LOC_Os05g09520 were associated with PGC (Table S2), among which one genetic variant present in the *PGC5.2* region showed epistatic interactions with the *PGC6.8* QTL (Table S3). Previously, we used deeper analysis in the *indica* germplasm and fine‐mapped *PGC5.2* to an uncharacterized unknown protein, responsible for influencing chalk, and present upstream to the *GW5* (Misra *et al*., 2019). Another gene, *GS6*/*D62* (LOC_Os06g03710), regulates grain shape (Sun *et al*., 2013), coinciding with the chalk QTL *PGC6.1*, but again *GS6* did not show any direct association with the chalk trait (Table S2). The *GW7* on chromosome 7, using the GWAS of F_2_ lines and recombinant inbred populations, was developed from parents with long slender grains that were not chalky and those with greater grain widths were chalky (Chen *et al*., 2016; Gong *et al*., 2017). Although the *qGW7* QTL region was identified in previous studies to regulate grain width (Gong *et al*., 2017; Misra *et al*., 2017), it was overlapped with the chalk QTL *PGC7.8* (22.9‐23.3Mb) in the present study, and the fine‐mapped target gene *LOC_Os07g41200* (*GW7*) did not signify any association with the chalk trait. In the diversity panel, the phenotypic variation for the PGC and grain width showed a weak positive correlation (~0.30) (Table S5). Taken together, our current results reinforce the conclusion that the key genes influencing grain width are not causal factors triggering chalkiness, and careful finer resolution studies using high‐density SNPs can separate the pleiotropic effects between the grain width and chalkiness.

### GWAS‐coupled epistasis interactions and gene regulatory networks identified the key regulatory complexes impacting chalk

The genome‐wide SNP mutations impacting chalk in the diversity panels were explored using GWAS‐based epistasis interactions and further validated the genetic network through transcriptome‐based gene regulatory networks. These complementary approaches resulted in the identification of two different MYB domain TFs [LOC_Os01g13740, *MYB62* located in *PGC1.1* and *G2‐like* TF (LOC_Os01g13740) located in *PGC1.2*] that epistatically interacted with several tandemly duplicated ent‐kaurene synthase genes (located on *PGC4.4*) belongs to gibberellic acid biosynthesis (Figure 4) impacting grain chalkiness. The gene regulatory network confirmed the gene interactions of *MYB62* and the *G2‐like* TFs with the ent‐kaurene synthase gene family members. Notably, the presence of the Myb/G2‐binding sites in the upstream regulatory region of the ent‐kaurene synthase gene (LOC_Os04g52240 from *PGC4.4*), suggests that there was overlapping of the DNA‐binding sites for the two MYB proteins (Figure 4). Furthermore, the relevance of the key candidates (*LOC_Os04g52230* and *LOC_Os04g52240*) from the *PGC4.4*, to impact on chalkiness was validated using the MAGIC population. G2‐like transcription factors belong to the GRAP subfamily of the MYB TF family in plants (Powell *et al*., 2012). Some of its members are also known to be involved in flowering, by affecting the photoperiodic pathway (Zeng *et al*., 2018). *MYB62* is characterized to mediate gibberellin signalling during flowering and growth in Arabidopsis (Gocal *et al*., 2001). Intriguingly, the *MYB* domain‐containing gene from *PGC1.1* matching *MYB62* has been demonstrated to affect inorganic phosphate homeostasis in a gibberellin‐dependent manner in rice (Gu *et al*., 2017). Markedly, with a lack of phosphate, rice grains exhibit an increase in chalkiness (Zhang *et al*., 2012). Taken together, these findings suggest that *MYB62* is likely to be involved in phosphate homeostasis, regulates GA‐biosynthetic genes from *PGC4.4* and contributes to an increase in chalkiness. In addition, the G2‐like TF gene epistatically interacts with the *MYB62* from *PGC1.1*, as well as the GA biosynthesis genes from the *PGC4.4* region (confirmed from the GWAS‐epistasis and gene regulatory networks), suggesting a potential link to the complex intermolecular epistasis interactions that contribute to an increase in grain chalkiness.

## Conclusions

The genome‐wide genetic interactions were identified by utilizing the synergy of statistical epistasis and association mapping and revealed high‐value genetic regions that influence grain chalkiness in diverse lines. The *PGC* QTLs identified in the selected breeding and MAGIC populations, exhibited a low‐chalk phenotype. This two‐pronged strategy of GWAS coupled with epistatic and gene regulatory network analyses identified the following epistatic interactions: *PGC1.1* and *PGC4.4*, and the impacts of these QTLs on chalkiness, and the *PGC6.1* (with higher PVE) contributions to lower grain chalkiness in low amylose varieties. In addition, our results distinguished the pleiotropic effects of grain‐size genes from the causal‐associated genetic factors impacting chalkiness. These genome‐wide superior and favourable alleles need to be deployed in future breeding programs to promote lower levels of the undesirable chalkiness trait, especially in hybrid breeding programs generated from intercrosses, to ensure higher economic benefits for farmers.

## Methods

### Plant materials

A natural diverse panel was utilized, consisting of 307 *japonica* and 318 *indica* accessions that were identified from 3000 re‐sequenced rice genetic resources (The 3000 Rice Genomes Project, 2014; Wang *et al*., 2018). They were cultivated in a completely randomized block design with three replications, during the 2015 dry season, by employing standard crop management practices (Misra *et al*., 2017) at the Zeigler Experimental Station, International Rice Research Institute (IRRI), Laguna, Philippines (14°10′N, 121°15′E). A set of 92 breeding lines, which have been developed by IRRI‐breeding program from 1965 till date were grown during the 2015 wet season and 2016 dry season, using the same standard crop management practices as described above. For validation purposes, an S6 derived fixed Heat‐MAGIC population, encompassing 834 progenies and 8 founder parents, were grown in field during the 2017 dry season, following an augmented design at Zeigler Experimental Station in IRRI, Laguna, Philippines, using the same standard crop management practices as described above, and a subset of 419 progenies and founders that were grown in the 2018 dry season. The harvesting was performed manually, when the grains had attained their optimum moisture content (MC) between 22%–24%, and they were subsequently threshed, and dried using the flat bed driers at IRRI until the MC reached 12%–14%. The dried seeds were stored in brown paper bags in a storage room maintained at 18 °C.

### Phenotyping for the measurement of the percent grain chalkiness

From each cultivar, 50 g of seed material, with three independent replicates, was obtained and subsequently estimated for grain chalkiness using the SeedCount SC5000 Image Analyzer, following the standard and optimized protocols (Next Instruments, NSW, Australia) to determine the PGC. Using the designated sample trays, grains were scattered onto the wider wells, to ensure that the grains were proportionally represented prior to the analyses. After scanning the tray, the PGC data were estimated using the built‐in software after proper calibration, following the manufacturer’s instructions.

### Assessing the population structures using principal component analysis

The re‐sequenced genomic resources that were generated from the 307 *japonica* and 318 *indica* germplasm were utilized for SNP‐calling against the Nipponbare reference genome (MSU version 7). We used variant call format (vcf) files generated from the pipeline of the 3K‐resequencing data for processing the genotyping data. We filtered individual samples, ensuring a Phred score of ≥30, and then merged all samples together. Subsequently, we identified SNPs from the merged vcf files. Genotypic files were subjected to a filtration criterion as follows: (a) alleles possessing a missing rate of not more than 1% at the individual genotype and SNP level, and (b) minor allele frequencies of ≥1% were retained. A filtering step based on pLink 2 resulted in the identification of a total set of 868 941 and 2 401 369 quality‐assured SNPs within the 284 *japonica* and 299 *indica* accessions. The underlying genetic variations of both sets of the germplasm panels were estimated using PCA, by accounting for the 868,941 SNPs in *japonica* and 2 401 369 SNPs from the combined germplasm panel, using the SNPRelate package in the R platform (Zheng *et al*., 2012); http://cran.r‐project.org). The genetic variations that were captured utilizing the first two principal components (PC1 and 2), were plotted in the PCA plot.

### Genome‐wide association study

In order to fulfil the data normalization requirements for the phenotypic data, the warped linear mixed models function was applied (Fusi *et al*., 2014). A single‐locus GWAS was performed using efficient mixed‐model association expedited (Kang *et al*., 2010), after incorporating the population structure and kinship as covariates. GWAS was initially run (using PC = 4) for the *japonica* panel (284 individuals) by associating the PGC phenotype with the genotyping data of the assured 868 941 SNPs, to reveal the associated significant genomic loci, with the criteria of the more conservative Bonferroni correction, set at −log_10_ (*P*) ≥ 7.24, (calculated using 0.05/m where m is the number of markers), and the FDR of *P* ≤ 0.05 (Benjamini and Hochberg, 1995). Likewise, GWAS was performed (using PC = 3), by utilizing the PGC phenotypes from a total of 583 accessions (after including the 299 *indica* and 284 *japonica* accessions), with the genotyping data of the 2 401 369 high‐quality SNPs, with the Bonferroni correction set as −log_10_ *P* ~ 7.68, and the FDR at *P* ≤ 0.05.

The genotype data of the MAGIC population were filtered with a 1% missing rate and 1% minor allele frequency, to maintain consistency for the panel with the 583 germplasm. The genotyping set accounted for 521 911 SNPs, from 826 samples, which were used for running the GWAS. The SNPs that retained an FDR of *P* ≤ 0.05 were investigated further to identify candidate genes.

First, the kinship matrix was calculated using the LDAK tool, and then an individual significant SNP set for each QTL region was used as the input for the PVE calculation (Speed *et al*., 2012). The calculated PVE was shown for all the QTLs, using the Circos tool (Krzywinski *et al*., 2009). Furthermore, the LD‐ plots were generated using a standard procedure that was previously described by Misra *et al*. (2017). Haploview (Barrett et al., 2005) was utilized to represent the LD blocks in the QTL hotpot regions. Furthermore, haplotypes were constructed by relying upon significant SNPs, lying in individual LD blocks. The haplotypes are represented in the boxplots with respective phenotypic distributions, utilizing an in‐house R‐script. In a pairwise comparison, the individual haplotypes representing the phenotypic distribution were assessed for their significance using the Wilcoxon test, and the corresponding p‐value between the respective pairs was highlighted using boxplots. The beta coefficient (or beta) refers to the effect size of a given allele on the respective phenotype and was obtained using the output of the GWAS as an effect. Furthermore, the narrow sense of heritability (*h* ^2^), which involves genetic variation, mainly due to additive genetic values, was calculated using a heritability R package (Kruijer *et al*., 2015).

### Targeted‐gene association analysis

All genes that overlapped with significant SNPs and had FDRs ≤ 0.05, were subjected to targeted association analysis. SNPs located in the 2000‐bp upstream, genic region and 1000‐bp downstream for each gene, were extracted and genotype files were made using the extracted SNPs. Association mapping was carried out using the EMMAx pipeline for each gene, based on the newly formed genotype data. The kinship matrix and PCA from the whole set of genotype data were used for the targeted associations. Significant SNPs were further identified and plotted on the gene structures, and the constructed haplotypes were represented in respective boxplots.

### Genome‐wide epistatic interaction analysis

To determine the potential genetic interactions among the different target genes/loci influencing the PGC, we conducted an epistatic interaction analysis using a linear regression model between the multiple loci in the diversity panel. Genome‐wide SNPs were identified after surpassing the basic FDR threshold criterion of *P* ≤ 0.05 (Benjamini and Hochberg, 1995), as identified in the single‐locus GWAS, and were subjected to whole‐genome epistatic interaction analysis using PLINK (Chang *et al*., 2015). Linear/logistic regression‐based tests were performed using PLINK v1.90 beta (PLINK2), where parameter epi1 was set to 1, to include all the possible epistatic interactions. This test used linear regressions to fit a model to estimate the significant interactions, using the following formula:

(1) Y=β0+β1gA+β2gB+β3gAgB

where A and B are two allele variants, gA and gB are allele counts; β0, β1 and β2 are regression coefficients, and β3 coefficients are tested for significance, for each allele pair (A, B). All interactions that surpassed the Bonferroni corrected *P*‐values (=1.80 E^‐07^) were considered a significant pair of interactions. The Bonferroni cutoff was calculated using the formula of 0.05/m, where *m* = number of tests (277 886 in the present analysis). Furthermore, the Cytoscape (Shannon *et al*., 2003) and Circos (Krzywinski *et al*., 2009) tools were employed to plot the significant epistatic interactions between the key significant SNPs within and across the chalk‐regulating QTL regions.

### Mining IRRI‐breeding lines for haplotypes

Leaf samples from 92 breeding lines were subjected to whole‐genome sequencing using the HiSeq X10 platform (Illumina). Paired‐end reads that were 150 bp in length, were generated at ~30× coverage. Low‐quality reads and adapter sequences were removed from the raw sequence reads. Furthermore, the raw sequence reads were mapped to the *japonica* reference genome (MSU version 7) and SNPs were further called using the BWA‐GATK pipeline (Li and Durbin, 2009; McKenna *et al*., 2010). The significant haplotypes represented in the *PGC* QTL regions were identified using pLink 2, the in‐house perl script, and identified as QTL^+^ lines.

### Weighted gene‐co‐expression network construction

Lines that had high and low chalkiness phenotypes were chosen, based on the contrasting haplotypes associated with *PGC4.4* (block 1, block 2, block 3 and block 4) and *PGC1.1* (snp_01_1548861, snp_01_1548874, snp_01_1548876 and snp_01_7687212). Sixteen days after fertilization, the seeds of 76 unique lines (71 high‐chalk and 5 low‐chalk) were subjected to RNA preparation, using the Qiagen RNeasy Plant Mini Kit. Samples with an integrity number of 7.0 or above were subjected to cDNA synthesis and cRNA labelling, using a single‐colour Low Input Quick Amp Labeling Kit. The labelled samples were hybridized to 60 K *indica* microarrays in a SureHyb chamber, and microarrays were scanned with an ozone barrier slide using a SureScan Microarray scanner, as described by Butardo *et al*. (2017). The quantified gene expression data were extracted using Agilent’s feature extraction software that was subjected to quantile normalization. The 24 223 probes were matched with 100% identity with the genes that were extracted from all 76 samples, and subjected to the WGCNA analysis developed by Langfelder and Horvath (2008) in R. Gene modules were obtained by using a dynamic tree cutting algorithm (Langfelder *et al*., 2007). To further analyse the module, we measured the eigengene of each module (MEs), which is the first principal component of a given module and merged those with a correlation of ≥0.75 using the mergeCloseModules function of WGCNA. Gene regulatory networks were visualized using Cytoscape (Shannon *et al*., 2003).

## Conflicts of interest

The authors declare no competing interests.

## Author contributions

G.M. conducted the GWAS, epistasis interactions and haplotype analyses. S.B. interpreted the GWAS and epistasis interaction data and drafted the manuscript. S.P. performed gene regulatory network analysis. R.K.S and H.L contributed to the development of MAGIC population and genetic data interpretation. F.L. and R.M. developed genotyping data of MAGIC population and genetic data interpretation. N.S. conceptualized the work, wrote the manuscript with contributions from S.B. All authors read and contributed to the revision of manuscript.

## Supporting information


**Figure S1** Phenotypic distribution for percent grain chalkiness in *Japonica* and *Indica* germplasm panel.
**Figure S2** Principal component analysis (PCA) performed in *Japonica* and combined germplasm panel.
**Figure S3** Mining haplotypes in the 92 breeding lines set for low chalkiness trait and their distribution across breeding lines.
**Figure S4** Distribution of 8 parental genotypes of MAGIC population along with 3000 germplasm lines.
**Figure S5** Genome‐wide epistatic interactions involved in regulating the PGC, established between key genomic regions.
**Figure S6** GWAS for PGC revealed the significant association on chromosome 4 within combined germplasm panel.
**Figure S7** GWAS conducted for PGC in the MAGIC population identified genomic loci on chromosome 4.
**Figure S8** Gene regulatory network created using the transcriptome data generated from contrasting chalk haplotype containing lines.
**Figure S9** GWAS for PGC revealed the significant association on chromosome 6 genomic regions within combined germplasm panel.

**Appendix S1** Discovery of chalk genetic regions through GWAS in Japonica panel.

**Appendix S2** Epistatic interactions that regulate PGC.

**Table S1** Significant QTLs (showing PVE≤10) identified for regulating PGC along with their physical position on chromosomes, underlying topmost SNP, and further details.
**Table S2** Details of total significant SNPs identified under chalk‐regulating QTLs with their significance level, effect, and their functional relevance.
**Table S3** Genome wide epistasis interaction targets (mentioned as region 1 and 2) identified from the GWAS genetic variants associated with grain chalkiness.
**Table S4** Co‐expressed gene regulatory networks of developing seeds derived from diversity lines containing contrasting chalk haplotypes. The interactions among target‐QTL containing epistasis genes located in Module (M1) and Module (M2).
**Table S5** Detail of germplasm used for the study with their phenotypic values for grain chalkiness and grain‐size parameters, along with their countries of origin.
