# Supplementary material for: Genome‐wide association coupled gene to gene interaction studies unveil novel epistatic targets among major effect loci impacting rice grain chalkiness
Source: Plant Biotechnol J. 2020 Dec 9;19(5):910–25. doi: 10.1111/pbi.13516 (PMC8131057; doi:10.1111/pbi.13516)
Supplement: Supplementary file 1 — Figure S1 Phenotypic distribution for percent grain chalkiness in Japonica and Indica germplasm panel. Figure S2 Principal component analysis (PCA) performed in Japonica and combined germplasm panel. Figure S3 Mining haplotypes in the 92 breeding lines set for low chalkiness trait and their distribution across breeding lines. Figure S4 Distribution of 8 parental genotypes of MAGIC population along with 3000 germplasm lines. Figure S5 Genome‐wide epistatic interactions involved in regulating the PGC, established between key genomic regions. Figure S6 GWAS for PGC revealed the significant association on chromosome 4 within combined germplasm panel. Figure S7 GWAS conducted for PGC in the MAGIC population identified genomic loci on chromosome 4. Figure S8 Gene regulatory network created using the transcriptome data generated from contrasting chalk haplotype containing lines. Figure S9 GWAS for PGC revealed the significant association on chromosome 6 genomic regions within combined germplasm panel. [file PBI-19-910-s003.pdf]

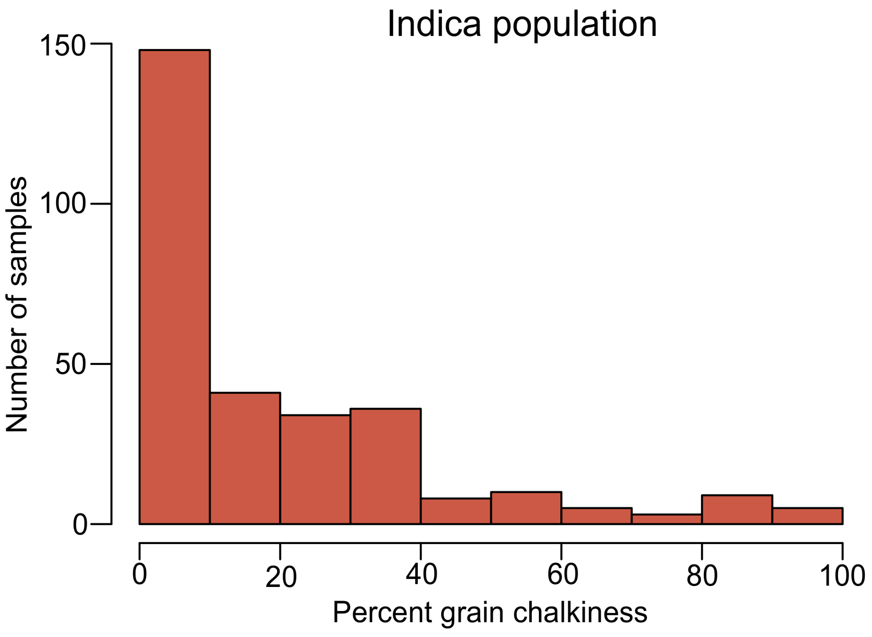

Figure S1: Phenotypic distribution for percent grain chalkiness in lines of *Japonica* and *Indica* subpopulation. X-axis of histogram represents percent grain chalkiness while y-axis defines total number of samples in respective subpopulation.

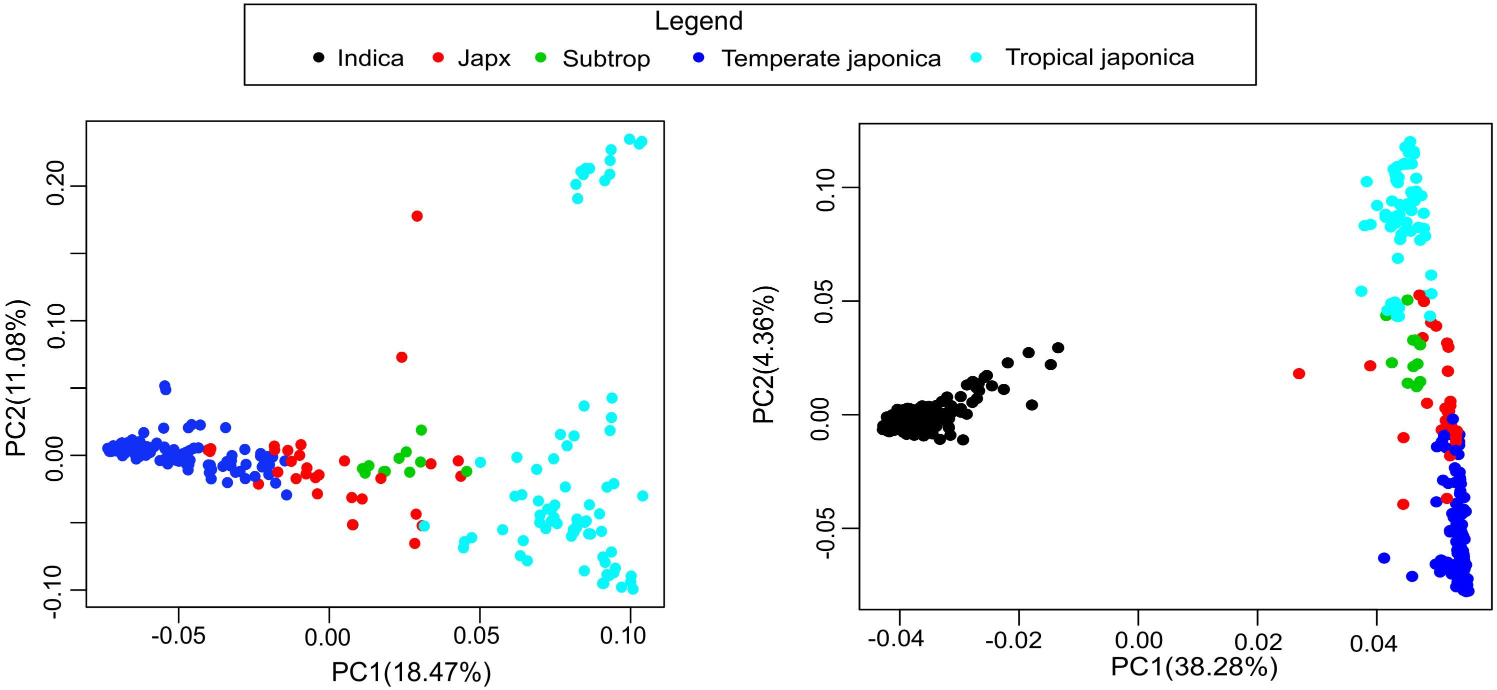

Figure S2: Principal component analysis (PCA) performed in *Japonica* and combined germplasm panel. In *Japonica* germplasm (left side), most variation present in *temperate Japonica*, *tropical japonica* and japx were captured by principal component (PC) 1, followed by PC 2, given both PCs contribute for a total of 29.55% variation. Likewise, in combined panel, PC 1 exhibits the highest variation (38.29%) which mainly distinguishes *indica* with *japonica* accessions; whereas, PC2 takes care of main variation (4.36%) exist within *Iaponica,* totaling up to 42.64% variation contributed by two top PCs.

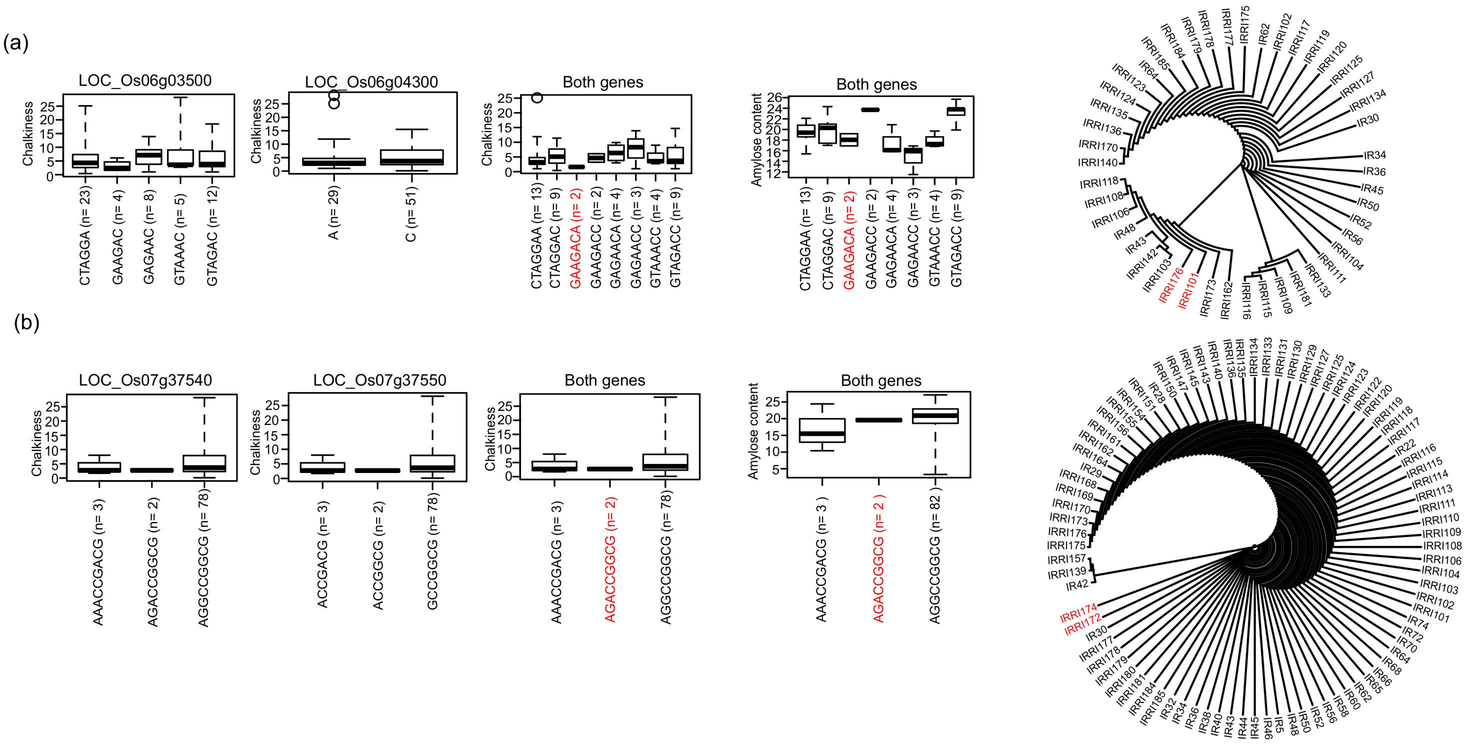

Figure S3: Mining haplotypes in the 92 breeding lines set for low chalkiness trait and their distribution across breeding lines. Haplotypes constructed from SNPs present within LOC_Os06g03500 and LOC_Os06g04300 (a), LOC_Os07g37540 and LOC_Os07g37540 (b), and both genes in combination from (a) and (b), along with their phenotypic variation for chalkiness and amylose content; on the extreme right, dendrogram constructed showing variation in IRRI varieties/breeding lines for the haplotypes showed in case of both genes. Haplotype highlighted in red refers to the superior haplotype identified after combining both genes.

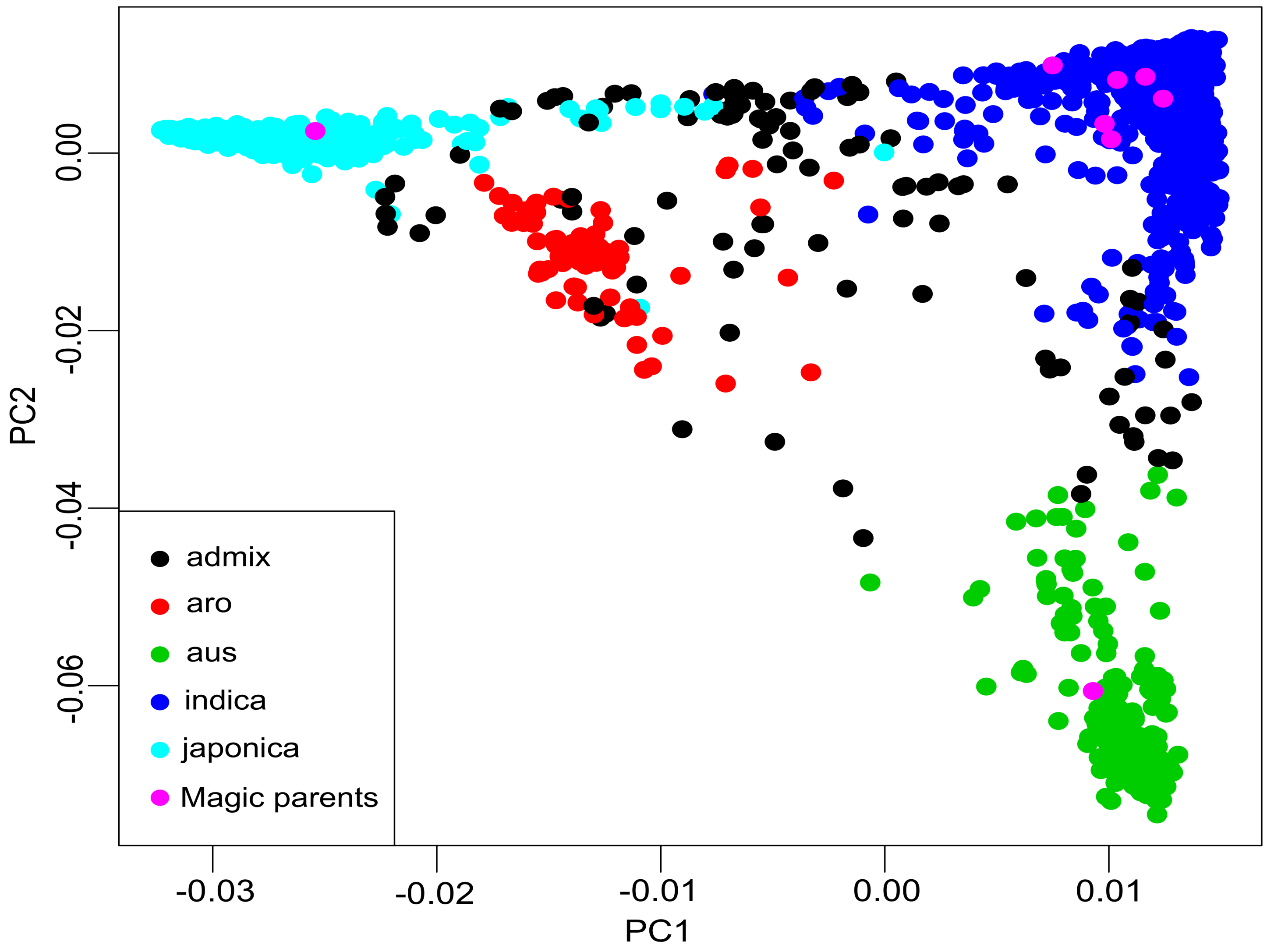

Figure S4: Distribution of 8 parental genotypes of MAGIC population along with 3000 germplasm lines. All of 8 parents of MAGIC population (MAGIC) were found distributed across the 3000 rice germplasm (source of 583 combined panel), mainly along with *indica, japonica,* and *aus* subpopulations.

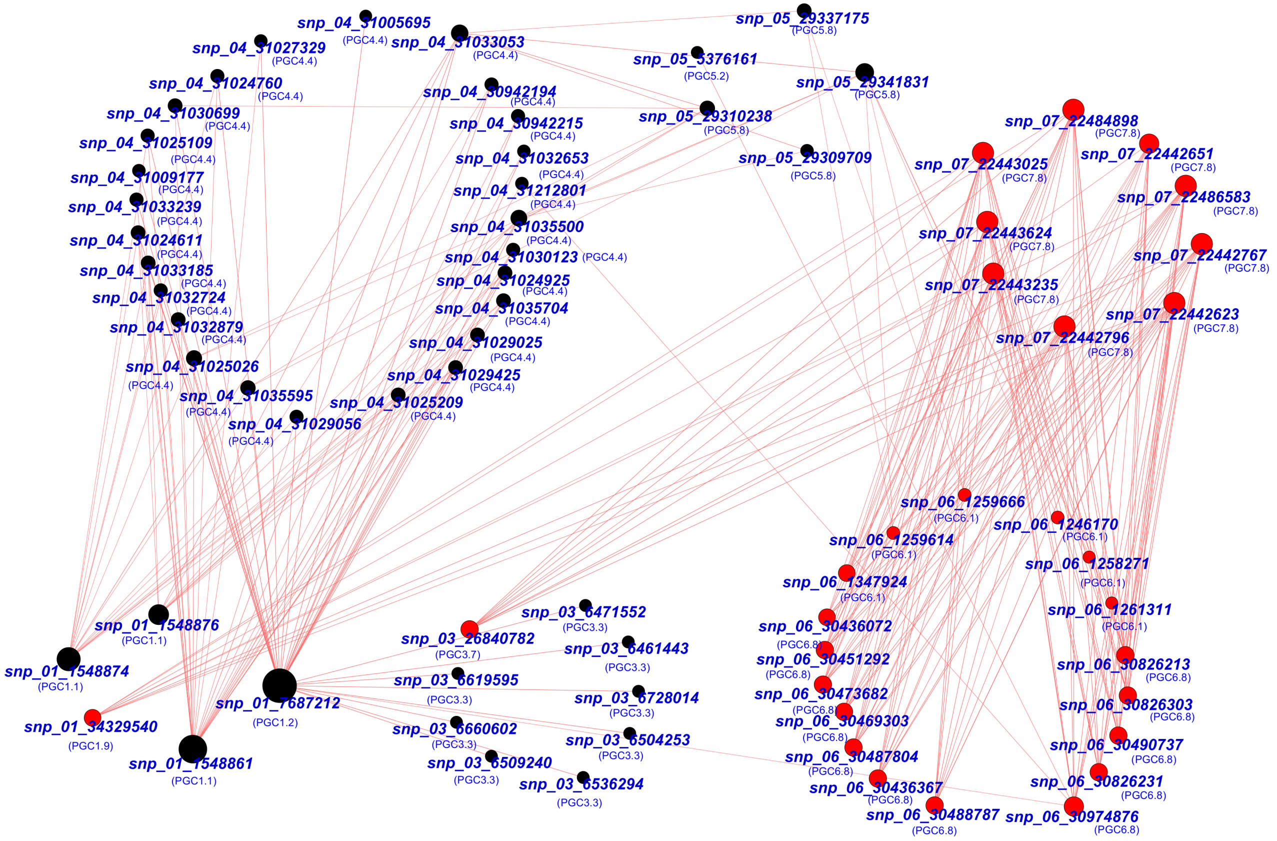

Figure S5: Genome-wide epistatic interactions involved in regulating the PGC, established between key genomic regions. Circles/nodes denote the genomic loci/SNPs, where size of node signifies effect of genomic loci on the trait; straight red line connecting two nodes depicts the epistatic interactions between them. Significant epistatic interactions were observed between chromosomes 6 (PGC 6.1 & PGC6.8) and 7 (PGC7.8), and chromosomes 1 (PGC1.1, PGC1.2, & PGC1.9) and 4 (PGC4.4), in addition to interactions observed independently from chromosomes 3 (PGC3.3 & PGC3.7) and 5 (PGC5.2 & PGC5.8). Notably, entire genomic loci involved in epistatic interactions from chromosomes 6 and 7, a locus from chromosome 1 (PGC1.9), and another locus from chromosome 3 (PGC3.7) signified the negative effect on the trait (red nodes; favouring lower chalkiness), whereas, entire loci from chromosomes 4 and most of chromosome 1 (PGC1.1 & PGC1.2) participating in epistatic interactions show the positive effect on the trait (black nodes; favouring higher chalkiness). Besides, rest loci from chromosome 3 (PGC3.3), and chromosome 5 (PGC5.2 & PGC5.8) exert positive effect on PGC.

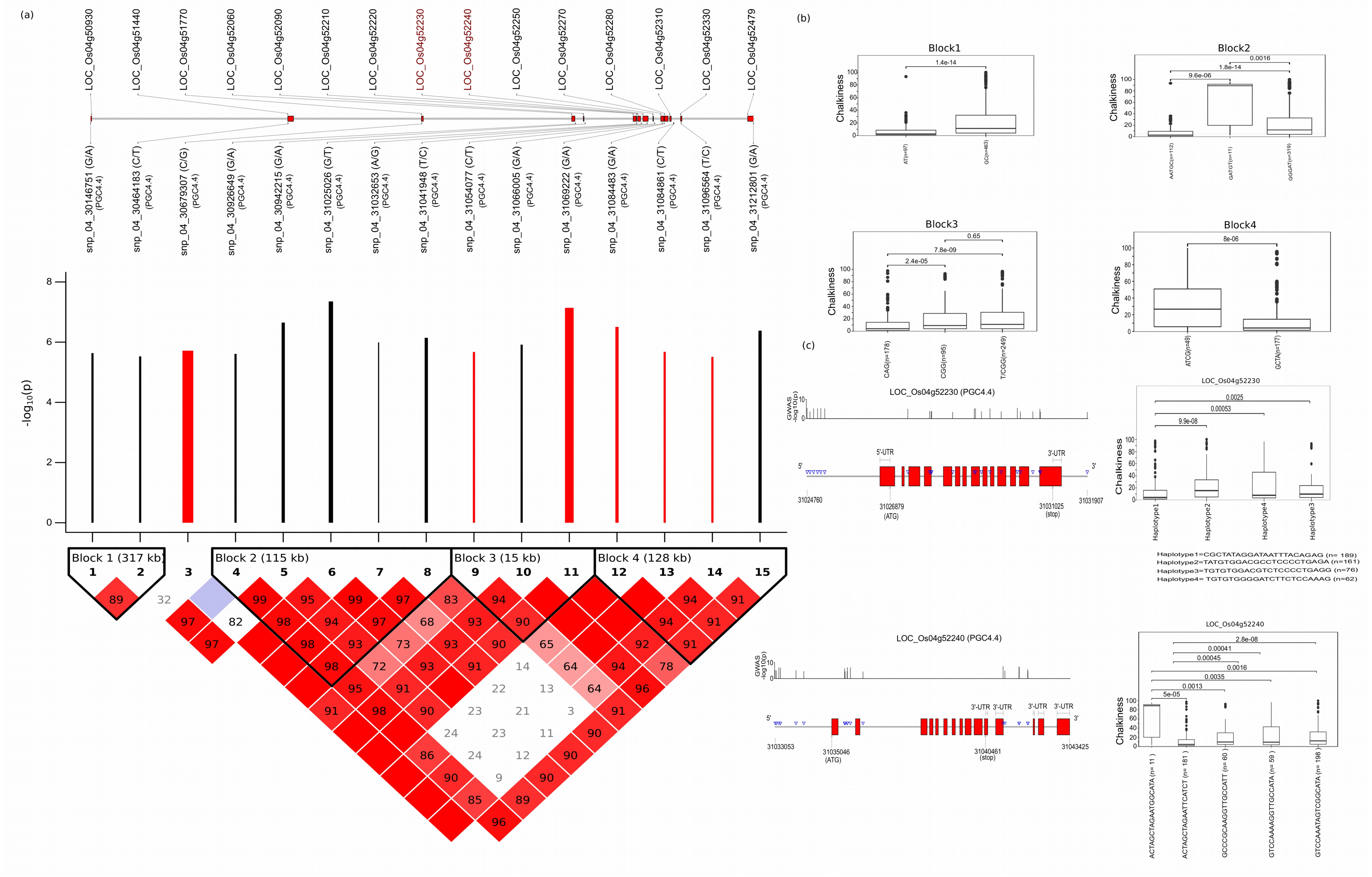

Figure S6: GWAS for PGC revealed the significant association on chromosome 4 within combined germplasm panel. (a) LD-plot containing 15-tag SNPs along with their respective QTL region (PGC4.4), within 4 LD-blocks on chromosome 4 along with their corresponding log_{10}-scaled P-values ($log_{10}(P)$), indicate significantly association with PGC. 6 out of 15 SNPs show negative effect (red bars), whereas rest show the positive effect (black bars) on PGC (b) Boxplots demonstrate the haplotypes constructed from a total of 4 LD blocks with contrasting phenotypic values for PGC; moreover, within boxplots, the pairwise significance level was tested using the Wilcoxon test, (c) Gene structure models of LOC_Os04g52230, and LOC_Os04g52240 genes showing underlying significant SNPs along with $-log_{10}(P)$ scale , and respective haplotypes constructed from these SNPs.

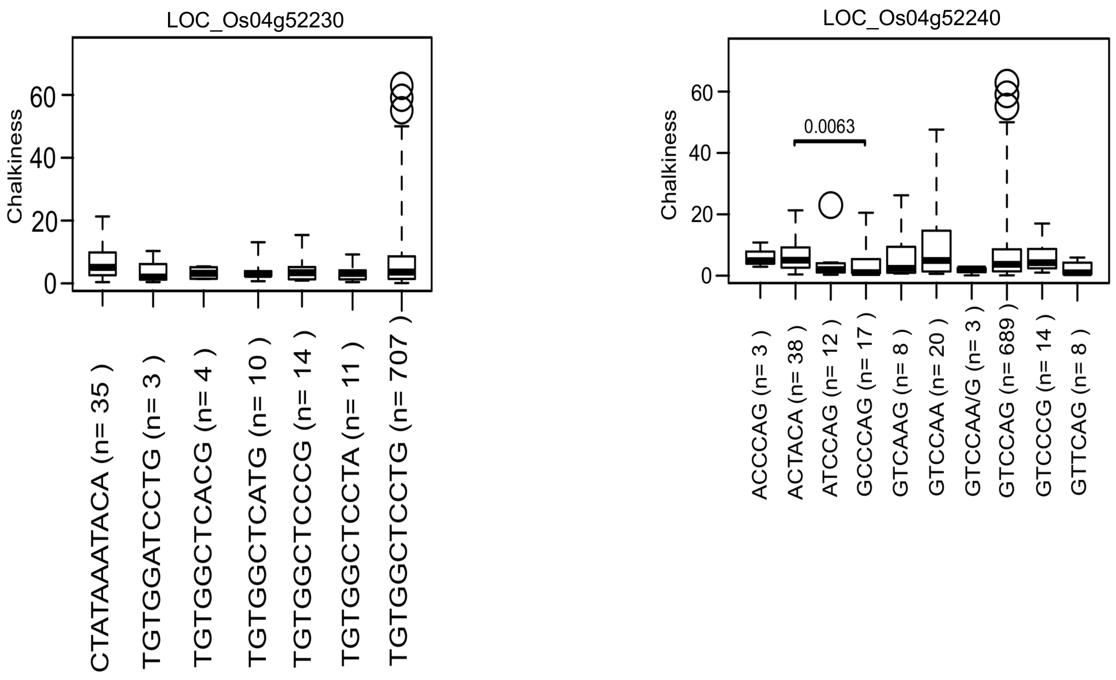

Figure S7: GWAS conducted for PGC in the MAGIC population identified genomic loci on chromosome 4. Subsequently, digging down the chromosome 4 region identified candidate genes LOC_Os04g52230, and LOC_Os04g52240, and validated the role of haplotypes identified earlier in combined germplasm panel for conferring lower chalkiness; within boxplots, the pairwise significance level was tested using the Wilcoxon test.

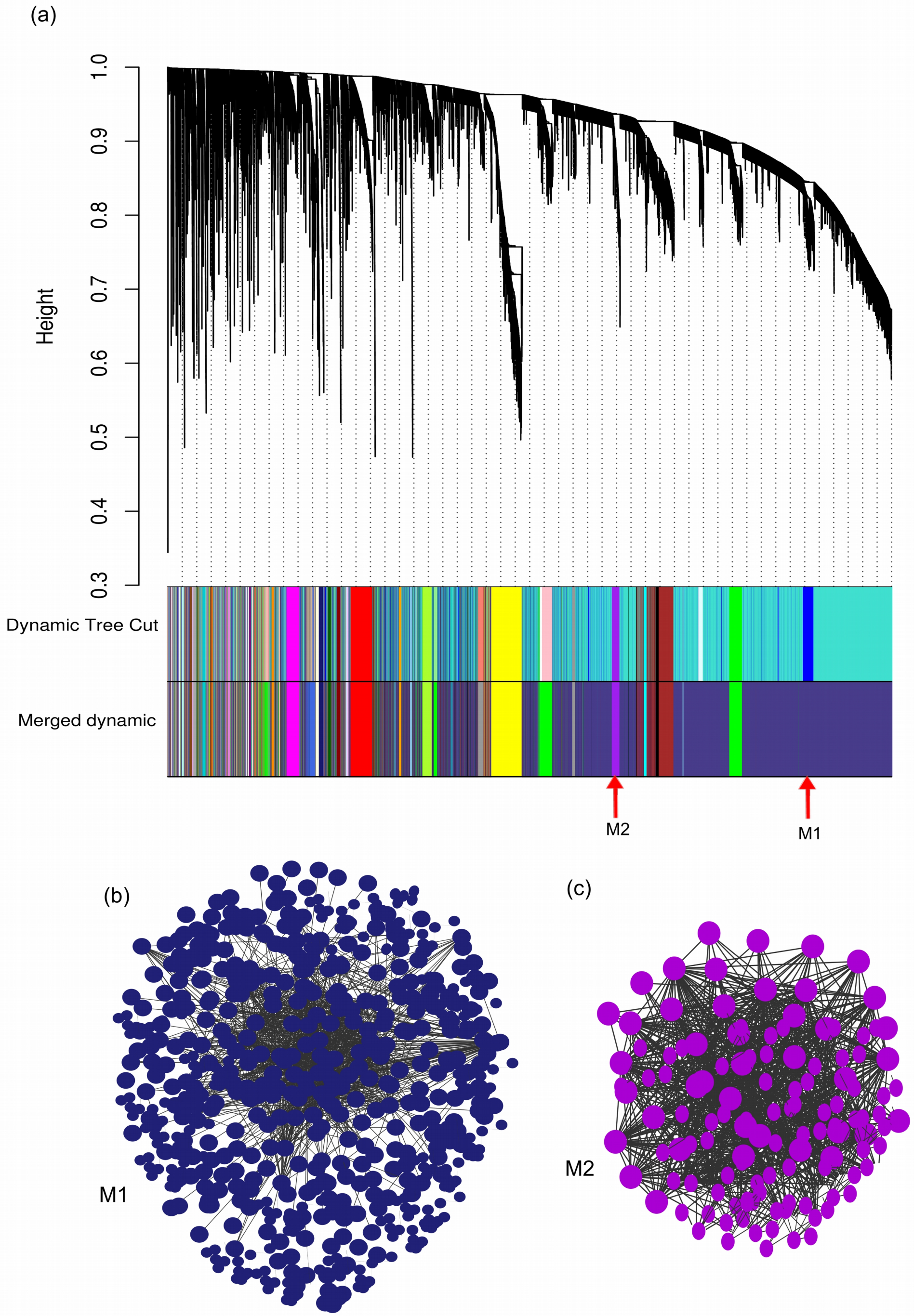

Figure S8: Gene regulatory network created using the transcriptome data generated from contrasting chalk haplotype containing lines. (a) Average linkage hierarchical cluster tree of genes with dissimilarity based on topological overlap. The color band underneath the dendrogram shows the visual module assignments by the dynamic tree cut as merged module colors and original module colors. (b) Visualization of network of modules M1 (darskslate blue) and M2 (purple) (c).

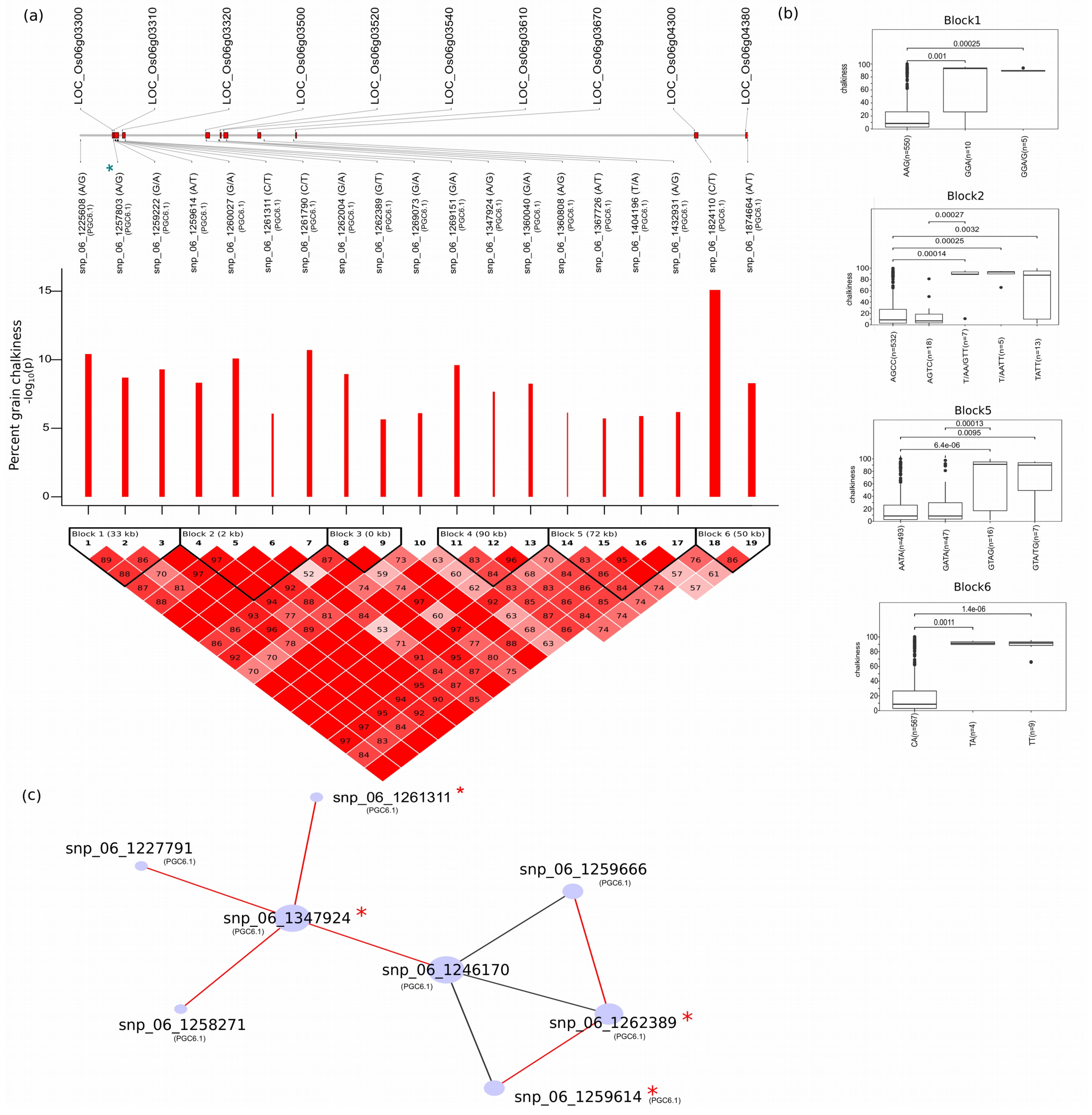

Figure S9: GWAS for PGC revealed the significant association on chromosome 6 genomic regions within combined germplasm panel. (a) Linkage disequilibrium (LD) plot containing 19-tag SNPs along with their respective QTL region (PGC6.1), present within 6 LD-blocks on chromosome 6 marked with the -$\log_{10}$-scaled P-values (-$\log_{10}(P)$), indicate significantly association with grain chalkiness. Notably, all 19-tag SNPs show negative effect on the PGC (red bars) though with varying effect size (signified by bars' width), thus promote lower grain chalkiness. (b) Boxplots show the haplotypes constructed from LD blocks 1, 2, 5 and 6, exhibiting the contrasting phenotypic values for PGC; within boxplots the pairwise significance level was tested using the Wilcoxon test, (c) Mutual interactions between key significant SNPs with corresponding QTL regions, along with SNPs found overlapping between both LD-plot and epistatic interactions are marked with red asterisks.
