# Supplementary material for: Genome‐wide association coupled gene to gene interaction studies unveil novel epistatic targets among major effect loci impacting rice grain chalkiness
Source: Plant Biotechnol J. 2020 Dec 9;19(5):910–25. doi: 10.1111/pbi.13516 (PMC8131057; doi:10.1111/pbi.13516)
Supplement: Supplementary file 2 — Appendix S1 Discovery of chalk genetic regions through GWAS in Japonica panel. [file PBI-19-910-s004.docx]

**Appendix S1**

**Discovery of chalk genetic regions through GWAS in Japonica panel**

A total of 284 *Japonica* diversity accessions covering from 44 countries were phenotyped for PGC, value of which ranges from 0 to 100 (Figure S1). Ge`nome-wide SNPs were called from the average ~15X resequencing data using the Nipponbare (MSU 7 release version) reference genome. A total of 868,941 high-quality SNPs were retained upon filtering from 284 *Japonica* lines to calculate the population structure using principal component analysis (PCA) and subsequently to conduct GWAS. The top two principle components (PCs) explained 29.55% variation where first PC contributed to separate the *tropical* and *temperate japonica* (18.47% of variation), while second PC explains 11.08% genetic variance which separates subset of *tropical japonica* and a *Japx* entries (Figure S2a). The GWAS identified significant loci regulating PGC on chromosomes 2 (*PGC2.1*) and chromosome 6 (*PGC6.1*) passing significant threshold criteria following Bonferroni corrected P-values with −log_10_(*P*)≥ 7.24 (horizontal red line), and false discovery rate (FDR) corrected P-values (P<0.05; green dots), while genetic loci *PGC1.2*, *PGC2.6,* and *PGC4.4* from chromosome 1, 2, and 4 surpassed only the FDR threshold criterion (Figure below). Nonetheless, in *Japonica* subspecies despite underlying high PGC variation only a few of the significant genetic regions were identified (Figure below).

Genome-wide random mutations occur in the diversity lines creates genetic diversity; these genomic variants (SNPs) impacting chalk identified through GWAS by linking genotype-phenotype data. Employing ultra-dense genotypic data form re-sequenced 284 *Japonica* rice accessions utilizing GWAS, identified limited genetic regions, possibly due to lower genetic variance. The finding is consistent with previous studies where overall lower genetic variation was assessed in *japonica* subspecies for key agronomic traits, bearing the extended linkage disequilibrium with narrow genetic variation (McCouch et al., 2016; Misra et al., 2017).

**Methods**

*Single-locus genome-wide association study (GWAS) for percent grain chalkiness (PGC)*. Manhattan plot for PGC utilizing 284 *japonica* accessions showed merely a total of two genomic regions PGC2.1 and PGC6.1, identified surpassing Bonferroni threshold (-log_10_*P* > 7.24; red line), whereas, total 5 genomic loci were found significant following false discovery rate (FDR) threshold criterion (P<0.05) represented by green dots.

**
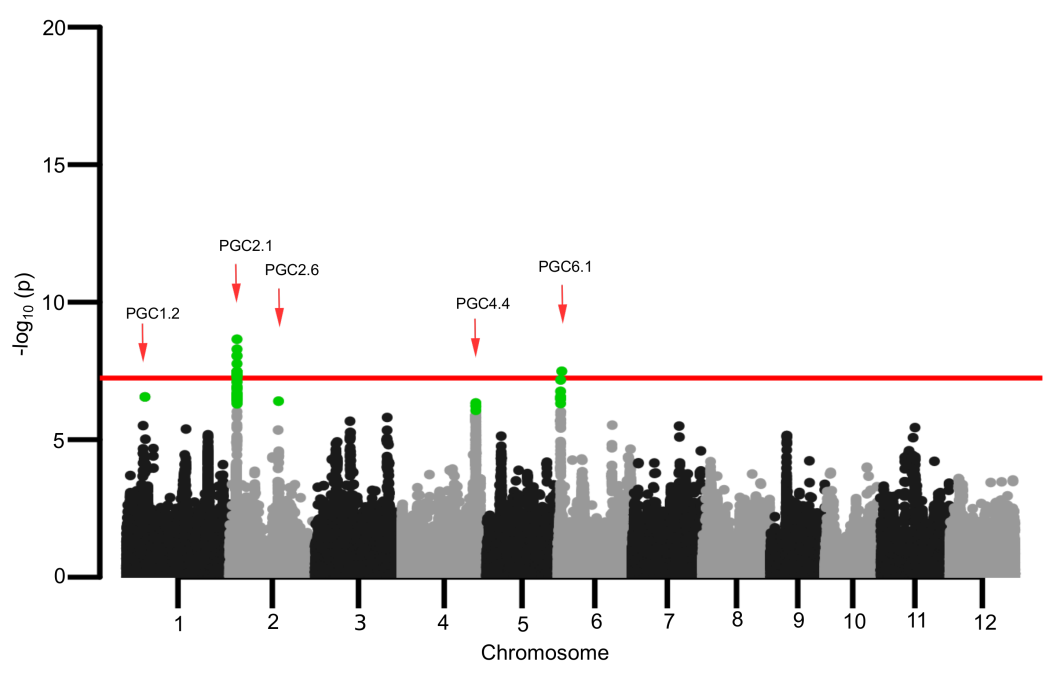
**

**Figure:** Single-locus genome-wide association study (GWAS) for percent grain chalkiness (PGC) using japonica panel (a) Manhattan plot for PGC utilizing 284 japonica accessions showed merely a total of two genomic regions PGC2.1 and PGC6.1, identified surpassing Bonferroni threshold (-log10P > 7.24; red line), whereas, total 5 genomic loci were found significant following false discovery rate (FDR) threshold criterion (P<0.05) represented by green dots.

**References**

McCouch, S.R., Wright, M.H., Tung, C.W., Maron, L.G., McNally, K.L., Fitzgerald, M., Singh, N., DeClerck, G., Agosto-Perez, F., Korniliev, P., Greenberg, A.J., Naredo, M.E., Mercado, S.M., Harrington, S.E., Shi, Y., Branchini, D.A., Kuser-Falcao, P.R., Leung, H., Ebana, K., Yano, M., Eizenga, G., McClung, A. and Mezey, J. (2016) Open access resources for genome-wide association mapping in rice. *Nat. Commun.* **7**, 10532.

Misra, G., Badoni, S., Anacleto, R., Graner, A., Alexandrov, N. and Sreenivasulu, N. (2017) Whole genome sequencing-based association study to unravel genetic architecture of cooked grain width and length traits in rice. *Scientific reports* **7**, 12478.
