# Supplementary material for: Genome‐wide association coupled gene to gene interaction studies unveil novel epistatic targets among major effect loci impacting rice grain chalkiness
Source: Plant Biotechnol J. 2020 Dec 9;19(5):910–25. doi: 10.1111/pbi.13516 (PMC8131057; doi:10.1111/pbi.13516)
Supplement: Supplementary file 3 — Appendix S2 Epistatic interactions that regulate PGC. [file PBI-19-910-s002.docx]

**Appendix S2: Epistatic interactions that regulate PGC**

To determine the potential genetic interactions among the different target genes/loci influencing the PGC, we conducted an epistatic interaction analysis using a linear regression model between the multiple loci in the diversity panel. The results revealed a substantial number of highly significant epistatic interactions between chromosomes 1 (*PGC1.1*, *PGC1.2*) and 4 (*PGC4.4*). The GWAS identified an important QTL *PGC4.4* that had a total of 15-tagged SNPs covering 15 candidate genes, across four different linkage disequilibrium (LD) blocks within the region. The candidate genes significantly affected chalkiness, both positively and negatively. This provided evidence for the bi-directional behavior of the loci present in the *PGC4.4* region (Figure S6, Table S2). The genes of *PGC4.4* belonged to a diverse array of functional categories, including GA biosynthesis, protein targeting, and a few loci of unknown function that were involved in epistatic interactions (Table S3), but the genes associated with cell vesicular transport and lignin biosynthesis, did not show any epistatic interactions. While LD blocks 1 and 2 positively impacted the chalkiness, the tagged SNPs within blocks 3 and 4, contributed to both increased and lower chalkiness within *PGC4.4* (Figure S6). Within LD block 3, the two tagged SNPs (snp_04_30679307 lying on LOC_Os04g51770 and snp_04_31069222 present on LOC_Os04g52270) were in the vicinity of several GA biosynthetic genes, that contributed highly to the increased chalkiness (Figure S6, Table S2). The candidate gene LOC_Os04g52250 (unknown function) that was present in LD block 3, had a negative effect, as it reduced the chalkiness.

The key snp_01_7687212 of *PGC1.2* interacted with the tandemly duplicated GA biosynthesis genes of *PGC4.4.* Moreover, this SNP also interacted with the other SNPs in the genic regions of chromosome 3 (*PGC3.3*), encoding heat shock factor-type proteins (snp_03_6536294 on LOC_Os03g12370), DNA-methyl transferases (snp_03_6660602 on LOC_Os03g12570), and other candidates with unknown functions (Figure S5, Table S2). These results emphasize the importance that a single SNP can have on the genetic interactions of the *PGC3.3, PGC4.4,* and *PGC6.8* loci to regulate the PGC. Epistatic interactions between *PGC4.4* and *PGC5.8* were identified (Figure S5). In addition, *PGC1.2* was found to interact with two different genetic regions*, PGC3.3* and *PGC6.8* (Figure S5). Interestingly, on chromosome 7 (*PGC7.8*) there were four epistatic interactions with different QTL regions*: PGC6.1, PGC6.8*, *PGC3.7*, and *PGC1.9*, which involved both minor and major effect loci influencing the chalk levels (Figure S5). Notably, most of the interacting SNPs from *PGC1.1* and *PGC3.3* showed a positive effect on the trait, conferring a higher degree of PGC, whereas all loci from *PGC1.9*, *PGC3.7*, *PGC6.1,* and *PGC6.8* had negative effects on the trait, conferring lower PGC (Table S2). Each of the QTLs (*PGC1.2*, *PGC4.4*, *PGC5.8*, *PGC6.8,* and *PGC7.8*) had a different set of SNPs contributing to its chalkiness, both positively and negatively (Table S2). We consequently looked carefully at the SNP levels, such as snp_05_5376161 from *PGC5.2* (PVE 19.37) that was located in the intergenic region of LOC_Os05g09520-LOC_Os05g09530 (with the *GW5* locus impacting grain width), and positively contributed to an increase in chalkiness. It also interacted epistatically with another locus, snp_06_30974876 from the QTL *PGC6.8*, which was shown to contribute to reducing the chalkiness (Figure S5, Table S2). Likewise, two SNPs (snp_05_29337175 and snp_05_29341831) on chromosome 5 (*PGC5.*8) contributed to an increase in chalkiness and interacted with a set of SNPs from *PGC6.8* of chromosome 6, to lower the chalk levels (Figure S5, Table S2).
