# Supplementary material for: Genome‐wide association coupled gene to gene interaction studies unveil novel epistatic targets among major effect loci impacting rice grain chalkiness
Source: Plant Biotechnol J. 2020 Dec 9;19(5):910–25. doi: 10.1111/pbi.13516 (PMC8131057; doi:10.1111/pbi.13516)
Supplement: Supplementary file 4 — Table S1 Significant QTLs (showing PVE≤10) identified for regulating PGC along with their physical position on chromosomes, underlying topmost SNP, and further details. Table S2 Details of total significant SNPs identified under chalk‐regulating QTLs with their significance level, effect, and their functional relevance. Table S3 Genome wide epistasis interaction targets (mentioned as region 1 and 2) identified from the GWAS genetic variants associated with grain chalkiness. Table S4 Co‐expressed gene regulatory networks of developing seeds derived from diversity lines containing contrasting chalk haplotypes. The interactions among target‐QTL containing epistasis genes located in Module (M1) and Module (M2). Table S5 Detail of germplasm used for the study with their phenotypic values for grain chalkiness and grain‐size parameters, along with their countries of origin. [file PBI-19-910-s001.pdf]

Table S1: Significant QTLs (showing PVE≤10) identified for regulating PGC along with their physical position on chromosomes, underlying topmost SNP, and further details.

QTL ID	Chr	Start	End	h2	PVE‡	Length (Kb)	Top SNP	Allele†	Beta	P-value	Ref	Alt	Overlapping Gene	SNP effect	Functional annotation
PGC1.1	1	1548861	1548876	0.01	3.9	0.015	snp_01_1548876	J	0.38	3.37E-07	G	A	LOC_Os01g03720	downstream gene variant	MYB transcription factor
PGC1.3	1	14264874	14640409	0.09	9	375.5	snp_01_14640409	J	-0.76	7.06E-08	A	G	LOC_Os01g25820-LOC_Os01g25839	intergenic region	SNP intergenic
PGC1.4	1	15427462	15427462	0.07	7	0.001	snp_01_15427462	J	-1.37	2.50E-07	C	T	LOC_Os01g27660	missense variant (Val->Ile)	Unknown
PGC1.5	1	19370071	19370071	0.08	9.6	0.001	snp_01_19370071	J	-1.02	3.01E-06	C	T	LOC_Os01g35000	upstream gene variant	Unknown
PGC1.6	1	23951914	24415741	0.08	8.5	463.8	snp_01_24415741	J	-0.82	1.08E-07	G	A	LOC_Os01g42909	missense variant (Thr->Ile)	Unknown
PGC1.7	1	30011423	30028390	0.05	5.2	17	snp_01_30012806	J	-0.56	1.18E-06	G	T	LOC_Os01g52180	upstream gene variant	Triacylglycerol lipase
PGC1.8	1	31725691	31725691	0.08	8.5	0.001	snp_01_31725691	J	-0.87	4.72E-07	G	C	LOC_Os01g55140	upstream gene variant	Unknown
PGC1.9	1	34024590	34329540	0.38	9.9	305	snp_01_34094388	J	-0.74	2.23E-08	A	G	LOC_Os01g58980	upstream gene variant	Unknown
PGC2.1	2	3155919	3155919	0.01	3.7	0.001	snp_02_3155919	I	-0.39	3.95E-07	G	A	LOC_Os02g06310	stop gained (Trp->*)	Unknown
PGC2.5	2	18033568	18033568	0.01	2	0.001	snp_02_18033568	J	0.54	2.50E-07	G	A	LOC_Os02g30300-LOC_Os02g30310	intergenic region	SNP intergenic
PGC2.7	2	25253542	25253542	0.01	5.9	0.001	snp_02_25253542	J	0.42	5.39E-07	C	T	LOC_Os02g42020	synonymous variant	Unknown
PGC3.1	3	778685	795874	0.07	7.8	17.2	snp_03_778685	J	-1.07	1.41E-06	G	A	LOC_Os03g02280	upstream gene variant	Unknown
PGC3.2	3	3476464	3476464	0.05	6.9	0.001	snp_03_3476464	J	-0.5	1.87E-06	T	A	LOC_Os03g06860	upstream gene variant	signalling light
PGC3.3	3	6461443	6728014	0.03	6.6	266.6	snp_03_6660602	J	0.77	1.52E-06	A	T	LOC_Os03g12570	synonymous variant	DNA methyltransferases
PGC3.4	3	7012241	7014371	0.01	2.2	2.1	snp_03_7012241	J	0.54	3.74E-07	C	T	LOC_Os03g12980	upstream gene variant	Unknown
PGC3.5	3	8735678	9265469	0.07	9	529.8	snp_03_8735678	J	-0.87	9.72E-08	C	T	LOC_Os03g15830	synonymous variant	DNA unspecified
PGC3.7	3	26840782	26840782	0.02	5.2	0.001	snp_03_26840782	J	-0.35	1.37E-06	A	G	LOC_Os03g47470-LOC_Os03g47480	intergenic region	SNP intergenic
PGC3.8	3	27649256	27649256	0.02	3.8	0.001	snp_03_27649256	J	0.35	2.61E-06	G	T	LOC_Os03g48480	upstream gene variant	Acyl-CoA thioesterase
PGC4.1	4	20000192	20000192	0.07	7.2	0.001	snp_04_20000192	J	-0.59	1.51E-06	T	C	LOC_Os04g33030	downstream gene variant	Unknown
PGC4.2	4	21339618	21430298	0.03	4.7	90.7	snp_04_21430298	J	-0.78	6.00E-07	A	T	LOC_Os04g35250	upstream gene variant	GRAS transcription factor
PGC4.3	4	26749483	26749483	0.08	9.6	0.001	snp_04_26749483	J	-1.02	3.07E-06	G	A	LOC_Os04g45270	upstream gene variant	Aspartate protease
PGC5.1	5	3874628	3893590	0.08	9.4	19	snp_05_3874628	J	-0.96	8.42E-08	C	T	LOC_Os05g07300	downstream gene variant	Postranslational modification
PGC5.4	5	13518841	13518841	0.03	3.9	0.001	snp_05_13518841	J	-0.47	1.68E-06	A	T	LOC_Os05g23570	downstream gene variant	Unknown
PGC5.5	5	17201845	17845842	0.01	3	644	snp_05_17845842	J	0.39	7.16E-07	C	T	LOC_Os05g30780	upstream gene variant	Nitrile lyases

QTL ID	Chr	Start	End	h2	PVE‡	Length (Kb)	Top SNP	Allele†	Beta	P-value	Ref	Alt	Overlapping Gene	SNP effect	Functional annotation
PGC5.6	5	19611671	19643800	0.07	7.8	32.1	snp_05_19635339	J	-1.07	1.41E-06	A	T	LOC_Os05g33410	missense variant (Asn->Ile)	Aspartate protease
PGC5.7	5	21681407	21681407	0.11	9.2	0.001	snp_05_21681407	J	-0.65	1.38E-06	A	G	LOC_Os05g37090-LOC_Os05g37100	intergenic region	SNP intergenic
PGC6.4	6	17782023	17782039	0.07	7.9	0.016	snp_06_17782023	J	-0.94	3.32E-07	G	A	LOC_Os06g30680	downstream gene variant	Unknown
PGC6.5	6	20015162	20406028	0.07	7.9	390.9	snp_06_20015162	J	-0.78	1.10E-06	C	T	LOC_Os06g34400	upstream gene variant	Unknown
PGC7.1	7	121611	121611	0.07	7.1	0.001	snp_07_121611	J	-1.07	1.47E-06	C	T	LOC_Os07g01200	upstream gene variant	Acetyl CoA Carboxylation
PGC7.2	7	1957012	1957012	0.02	3.1	0.001	snp_07_1957012	J	-0.58	4.97E-07	T	C	LOC_Os07g04400	upstream gene variant	Unknown
PGC7.3	7	2680353	2732891	0.04	6.6	52.5	snp_07_2732891	J	0.34	5.76E-07	A	T	LOC_Os07g05710	upstream gene variant	Unknown
PGC7.4	7	4516513	4516513	0.1	5.9	0.001	snp_07_4516513	J	-0.8	1.23E-06	A	C	LOC_Os07g08770	upstream gene variant	Development unspecified
PGC7.5	7	7622206	7622206	0.06	5.3	0.001	snp_07_7622206	J	-0.84	9.86E-07	A	T	LOC_Os07g13270	upstream gene variant	SNF7 transcription factor
PGC7.6	7	17273541	17277936	0.02	2.9	4.4	snp_07_17273541	J	0.48	1.88E-06	C	T	LOC_Os07g29420	missense variant (Val->Met)	RNA transcription
PGC7.7	7	19793873	19910304	0.01	3.9	116.4	snp_07_19793873	J	0.43	4.02E-07	G	A	LOC_Os07g33150	upstream gene variant	Unknown
PGC7.8	7	22404589	22502462	0.37	5.2	97.9	snp_07_22443235	J	-0.39	3.75E-08	G	A	LOC_Os07g37480	missense variant (Pro->Ser)	Unknown
PGC8.1	8	3648813	3648813	0.02	2.8	0.001	snp_08_3648813	J	0.39	2.39E-06	A	G	LOC_Os08g06474-LOC_Os08g06478	intergenic region	SNP intergenic
PGC8.2	8	4173699	4173699	0.03	2.9	0.001	snp_08_4173699	J	0.34	1.76E-07	G	A	LOC_Os08g07430	splice region variant&intron variant	Unknown
PGC8.3	8	6071731	6071731	0.08	7.3	0.001	snp_08_6071731	J	-0.47	2.10E-07	C	T	LOC_Os08g10360	missense variant (Pro->Ser)	Unknown
PGC8.4	8	24527577	24527577	0.03	4.1	0.001	snp_08_24527577	J	0.41	1.31E-06	C	T	LOC_Os08g38790	upstream gene variant	Unknown
PGC8.5	8	26380470	26380470	0.04	6.2	0.001	snp_08_26380470	J	-0.91	2.83E-06	G	A	LOC_Os08g41780	missense variant (Arg->Cys)	Triacylglycerol lipase
PGC8.6	8	27238987	27238987	0.04	6.6	0.001	snp_08_27238987	J	-0.99	7.30E-07	G	C	LOC_Os08g43090	intron variant	bZIP transcription factor
PGC8.7	8	28221949	28260958	0.06	7.8	39	snp_08_28221949	J	-0.64	1.66E-07	C	T	LOC_Os08g44950	upstream gene variant	Unknown
PGC9.2	9	6549110	6905233	0.02	7.1	356.1	snp_09_6900752	J	-0.56	1.15E-07	A	G	LOC_Os09g12200	downstream gene variant	Unknown
PGC9.3	9	17170796	17170796	0.09	9.6	0.001	snp_09_17170796	J	-0.84	2.59E-06	C	T	LOC_Os09g28290	upstream gene variant	Unknown
PGC9.4	9	19571667	19571667	0.02	7.2	0.001	snp_09_19571667	J	0.45	1.81E-06	A	G	LOC_Os09g32820	synonymous variant	Phospho ribosyl transferases
PGC10.1	10	447233	447233	0.01	2.7	0.001	snp_10_447233	J	0.73	1.24E-06	G	A	LOC_Os10g01670	upstream gene variant	Unknown
PGC11.1	11	6246057	6246057	0.07	5.7	0.001	snp_11_6246057	J	-0.89	1.33E-06	C	T	LOC_Os11g11280	upstream gene variant	Unknown

QTL ID	Chr	Start	End	h2	PVE‡	Length (Kb)	Top SNP	Allele†	Beta	P-value	Ref	Alt	Overlapping Gene	SNP effect	Functional annotation
PGC11.2	11	10259061	10259061	0.05	5.5	0.001	snp_11_10259061	J	-0.58	1.19E-06	G	A	LOC_Os11g18194	intron variant	Brassinosteroid synthesis-degradation
PGC11.3	11	11126781	11198781	0.25	3.7	72	snp_11_11197785	J	-0.7	1.25E-07	C	T	LOC_Os11g19450	upstream gene variant	Unknown
PGC11.4	11	14301203	15443889	0.04	6	1143	snp_11_14374189	J	-0.76	5.91E-08	C	T	LOC_Os11g25230	downstream gene variant	Nitrile lyases
PGC11.5	11	28872156	28924197	0.05	7.4	52	snp_11_28889703	J	-1.02	1.11E-07	T	G	LOC_Os11g47900	upstream gene variant	GRAS transcription factor
PGC12.1	12	2419472	2419472	0.08	9.5	0.001	snp_12_2419472	J	-1.16	1.18E-10	G	A	LOC_Os12g05360	downstream gene variant	Unknown
PGC12.2	12	3384075	3588777	0.04	6.1	204.7	snp_12_3384075	J	-0.89	3.00E-07	C	T	LOC_Os12g06920	downstream gene variant	Stress biotic
PGC12.3	12	10819584	10819586	0.17	5.8	0.002	snp_12_10819586	J	-0.68	1.05E-06	T	C	LOC_Os12g18729	upstream gene variant	Unknown
PGC12.4	12	21592589	21889863	0.3	9.7	297.3	snp_12_21592589	J	-0.5	3.46E-07	C	T	LOC_Os12g35510	downstream gene variant	Unknown

†represent the source subspecies showing the major occurrence of alternate allele, where J and I indicate *Japonica* , and *Indica* subspecies, respectively.

‡percent of phenotypic variation explained by the respective QTL

Table S2: Details of total significant SNPs identified under chalk regulating QTLs with their significance level, effect, and their functional relevance.

QTL ID	SNP ID	Allele†	Beta	P-value	Chr	Ref	Alt	Region	Gene	Amino acid change	GoMAPMAN
PGC1.1	snp_01_1548861	J	0.36	2.94E-06	1	T	C	downstream_gene_variant	LOC_Os01g03720	-	'27.3.25'_'RNA.regulation_of_transcription.MYB_domai
PGC1.1	snp_01_1548874	J	0.38	6.57E-07	1	A	G	downstream_gene_variant	LOC_Os01g03720	-	'27.3.25'_'RNA.regulation_of_transcription.MYB_domain_transcription_factor_family'
PGC1.1	snp_01_1548876	J	0.38	3.37E-07	1	G	A	downstream_gene_variant	LOC_Os01g03720	-	'27.3.25'_'RNA.regulation_of_transcription.MYB_domain_transcription_factor_family'
PGC1.2	snp_01_6520765	J	-0.92	2.68E-08	1	G	C	5_prime_UTR_variant	LOC_Os01g11960	-	'27.3.69'_'RNA.regulation_of_transcription.SET-domain_transcriptional_regulator_family'
PGC1.2	snp_01_6896083	J	-0.49	1.54E-06	1	T	A	3_prime_UTR_variant	LOC_Os01g12530	-	28.1'_'DNA.synthesis/Chromatin_structure'
PGC1.2	snp_01_7002807	J	-0.55	2.29E-07	1	G	A	intergenic_region	LOC_Os01g12680-LOC_Os01g12690	-	NA
PGC1.2	snp_01_7687212	J	0.73	2.05E-07	1	C	T	3_prime_UTR_variant	LOC_Os01g13740	-	'27.3.20'_'RNA.regulation_of_transcription.G2-like_transcription_factor_family,_GARP'
PGC1.2	snp_01_7754524	J	-0.83	9.18E-09	1	C	T	upstream_gene_variant	LOC_Os01g13820	-	'35.2'_'not_assigned.unknown'
PGC1.2	snp_01_7765181	J	-0.83	9.46E-09	1	C	T	upstream_gene_variant	LOC_Os01g13850	-	'35.2'_'not_assigned.unknown'
PGC1.2	snp_01_7777612	J	-0.55	1.50E-08	1	T	A	upstream_gene_variant	LOC_Os01g13880	-	'35.2'_'not_assigned.unknown'
PGC1.2	snp_01_7792418	J	-0.83	9.43E-09	1	T	C	upstream_gene_variant	LOC_Os01g13900	-	'35.2'_'not_assigned.unknown'
PGC1.2	snp_01_7794702	J	-0.83	9.46E-09	1	A	G	upstream_gene_variant	LOC_Os01g13900	-	'35.2'_'not_assigned.unknown'
PGC1.2	snp_01_7801197	J	-0.82	9.85E-09	1	T	C	upstream_gene_variant	LOC_Os01g13920	-	35.2'_'not_assigned.unknown'
PGC1.2	snp_01_7865741	J	-0.71	1.35E-07	1	G	T	intron_variant	LOC_Os01g14050	-	'30.3'_'signalling.calcium'
PGC1.2	snp_01_7901620	J	-0.71	1.38E-07	1	T	C	upstream_gene_variant	LOC_Os01g14100	-	'34.99'_'transport.misc'
PGC1.2	snp_01_7965859	J	0.36	2.32E-07	1	G	A	upstream_gene_variant	LOC_Os01g14210	-	'35.2'_'not_assigned.unknown'
PGC1.2	snp_01_8010900	J	-0.71	1.33E-07	1	C	T	upstream_gene_variant	LOC_Os01g14300	-	'35.2'_'not_assigned.unknown'
PGC1.2	snp_01_8022213	J	-0.68	1.13E-07	1	A	G	upstream_gene_variant	LOC_Os01g14310	-	'35.2'_'not_assigned.unknown'
PGC1.2	snp_01_8055815	J	-0.71	1.37E-07	1	C	T	upstream_gene_variant	LOC_Os01g14370	-	'27.3.54'_'RNA.regulation_of_transcription.Histone_acetyltransferases'
PGC1.2	snp_01_8059695	J	-0.65	1.35E-06	1	G	T	synonymous_variant	LOC_Os01g14390	p.Val304Val	'35.2'_'not_assigned.unknown'
PGC1.3	snp_01_14264874	J	0.62	2.17E-06	1	C	T	missense_variant	LOC_Os01g25270	p.Ala154Val	'35.1.5'_'not_assigned.no_ontology.pentatricopeptide_(PPR)_repeat-containing_protein'
PGC1.3	snp_01_14326807	J	0.63	1.85E-06	1	C	T	synonymous_variant	LOC_Os01g25360	p.Ser182Ser	'26.8'_'misc.nitrilases,_*nitrile_lyases,_berberine_bridge_enzymes,_reticuline_oxidases,_troponine_reductases'
PGC1.3	snp_01_14640409	J	-0.76	7.06E-08	1	A	G	intergenic_region	LOC_Os01g25820-LOC_Os01g25839	-	NA
PGC1.4	snp_01_15427462	J	-1.38	2.50E-07	1	C	T	missense_variant	LOC_Os01g27660	p.Val665Ile	'35.2'_'not_assigned.unknown'
PGC1.5	snp_01_19370071	J	-1.02	3.01E-06	1	C	T	upstream_gene_variant	LOC_Os01g35000	-	'35.2'_'not_assigned.unknown'
PGC1.6	snp_01_23951914	J	0.48	2.22E-06	1	G	A	upstream_gene_variant	LOC_Os01g42260	-	'33.99'_'development.unspecified'
PGC1.6	snp_01_24415530	J	-0.82	1.15E-06	1	C	T	downstream_gene_variant	LOC_Os01g42909	-	'35.2'_'not_assigned.unknown'
PGC1.6	snp_01_24415613	J	-0.83	1.10E-07	1	G	A	downstream_gene_variant	LOC_Os01g42909	-	'35.2'_'not_assigned.unknown'
PGC1.6	snp_01_24415741	J	-0.83	1.08E-07	1	G	A	missense_variant	LOC_Os01g42909	p.Thr53Ile	'35.2'_'not_assigned.unknown'
PGC1.7	snp_01_30011423	J	-0.56	1.20E-06	1	T	A	upstream_gene_variant	LOC_Os01g52180	-	'11.9.2.1'_'lipid_metabolism.lipid_degradation.lipases.triacylglycerol_lipase'

QTL ID	SNP ID	Allele†	Beta	P-value	Chr	Ref	Alt	Region	Gene	Amino acid change	GoMAPMAN
PGC1.7	snp_01_30012806	J	-0.56	1.18E-06	1	G	T	upstream_gene_variant	LOC_Os01g52180	-	'11.9.2.1'_'lipid_metabolism.lipid_degradation.lipases.triacylglycerol_lipase'
PGC1.7	snp_01_30028390	J	-0.56	1.20E-06	1	G	T	upstream_gene_variant	LOC_Os01g52214	-	'9.1.2'_'mitochondrial_electron_transport_/_ATP_synthesis.NADH-DH.localisation_not_clear'
PGC1.8	snp_01_31725691	J	-0.87	4.73E-07	1	G	C	upstream_gene_variant	LOC_Os01g55140	-	'35.2'_'not_assigned.unknown'
PGC1.9	snp_01_34024590	J	-0.70	7.05E-07	1	G	A	downstream_gene_variant	LOC_Os01g58880	-	'35.2'_'not_assigned.unknown'
PGC1.9	snp_01_34025169	J	-0.81	7.78E-08	1	G	A	downstream_gene_variant	LOC_Os01g58880	-	'35.2'_'not_assigned.unknown'
PGC1.9	snp_01_34026394	J	-0.81	7.85E-08	1	G	A	downstream_gene_variant	LOC_Os01g58880	-	'35.2'_'not_assigned.unknown'
PGC1.9	snp_01_34026451	J	-0.81	7.60E-08	1	G	A	downstream_gene_variant	LOC_Os01g58880	-	'35.2'_'not_assigned.unknown'
PGC1.9	snp_01_34027468	J	-0.67	1.28E-06	1	G	A	intergenic_region	LOC_Os01g58880-LOC_Os01g58890	-	NA
PGC1.9	snp_01_34046764	J	-0.70	4.67E-07	1	G	A	upstream_gene_variant	LOC_Os01g58910	-	'33.99'_'development.unspecified'
PGC1.9	snp_01_34073646	J	-0.67	1.24E-06	1	G	A	upstream_gene_variant	LOC_Os01g58950	-	'26.10'_'misc.cytoome_P450'
PGC1.9	snp_01_34088908	J	-0.75	1.65E-07	1	C	T	upstream_gene_variant	LOC_Os01g58970	-	'26.10'_'misc.cytoome_P450'
PGC1.9	snp_01_34090967	J	-0.61	1.22E-06	1	G	C	synonymous_variant	LOC_Os01g58970	p.Val488Val	'26.10'_'misc.cytoome_P450'
PGC1.9	snp_01_34091282	J	-0.61	2.60E-06	1	T	C	3_prime_UTR_variant	LOC_Os01g58970	-	'26.10'_'misc.cytoome_P450'
PGC1.9	snp_01_34091330	J	-1.22	1.22E-06	1	G	A	3_prime_UTR_variant	LOC_Os01g58970	-	'26.10'_'misc.cytoome_P450'
PGC1.9	snp_01_34091573	J	-0.61	1.22E-06	1	C	T	upstream_gene_variant	LOC_Os01g58990	-	'26.10'_'misc.cytoome_P450'
PGC1.9	snp_01_34091603	J	-0.61	1.25E-06	1	C	T	upstream_gene_variant	LOC_Os01g58990	-	'26.10'_'misc.cytoome_P450'
PGC1.9	snp_01_34091689	J	-0.61	1.11E-06	1	C	T	upstream_gene_variant	LOC_Os01g58990	-	'26.10'_'misc.cytoome_P450'
PGC1.9	snp_01_34093214	J	-0.61	1.22E-06	1	T	C	upstream_gene_variant	LOC_Os01g58990	-	'26.10'_'misc.cytoome_P450'
PGC1.9	snp_01_34093308	J	-0.61	1.40E-06	1	G	A	upstream_gene_variant	LOC_Os01g58990	-	'26.10'_'misc.cytoome_P450'
PGC1.9	snp_01_34093361	J	-0.61	1.17E-06	1	C	T	upstream_gene_variant	LOC_Os01g58990	-	'26.10'_'misc.cytoome_P450'
PGC1.9	snp_01_34093477	J	-0.63	1.19E-06	1	C	T	upstream_gene_variant	LOC_Os01g58990	-	'26.10'_'misc.cytoome_P450'
PGC1.9	snp_01_34093486	J	-0.63	1.19E-06	1	C	T	upstream_gene_variant	LOC_Os01g58990	-	'26.10'_'misc.cytoome_P450'
PGC1.9	snp_01_34093489	J	-0.63	1.19E-06	1	G	A	upstream_gene_variant	LOC_Os01g58990	-	'26.10'_'misc.cytoome_P450'
PGC1.9	snp_01_34093506	J	-0.61	1.22E-06	1	G	A	upstream_gene_variant	LOC_Os01g58990	-	'26.10'_'misc.cytoome_P450'
PGC1.9	snp_01_34093651	J	-0.61	1.26E-06	1	C	T	upstream_gene_variant	LOC_Os01g58990	-	'26.10'_'misc.cytoome_P450'
PGC1.9	snp_01_34093658	J	-0.61	1.26E-06	1	C	T	upstream_gene_variant	LOC_Os01g58990	-	'26.10'_'misc.cytoome_P450'
PGC1.9	snp_01_34093869	J	-0.61	1.15E-06	1	C	T	upstream_gene_variant	LOC_Os01g58990	-	'26.10'_'misc.cytoome_P450'
PGC1.9	snp_01_34093972	J	-0.61	1.17E-06	1	C	T	synonymous_variant	LOC_Os01g58980	p.Leu59Leu	'35.2'_'not_assigned.unknown'
PGC1.9	snp_01_34094030	J	-0.61	1.24E-06	1	G	A	missense_variant	LOC_Os01g58980	p.Ala40Val	'35.2'_'not_assigned.unknown'
PGC1.9	snp_01_34094042	J	-0.61	1.26E-06	1	C	T	missense_variant	LOC_Os01g58980	p.Gly36Glu	'35.2'_'not_assigned.unknown'
PGC1.9	snp_01_34094388	J	-0.75	2.23E-08	1	A	G	upstream_gene_variant	LOC_Os01g58980	-	'35.2'_'not_assigned.unknown'
PGC1.9	snp_01_34095211	J	-0.61	1.10E-06	1	G	A	synonymous_variant	LOC_Os01g58990	p.Pro201Pro	'26.10'_'misc.cytoome_P450'
PGC1.9	snp_01_34096098	J	-0.61	1.22E-06	1	T	G	missense_variant	LOC_Os01g58990	p.Leu497Arg	'26.10'_'misc.cytoome_P450'
PGC1.9	snp_01_34096532	J	-0.61	1.22E-06	1	A	G	upstream_gene_variant	LOC_Os01g58980	-	'35.2'_'not_assigned.unknown'
PGC1.9	snp_01_34096916	J	-0.61	1.22E-06	1	T	C	upstream_gene_variant	LOC_Os01g58980	-	'35.2'_'not_assigned.unknown'
PGC1.9	snp_01_34096952	J	-0.68	1.52E-07	1	G	A	upstream_gene_variant	LOC_Os01g58980	-	'35.2'_'not_assigned.unknown'
PGC1.9	snp_01_34097188	J	-0.61	1.22E-06	1	C	T	upstream_gene_variant	LOC_Os01g58980	-	'35.2'_'not_assigned.unknown'
PGC1.9	snp_01_34097538	J	-0.61	1.21E-06	1	C	T	upstream_gene_variant	LOC_Os01g58980	-	'35.2'_'not_assigned.unknown'
PGC1.9	snp_01_34097564	J	-0.61	1.22E-06	1	T	C	upstream_gene_variant	LOC_Os01g58980	-	'35.2'_'not_assigned.unknown'
PGC1.9	snp_01_34097643	J	-0.61	1.22E-06	1	C	T	upstream_gene_variant	LOC_Os01g58980	-	'35.2'_'not_assigned.unknown'

QTL ID	SNP ID	Allele†	Beta	P-value	Chr	Ref	Alt	Region	Gene	Amino acid change	GoMAPMAN
PGC1.9	snp_01_34097928	J	-0.61	1.34E-06	1	G	A	upstream_gene_variant	LOC_Os01g58980	-	'35.2'_'not_assigned.unknown'
PGC1.9	snp_01_34097970	J	-0.61	1.19E-06	1	G	A	upstream_gene_variant	LOC_Os01g58980	-	'35.2'_'not_assigned.unknown'
PGC1.9	snp_01_34099265	J	-0.61	1.26E-06	1	G	A	upstream_gene_variant	LOC_Os01g59000	-	'26.10'_'misc.cytoome_P450'
PGC1.9	snp_01_34099326	J	-0.61	1.18E-06	1	C	G	upstream_gene_variant	LOC_Os01g59000	-	'26.10'_'misc.cytoome_P450'
PGC1.9	snp_01_34100743	J	-0.61	1.25E-06	1	G	A	missense_variant	LOC_Os01g59000	p.Gly434Ser	'26.10'_'misc.cytoome_P450'
PGC1.9	snp_01_34101110	J	-0.61	1.26E-06	1	G	C	3_prime_UTR_variant	LOC_Os01g59000	-	'26.10'_'misc.cytoome_P450'
PGC1.9	snp_01_34101744	J	-0.61	1.22E-06	1	C	T	downstream_gene_variant	LOC_Os01g59000	-	'26.10'_'misc.cytoome_P450'
PGC1.9	snp_01_34101846	J	-0.61	1.22E-06	1	A	G	downstream_gene_variant	LOC_Os01g59000	-	'26.10'_'misc.cytoome_P450'
PGC1.9	snp_01_34101894	J	-0.63	1.13E-06	1	A	C	downstream_gene_variant	LOC_Os01g59000	-	'26.10'_'misc.cytoome_P450'
PGC1.9	snp_01_34102220	J	-0.61	1.21E-06	1	G	C	downstream_gene_variant	LOC_Os01g59000	-	'26.10'_'misc.cytoome_P450'
PGC1.9	snp_01_34102243	J	-0.61	1.22E-06	1	T	C	downstream_gene_variant	LOC_Os01g59000	-	'26.10'_'misc.cytoome_P450'
PGC1.9	snp_01_34102343	J	-0.61	1.14E-06	1	G	A	downstream_gene_variant	LOC_Os01g59000	-	'26.10'_'misc.cytoome_P450'
PGC1.9	snp_01_34102485	J	-0.61	1.22E-06	1	A	G	downstream_gene_variant	LOC_Os01g59000	-	'26.10'_'misc.cytoome_P450'
PGC1.9	snp_01_34105324	J	-0.61	1.12E-06	1	G	T	downstream_gene_variant	LOC_Os01g59000	-	'26.10'_'misc.cytoome_P450'
PGC1.9	snp_01_34106207	J	-0.61	1.19E-06	1	T	C	intergenic_region	LOC_Os01g59000-LOC_Os01g59009	-	NA
PGC1.9	snp_01_34106212	J	-0.61	1.19E-06	1	G	A	intergenic_region	LOC_Os01g59000-LOC_Os01g59009	-	NA
PGC1.9	snp_01_34107076	J	-1.22	1.20E-06	1	A	G	intergenic_region	LOC_Os01g59000-LOC_Os01g59009	-	NA
PGC1.9	snp_01_34107627	J	-0.61	1.22E-06	1	C	T	intergenic_region	LOC_Os01g59000-LOC_Os01g59009	-	NA
PGC1.9	snp_01_34107647	J	-0.61	1.22E-06	1	G	A	intergenic_region	LOC_Os01g59000-LOC_Os01g59009	-	NA
PGC1.9	snp_01_34108181	J	-0.61	1.22E-06	1	C	T	upstream_gene_variant	LOC_Os01g59009	-	'35.2'_'not_assigned.unknown'
PGC1.9	snp_01_34108217	J	-0.61	1.22E-06	1	A	G	upstream_gene_variant	LOC_Os01g59009	-	'35.2'_'not_assigned.unknown'
PGC1.9	snp_01_34108413	J	-0.61	1.22E-06	1	T	C	upstream_gene_variant	LOC_Os01g59009	-	'35.2'_'not_assigned.unknown'
PGC1.9	snp_01_34108798	J	-0.61	1.26E-06	1	C	T	upstream_gene_variant	LOC_Os01g59009	-	'35.2'_'not_assigned.unknown'
PGC1.9	snp_01_34109034	J	-0.68	1.54E-07	1	A	T	upstream_gene_variant	LOC_Os01g59009	-	'35.2'_'not_assigned.unknown'
PGC1.9	snp_01_34109276	J	-0.61	1.20E-06	1	G	A	upstream_gene_variant	LOC_Os01g59009	-	'35.2'_'not_assigned.unknown'
PGC1.9	snp_01_34109750	J	-1.12	5.24E-07	1	A	T	upstream_gene_variant	LOC_Os01g59009	-	'35.2'_'not_assigned.unknown'
PGC1.9	snp_01_34109965	J	-1.22	1.22E-06	1	C	T	upstream_gene_variant	LOC_Os01g59009	-	'35.2'_'not_assigned.unknown'
PGC1.9	snp_01_34110220	J	-1.22	1.21E-06	1	T	A	upstream_gene_variant	LOC_Os01g59009	-	'35.2'_'not_assigned.unknown'
PGC1.9	snp_01_34110893	J	-0.85	1.07E-06	1	T	A	upstream_gene_variant	LOC_Os01g59009	-	'35.2'_'not_assigned.unknown'
PGC1.9	snp_01_34111028	J	-0.61	1.22E-06	1	G	A	upstream_gene_variant	LOC_Os01g59009	-	'35.2'_'not_assigned.unknown'
PGC1.9	snp_01_34111044	J	-0.61	1.18E-06	1	G	A	upstream_gene_variant	LOC_Os01g59009	-	'35.2'_'not_assigned.unknown'
PGC1.9	snp_01_34111087	J	-0.61	1.26E-06	1	T	C	upstream_gene_variant	LOC_Os01g59009	-	'35.2'_'not_assigned.unknown'
PGC1.9	snp_01_34111112	J	-0.61	1.24E-06	1	T	A	upstream_gene_variant	LOC_Os01g59009	-	'35.2'_'not_assigned.unknown'
PGC1.9	snp_01_34111225	J	-0.61	1.18E-06	1	G	A	upstream_gene_variant	LOC_Os01g59009	-	'35.2'_'not_assigned.unknown'
PGC1.9	snp_01_34111273	J	-0.61	1.22E-06	1	A	T	upstream_gene_variant	LOC_Os01g59009	-	'35.2'_'not_assigned.unknown'
PGC1.9	snp_01_34111274	J	-0.61	1.23E-06	1	G	A	upstream_gene_variant	LOC_Os01g59009	-	'35.2'_'not_assigned.unknown'
PGC1.9	snp_01_34111290	J	-0.61	1.22E-06	1	A	G	upstream_gene_variant	LOC_Os01g59009	-	'35.2'_'not_assigned.unknown'
PGC1.9	snp_01_34113132	J	-0.61	1.26E-06	1	C	T	stop_gained	LOC_Os01g59009	p.Arg42*	'35.2'_'not_assigned.unknown'

QTL ID	SNP ID	Allele†	Beta	P-value	Chr	Ref	Alt	Region	Gene	Amino acid change	GoMAPMAN
PGC1.9	snp_01_34113207	J	-0.61	1.18E-06	1	G	A	missense_variant	LOC_Os01g59009	p.Val67Ile	'35.2'_'not_assigned.unknown'
PGC1.9	snp_01_34113208	J	-0.60	1.28E-06	1	T	C	missense_variant	LOC_Os01g59009	p.Val67Ala	'35.2'_'not_assigned.unknown'
PGC1.9	snp_01_34113251	J	-0.61	1.26E-06	1	G	A	synonymous_variant	LOC_Os01g59009	p.Thr81Thr	'35.2'_'not_assigned.unknown'
PGC1.9	snp_01_34113291	J	-0.61	1.18E-06	1	C	T	missense_variant	LOC_Os01g59009	p.His95Tyr	'35.2'_'not_assigned.unknown'
PGC1.9	snp_01_34113515	J	-0.61	1.25E-06	1	T	C	upstream_gene_variant	LOC_Os01g59020	-	'26.10'_'misc.cytoome_P450'
PGC1.9	snp_01_34113641	J	-0.61	1.14E-06	1	C	T	upstream_gene_variant	LOC_Os01g59020	-	'26.10'_'misc.cytoome_P450'
PGC1.9	snp_01_34114447	J	-0.61	1.22E-06	1	C	T	upstream_gene_variant	LOC_Os01g59020	-	'26.10'_'misc.cytoome_P450'
PGC1.9	snp_01_34115426	J	-0.61	1.21E-06	1	G	A	missense_variant	LOC_Os01g59020	p.Val109Met	'26.10'_'misc.cytoome_P450'
PGC1.9	snp_01_34116173	J	-0.61	1.22E-06	1	G	A	missense_variant	LOC_Os01g59020	p.Asp358Asn	'26.10'_'misc.cytoome_P450'
PGC1.9	snp_01_34117353	J	-0.61	1.16E-06	1	C	T	upstream_gene_variant	LOC_Os01g59030	-	'35.2'_'not_assigned.unknown'
PGC1.9	snp_01_34127740	J	-0.63	1.01E-06	1	G	A	upstream_gene_variant	LOC_Os01g59040	-	'35.2'_'not_assigned.unknown'
PGC1.9	snp_01_34129821	J	-0.61	1.28E-06	1	C	A	upstream_gene_variant	LOC_Os01g59040	-	'35.2'_'not_assigned.unknown'
PGC1.9	snp_01_34135362	J	-0.61	1.24E-06	1	C	A	splice_region_variant&intron	LOC_Os01g59060	-	'35.2'_'not_assigned.unknown'
PGC1.9	snp_01_34135914	J	-0.61	1.22E-06	1	C	T	missense_variant	LOC_Os01g59070	p.Ser16Phe	'35.2'_'not_assigned.unknown'
PGC1.9	snp_01_34138372	J	-0.61	1.18E-06	1	A	G	3_prime_UTR_variant	LOC_Os01g59080	-	'35.2'_'not_assigned.unknown'
PGC1.9	snp_01_34147123	J	-0.56	1.66E-06	1	C	T	downstream_gene_variant	LOC_Os01g59100	-	'26.2'_'misc.UDP_glucosyl_and_glucoronyl_transferases'
PGC1.9	snp_01_34329540	J	-0.46	1.16E-06	1	G	A	downstream_gene_variant	LOC_Os01g59360	-	'30.3'_'signalling.calcium'
PGC1.10	snp_01_39443793	J	0.42	1.29E-06	1	G	A	upstream_gene_variant	LOC_Os01g67870	-	'11.8.1.3'_'lipid_metabolism.''exotics''_(steroids,_squalene_etc).sphingolipids.ceramide_glucosyltransferase'
PGC1.10	snp_01_40366212	J	0.38	1.47E-06	1	T	C	downstream_gene_variant	LOC_Os01g69850	-	'33.99'_'development.unspecified'
PGC1.10	snp_01_40488152	J	0.46	5.03E-07	1	G	A	synonymous_variant	LOC_Os01g69990	p.Cys295Cys	'35.1'_'not_assigned.no_ontology'
PGC1.10	snp_01_40488340	J	0.43	2.29E-06	1	G	C	synonymous_variant	LOC_Os01g69990	p.Gly232Gly	'35.1'_'not_assigned.no_ontology'
PGC1.10	snp_01_40495067	J	0.43	2.12E-06	1	C	A	downstream_gene_variant	LOC_Os01g70010	-	'29.2.1.2.2.57'_'protein.synthesis.ribosomal_protein.eukaryotic.60S_subunit.L7A'
PGC1.10	snp_01_40558541	J	0.44	1.30E-06	1	C	G	synonymous_variant	LOC_Os01g70080	p.Phe173Phe	'20.1'_'stress.biotic'
PGC1.10	snp_01_40588603	J	0.40	1.05E-06	1	A	G	downstream_gene_variant	LOC_Os01g70120	-	'35.2'_'not_assigned.unknown'
PGC1.10	snp_01_40600963	J	0.38	2.76E-06	1	T	C	upstream_gene_variant	LOC_Os01g70140	-	'29.5.11'_'protein.degradation.ubiquitin'
PGC1.10	snp_01_40601690	J	0.38	2.56E-06	1	C	T	upstream_gene_variant	LOC_Os01g70140	-	'29.5.11'_'protein.degradation.ubiquitin'
PGC1.10	snp_01_40602040	J	0.38	2.28E-06	1	A	T	upstream_gene_variant	LOC_Os01g70140	-	'29.5.11'_'protein.degradation.ubiquitin'
PGC1.10	snp_01_40609378	J	0.34	2.02E-06	1	A	G	synonymous_variant	LOC_Os01g70160	p.Gly58Gly	'29.5.11.4.2'_'protein.degradation.ubiquitin.E3.RING'
PGC1.10	snp_01_40645958	J	0.28	1.55E-06	1	C	T	intron_variant	LOC_Os01g70210	-	'29.3.4.99'_'protein.targeting.secretory_pathway.unspecified'
PGC2.1	snp_02_3155919	I	-0.39	3.96E-07	2	G	A	stop_gained	LOC_Os02g06310	p.Trp353*	'35.2'_'not_assigned.unknown'
PGC2.2	snp_02_5081459	J	-0.76	2.17E-06	2	C	T	upstream_gene_variant	LOC_Os02g09850	-	'35.2'_'not_assigned.unknown'
PGC2.2	snp_02_5108338	J	-0.86	1.11E-07	2	C	A	upstream_gene_variant	LOC_Os02g09880	-	'35.2'_'not_assigned.unknown'
PGC2.2	snp_02_5765468	J	-0.75	1.07E-07	2	G	C	upstream_gene_variant	LOC_Os02g10860	-	'27.3.35'_'RNA.regulation_of_transcription.bZIP_transcription_factor_family'
PGC2.3	snp_02_6019127	J	-0.93	2.44E-06	2	T	A	upstream_gene_variant	LOC_Os02g11700	-	'26.2'_'misc.UDP_glucosyl_and_glucoronyl_transferases'
PGC2.3	snp_02_6022267	J	-0.91	7.77E-07	2	G	A	upstream_gene_variant	LOC_Os02g11700	-	'26.2'_'misc.UDP_glucosyl_and_glucoronyl_transferases'

QTL ID	SNP ID	Allele†	Beta	P-value	Chr	Ref	Alt	Region	Gene	Amino acid change	GoMAPMAN
PGC2.3	snp_02_6093939	J	-0.91	7.88E-07	2	G	T	downstream_gene_variant	LOC_Os02g11780	-	'27.3.67'_'RNA.regulation_of_transcription.putative_transcription_regulator'
PGC2.3	snp_02_6114633	J	-0.91	7.77E-07	2	A	G	missense_variant	LOC_Os02g11820	p.Thr439Ala	'30.5'_'signalling.G-proteins'
PGC2.3	snp_02_6115119	J	-0.91	7.77E-07	2	C	T	3_prime_UTR_variant	LOC_Os02g11820	-	'30.5'_'signalling.G-proteins'
PGC2.3	snp_02_6117330	J	-0.91	7.77E-07	2	T	A	missense_variant	LOC_Os02g11820	p.Leu646His	'30.5'_'signalling.G-proteins'
PGC2.3	snp_02_6117690	J	-0.91	7.77E-07	2	A	T	downstream_gene_variant	LOC_Os02g11830	-	'31.4'_'cell.vesicle_transport'
PGC2.3	snp_02_6119022	J	-0.91	7.77E-07	2	T	C	downstream_gene_variant	LOC_Os02g11820	-	'30.5'_'signalling.G-proteins'
PGC2.3	snp_02_6119553	J	-0.91	7.81E-07	2	A	T	downstream_gene_variant	LOC_Os02g11820	-	'30.5'_'signalling.G-proteins'
PGC2.3	snp_02_6123889	J	-0.91	7.77E-07	2	G	A	intron_variant	LOC_Os02g11830	-	'31.4'_'cell.vesicle_transport'
PGC2.3	snp_02_6124371	J	-0.90	8.20E-07	2	C	T	intron_variant	LOC_Os02g11830	-	'31.4'_'cell.vesicle_transport'
PGC2.3	snp_02_6124608	J	-0.91	7.92E-07	2	C	T	intron_variant	LOC_Os02g11830	-	'31.4'_'cell.vesicle_transport'
PGC2.3	snp_02_6125333	J	-0.91	7.77E-07	2	G	T	synonymous_variant	LOC_Os02g11830	p.Thr351Thr	'31.4'_'cell.vesicle_transport'
PGC2.3	snp_02_6125893	J	-0.90	8.57E-07	2	C	A	intron_variant	LOC_Os02g11830	-	'31.4'_'cell.vesicle_transport'
PGC2.3	snp_02_6126687	J	-0.91	7.77E-07	2	T	C	intron_variant	LOC_Os02g11830	-	'31.4'_'cell.vesicle_transport'
PGC2.3	snp_02_6128937	J	-0.92	1.27E-06	2	C	T	downstream_gene_variant	LOC_Os02g11840	-	'31.4'_'cell.vesicle_transport'
PGC2.3	snp_02_6128987	J	-0.91	7.83E-07	2	C	A	downstream_gene_variant	LOC_Os02g11840	-	'31.4'_'cell.vesicle_transport'
PGC2.3	snp_02_6129161	J	-0.91	7.77E-07	2	A	G	5_prime_UTR_variant	LOC_Os02g11830	-	'31.4'_'cell.vesicle_transport'
PGC2.3	snp_02_6129166	J	-0.91	7.77E-07	2	C	T	5_prime_UTR_variant	LOC_Os02g11830	-	'31.4'_'cell.vesicle_transport'
PGC2.3	snp_02_6129259	J	-0.91	7.77E-07	2	T	A	upstream_gene_variant	LOC_Os02g11830	-	'31.4'_'cell.vesicle_transport'
PGC2.3	snp_02_6129357	J	-0.91	7.77E-07	2	A	G	upstream_gene_variant	LOC_Os02g11830	-	'31.4'_'cell.vesicle_transport'
PGC2.3	snp_02_6129363	J	-0.91	7.77E-07	2	T	C	upstream_gene_variant	LOC_Os02g11830	-	'31.4'_'cell.vesicle_transport'
PGC2.3	snp_02_6129392	J	-0.91	7.93E-07	2	G	T	upstream_gene_variant	LOC_Os02g11830	-	'31.4'_'cell.vesicle_transport'
PGC2.3	snp_02_6129396	J	-0.90	8.37E-07	2	G	C	upstream_gene_variant	LOC_Os02g11830	-	'31.4'_'cell.vesicle_transport'
PGC2.3	snp_02_6129405	J	-0.91	7.77E-07	2	A	G	upstream_gene_variant	LOC_Os02g11830	-	'31.4'_'cell.vesicle_transport'
PGC2.3	snp_02_6129516	J	-0.91	7.93E-07	2	G	A	upstream_gene_variant	LOC_Os02g11830	-	'31.4'_'cell.vesicle_transport'
PGC2.3	snp_02_6129603	J	-0.91	7.64E-07	2	A	G	upstream_gene_variant	LOC_Os02g11830	-	'31.4'_'cell.vesicle_transport'
PGC2.3	snp_02_6129611	J	-0.91	7.65E-07	2	C	T	upstream_gene_variant	LOC_Os02g11830	-	'31.4'_'cell.vesicle_transport'
PGC2.3	snp_02_6129636	J	-0.91	7.45E-07	2	C	G	upstream_gene_variant	LOC_Os02g11830	-	'31.4'_'cell.vesicle_transport'
PGC2.3	snp_02_6129692	J	-0.91	7.77E-07	2	C	T	upstream_gene_variant	LOC_Os02g11830	-	'31.4'_'cell.vesicle_transport'
PGC2.3	snp_02_6129720	J	-0.91	7.77E-07	2	C	T	upstream_gene_variant	LOC_Os02g11830	-	'31.4'_'cell.vesicle_transport'
PGC2.3	snp_02_6129726	J	-0.91	7.71E-07	2	C	T	upstream_gene_variant	LOC_Os02g11830	-	'31.4'_'cell.vesicle_transport'
PGC2.3	snp_02_6129752	J	-0.99	2.88E-07	2	T	C	upstream_gene_variant	LOC_Os02g11830	-	'31.4'_'cell.vesicle_transport'
PGC2.3	snp_02_6129811	J	-0.99	2.84E-07	2	T	C	upstream_gene_variant	LOC_Os02g11830	-	'31.4'_'cell.vesicle_transport'
PGC2.3	snp_02_6129843	J	-0.91	6.87E-07	2	A	C	upstream_gene_variant	LOC_Os02g11830	-	'31.4'_'cell.vesicle_transport'
PGC2.3	snp_02_6129849	J	-0.91	8.01E-07	2	G	T	upstream_gene_variant	LOC_Os02g11830	-	'31.4'_'cell.vesicle_transport'
PGC2.3	snp_02_6129879	J	-0.91	7.79E-07	2	C	T	upstream_gene_variant	LOC_Os02g11830	-	'31.4'_'cell.vesicle_transport'
PGC2.3	snp_02_6129907	J	-0.91	7.77E-07	2	G	A	upstream_gene_variant	LOC_Os02g11830	-	'31.4'_'cell.vesicle_transport'
PGC2.3	snp_02_6129952	J	-0.91	7.77E-07	2	G	A	upstream_gene_variant	LOC_Os02g11830	-	'31.4'_'cell.vesicle_transport'
PGC2.3	snp_02_6129958	J	-0.91	7.77E-07	2	A	G	upstream_gene_variant	LOC_Os02g11830	-	'31.4'_'cell.vesicle_transport'
PGC2.3	snp_02_6130017	J	-0.91	7.77E-07	2	T	C	upstream_gene_variant	LOC_Os02g11830	-	'31.4'_'cell.vesicle_transport'
PGC2.3	snp_02_6130040	J	-0.90	8.45E-07	2	G	C	upstream_gene_variant	LOC_Os02g11830	-	'31.4'_'cell.vesicle_transport'
PGC2.3	snp_02_6130042	J	-0.91	7.70E-07	2	A	G	upstream_gene_variant	LOC_Os02g11830	-	'31.4'_'cell.vesicle_transport'
PGC2.3	snp_02_6130091	J	-0.91	7.77E-07	2	T	C	upstream_gene_variant	LOC_Os02g11830	-	'31.4'_'cell.vesicle_transport'

QTL ID	SNP ID	Allele†	Beta	P-value	Chr	Ref	Alt	Region	Gene	Amino acid change	GoMAPMAN
PGC2.3	snp_02_6130170	J	-0.99	2.84E-07	2	A	G	upstream_gene_variant	LOC_Os02g11830	-	'31.4'_'cell.vesicle_transport'
PGC2.3	snp_02_6130172	J	-0.99	2.55E-07	2	C	T	upstream_gene_variant	LOC_Os02g11830	-	'31.4'_'cell.vesicle_transport'
PGC2.3	snp_02_6130182	J	-0.91	7.68E-07	2	C	A	upstream_gene_variant	LOC_Os02g11830	-	'31.4'_'cell.vesicle_transport'
PGC2.3	snp_02_6130265	J	-0.91	7.24E-07	2	C	T	upstream_gene_variant	LOC_Os02g11830	-	'31.4'_'cell.vesicle_transport'
PGC2.3	snp_02_6130266	J	-0.91	7.77E-07	2	A	G	upstream_gene_variant	LOC_Os02g11830	-	'31.4'_'cell.vesicle_transport'
PGC2.3	snp_02_6130274	J	-0.91	7.77E-07	2	C	T	upstream_gene_variant	LOC_Os02g11830	-	'31.4'_'cell.vesicle_transport'
PGC2.3	snp_02_6130286	J	-0.91	6.80E-07	2	A	T	upstream_gene_variant	LOC_Os02g11830	-	'31.4'_'cell.vesicle_transport'
PGC2.3	snp_02_6130307	J	-0.91	7.77E-07	2	A	G	upstream_gene_variant	LOC_Os02g11830	-	'31.4'_'cell.vesicle_transport'
PGC2.3	snp_02_6130318	J	-0.91	7.73E-07	2	A	G	upstream_gene_variant	LOC_Os02g11830	-	'31.4'_'cell.vesicle_transport'
PGC2.3	snp_02_6130323	J	-0.91	7.77E-07	2	A	G	upstream_gene_variant	LOC_Os02g11830	-	'31.4'_'cell.vesicle_transport'
PGC2.3	snp_02_6130376	J	-0.91	7.98E-07	2	G	T	upstream_gene_variant	LOC_Os02g11830	-	'31.4'_'cell.vesicle_transport'
PGC2.3	snp_02_6130401	J	-0.91	7.64E-07	2	C	T	upstream_gene_variant	LOC_Os02g11830	-	'31.4'_'cell.vesicle_transport'
PGC2.3	snp_02_6130419	J	-0.91	7.77E-07	2	T	C	upstream_gene_variant	LOC_Os02g11830	-	'31.4'_'cell.vesicle_transport'
PGC2.3	snp_02_6130427	J	-0.91	7.76E-07	2	A	G	upstream_gene_variant	LOC_Os02g11830	-	'31.4'_'cell.vesicle_transport'
PGC2.3	snp_02_6130451	J	-0.91	7.78E-07	2	G	A	upstream_gene_variant	LOC_Os02g11830	-	'31.4'_'cell.vesicle_transport'
PGC2.3	snp_02_6130511	J	-0.91	7.66E-07	2	T	A	upstream_gene_variant	LOC_Os02g11830	-	'31.4'_'cell.vesicle_transport'
PGC2.3	snp_02_6130525	J	-0.91	7.77E-07	2	C	T	upstream_gene_variant	LOC_Os02g11830	-	'31.4'_'cell.vesicle_transport'
PGC2.3	snp_02_6130555	J	-0.91	7.77E-07	2	G	A	upstream_gene_variant	LOC_Os02g11830	-	'31.4'_'cell.vesicle_transport'
PGC2.3	snp_02_6131038	J	-0.91	7.49E-07	2	T	C	upstream_gene_variant	LOC_Os02g11830	-	'31.4'_'cell.vesicle_transport'
PGC2.3	snp_02_6131074	J	-0.90	8.15E-07	2	C	T	upstream_gene_variant	LOC_Os02g11830	-	'31.4'_'cell.vesicle_transport'
PGC2.3	snp_02_6131080	J	-0.91	7.83E-07	2	T	C	upstream_gene_variant	LOC_Os02g11830	-	'31.4'_'cell.vesicle_transport'
PGC2.3	snp_02_6131113	J	-0.91	7.21E-07	2	C	T	upstream_gene_variant	LOC_Os02g11830	-	'31.4'_'cell.vesicle_transport'
PGC2.3	snp_02_6131143	J	-0.91	7.49E-07	2	G	A	upstream_gene_variant	LOC_Os02g11830	-	'31.4'_'cell.vesicle_transport'
PGC2.3	snp_02_6131170	J	-0.91	7.76E-07	2	C	T	upstream_gene_variant	LOC_Os02g11830	-	'31.4'_'cell.vesicle_transport'
PGC2.3	snp_02_6131271	J	-0.90	8.34E-07	2	T	C	upstream_gene_variant	LOC_Os02g11830	-	'31.4'_'cell.vesicle_transport'
PGC2.3	snp_02_6131344	J	-0.91	8.05E-07	2	C	T	upstream_gene_variant	LOC_Os02g11830	-	'31.4'_'cell.vesicle_transport'
PGC2.3	snp_02_6131374	J	-0.91	7.90E-07	2	C	T	upstream_gene_variant	LOC_Os02g11830	-	'31.4'_'cell.vesicle_transport'
PGC2.3	snp_02_6131376	J	-0.91	7.90E-07	2	C	T	upstream_gene_variant	LOC_Os02g11830	-	'31.4'_'cell.vesicle_transport'
PGC2.3	snp_02_6131400	J	-0.91	8.02E-07	2	T	C	upstream_gene_variant	LOC_Os02g11830	-	'31.4'_'cell.vesicle_transport'
PGC2.3	snp_02_6131433	J	-0.91	7.92E-07	2	C	A	upstream_gene_variant	LOC_Os02g11830	-	'31.4'_'cell.vesicle_transport'
PGC2.3	snp_02_6131447	J	-0.91	7.92E-07	2	A	C	upstream_gene_variant	LOC_Os02g11830	-	'31.4'_'cell.vesicle_transport'
PGC2.3	snp_02_6131453	J	-0.91	7.98E-07	2	G	T	upstream_gene_variant	LOC_Os02g11830	-	'31.4'_'cell.vesicle_transport'
PGC2.3	snp_02_6131480	J	-0.91	7.77E-07	2	T	G	upstream_gene_variant	LOC_Os02g11830	-	'31.4'_'cell.vesicle_transport'
PGC2.3	snp_02_6131535	J	-0.91	7.99E-07	2	C	T	upstream_gene_variant	LOC_Os02g11830	-	'31.4'_'cell.vesicle_transport'
PGC2.3	snp_02_6131603	J	-0.91	7.99E-07	2	C	T	upstream_gene_variant	LOC_Os02g11830	-	'31.4'_'cell.vesicle_transport'
PGC2.3	snp_02_6131607	J	-0.91	7.98E-07	2	C	T	upstream_gene_variant	LOC_Os02g11830	-	'31.4'_'cell.vesicle_transport'
PGC2.3	snp_02_6131616	J	-0.91	7.77E-07	2	C	G	upstream_gene_variant	LOC_Os02g11830	-	'31.4'_'cell.vesicle_transport'
PGC2.3	snp_02_6131626	J	-0.91	7.69E-07	2	C	G	upstream_gene_variant	LOC_Os02g11830	-	'31.4'_'cell.vesicle_transport'
PGC2.3	snp_02_6131633	J	-0.91	7.77E-07	2	C	T	upstream_gene_variant	LOC_Os02g11830	-	'31.4'_'cell.vesicle_transport'
PGC2.3	snp_02_6131643	J	-0.91	7.65E-07	2	A	T	upstream_gene_variant	LOC_Os02g11830	-	'31.4'_'cell.vesicle_transport'
PGC2.3	snp_02_6131663	J	-0.91	8.00E-07	2	C	T	upstream_gene_variant	LOC_Os02g11830	-	'31.4'_'cell.vesicle_transport'
PGC2.3	snp_02_6131743	J	-0.91	7.77E-07	2	T	A	upstream_gene_variant	LOC_Os02g11830	-	'31.4'_'cell.vesicle_transport'
PGC2.3	snp_02_6131967	J	-0.91	7.77E-07	2	C	T	3_prime_UTR_variant	LOC_Os02g11840	-	'31.4'_'cell.vesicle_transport'

QTL ID	SNP ID	Allele†	Beta	P-value	Chr	Ref	Alt	Region	Gene	Amino acid change	GoMAPMAN
PGC2.3	snp_02_6132038	J	-0.91	7.77E-07	2	C	T	splice_region_variant	LOC_Os02g11840	-	'31.4'_'cell.vesicle_transport'
PGC2.3	snp_02_6132420	J	-0.91	7.77E-07	2	G	A	upstream_gene_variant	LOC_Os02g11830	-	'31.4'_'cell.vesicle_transport'
PGC2.3	snp_02_6132433	J	-0.91	7.21E-07	2	T	G	upstream_gene_variant	LOC_Os02g11830	-	'31.4'_'cell.vesicle_transport'
PGC2.3	snp_02_6132489	J	-0.91	7.87E-07	2	G	A	upstream_gene_variant	LOC_Os02g11830	-	'31.4'_'cell.vesicle_transport'
PGC2.3	snp_02_6132735	J	-0.99	2.84E-07	2	A	G	upstream_gene_variant	LOC_Os02g11830	-	'31.4'_'cell.vesicle_transport'
PGC2.3	snp_02_6132887	J	-0.91	7.63E-07	2	C	T	upstream_gene_variant	LOC_Os02g11830	-	'31.4'_'cell.vesicle_transport'
PGC2.3	snp_02_6132919	J	-0.91	6.64E-07	2	T	C	missense_variant	LOC_Os02g11840	p.Thr564Ala	'31.4'_'cell.vesicle_transport'
PGC2.3	snp_02_6132954	J	-0.91	7.91E-07	2	A	T	missense_variant	LOC_Os02g11840	p.Leu552Gln	'31.4'_'cell.vesicle_transport'
PGC2.3	snp_02_6133056	J	-0.91	7.66E-07	2	C	T	upstream_gene_variant	LOC_Os02g11830	-	'31.4'_'cell.vesicle_transport'
PGC2.3	snp_02_6133101	J	-0.91	7.77E-07	2	C	T	upstream_gene_variant	LOC_Os02g11830	-	'31.4'_'cell.vesicle_transport'
PGC2.3	snp_02_6133171	J	-0.91	7.77E-07	2	C	T	upstream_gene_variant	LOC_Os02g11830	-	'31.4'_'cell.vesicle_transport'
PGC2.3	snp_02_6133188	J	-0.91	7.77E-07	2	C	G	upstream_gene_variant	LOC_Os02g11830	-	'31.4'_'cell.vesicle_transport'
PGC2.3	snp_02_6133534	J	-0.91	7.38E-07	2	T	C	upstream_gene_variant	LOC_Os02g11830	-	'31.4'_'cell.vesicle_transport'
PGC2.3	snp_02_6133621	J	-0.91	7.77E-07	2	G	T	upstream_gene_variant	LOC_Os02g11830	-	'31.4'_'cell.vesicle_transport'
PGC2.3	snp_02_6133677	J	-0.91	7.99E-07	2	C	T	upstream_gene_variant	LOC_Os02g11830	-	'31.4'_'cell.vesicle_transport'
PGC2.3	snp_02_6133740	J	-0.92	1.29E-06	2	T	C	upstream_gene_variant	LOC_Os02g11830	-	'31.4'_'cell.vesicle_transport'
PGC2.3	snp_02_6133795	J	-0.91	6.87E-07	2	C	T	upstream_gene_variant	LOC_Os02g11830	-	'31.4'_'cell.vesicle_transport'
PGC2.3	snp_02_6134366	J	-0.91	8.07E-07	2	C	A	intron_variant	LOC_Os02g11840	-	'31.4'_'cell.vesicle_transport'
PGC2.3	snp_02_6134372	J	-0.91	7.77E-07	2	G	A	intron_variant	LOC_Os02g11840	-	'31.4'_'cell.vesicle_transport'
PGC2.3	snp_02_6134375	J	-0.91	7.88E-07	2	G	T	intron_variant	LOC_Os02g11840	-	'31.4'_'cell.vesicle_transport'
PGC2.3	snp_02_6134467	J	-0.91	7.77E-07	2	A	G	intron_variant	LOC_Os02g11840	-	'31.4'_'cell.vesicle_transport'
PGC2.3	snp_02_6134523	J	-0.91	7.83E-07	2	A	G	intron_variant	LOC_Os02g11840	-	'31.4'_'cell.vesicle_transport'
PGC2.3	snp_02_6134556	J	-0.91	7.80E-07	2	T	A	intron_variant	LOC_Os02g11840	-	'31.4'_'cell.vesicle_transport'
PGC2.3	snp_02_6134585	J	-0.91	7.77E-07	2	G	C	intron_variant	LOC_Os02g11840	-	'31.4'_'cell.vesicle_transport'
PGC2.3	snp_02_6134699	J	-0.91	7.75E-07	2	G	T	intron_variant	LOC_Os02g11840	-	'31.4'_'cell.vesicle_transport'
PGC2.3	snp_02_6134790	J	-0.91	8.03E-07	2	G	A	intron_variant	LOC_Os02g11840	-	'31.4'_'cell.vesicle_transport'
PGC2.3	snp_02_6135032	J	-0.91	7.77E-07	2	C	G	intron_variant	LOC_Os02g11840	-	'31.4'_'cell.vesicle_transport'
PGC2.3	snp_02_6135247	J	-0.91	8.20E-07	2	A	G	intron_variant	LOC_Os02g11840	-	'31.4'_'cell.vesicle_transport'
PGC2.3	snp_02_6135260	J	-0.91	7.77E-07	2	T	C	intron_variant	LOC_Os02g11840	-	'31.4'_'cell.vesicle_transport'
PGC2.3	snp_02_6135392	J	-0.91	7.68E-07	2	C	G	intron_variant	LOC_Os02g11840	-	'31.4'_'cell.vesicle_transport'
PGC2.3	snp_02_6135618	J	-0.91	7.91E-07	2	A	G	intron_variant	LOC_Os02g11840	-	'31.4'_'cell.vesicle_transport'
PGC2.3	snp_02_6135944	J	-0.91	7.94E-07	2	C	T	intron_variant	LOC_Os02g11840	-	'31.4'_'cell.vesicle_transport'
PGC2.3	snp_02_6135974	J	-0.91	7.86E-07	2	G	A	intron_variant	LOC_Os02g11840	-	'31.4'_'cell.vesicle_transport'
PGC2.3	snp_02_6136052	J	-0.91	7.78E-07	2	C	T	synonymous_variant	LOC_Os02g11840	p.Arg248Arg	'31.4'_'cell.vesicle_transport'
PGC2.3	snp_02_6137015	J	-0.91	7.48E-07	2	A	G	intron_variant	LOC_Os02g11840	-	'31.4'_'cell.vesicle_transport'
PGC2.3	snp_02_6137165	J	-0.91	7.43E-07	2	T	C	intron_variant	LOC_Os02g11840	-	'31.4'_'cell.vesicle_transport'
PGC2.3	snp_02_6137595	J	-0.91	7.77E-07	2	A	T	intron_variant	LOC_Os02g11840	-	'31.4'_'cell.vesicle_transport'
PGC2.3	snp_02_6137632	J	-0.91	7.77E-07	2	A	T	intron_variant	LOC_Os02g11840	-	'31.4'_'cell.vesicle_transport'
PGC2.3	snp_02_6137803	J	-0.88	2.54E-06	2	G	A	intron_variant	LOC_Os02g11840	-	'31.4'_'cell.vesicle_transport'
PGC2.3	snp_02_6138174	J	-0.91	7.81E-07	2	A	C	upstream_gene_variant	LOC_Os02g11840	-	'31.4'_'cell.vesicle_transport'
PGC2.3	snp_02_6138749	J	-0.91	7.72E-07	2	T	C	upstream_gene_variant	LOC_Os02g11840	-	'31.4'_'cell.vesicle_transport'
PGC2.3	snp_02_6139109	J	-0.91	7.84E-07	2	A	G	upstream_gene_variant	LOC_Os02g11840	-	'31.4'_'cell.vesicle_transport'
PGC2.3	snp_02_6139484	J	-0.91	7.77E-07	2	C	T	upstream_gene_variant	LOC_Os02g11840	-	'31.4'_'cell.vesicle_transport'

QTL ID	SNP ID	Allele†	Beta	P-value	Chr	Ref	Alt	Region	Gene	Amino acid change	GoMAPMAN
PGC2.3	snp_02_6139485	J	-0.91	7.91E-07	2	C	T	upstream_gene_variant	LOC_Os02g11840	-	'31.4'_'cell.vesicle_transport'
PGC2.3	snp_02_6139493	J	-0.91	7.77E-07	2	A	G	upstream_gene_variant	LOC_Os02g11840	-	'31.4'_'cell.vesicle_transport'
PGC2.3	snp_02_6139514	J	-0.91	7.69E-07	2	C	T	upstream_gene_variant	LOC_Os02g11840	-	'31.4'_'cell.vesicle_transport'
PGC2.3	snp_02_6139527	J	-0.91	7.77E-07	2	T	G	upstream_gene_variant	LOC_Os02g11840	-	'31.4'_'cell.vesicle_transport'
PGC2.3	snp_02_6139614	J	-0.91	7.76E-07	2	C	T	upstream_gene_variant	LOC_Os02g11840	-	'31.4'_'cell.vesicle_transport'
PGC2.3	snp_02_6139878	J	-0.92	2.10E-06	2	T	C	upstream_gene_variant	LOC_Os02g11840	-	'31.4'_'cell.vesicle_transport'
PGC2.3	snp_02_6140562	J	-0.94	2.10E-06	2	C	A	upstream_gene_variant	LOC_Os02g11840	-	'31.4'_'cell.vesicle_transport'
PGC2.3	snp_02_6140571	J	-0.91	7.73E-07	2	A	T	upstream_gene_variant	LOC_Os02g11840	-	'31.4'_'cell.vesicle_transport'
PGC2.3	snp_02_6140690	J	-0.91	7.96E-07	2	T	C	upstream_gene_variant	LOC_Os02g11840	-	'31.4'_'cell.vesicle_transport'
PGC2.3	snp_02_6140733	J	-0.91	7.77E-07	2	C	G	upstream_gene_variant	LOC_Os02g11840	-	'31.4'_'cell.vesicle_transport'
PGC2.3	snp_02_6140770	J	-0.91	7.77E-07	2	G	T	upstream_gene_variant	LOC_Os02g11840	-	'31.4'_'cell.vesicle_transport'
PGC2.3	snp_02_6140778	J	-0.91	7.77E-07	2	G	T	upstream_gene_variant	LOC_Os02g11840	-	'31.4'_'cell.vesicle_transport'
PGC2.3	snp_02_6141091	J	-0.91	8.00E-07	2	C	T	upstream_gene_variant	LOC_Os02g11840	-	'31.4'_'cell.vesicle_transport'
PGC2.3	snp_02_6141338	J	-0.91	7.68E-07	2	C	A	upstream_gene_variant	LOC_Os02g11840	-	'31.4'_'cell.vesicle_transport'
PGC2.3	snp_02_6141398	J	-0.91	7.37E-07	2	C	G	upstream_gene_variant	LOC_Os02g11840	-	'31.4'_'cell.vesicle_transport'
PGC2.3	snp_02_6141399	J	-0.91	7.77E-07	2	G	A	upstream_gene_variant	LOC_Os02g11840	-	'31.4'_'cell.vesicle_transport'
PGC2.3	snp_02_6141586	J	-0.91	8.07E-07	2	G	A	upstream_gene_variant	LOC_Os02g11840	-	'31.4'_'cell.vesicle_transport'
PGC2.3	snp_02_6141602	J	-0.91	7.84E-07	2	G	A	upstream_gene_variant	LOC_Os02g11840	-	'31.4'_'cell.vesicle_transport'
PGC2.3	snp_02_6141605	J	-0.91	7.64E-07	2	G	A	upstream_gene_variant	LOC_Os02g11840	-	'31.4'_'cell.vesicle_transport'
PGC2.3	snp_02_6141641	J	-0.91	7.57E-07	2	G	C	upstream_gene_variant	LOC_Os02g11840	-	'31.4'_'cell.vesicle_transport'
PGC2.3	snp_02_6142474	J	-0.91	7.79E-07	2	T	C	upstream_gene_variant	LOC_Os02g11840	-	'31.4'_'cell.vesicle_transport'
PGC2.3	snp_02_6142482	J	-0.91	7.79E-07	2	C	T	upstream_gene_variant	LOC_Os02g11840	-	'31.4'_'cell.vesicle_transport'
PGC2.3	snp_02_6142537	J	-0.91	7.97E-07	2	G	A	upstream_gene_variant	LOC_Os02g11840	-	'31.4'_'cell.vesicle_transport'
PGC2.3	snp_02_6143226	J	-0.91	7.77E-07	2	A	G	downstream_gene_variant	LOC_Os02g11850	-	'35.2'_'not_assigned.unknown'
PGC2.3	snp_02_6143453	J	-0.91	7.77E-07	2	C	G	downstream_gene_variant	LOC_Os02g11850	-	'35.2'_'not_assigned.unknown'
PGC2.3	snp_02_6143505	J	-0.91	7.63E-07	2	C	T	downstream_gene_variant	LOC_Os02g11850	-	'35.2'_'not_assigned.unknown'
PGC2.3	snp_02_6143510	J	-0.91	7.56E-07	2	A	G	downstream_gene_variant	LOC_Os02g11850	-	'35.2'_'not_assigned.unknown'
PGC2.3	snp_02_6143557	J	-0.91	7.76E-07	2	G	A	downstream_gene_variant	LOC_Os02g11850	-	'35.2'_'not_assigned.unknown'
PGC2.3	snp_02_6143606	J	-0.91	7.77E-07	2	C	T	downstream_gene_variant	LOC_Os02g11850	-	'35.2'_'not_assigned.unknown'
PGC2.3	snp_02_6145977	J	-0.91	7.22E-07	2	A	G	upstream_gene_variant	LOC_Os02g11850	-	'35.2'_'not_assigned.unknown'
PGC2.3	snp_02_6145993	J	-0.91	7.77E-07	2	T	C	3_prime_UTR_variant	LOC_Os02g11859	-	'35.2'_'not_assigned.unknown'
PGC2.3	snp_02_6146056	J	-0.91	7.77E-07	2	T	C	3_prime_UTR_variant	LOC_Os02g11859	-	'35.2'_'not_assigned.unknown'
PGC2.3	snp_02_6146070	J	-0.90	8.19E-07	2	G	T	3_prime_UTR_variant	LOC_Os02g11859	-	'35.2'_'not_assigned.unknown'
PGC2.3	snp_02_6153031	J	-0.91	8.00E-07	2	C	T	upstream_gene_variant	LOC_Os02g11870	-	'35.2'_'not_assigned.unknown'
PGC2.3	snp_02_6153039	J	-0.91	8.00E-07	2	C	T	upstream_gene_variant	LOC_Os02g11870	-	'35.2'_'not_assigned.unknown'
PGC2.3	snp_02_6153128	J	-0.91	7.77E-07	2	G	A	upstream_gene_variant	LOC_Os02g11870	-	'35.2'_'not_assigned.unknown'
PGC2.3	snp_02_6153715	J	-0.91	7.77E-07	2	G	A	upstream_gene_variant	LOC_Os02g11870	-	'35.2'_'not_assigned.unknown'
PGC2.3	snp_02_6155865	J	-0.99	2.84E-07	2	A	C	upstream_gene_variant	LOC_Os02g11880	-	'35.2'_'not_assigned.unknown'
PGC2.3	snp_02_6155922	J	-0.99	2.84E-07	2	C	T	upstream_gene_variant	LOC_Os02g11880	-	'35.2'_'not_assigned.unknown'
PGC2.3	snp_02_6158739	J	-0.91	7.77E-07	2	G	A	synonymous_variant	LOC_Os02g11890	p.Leu184Leu	'31.4'_'cell.vesicle_transport'
PGC2.3	snp_02_6764968	J	-1.06	2.98E-07	2	A	T	intergenic_region	LOC_Os02g12830-LOC_Os02g12840	-	NA
PGC2.4	snp_02_7100959	J	-1.20	3.35E-10	2	G	C	upstream_gene_variant	LOC_Os02g13320	-	'35.2'_'not_assigned.unknown'

QTL ID	SNP ID	Allele†	Beta	P-value	Chr	Ref	Alt	Region	Gene	Amino acid change	GoMAPMAN
PGC2.4	snp_02_7107571	J	-1.09	2.53E-08	2	T	C	downstream_gene_variant	LOC_Os02g13320	-	'35.2'_'not_assigned.unknown'
PGC2.4	snp_02_7107572	J	-1.09	2.55E-08	2	C	A	downstream_gene_variant	LOC_Os02g13320	-	'35.2'_'not_assigned.unknown'
PGC2.4	snp_02_7122058	J	-1.09	1.74E-08	2	T	C	upstream_gene_variant	LOC_Os02g13350	-	'23.3.3'_'nucleotide_metabolism.salvage.NUDIX_hydrolases'
PGC2.4	snp_02_7124199	J	-1.09	1.73E-08	2	G	C	upstream_gene_variant	LOC_Os02g13360	-	'35.1'_'not_assigned.no_ontology'
PGC2.4	snp_02_7128428	J	-1.09	1.72E-08	2	G	A	missense_variant	LOC_Os02g13360	p.Val345Ile	'35.1'_'not_assigned.no_ontology'
PGC2.4	snp_02_7131392	J	-1.09	1.73E-08	2	A	G	3_prime_UTR_variant	LOC_Os02g13360	-	'35.1'_'not_assigned.no_ontology'
PGC2.4	snp_02_7133054	J	-1.09	1.90E-08	2	A	G	missense_variant	LOC_Os02g13370	p.Leu119Ser	'35.2'_'not_assigned.unknown'
PGC2.4	snp_02_7133549	J	-1.09	1.72E-08	2	G	C	upstream_gene_variant	LOC_Os02g13370	-	'35.2'_'not_assigned.unknown'
PGC2.4	snp_02_7133676	J	-1.09	1.74E-08	2	A	C	upstream_gene_variant	LOC_Os02g13370	-	'35.2'_'not_assigned.unknown'
PGC2.4	snp_02_7134016	J	-1.09	1.73E-08	2	A	T	upstream_gene_variant	LOC_Os02g13370	-	'35.2'_'not_assigned.unknown'
PGC2.4	snp_02_7135385	J	-1.09	1.73E-08	2	A	G	3_prime_UTR_variant	LOC_Os02g13380	-	'35.2'_'not_assigned.unknown'
PGC2.4	snp_02_7135563	J	-1.09	1.71E-08	2	G	A	synonymous_variant	LOC_Os02g13380	p.Cys126Cys	'35.2'_'not_assigned.unknown'
PGC2.4	snp_02_7136961	J	-1.09	1.74E-08	2	G	T	upstream_gene_variant	LOC_Os02g13370	-	'35.2'_'not_assigned.unknown'
PGC2.5	snp_02_18033568	J	0.55	2.51E-07	2	G	A	intergenic_region	LOC_Os02g30300-LOC_Os02g30310	-	NA
PGC2.6	snp_02_20302120	J	0.39	2.81E-06	2	G	T	upstream_gene_variant	LOC_Os02g34000	-	'35.2'_'not_assigned.unknown'
PGC2.6	snp_02_20335697	J	0.40	1.14E-06	2	G	C	downstream_gene_variant	LOC_Os02g34040	-	'30.2.17'_'signalling.receptor_kinases.DUF_26'
PGC2.6	snp_02_20354910	J	0.43	8.56E-08	2	C	T	synonymous_variant	LOC_Os02g34060	p.Val120Val	'35.2'_'not_assigned.unknown'
PGC2.6	snp_02_20404432	J	0.45	2.89E-08	2	C	T	downstream_gene_variant	LOC_Os02g34120	-	'35.2'_'not_assigned.unknown'
PGC2.6	snp_02_20575016	J	0.47	9.75E-09	2	G	A	synonymous_variant	LOC_Os02g34380	p.Lys491Lys	'35.2'_'not_assigned.unknown'
PGC2.6	snp_02_20653171	J	-0.30	2.97E-06	2	G	A	5_prime_UTR_variant	LOC_Os02g34470	-	'35.2'_'not_assigned.unknown'
PGC2.6	snp_02_20668145	J	0.41	4.86E-07	2	C	T	synonymous_variant	LOC_Os02g34490	p.Leu853Leu	'35.1'_'not_assigned.no_ontology'
PGC2.6	snp_02_20668699	J	0.38	1.85E-06	2	G	A	synonymous_variant	LOC_Os02g34490	p.Lys1038Lys	'35.1'_'not_assigned.no_ontology'
PGC2.6	snp_02_20811506	J	-0.40	2.00E-07	2	C	A	missense_variant	LOC_Os02g34690	p.Leu18Ile	'35.1'_'not_assigned.no_ontology'
PGC2.6	snp_02_21013917	J	-0.98	1.01E-06	2	C	A	downstream_gene_variant	LOC_Os02g35020	-	'10.3.2'_'cell_wall.hemicellulose_synthesis.glucuronoxylan'
PGC2.6	snp_02_21014024	J	-0.98	1.03E-06	2	C	T	downstream_gene_variant	LOC_Os02g35020	-	'10.3.2'_'cell_wall.hemicellulose_synthesis.glucuronoxylan'
PGC2.6	snp_02_21021406	J	-0.98	1.01E-06	2	G	A	upstream_gene_variant	LOC_Os02g35020	-	'10.3.2'_'cell_wall.hemicellulose_synthesis.glucuronoxylan'
PGC2.6	snp_02_21021987	J	-0.98	9.87E-07	2	G	C	upstream_gene_variant	LOC_Os02g35020	-	'10.3.2'_'cell_wall.hemicellulose_synthesis.glucuronoxylan'
PGC2.6	snp_02_21026828	J	-0.98	1.00E-06	2	C	T	upstream_gene_variant	LOC_Os02g35039	-	'35.2'_'not_assigned.unknown'
PGC2.6	snp_02_21029550	J	-0.98	1.05E-06	2	A	T	intron_variant	LOC_Os02g35039	-	'35.2'_'not_assigned.unknown'
PGC2.6	snp_02_21031429	J	-0.98	1.03E-06	2	G	A	intron_variant	LOC_Os02g35039	-	'35.2'_'not_assigned.unknown'
PGC2.6	snp_02_21037472	J	-0.98	1.01E-06	2	A	G	downstream_gene_variant	LOC_Os02g35060	-	'35.2'_'not_assigned.unknown'
PGC2.6	snp_02_21071119	J	-0.97	1.08E-06	2	G	A	upstream_gene_variant	LOC_Os02g35100	-	27.3.62'_'RNA.regulation_of_transcription.Nucleosome/Chromatin_assembly_factor_group'
PGC2.6	snp_02_21385729	J	-0.59	2.12E-07	2	T	A	intergenic_region	LOC_Os02g35540-LOC_Os02g35560	-	NA
PGC2.6	snp_02_21389238	J	-0.57	1.77E-06	2	C	T	intergenic_region	LOC_Os02g35540-LOC_Os02g35560	-	NA

QTL ID	SNP ID	Allele†	Beta	P-value	Chr	Ref	Alt	Region	Gene	Amino acid change	GoMAPMAN
PGC2.6	snp_02_21594232	J	-0.38	2.30E-06	2	C	T	upstream_gene_variant	LOC_Os02g35950	-	'28.99'_'DNA.unspecified'
PGC2.6	snp_02_21603338	J	-0.38	2.62E-06	2	T	C	upstream_gene_variant	LOC_Os02g35960	-	'35.2'_'not_assigned.unknown'
PGC2.7	snp_02_25253542	J	0.43	5.40E-07	2	C	T	synonymous_variant	LOC_Os02g42020	p.Thr57Thr	'35.1'_'not_assigned.no_ontology'
PGC3.1	snp_03_778685	J	-1.07	1.41E-06	3	G	A	upstream_gene_variant	LOC_Os03g02280	-	'35.2'_'not_assigned.unknown'
PGC3.1	snp_03_795873	J	-1.07	1.41E-06	3	C	A	upstream_gene_variant	LOC_Os03g02320	-	'29.4'_'protein.postranslational_modification'
PGC3.1	snp_03_795874	J	-1.07	1.41E-06	3	A	C	upstream_gene_variant	LOC_Os03g02320	-	'29.4'_'protein.postranslational_modification'
PGC3.2	snp_03_3476464	J	-0.50	1.87E-06	3	T	A	upstream_gene_variant	LOC_Os03g06860	-	'30.11'_'signalling.light'
PGC3.3	snp_03_6461443	J	0.81	2.22E-06	3	T	A	downstream_gene_variant	LOC_Os03g12280	-	'35.2'_'not_assigned.unknown'
PGC3.3	snp_03_6471552	J	0.86	2.61E-06	3	G	A	intron_variant	LOC_Os03g12300	-	'35.2'_'not_assigned.unknown'
PGC3.3	snp_03_6504253	J	0.84	1.88E-06	3	T	A	upstream_gene_variant	LOC_Os03g12340	-	'35.2'_'not_assigned.unknown'
PGC3.3	snp_03_6509240	J	0.78	1.94E-06	3	G	A	upstream_gene_variant	LOC_Os03g12330	-	'35.2'_'not_assigned.unknown'
PGC3.3	snp_03_6536294	J	0.82	2.01E-06	3	A	G	upstream_gene_variant	LOC_Os03g12370	-	'27.3.23'_'RNA.regulation_of_transcription.HSF,Heat-shock_transcription_factor_family'
PGC3.3	snp_03_6619595	J	0.84	2.21E-06	3	G	A	intergenic_region	LOC_Os03g12500-LOC_Os03g12510	-	NA
PGC3.3	snp_03_6660602	J	0.78	1.52E-06	3	A	T	synonymous_variant	LOC_Os03g12570	p.Cys521Cys	'27.3.46'_'RNA.regulation_of_transcription.DNA_methyltransferases'
PGC3.3	snp_03_6728014	J	0.75	2.96E-06	3	G	A	intergenic_region	LOC_Os03g12640-LOC_Os03g12650	-	NA
PGC3.4	snp_03_7012241	J	0.54	3.74E-07	3	C	T	upstream_gene_variant	LOC_Os03g12980	-	'35.2'_'not_assigned.unknown'
PGC3.4	snp_03_7014371	J	0.54	3.74E-07	3	C	G	upstream_gene_variant	LOC_Os03g12980	-	'35.2'_'not_assigned.unknown'
PGC3.5	snp_03_8735678	J	-0.87	9.73E-08	3	C	T	synonymous_variant	LOC_Os03g15830	p.Leu445Leu	'28.99'_'DNA.unspecified'
PGC3.5	snp_03_9265469	J	-0.55	9.81E-07	3	G	A	upstream_gene_variant	LOC_Os03g16718	-	'35.2'_'not_assigned.unknown'
PGC3.6	snp_03_15024656	J	-0.80	2.22E-06	3	G	C	downstream_gene_variant	LOC_Os03g26260	-	'20.1'_'stress.biotic'
PGC3.7	snp_03_26840782	J	-0.36	1.38E-06	3	A	G	intergenic_region	LOC_Os03g47470-LOC_Os03g47480	-	NA
PGC3.8	snp_03_27649256	J	0.36	2.62E-06	3	G	T	upstream_gene_variant	LOC_Os03g48480	-	'11.9.4.5'_'lipid_metabolism.lipid_degradation.beta-oxidation.acyl-CoA_thioesterase'
PGC3.9	snp_03_30084300	J	-0.43	1.00E-06	3	G	A	synonymous_variant	LOC_Os03g52420	p.Arg61Arg	'35.2'_'not_assigned.unknown'
PGC3.9	snp_03_30149664	J	-0.43	9.52E-07	3	G	A	upstream_gene_variant	LOC_Os03g52550	-	'35.2'_'not_assigned.unknown'
PGC3.9	snp_03_30154139	J	-0.43	1.04E-06	3	A	T	upstream_gene_variant	LOC_Os03g52570	-	'11.3'_'lipid_metabolism.Phospholipid_synthesis'
PGC3.9	snp_03_30154942	J	-0.43	9.13E-07	3	C	T	upstream_gene_variant	LOC_Os03g52570	-	'11.3'_'lipid_metabolism.Phospholipid_synthesis'
PGC3.9	snp_03_30190541	J	0.32	1.95E-06	3	C	T	splice_region_variant&intror	LOC_Os03g52640	-	'29.2.2.3.3'_'protein.synthesis.ribosome_biogenesis.Pre-rRNA_processing_and_modifications.methylotransferases'
PGC3.9	snp_03_30191879	J	-0.44	3.51E-07	3	G	A	upstream_gene_variant	LOC_Os03g52640	-	'29.2.2.3.3'_'protein.synthesis.ribosome_biogenesis.Pre-rRNA_processing_and_modifications.methylotransferases'

QTL ID	SNP ID	Allele†	Beta	P-value	Chr	Ref	Alt	Region	Gene	Amino acid change	GoMAPMAN
PGC3.9	snp_03_30196218	J	0.34	1.96E-06	3	C	T	upstream_gene_variant	LOC_Os03g52640	-	'29.2.2.3.3'_'protein.synthesis.ribosome_biogenesis.Pre-rRNA_processing_and_modifications.methylotransferases'
PGC3.9	snp_03_30241680	J	-0.51	2.86E-07	3	G	A	downstream_gene_variant	LOC_Os03g52750	-	'31.3'_'cell.cycle'
PGC3.9	snp_03_30244613	J	0.34	1.56E-06	3	T	C	synonymous_variant	LOC_Os03g52760	p.Met355Met	'3.5'_'minor_CHO_metabolism.others'
PGC3.9	snp_03_30342438	J	0.34	2.00E-06	3	C	T	downstream_gene_variant	LOC_Os03g52900	-	'35.2'_'not_assigned.unknown'
PGC3.9	snp_03_30523640	J	0.62	3.31E-07	3	C	T	synonymous_variant	LOC_Os03g53220	p.Phe190Phe	28.1'_'DNA.synthesis/Chromatin_structure'
PGC3.9	snp_03_30734898	J	0.58	2.81E-06	3	A	G	upstream_gene_variant	LOC_Os03g53590	-	'35.2'_'not_assigned.unknown'
PGC3.9	snp_03_30791743	J	-0.49	1.32E-07	3	G	A	5_prime_UTR_variant	LOC_Os03g53700	-	'27.3.67'_'RNA.regulation_of_transcription.putative_transcription_regulator'
PGC4.1	snp_04_20000192	J	-0.60	1.51E-06	4	T	C	downstream_gene_variant	LOC_Os04g33030	-	'35.2'_'not_assigned.unknown'
PGC4.2	snp_04_21339618	J	-0.68	3.05E-06	4	C	T	upstream_gene_variant	LOC_Os04g35100	-	'30.6'_'signalling.MAP_kinases'
PGC4.2	snp_04_21430298	J	-0.79	6.00E-07	4	A	T	upstream_gene_variant	LOC_Os04g35250	-	'27.3.21'_'RNA.regulation_of_transcription.GRAS_transcription_factor_family'
PGC4.3	snp_04_26749483	J	-1.02	3.07E-06	4	G	A	upstream_gene_variant	LOC_Os04g45270	-	'29.5.4'_'protein.degradation.aspartate_protease'
PGC4.4	snp_04_29923839	J	0.30	9.47E-07	4	C	T	downstream_gene_variant	LOC_Os04g50164	-	'35.2'_'not_assigned.unknown'
PGC4.4	snp_04_29925769	J	0.31	5.97E-07	4	C	T	downstream_gene_variant	LOC_Os04g50164	-	'35.2'_'not_assigned.unknown'
PGC4.4	snp_04_30106843	J	0.34	1.53E-06	4	C	T	downstream_gene_variant	LOC_Os04g50880	-	'1.3.12'_'PS.calvin_cycle.PRK'
PGC4.4	snp_04_30146751	J	0.33	2.31E-06	4	G	A	synonymous_variant	LOC_Os04g50930	p.Arg173Arg	'34.13'_'transport.peptides_and_oligopeptides'
PGC4.4	snp_04_30464183	J	0.32	2.97E-06	4	C	T	synonymous_variant	LOC_Os04g51440	p.Gln936Gln	'31.1.1.1.3'_'cell.organisation.cytoskeleton.actin.actin_binding'
PGC4.4	snp_04_30679307	J	-0.65	1.92E-06	4	C	G	3_prime_UTR_variant	LOC_Os04g51770	-	'35.2'_'not_assigned.unknown'
PGC4.4	snp_04_30786975	J	0.33	2.06E-06	4	A	G	upstream_gene_variant	LOC_Os04g51900	-	'29.3.1'_'protein.targeting.nucleus'
PGC4.4	snp_04_30850022	J	-0.65	1.93E-06	4	A	G	downstream_gene_variant	LOC_Os04g51960	-	'35.2'_'not_assigned.unknown'
PGC4.4	snp_04_30864427	J	0.32	2.89E-06	4	G	A	downstream_gene_variant	LOC_Os04g51980	-	'16.2'_'secondary_metabolism.phenylpropanoids'
PGC4.4	snp_04_30887509	J	-0.79	2.78E-08	4	G	A	downstream_gene_variant	LOC_Os04g52020	-	'33.99'_'development.unspecified'
PGC4.4	snp_04_30926649	J	0.33	2.46E-06	4	G	A	upstream_gene_variant	LOC_Os04g52060	-	'35.2'_'not_assigned.unknown'
PGC4.4	snp_04_30942194	J	0.35	5.10E-07	4	C	T	downstream_gene_variant	LOC_Os04g52090	-	'35.2'_'not_assigned.unknown'
PGC4.4	snp_04_30942215	J	0.36	2.24E-07	4	G	A	downstream_gene_variant	LOC_Os04g52090	-	'35.2'_'not_assigned.unknown'
PGC4.4	snp_04_31005695	J	0.40	1.80E-07	4	G	T	downstream_gene_variant	LOC_Os04g52180	-	'35.2'_'not_assigned.unknown'
PGC4.4	snp_04_31009177	J	0.38	3.28E-07	4	T	A	downstream_gene_variant	LOC_Os04g52190	-	'29.3.4.3'_'protein.targeting.secretory_pathway.vacuole'
PGC4.4	snp_04_31024611	J	0.39	1.66E-07	4	A	G	upstream_gene_variant	LOC_Os04g52220	-	'35.2'_'not_assigned.unknown'
PGC4.4	snp_04_31024760	J	0.44	2.68E-09	4	T	C	synonymous_variant	LOC_Os04g52220	p.Ser39Ser	'35.2'_'not_assigned.unknown'
PGC4.4	snp_04_31024925	J	0.43	6.64E-09	4	T	C	synonymous_variant	LOC_Os04g52220	p.Val94Val	'35.2'_'not_assigned.unknown'
PGC4.4	snp_04_31025026	J	0.41	4.44E-08	4	G	T	synonymous_variant	LOC_Os04g52220	p.Leu128Leu	'35.2'_'not_assigned.unknown'
PGC4.4	snp_04_31025109	J	0.44	3.23E-09	4	T	A	upstream_gene_variant	LOC_Os04g52230	-	'17.6.1.2'_'hormone_metabolism.gibberelin.synthesis-degradation.ent-kaurene_synthase'
PGC4.4	snp_04_31025209	J	0.43	1.39E-08	4	G	T	upstream_gene_variant	LOC_Os04g52230	-	'17.6.1.2'_'hormone_metabolism.gibberelin.synthesis-degradation.ent-kaurene_synthase'
PGC4.4	snp_04_31027329	J	0.41	2.62E-08	4	G	A	downstream_gene_variant	LOC_Os04g52210	-	17.6.1.2'_'hormone_metabolism.gibberelin.synthesis-degradation.ent-kaurene_synthase'

QTL ID	SNP ID	Allele†	Beta	P-value	Chr	Ref	Alt	Region	Gene	Amino acid change	GoMAPMAN
PGC4.4	snp_04_31029025	J	0.43	9.11E-09	4	C	A	synonymous_variant	LOC_Os04g52230	p.Ile387Ile	'17.6.1.2'_'hormone_metabolism.gibberelin.synthesis-degradation.ent-kaurene_synthase'
PGC4.4	snp_04_31029056	J	0.43	8.43E-09	4	T	A	synonymous_variant	LOC_Os04g52230	p.Lys397Lys	'17.6.1.2'_'hormone_metabolism.gibberelin.synthesis-degradation.ent-kaurene_synthase'
PGC4.4	snp_04_31029425	J	0.42	2.06E-08	4	C	T	downstream_gene_variant	LOC_Os04g52220	-	'35.2'_'not_assigned.unknown'
PGC4.4	snp_04_31030123	J	0.43	7.00E-09	4	C	A	upstream_gene_variant	LOC_Os04g52240	-	'17.6.1.2'_'hormone_metabolism.gibberelin.synthesis-degradation.ent-kaurene_synthase'
PGC4.4	snp_04_31030699	J	0.40	6.26E-08	4	A	G	synonymous_variant	LOC_Os04g52230	p.Ser705Ser	'17.6.1.2'_'hormone_metabolism.gibberelin.synthesis-degradation.ent-kaurene_synthase'
PGC4.4	snp_04_31032653	J	0.31	1.02E-06	4	A	G	upstream_gene_variant	LOC_Os04g52240	-	'17.6.1.2'_'hormone_metabolism.gibberelin.synthesis-degradation.ent-kaurene_synthase'
PGC4.4	snp_04_31032724	J	0.42	2.68E-08	4	T	A	upstream_gene_variant	LOC_Os04g52240	-	'17.6.1.2'_'hormone_metabolism.gibberelin.synthesis-degradation.ent-kaurene_synthase'
PGC4.4	snp_04_31032879	J	0.43	1.24E-08	4	G	A	upstream_gene_variant	LOC_Os04g52240	-	'17.6.1.2'_'hormone_metabolism.gibberelin.synthesis-degradation.ent-kaurene_synthase'
PGC4.4	snp_04_31033053	J	0.38	6.43E-07	4	G	A	upstream_gene_variant	LOC_Os04g52240	-	'17.6.1.2'_'hormone_metabolism.gibberelin.synthesis-degradation.ent-kaurene_synthase'
PGC4.4	snp_04_31033185	J	0.41	4.12E-08	4	C	T	upstream_gene_variant	LOC_Os04g52240	-	'17.6.1.2'_'hormone_metabolism.gibberelin.synthesis-degradation.ent-kaurene_synthase'
PGC4.4	snp_04_31033239	J	0.41	2.80E-08	4	C	A	upstream_gene_variant	LOC_Os04g52240	-	'17.6.1.2'_'hormone_metabolism.gibberelin.synthesis-degradation.ent-kaurene_synthase'
PGC4.4	snp_04_31035500	J	0.39	1.41E-07	4	A	T	downstream_gene_variant	LOC_Os04g52230	-	'17.6.1.2'_'hormone_metabolism.gibberelin.synthesis-degradation.ent-kaurene_synthase'
PGC4.4	snp_04_31035595	J	0.42	2.50E-08	4	G	A	downstream_gene_variant	LOC_Os04g52230	-	'17.6.1.2'_'hormone_metabolism.gibberelin.synthesis-degradation.ent-kaurene_synthase'
PGC4.4	snp_04_31035704	J	0.43	8.00E-09	4	T	A	downstream_gene_variant	LOC_Os04g52230	-	'17.6.1.2'_'hormone_metabolism.gibberelin.synthesis-degradation.ent-kaurene_synthase'
PGC4.4	snp_04_31041080	J	0.42	6.63E-09	4	G	T	3_prime_UTR_variant	LOC_Os04g52240	-	'17.6.1.2'_'hormone_metabolism.gibberelin.synthesis-degradation.ent-kaurene_synthase'
PGC4.4	snp_04_31041640	J	0.41	1.59E-08	4	C	A	intron_variant	LOC_Os04g52240	-	'17.6.1.2'_'hormone_metabolism.gibberelin.synthesis-degradation.ent-kaurene_synthase'
PGC4.4	snp_04_31041648	J	0.41	1.01E-08	4	A	T	intron_variant	LOC_Os04g52240	-	'17.6.1.2'_'hormone_metabolism.gibberelin.synthesis-degradation.ent-kaurene_synthase'
PGC4.4	snp_04_31041948	J	0.35	7.18E-07	4	T	C	intron_variant	LOC_Os04g52240	-	'17.6.1.2'_'hormone_metabolism.gibberelin.synthesis-degradation.ent-kaurene_synthase'
PGC4.4	snp_04_31053667	I	-0.35	3.63E-07	4	C	T	downstream_gene_variant	LOC_Os04g52250	-	'35.2'_'not_assigned.unknown'
PGC4.4	snp_04_31054077	I	-0.33	2.12E-06	4	C	T	downstream_gene_variant	LOC_Os04g52250	-	'35.2'_'not_assigned.unknown'
PGC4.4	snp_04_31056149	J	0.32	1.47E-06	4	C	T	downstream_gene_variant	LOC_Os04g52250	-	'35.2'_'not_assigned.unknown'
PGC4.4	snp_04_31062488	J	-0.35	5.56E-07	4	T	C	3_prime_UTR_variant	LOC_Os04g52260	-	'35.2'_'not_assigned.unknown'
PGC4.4	snp_04_31062729	J	-0.35	4.50E-07	4	T	C	downstream_gene_variant	LOC_Os04g52260	-	'35.2'_'not_assigned.unknown'
PGC4.4	snp_04_31063102	J	-0.36	2.80E-07	4	C	T	downstream_gene_variant	LOC_Os04g52260	-	'35.2'_'not_assigned.unknown'
PGC4.4	snp_04_31063121	J	-0.36	2.64E-07	4	G	T	downstream_gene_variant	LOC_Os04g52260	-	'35.2'_'not_assigned.unknown'
PGC4.4	snp_04_31063198	J	-0.34	5.60E-07	4	C	T	downstream_gene_variant	LOC_Os04g52260	-	'35.2'_'not_assigned.unknown'

QTL ID	SNP ID	Allele†	Beta	P-value	Chr	Ref	Alt	Region	Gene	Amino acid change	GoMAPMAN
PGC4.4	snp_04_31065971	J	-0.38	5.02E-08	4	A	G	upstream_gene_variant	LOC_Os04g52280	-	'16.2.1.10'_'secondary_metabolism.phenylpropanoids.lignin_biosynthesis.CAD'
PGC4.4	snp_04_31066005	J	0.33	1.21E-06	4	G	A	upstream_gene_variant	LOC_Os04g52280	-	'16.2.1.10'_'secondary_metabolism.phenylpropanoids.lignin_biosynthesis.CAD'
PGC4.4	snp_04_31067030	J	-0.35	3.07E-07	4	G	A	upstream_gene_variant	LOC_Os04g52280	-	'16.2.1.10'_'secondary_metabolism.phenylpropanoids.lignin_biosynthesis.CAD'
PGC4.4	snp_04_31067638	J	-0.33	1.79E-06	4	C	A	upstream_gene_variant	LOC_Os04g52280	-	'16.2.1.10'_'secondary_metabolism.phenylpropanoids.lignin_biosynthesis.CAD'
PGC4.4	snp_04_31068157	J	-0.35	3.43E-07	4	C	A	upstream_gene_variant	LOC_Os04g52280	-	'16.2.1.10'_'secondary_metabolism.phenylpropanoids.lignin_biosynthesis.CAD'
PGC4.4	snp_04_31068172	J	0.32	2.98E-06	4	C	G	upstream_gene_variant	LOC_Os04g52280	-	'16.2.1.10'_'secondary_metabolism.phenylpropanoids.lignin_biosynthesis.CAD'
PGC4.4	snp_04_31068235	J	-0.35	2.09E-07	4	C	T	upstream_gene_variant	LOC_Os04g52280	-	'16.2.1.10'_'secondary_metabolism.phenylpropanoids.lignin_biosynthesis.CAD'
PGC4.4	snp_04_31068245	J	-0.35	3.07E-07	4	G	A	upstream_gene_variant	LOC_Os04g52280	-	'16.2.1.10'_'secondary_metabolism.phenylpropanoids.lignin_biosynthesis.CAD'
PGC4.4	snp_04_31068491	J	0.32	1.92E-06	4	C	T	initiator_codon_variant&nor	LOC_Os04g52270	p.Met1?	'31.4'_'cell.vesicle_transport'
PGC4.4	snp_04_31069222	J	-0.56	7.25E-08	4	G	A	upstream_gene_variant	LOC_Os04g52270	-	'31.4'_'cell.vesicle_transport'
PGC4.4	snp_04_31069275	J	-0.33	1.86E-06	4	G	A	upstream_gene_variant	LOC_Os04g52270	-	'31.4'_'cell.vesicle_transport'
PGC4.4	snp_04_31070300	J	-0.32	2.24E-06	4	C	A	upstream_gene_variant	LOC_Os04g52270	-	'31.4'_'cell.vesicle_transport'
PGC4.4	snp_04_31070759	J	-0.34	6.87E-07	4	G	T	upstream_gene_variant	LOC_Os04g52270	-	'31.4'_'cell.vesicle_transport'
PGC4.4	snp_04_31080395	J	-0.35	3.24E-07	4	G	A	5_prime_UTR_premature_st	LOC_Os04g52310	-	'34.12'_'transport.metal'
PGC4.4	snp_04_31081582	J	-0.35	3.61E-07	4	C	A	upstream_gene_variant	LOC_Os04g52310	-	'34.12'_'transport.metal'
PGC4.4	snp_04_31081690	J	-0.35	2.80E-07	4	C	A	upstream_gene_variant	LOC_Os04g52310	-	'34.12'_'transport.metal'
PGC4.4	snp_04_31084483	J	-0.35	3.12E-07	4	G	A	upstream_gene_variant	LOC_Os04g52310	-	'34.12'_'transport.metal'
PGC4.4	snp_04_31084575	J	-0.33	1.86E-06	4	C	T	upstream_gene_variant	LOC_Os04g52310	-	'34.12'_'transport.metal'
PGC4.4	snp_04_31084713	J	-0.35	5.59E-07	4	C	T	upstream_gene_variant	LOC_Os04g52310	-	'34.12'_'transport.metal'
PGC4.4	snp_04_31084797	J	-0.34	4.44E-07	4	T	A	upstream_gene_variant	LOC_Os04g52310	-	'34.12'_'transport.metal'
PGC4.4	snp_04_31084861	J	-0.33	2.10E-06	4	C	T	upstream_gene_variant	LOC_Os04g52310	-	'34.12'_'transport.metal'
PGC4.4	snp_04_31085234	J	-0.34	4.48E-07	4	C	A	upstream_gene_variant	LOC_Os04g52310	-	'34.12'_'transport.metal'
PGC4.4	snp_04_31096564	J	-0.32	3.09E-06	4	T	C	downstream_gene_variant	LOC_Os04g52320	-	'35.2'_'not_assigned.unknown'
PGC4.4	snp_04_31212801	J	0.35	4.16E-07	4	G	A	synonymous_variant	LOC_Os04g52479	p.His233His	'35.2'_'not_assigned.unknown'
PGC5.1	snp_05_3874628	J	-0.96	8.42E-08	5	C	T	downstream_gene_variant	LOC_Os05g07300	-	'29.4'_'protein.postranslational_modification'
PGC5.1	snp_05_3893590	J	-0.96	9.06E-08	5	C	T	upstream_gene_variant	LOC_Os05g07330	-	'35.2'_'not_assigned.unknown'
PGC5.2	snp_05_5023459	I	0.28	1.28E-06	5	G	A	upstream_gene_variant	LOC_Os05g09040	-	'35.2'_'not_assigned.unknown'
PGC5.2	snp_05_5369111	I	-0.24	3.01E-06	5	T	A	downstream_gene_variant	LOC_Os05g09520	-	'30.3'_'signalling.calcium'
PGC5.2	snp_05_5369555	I	-0.24	2.74E-06	5	T	A	downstream_gene_variant	LOC_Os05g09520	-	'30.3'_'signalling.calcium'
PGC5.2	snp_05_5371529	I	0.32	3.15E-10	5	C	A	downstream_gene_variant	LOC_Os05g09520	-	'30.3'_'signalling.calcium'
PGC5.2	snp_05_5371609	I	0.32	1.89E-10	5	A	G	downstream_gene_variant	LOC_Os05g09520	-	'30.3'_'signalling.calcium'
PGC5.2	snp_05_5371716	I	0.33	7.01E-11	5	G	A	intergenic_region	LOC_Os05g09520-LOC_Os05g09530	-	NA
PGC5.2	snp_05_5371772	I	0.32	2.13E-10	5	G	A	intergenic_region	LOC_Os05g09520-LOC_Os05g09530	-	NA

QTL ID	SNP ID	Allele†	Beta	P-value	Chr	Ref	Alt	Region	Gene	Amino acid change	GoMAPMAN
PGC5.2	snp_05_5373841	I	0.33	1.25E-10	5	C	T	intergenic_region	LOC_Os05g09520-LOC_Os05g09530	-	NA
PGC5.2	snp_05_5376138	I	0.30	4.63E-09	5	G	A	intergenic_region	LOC_Os05g09520-LOC_Os05g09530	-	NA
PGC5.2	snp_05_5376161	I	0.28	3.89E-08	5	A	G	intergenic_region	LOC_Os05g09520-LOC_Os05g09530	-	NA
PGC5.2	snp_05_5377112	J	-0.29	1.39E-08	5	A	T	intergenic_region	LOC_Os05g09520-LOC_Os05g09530	-	NA
PGC5.2	snp_05_5377198	J	-0.30	3.33E-09	5	C	T	intergenic_region	LOC_Os05g09520-LOC_Os05g09530	-	NA
PGC5.2	snp_05_5378981	J	-0.30	1.14E-08	5	T	A	intergenic_region	LOC_Os05g09520-LOC_Os05g09530	-	NA
PGC5.2	snp_05_5436924	I	0.27	3.01E-06	5	T	C	upstream_gene_variant	LOC_Os05g09630	-	'35.2'_'not_assigned.unknown'
PGC5.2	snp_05_5966964	J	-0.90	5.04E-07	5	T	C	upstream_gene_variant	LOC_Os05g10790	-	'35.2'_'not_assigned.unknown'
PGC5.2	snp_05_5986536	J	-0.88	1.21E-06	5	G	A	missense_variant	LOC_Os05g10830	p.Gly131Arg	'35.2'_'not_assigned.unknown'
PGC5.3	snp_05_6146864	I	-0.30	1.52E-07	5	T	C	downstream_gene_variant	LOC_Os05g10990	-	'35.2'_'not_assigned.unknown'
PGC5.3	snp_05_6189898	J	-0.68	1.36E-06	5	G	A	upstream_gene_variant	LOC_Os05g11040	-	'35.2'_'not_assigned.unknown'
PGC5.3	snp_05_6194136	I	-0.29	2.27E-06	5	T	C	upstream_gene_variant	LOC_Os05g11040	-	'35.2'_'not_assigned.unknown'
PGC5.3	snp_05_6194270	I	-0.29	2.46E-06	5	G	A	upstream_gene_variant	LOC_Os05g11040	-	'35.2'_'not_assigned.unknown'
PGC5.3	snp_05_6628743	J	-1.07	1.43E-06	5	G	A	upstream_gene_variant	LOC_Os05g11680	-	'35.2'_'not_assigned.unknown'
PGC5.3	snp_05_6664004	J	-1.01	2.45E-06	5	A	T	upstream_gene_variant	LOC_Os05g11730	-	'29.4'_'protein.postranslational_modification'
PGC5.4	snp_05_13518841	J	-0.47	1.68E-06	5	A	T	downstream_gene_variant	LOC_Os05g23570	-	'35.2'_'not_assigned.unknown'
PGC5.5	snp_05_17201845	J	0.41	1.65E-06	5	C	T	upstream_gene_variant	LOC_Os05g29750	-	'35.2'_'not_assigned.unknown'
PGC5.5	snp_05_17203575	J	0.41	2.28E-06	5	C	T	downstream_gene_variant	LOC_Os05g29735	-	'35.2'_'not_assigned.unknown'
PGC5.5	snp_05_17204763	J	0.40	2.98E-06	5	G	C	upstream_gene_variant	LOC_Os05g29760	-	'19.20'_'tetrapyrrole_synthesis.ferrochelatase'
PGC5.5	snp_05_17205125	J	0.41	1.66E-06	5	C	T	upstream_gene_variant	LOC_Os05g29760	-	'19.20'_'tetrapyrrole_synthesis.ferrochelatase'
PGC5.5	snp_05_17205676	J	0.41	1.72E-06	5	T	C	upstream_gene_variant	LOC_Os05g29760	-	'19.20'_'tetrapyrrole_synthesis.ferrochelatase'
PGC5.5	snp_05_17207311	J	0.41	1.78E-06	5	C	T	upstream_gene_variant	LOC_Os05g29760	-	'19.20'_'tetrapyrrole_synthesis.ferrochelatase'
PGC5.5	snp_05_17845842	J	0.39	7.17E-07	5	C	T	upstream_gene_variant	LOC_Os05g30780	-	'26.8'_'misc.nitrilases,_*nitrile_lyases,_berberine_bridge_enzymes,_reticuline_oxidases,_troponine_reductases'
PGC5.6	snp_05_19611671	J	-1.07	1.47E-06	5	G	A	downstream_gene_variant	LOC_Os05g33380	-	'4.3.10'_'glycolysis.unclear/dually_targeted.aldolase'
PGC5.6	snp_05_19635339	J	-1.07	1.41E-06	5	A	T	missense_variant	LOC_Os05g33410	p.Asn280Ile	'29.5.4'_'protein.degradation.aspartate_protease'
PGC5.6	snp_05_19643800	J	-1.07	1.47E-06	5	C	T	upstream_gene_variant	LOC_Os05g33430	-	'29.5.4'_'protein.degradation.aspartate_protease'
PGC5.7	snp_05_21681407	J	-0.66	1.38E-06	5	A	G	intergenic_region	LOC_Os05g37090-LOC_Os05g37100	-	NA
PGC5.8	snp_05_29309709	J	0.40	1.77E-06	5	T	A	downstream_gene_variant	LOC_Os05g51080	-	'35.2'_'not_assigned.unknown'
PGC5.8	snp_05_29310238	J	0.39	2.42E-06	5	C	T	downstream_gene_variant	LOC_Os05g51080	-	'35.2'_'not_assigned.unknown'
PGC5.8	snp_05_29325121	J	-0.60	8.32E-07	5	A	C	upstream_gene_variant	LOC_Os05g51110	-	'35.1.25'_'not_assigned.no_ontology.paired_amphipathic_helix_repeat-containing_protein'
PGC5.8	snp_05_29337175	J	0.43	3.43E-07	5	C	T	synonymous_variant	LOC_Os05g51140	p.Phe91Phe	'35.2'_'not_assigned.unknown'
PGC5.8	snp_05_29341831	J	0.41	2.67E-06	5	T	C	synonymous_variant	LOC_Os05g51150	p.Pro58Pro	'27.3.85'_'RNA.regulation_of_transcription.sigma_like_plant'

QTL ID	SNP ID	Allele†	Beta	P-value	Chr	Ref	Alt	Region	Gene	Amino acid change	GoMAPMAN
PGC6.1	snp_06_1225608	J	-0.69	3.86E-11	6	A	G	downstream_gene_variant	LOC_Os06g03230	-	'35.2'_'not_assigned.unknown'
PGC6.1	snp_06_1227791	J	-0.38	3.78E-07	6	A	G	upstream_gene_variant	LOC_Os06g03250	-	'35.2'_'not_assigned.unknown'
PGC6.1	snp_06_1245358	J	-0.64	1.92E-11	6	C	T	synonymous_variant	LOC_Os06g03260	p.Thr113Thr	'35.2'_'not_assigned.unknown'
PGC6.1	snp_06_1246170	J	-0.36	1.58E-06	6	T	A	upstream_gene_variant	LOC_Os06g03280	-	'35.2'_'not_assigned.unknown'
PGC6.1	snp_06_1257803	J	-0.67	2.00E-09	6	A	G	missense_variant	LOC_Os06g03300	p.Thr194Ala	'35.2'_'not_assigned.unknown'
PGC6.1	snp_06_1258271	J	-0.37	8.42E-07	6	G	A	synonymous_variant	LOC_Os06g03310	p.Asp1263Asp	'35.2'_'not_assigned.unknown'
PGC6.1	snp_06_1258527	J	-0.64	1.96E-11	6	A	T	missense_variant	LOC_Os06g03310	p.Leu1178His	'35.2'_'not_assigned.unknown'
PGC6.1	snp_06_1259222	J	-0.62	5.06E-10	6	G	A	upstream_gene_variant	LOC_Os06g03290	-	'35.2'_'not_assigned.unknown'
PGC6.1	snp_06_1259614	J	-0.65	4.78E-09	6	A	T	missense_variant	LOC_Os06g03310	p.Ile952Asn	'35.2'_'not_assigned.unknown'
PGC6.1	snp_06_1259666	J	-0.66	4.32E-09	6	A	G	missense_variant	LOC_Os06g03310	p.Trp935Arg	'35.2'_'not_assigned.unknown'
PGC6.1	snp_06_1259835	J	-0.64	4.14E-11	6	C	T	synonymous_variant	LOC_Os06g03310	p.Val878Val	'35.2'_'not_assigned.unknown'
PGC6.1	snp_06_1260027	J	-0.68	8.17E-11	6	G	A	synonymous_variant	LOC_Os06g03310	p.Asp814Asp	'35.2'_'not_assigned.unknown'
PGC6.1	snp_06_1261092	J	-0.64	2.01E-11	6	C	T	synonymous_variant	LOC_Os06g03310	p.Ala525Ala	'35.2'_'not_assigned.unknown'
PGC6.1	snp_06_1261098	J	-0.64	1.96E-11	6	T	A	synonymous_variant	LOC_Os06g03310	p.Leu523Leu	'35.2'_'not_assigned.unknown'
PGC6.1	snp_06_1261197	J	-0.64	2.13E-11	6	C	A	synonymous_variant	LOC_Os06g03310	p.Ala490Ala	'35.2'_'not_assigned.unknown'
PGC6.1	snp_06_1261311	J	-0.36	8.60E-07	6	C	T	synonymous_variant	LOC_Os06g03310	p.Thr452Thr	'35.2'_'not_assigned.unknown'
PGC6.1	snp_06_1261790	J	-0.64	1.97E-11	6	C	T	missense_variant	LOC_Os06g03310	p.Glu293Lys	'35.2'_'not_assigned.unknown'
PGC6.1	snp_06_1261827	J	-0.64	1.97E-11	6	C	T	synonymous_variant	LOC_Os06g03310	p.Leu280Leu	'35.2'_'not_assigned.unknown'
PGC6.1	snp_06_1262004	J	-0.51	1.11E-09	6	G	A	synonymous_variant	LOC_Os06g03310	p.Leu221Leu	'35.2'_'not_assigned.unknown'
PGC6.1	snp_06_1262389	J	-0.61	2.24E-06	6	G	T	missense_variant	LOC_Os06g03310	p.Pro93His	'35.2'_'not_assigned.unknown'
PGC6.1	snp_06_1266484	J	-0.64	1.84E-11	6	G	A	missense_variant	LOC_Os06g03320	p.Arg807Cys	'35.2'_'not_assigned.unknown'
PGC6.1	snp_06_1267111	J	-0.64	1.94E-11	6	C	T	splice_region_variant&intron	LOC_Os06g03320	-	'35.2'_'not_assigned.unknown'
PGC6.1	snp_06_1267143	J	-0.64	4.03E-11	6	G	A	missense_variant	LOC_Os06g03320	p.Ser610Phe	'35.2'_'not_assigned.unknown'
PGC6.1	snp_06_1268126	J	-0.66	1.12E-11	6	G	A	synonymous_variant	LOC_Os06g03320	p.Leu318Leu	'35.2'_'not_assigned.unknown'
PGC6.1	snp_06_1268234	J	-0.64	1.96E-11	6	C	T	missense_variant	LOC_Os06g03320	p.Gly282Ser	'35.2'_'not_assigned.unknown'
PGC6.1	snp_06_1269073	J	-0.48	7.90E-07	6	G	A	missense_variant	LOC_Os06g03320	p.Ser2Leu	'35.2'_'not_assigned.unknown'
PGC6.1	snp_06_1269151	J	-0.56	2.48E-10	6	G	A	upstream_gene_variant	LOC_Os06g03320	-	'35.2'_'not_assigned.unknown'
PGC6.1	snp_06_1288331	J	-0.67	7.49E-12	6	C	T	upstream_gene_variant	LOC_Os06g03370	-	'35.2'_'not_assigned.unknown'
PGC6.1	snp_06_1288866	J	-0.64	1.96E-11	6	A	T	upstream_gene_variant	LOC_Os06g03370	-	'35.2'_'not_assigned.unknown'
PGC6.1	snp_06_1289431	J	-0.64	1.96E-11	6	T	A	stop_gained	LOC_Os06g03380	p.Leu109*	'35.2'_'not_assigned.unknown'
PGC6.1	snp_06_1291182	J	-0.64	1.97E-11	6	A	C	missense_variant	LOC_Os06g03380	p.Lys693Gln	'35.2'_'not_assigned.unknown'
PGC6.1	snp_06_1347924	I	-0.35	2.17E-08	6	A	G	missense_variant	LOC_Os06g03500	p.Tyr794His	'20.1'_'stress.biotic'
PGC6.1	snp_06_1357299	J	-0.66	9.83E-12	6	G	T	upstream_gene_variant	LOC_Os06g03520	-	'35.2'_'not_assigned.unknown'
PGC6.1	snp_06_1358589	J	-0.64	4.40E-11	6	C	T	upstream_gene_variant	LOC_Os06g03520	-	'35.2'_'not_assigned.unknown'
PGC6.1	snp_06_1359124	J	-0.63	7.55E-11	6	C	T	upstream_gene_variant	LOC_Os06g03520	-	'35.2'_'not_assigned.unknown'
PGC6.1	snp_06_1360040	J	-0.48	5.63E-09	6	G	A	upstream_gene_variant	LOC_Os06g03520	-	'35.2'_'not_assigned.unknown'
PGC6.1	snp_06_1360808	J	-0.30	7.24E-07	6	A	G	upstream_gene_variant	LOC_Os06g03520	-	'35.2'_'not_assigned.unknown'
PGC6.1	snp_06_1367726	J	-0.47	1.92E-06	6	A	T	upstream_gene_variant	LOC_Os06g03550	-	'35.2'_'not_assigned.unknown'
PGC6.1	snp_06_1404196	J	-0.54	1.29E-06	6	T	A	upstream_gene_variant	LOC_Os06g03610	-	'30.2.16'_'signalling.receptor_kinases.Catharanthus_roseus-like_RLK1'
PGC6.1	snp_06_1432931	J	-0.48	6.52E-07	6	A	G	upstream_gene_variant	LOC_Os06g03670	-	'27.3.3'_'RNA.regulation_of_transcription.AP2/EREBP,_APETALA2/Ethylene-responsive_element_binding_protein_family'

QTL ID	SNP ID	Allele†	Beta	P-value	Chr	Ref	Alt	Region	Gene	Amino acid change	GoMAPMAN
PGC6.1	snp_06_1824110	J	-1.08	8.13E-16	6	C	T	intron_variant	LOC_Os06g04300	-	'33.99'_'development.unspecified'
PGC6.1	snp_06_1874664	J	-0.79	5.17E-09	6	A	T	downstream_gene_variant	LOC_Os06g04390	-	'35.1.40'_'not_assigned.no_ontology.glycine_rich_proteins'
PGC6.2	snp_06_2032400	J	-0.67	5.60E-07	6	G	A	upstream_gene_variant	LOC_Os06g04650	-	'29.4'_'protein.postranslational_modification'
PGC6.2	snp_06_2113328	J	-0.84	9.73E-07	6	G	A	synonymous_variant	LOC_Os06g04840	p.Pro701Pro	'20.1.7'_'stress.biotic.PR-proteins'
PGC6.2	snp_06_2232920	J	-0.95	6.30E-10	6	G	A	missense_variant	LOC_Os06g05050	p.Ala730Thr	'30.2.25'_'signalling.receptor_kinases.wall_associated_kinase'
PGC6.3	snp_06_4005919	J	-0.74	2.51E-07	6	C	T	upstream_gene_variant	LOC_Os06g08270	-	'35.2'_'not_assigned.unknown'
PGC6.3	snp_06_4647272	J	-0.82	3.08E-06	6	G	A	upstream_gene_variant	LOC_Os06g09230	-	'29.4.1.59'_'protein.postranslational_modification.kinase.receptor_like_cytoplasmatic_kinase_IX'
NA	snp_06_10554929	J	-0.74	2.34E-07	6	G	C	3_prime_UTR_variant	LOC_Os06g18140	-	'26.2'_'misc.UDP_glucosyl_and_glucoronyl_transferases'
NA	snp_06_10583801	J	-0.74	2.61E-07	6	C	G	downstream_gene_variant	LOC_Os06g18164	-	'35.2'_'not_assigned.unknown'
PGC6.4	snp_06_17782023	J	-0.95	3.32E-07	6	G	A	downstream_gene_variant	LOC_Os06g30680	-	'35.1'_'not_assigned.no_ontology'
PGC6.4	snp_06_17782039	J	-0.97	1.39E-06	6	A	T	downstream_gene_variant	LOC_Os06g30680	-	'35.1'_'not_assigned.no_ontology'
PGC6.5	snp_06_20015162	J	-0.78	1.10E-06	6	C	T	upstream_gene_variant	LOC_Os06g34400	-	'35.2'_'not_assigned.unknown'
PGC6.5	snp_06_20406028	J	-0.68	2.26E-06	6	T	A	upstream_gene_variant	LOC_Os06g35060	-	'35.2'_'not_assigned.unknown'
PGC6.6	snp_06_22794466	J	-0.64	7.33E-07	6	C	T	3_prime_UTR_variant	LOC_Os06g38470	-	'27.3.55'_'RNA.regulation_of_transcription.HDA'
PGC6.6	snp_06_22794519	J	-0.64	7.86E-07	6	T	C	3_prime_UTR_variant	LOC_Os06g38470	-	'27.3.55'_'RNA.regulation_of_transcription.HDA'
PGC6.6	snp_06_22794717	J	-0.64	7.75E-07	6	A	G	3_prime_UTR_variant	LOC_Os06g38470	-	'27.3.55'_'RNA.regulation_of_transcription.HDA'
PGC6.6	snp_06_22795136	J	-0.63	1.49E-06	6	C	T	missense_variant	LOC_Os06g38470	p.Ala462Thr	'27.3.55'_'RNA.regulation_of_transcription.HDA'
PGC6.6	snp_06_22795411	J	-0.63	8.46E-07	6	C	A	upstream_gene_variant	LOC_Os06g38460	-	'35.2'_'not_assigned.unknown'
PGC6.6	snp_06_22795905	J	-0.64	7.75E-07	6	C	T	upstream_gene_variant	LOC_Os06g38460	-	'35.2'_'not_assigned.unknown'
PGC6.6	snp_06_22796392	J	-0.64	6.99E-07	6	G	T	upstream_gene_variant	LOC_Os06g38460	-	'35.2'_'not_assigned.unknown'
PGC6.6	snp_06_22797322	J	-0.64	7.75E-07	6	T	G	3_prime_UTR_variant	LOC_Os06g38470	-	'27.3.55'_'RNA.regulation_of_transcription.HDA'
PGC6.6	snp_06_22797853	J	-0.63	8.64E-07	6	C	T	intron_variant	LOC_Os06g38470	-	'27.3.55'_'RNA.regulation_of_transcription.HDA'
PGC6.6	snp_06_22797972	J	-0.64	7.60E-07	6	C	T	intron_variant	LOC_Os06g38470	-	'27.3.55'_'RNA.regulation_of_transcription.HDA'
PGC6.6	snp_06_22798201	J	-0.64	7.83E-07	6	A	C	intron_variant	LOC_Os06g38470	-	'27.3.55'_'RNA.regulation_of_transcription.HDA'
PGC6.6	snp_06_22798704	J	-0.64	8.08E-07	6	A	G	intron_variant	LOC_Os06g38470	-	'27.3.55'_'RNA.regulation_of_transcription.HDA'
PGC6.6	snp_06_22798923	J	-0.64	7.27E-07	6	C	T	intron_variant	LOC_Os06g38470	-	'27.3.55'_'RNA.regulation_of_transcription.HDA'
PGC6.6	snp_06_22799472	J	-0.64	7.75E-07	6	G	A	synonymous_variant	LOC_Os06g38470	p.Ile189Ile	'27.3.55'_'RNA.regulation_of_transcription.HDA'
PGC6.6	snp_06_22803814	J	-0.63	8.85E-07	6	C	G	upstream_gene_variant	LOC_Os06g38470	-	'27.3.55'_'RNA.regulation_of_transcription.HDA'
PGC6.6	snp_06_22810221	J	-0.62	7.96E-07	6	G	T	missense_variant	LOC_Os06g38490	p.Gly73Cys	'30.2.12'_'signalling.receptor_kinases.leucine_rich_repeat_XII'
PGC6.6	snp_06_22812080	J	-0.58	2.22E-06	6	C	G	missense_variant	LOC_Os06g38490	p.Ile619Met	'30.2.12'_'signalling.receptor_kinases.leucine_rich_repeat_XII'
PGC6.6	snp_06_23104134	J	-0.81	1.29E-06	6	C	A	upstream_gene_variant	LOC_Os06g38940	-	'29.5.11.4.2'_'protein.degradation.ubiquitin.E3.RING'
NA	snp_06_23501344	J	-0.82	2.41E-06	6	G	A	upstream_gene_variant	LOC_Os06g39590	-	'27.3.40'_'RNA.regulation_of_transcription.Aux/IAA_family'
PGC6.7	snp_06_29158719	J	-0.76	1.13E-06	6	T	C	upstream_gene_variant	LOC_Os06g48200	-	'10.7'_'cell_wall.modification'
PGC6.7	snp_06_29159100	J	-0.87	3.34E-07	6	A	G	upstream_gene_variant	LOC_Os06g48200	-	'10.7'_'cell_wall.modification'
PGC6.7	snp_06_29160016	J	-0.76	1.12E-06	6	G	A	upstream_gene_variant	LOC_Os06g48200	-	'10.7'_'cell_wall.modification'
PGC6.7	snp_06_29160457	J	-0.76	1.16E-06	6	C	A	upstream_gene_variant	LOC_Os06g48200	-	'10.7'_'cell_wall.modification'

QTL ID	SNP ID	Allele†	Beta	P-value	Chr	Ref	Alt	Region	Gene	Amino acid change	GoMAPMAN
PGC6.7	snp_06_29160497	J	-0.81	3.56E-08	6	T	C	upstream_gene_variant	LOC_Os06g48200	-	'10.7'_'cell_wall.modification'
PGC6.7	snp_06_29168610	J	-0.87	3.49E-07	6	G	T	upstream_gene_variant	LOC_Os06g48220	-	'35.2'_'not_assigned.unknown'
PGC6.7	snp_06_29183343	J	-0.96	7.94E-08	6	C	T	upstream_gene_variant	LOC_Os06g48260	-	'35.2'_'not_assigned.unknown'
PGC6.7	snp_06_29692340	J	-0.82	4.20E-07	6	A	G	upstream_gene_variant	LOC_Os06g48990	-	'20.1'_'stress.biotic'
PGC6.7	snp_06_29692358	J	-0.82	4.20E-07	6	C	T	upstream_gene_variant	LOC_Os06g48990	-	'20.1'_'stress.biotic'
PGC6.7	snp_06_29692444	J	-0.82	4.20E-07	6	C	T	upstream_gene_variant	LOC_Os06g48990	-	'20.1'_'stress.biotic'
PGC6.7	snp_06_29692543	J	-0.85	3.84E-07	6	G	A	upstream_gene_variant	LOC_Os06g48990	-	'20.1'_'stress.biotic'
PGC6.7	snp_06_29692981	J	-0.82	4.39E-07	6	T	A	upstream_gene_variant	LOC_Os06g48990	-	'20.1'_'stress.biotic'
PGC6.7	snp_06_29693321	J	-0.92	7.21E-08	6	T	G	upstream_gene_variant	LOC_Os06g48990	-	'20.1'_'stress.biotic'
PGC6.7	snp_06_29694566	J	-0.71	8.02E-07	6	G	A	upstream_gene_variant	LOC_Os06g48990	-	'20.1'_'stress.biotic'
PGC6.7	snp_06_29694673	J	-0.71	7.51E-07	6	G	C	upstream_gene_variant	LOC_Os06g48990	-	'20.1'_'stress.biotic'
PGC6.7	snp_06_29694840	J	-0.71	7.75E-07	6	C	T	upstream_gene_variant	LOC_Os06g48990	-	'20.1'_'stress.biotic'
PGC6.7	snp_06_29694892	J	-0.71	7.76E-07	6	G	C	upstream_gene_variant	LOC_Os06g48990	-	'20.1'_'stress.biotic'
PGC6.7	snp_06_29695092	J	-0.71	7.76E-07	6	A	G	upstream_gene_variant	LOC_Os06g48990	-	'20.1'_'stress.biotic'
PGC6.7	snp_06_29695098	J	-0.72	7.43E-07	6	A	G	upstream_gene_variant	LOC_Os06g48990	-	'20.1'_'stress.biotic'
PGC6.7	snp_06_29695113	J	-0.71	8.76E-07	6	G	T	upstream_gene_variant	LOC_Os06g49000	-	'35.2'_'not_assigned.unknown'
PGC6.7	snp_06_29695945	J	-0.71	7.73E-07	6	C	A	upstream_gene_variant	LOC_Os06g49000	-	'35.2'_'not_assigned.unknown'
PGC6.7	snp_06_29696104	J	-0.70	2.21E-06	6	C	T	upstream_gene_variant	LOC_Os06g49000	-	'35.2'_'not_assigned.unknown'
PGC6.7	snp_06_29696741	J	-0.71	7.76E-07	6	G	C	missense_variant	LOC_Os06g49000	p.Val2Leu	'35.2'_'not_assigned.unknown'
PGC6.7	snp_06_29704953	J	-0.63	9.20E-07	6	G	A	downstream_gene_variant	LOC_Os06g49000	-	'35.2'_'not_assigned.unknown'
PGC6.7	snp_06_29705182	J	-0.63	9.75E-07	6	G	A	downstream_gene_variant	LOC_Os06g49000	-	'35.2'_'not_assigned.unknown'
PGC6.7	snp_06_29705842	J	-0.63	9.44E-07	6	C	T	downstream_gene_variant	LOC_Os06g49000	-	'35.2'_'not_assigned.unknown'
PGC6.7	snp_06_29706892	J	-0.63	9.26E-07	6	C	T	missense_variant	LOC_Os06g49010	p.Gly57Ser	'33.3'_'development.squamosa_promoter_binding_like_(SPL)'
PGC6.7	snp_06_29708892	J	-0.63	9.44E-07	6	G	T	upstream_gene_variant	LOC_Os06g49010	-	'33.3'_'development.squamosa_promoter_binding_like_(SPL)'
PGC6.8	snp_06_30184764	J	-0.65	5.44E-07	6	G	A	upstream_gene_variant	LOC_Os06g49850	-	'35.2'_'not_assigned.unknown'
PGC6.8	snp_06_30325939	J	-0.64	1.10E-06	6	G	A	intergenic_region	LOC_Os06g50040-LOC_Os06g50050	-	NA
PGC6.8	snp_06_30334155	J	0.47	1.12E-06	6	T	G	downstream_gene_variant	LOC_Os06g50060	-	'30.5'_'signalling.G-proteins'
PGC6.8	snp_06_30371937	J	0.54	3.96E-08	6	G	A	synonymous_variant	LOC_Os06g50154	p.Met31Met	'35.1'_'not_assigned.no_ontology'
PGC6.8	snp_06_30378142	J	-0.73	1.24E-08	6	C	A	downstream_gene_variant	LOC_Os06g50154	-	'35.1'_'not_assigned.no_ontology'
PGC6.8	snp_06_30404780	J	-0.74	8.30E-08	6	C	T	upstream_gene_variant	LOC_Os06g50200	-	'28.99'_'DNA.unspecified'
PGC6.8	snp_06_30405618	J	-0.68	7.39E-07	6	C	A	upstream_gene_variant	LOC_Os06g50210	-	'35.2'_'not_assigned.unknown'
PGC6.8	snp_06_30436072	J	-0.41	2.24E-06	6	A	T	upstream_gene_variant	LOC_Os06g50270	-	'35.2'_'not_assigned.unknown'
PGC6.8	snp_06_30436367	J	-0.43	1.98E-06	6	C	T	upstream_gene_variant	LOC_Os06g50270	-	'35.2'_'not_assigned.unknown'
PGC6.8	snp_06_30451292	J	-0.39	2.53E-06	6	G	A	upstream_gene_variant	LOC_Os06g50300	-	'20.2.1'_'stress.abiotic.heat'
PGC6.8	snp_06_30468379	J	-0.73	1.19E-08	6	A	G	upstream_gene_variant	LOC_Os06g50330	-	'33.99'_'development.unspecified'
PGC6.8	snp_06_30469303	J	-0.41	3.08E-06	6	C	T	upstream_gene_variant	LOC_Os06g50330	-	'33.99'_'development.unspecified'
PGC6.8	snp_06_30473682	J	-0.41	2.99E-06	6	G	A	missense_variant	LOC_Os06g50340	p.Pro260Ser	'30.2.11'_'signalling.receptor_kinases.leucine_rich_repeat_XI'
PGC6.8	snp_06_30487804	J	-0.45	4.79E-07	6	C	T	upstream_gene_variant	LOC_Os06g50360	-	'29.1.30'_'protein.aa_activation.pseudouridylate_synthase'

QTL ID	SNP ID	Allele†	Beta	P-value	Chr	Ref	Alt	Region	Gene	Amino acid change	GoMAPMAN
PGC6.8	snp_06_30488787	J	-0.44	1.05E-06	6	C	T	upstream_gene_variant	LOC_Os06g50360	-	'29.1.30'_'protein.aa_activation.pseudouridylate_synthase'
PGC6.8	snp_06_30490737	J	-0.43	2.27E-06	6	A	G	upstream_gene_variant	LOC_Os06g50370	-	'29.5.11.4.2'_'protein.degradation.ubiquitin.E3.RING'
PGC6.8	snp_06_30521543	J	-0.74	8.30E-08	6	C	A	downstream_gene_variant	LOC_Os06g50400	-	'10.7'_'cell_wall.modification'
PGC6.8	snp_06_30791087	J	0.30	5.13E-07	6	C	T	intergenic_region	LOC_Os06g50890-LOC_Os06g50910	-	NA
PGC6.8	snp_06_30817976	J	0.44	1.88E-06	6	C	A	upstream_gene_variant	LOC_Os06g50910	-	'31.3'_'cell.cycle'
PGC6.8	snp_06_30826213	J	-0.45	4.59E-07	6	G	A	upstream_gene_variant	LOC_Os06g50930	-	'33.99'_'development.unspecified'
PGC6.8	snp_06_30826231	J	-0.44	4.13E-07	6	A	G	upstream_gene_variant	LOC_Os06g50930	-	'33.99'_'development.unspecified'
PGC6.8	snp_06_30826303	J	-0.46	1.35E-07	6	A	G	upstream_gene_variant	LOC_Os06g50930	-	'33.99'_'development.unspecified'
PGC6.8	snp_06_30974876	J	-0.47	2.44E-06	6	C	T	upstream_gene_variant	LOC_Os06g51200	-	'35.2'_'not_assigned.unknown'
PGC7.1	snp_07_121611	J	-1.08	1.48E-06	7	C	T	upstream_gene_variant	LOC_Os07g01200	-	'11.1.1'_'lipid_metabolism.FA_synthesis_and_FA_elongation.Acetyl_CoA_Carboxylation'
PGC7.2	snp_07_1957012	J	-0.58	4.97E-07	7	T	C	upstream_gene_variant	LOC_Os07g04400	-	'35.2'_'not_assigned.unknown'
PGC7.3	snp_07_2680353	J	0.36	6.80E-07	7	A	C	downstream_gene_variant	LOC_Os07g05630	-	'35.2'_'not_assigned.unknown'
PGC7.3	snp_07_2729357	J	0.33	2.15E-06	7	G	A	intron_variant	LOC_Os07g05710	-	'35.2'_'not_assigned.unknown'
PGC7.3	snp_07_2732891	J	0.34	5.77E-07	7	A	T	upstream_gene_variant	LOC_Os07g05710	-	'35.2'_'not_assigned.unknown'
PGC7.4	snp_07_4516513	J	-0.81	1.24E-06	7	A	C	upstream_gene_variant	LOC_Os07g08770	-	'33.99'_'development.unspecified'
PGC7.5	snp_07_7622206	J	-0.85	9.86E-07	7	A	T	upstream_gene_variant	LOC_Os07g13270	-	'27.3.71'_'RNA.regulation_of_transcription.SNF7'
PGC7.6	snp_07_17273541	J	0.48	1.88E-06	7	C	T	missense_variant	LOC_Os07g29420	p.Val3Met	'27.2'_'RNA.transcription'
PGC7.6	snp_07_17277936	J	0.48	2.02E-06	7	A	C	upstream_gene_variant	LOC_Os07g29420	-	'27.2'_'RNA.transcription'
PGC7.7	snp_07_19793873	J	0.43	4.03E-07	7	G	A	upstream_gene_variant	LOC_Os07g33150	-	'35.2'_'not_assigned.unknown'
PGC7.7	snp_07_19910304	J	0.41	1.47E-06	7	C	T	upstream_gene_variant	LOC_Os07g33300	-	'35.2'_'not_assigned.unknown'
PGC7.8	snp_07_22404589	J	0.40	2.41E-06	7	C	T	synonymous_variant	LOC_Os07g37385	p.Pro207Pro	'35.2'_'not_assigned.unknown'
PGC7.8	snp_07_22404606	J	0.39	3.06E-06	7	G	A	synonymous_variant	LOC_Os07g37385	p.Ser202Ser	'35.2'_'not_assigned.unknown'
PGC7.8	snp_07_22409773	J	0.44	1.65E-07	7	G	A	upstream_gene_variant	LOC_Os07g37385	-	'35.2'_'not_assigned.unknown'
PGC7.8	snp_07_22422873	J	0.40	1.48E-06	7	C	T	upstream_gene_variant	LOC_Os07g37440	-	'35.2'_'not_assigned.unknown'
PGC7.8	snp_07_22425952	J	0.39	2.91E-06	7	C	T	upstream_gene_variant	LOC_Os07g37454	-	'34.99'_'transport.misc'
PGC7.8	snp_07_22426022	J	0.39	2.57E-06	7	C	G	upstream_gene_variant	LOC_Os07g37454	-	'34.99'_'transport.misc'
PGC7.8	snp_07_22442623	J	-0.32	7.03E-07	7	G	A	missense_variant	LOC_Os07g37480	p.Arg229Trp	'35.2'_'not_assigned.unknown'
PGC7.8	snp_07_22442651	J	-0.29	2.86E-06	7	G	A	synonymous_variant	LOC_Os07g37480	p.Thr219Thr	'35.2'_'not_assigned.unknown'
PGC7.8	snp_07_22442767	J	-0.34	1.84E-07	7	G	T	missense_variant	LOC_Os07g37480	p.Pro181Thr	'35.2'_'not_assigned.unknown'
PGC7.8	snp_07_22442796	J	-0.32	8.62E-07	7	C	T	missense_variant	LOC_Os07g37480	p.Arg171Gln	'35.2'_'not_assigned.unknown'
PGC7.8	snp_07_22443025	J	-0.32	6.96E-07	7	T	C	missense_variant	LOC_Os07g37480	p.Ile95Val	'35.2'_'not_assigned.unknown'
PGC7.8	snp_07_22443033	J	0.40	1.01E-06	7	G	A	synonymous_variant	LOC_Os07g37480	p.Leu92Leu	'35.2'_'not_assigned.unknown'
PGC7.8	snp_07_22443198	J	0.42	9.91E-07	7	G	A	synonymous_variant	LOC_Os07g37480	p.Ile37Ile	'35.2'_'not_assigned.unknown'
PGC7.8	snp_07_22443235	J	-0.40	3.76E-08	7	G	A	missense_variant	LOC_Os07g37480	p.Pro25Ser	'35.2'_'not_assigned.unknown'
PGC7.8	snp_07_22443316	J	0.42	9.91E-07	7	G	A	5_prime_UTR_variant	LOC_Os07g37480	-	'35.2'_'not_assigned.unknown'
PGC7.8	snp_07_22443424	J	0.41	1.34E-06	7	C	T	5_prime_UTR_variant	LOC_Os07g37480	-	'35.2'_'not_assigned.unknown'
PGC7.8	snp_07_22443624	J	-0.33	4.23E-07	7	T	C	5_prime_UTR_premature_st	LOC_Os07g37480	-	'35.2'_'not_assigned.unknown'
PGC7.8	snp_07_22444848	J	0.40	2.46E-06	7	G	T	upstream_gene_variant	LOC_Os07g37480	-	'35.2'_'not_assigned.unknown'
PGC7.8	snp_07_22457720	J	0.42	1.04E-06	7	C	A	synonymous_variant	LOC_Os07g37510	p.Asn270Asn	'34.99'_'transport.misc'
PGC7.8	snp_07_22478231	J	0.40	2.07E-06	7	G	T	downstream_gene_variant	LOC_Os07g37530	-	'34.99'_'transport.misc'

QTL ID	SNP ID	Allele†	Beta	P-value	Chr	Ref	Alt	Region	Gene	Amino acid change	GoMAPMAN
PGC7.8	snp_07_22483669	J	0.40	2.85E-06	7	C	T	upstream_gene_variant	LOC_Os07g37540	-	'35.2'_'not_assigned.unknown'
PGC7.8	snp_07_22483789	J	0.41	8.18E-07	7	C	T	upstream_gene_variant	LOC_Os07g37540	-	'35.2'_'not_assigned.unknown'
PGC7.8	snp_07_22484898	J	-0.32	4.77E-07	7	G	A	upstream_gene_variant	LOC_Os07g37540	-	'35.2'_'not_assigned.unknown'
PGC7.8	snp_07_22485648	J	0.42	8.31E-07	7	C	T	upstream_gene_variant	LOC_Os07g37540	-	'35.2'_'not_assigned.unknown'
PGC7.8	snp_07_22486262	J	-0.32	1.78E-06	7	G	A	synonymous_variant	LOC_Os07g37540	p.Ser44Ser	'35.2'_'not_assigned.unknown'
PGC7.8	snp_07_22486583	J	-0.33	3.10E-07	7	G	A	upstream_gene_variant	LOC_Os07g37550	-	'1.1.1.1'_'PS.lightreaction.photosystem_II.LHC-II'
PGC7.8	snp_07_22492865	J	0.42	8.75E-07	7	T	G	downstream_gene_variant	LOC_Os07g37550	-	'1.1.1.1'_'PS.lightreaction.photosystem_II.LHC-II'
PGC7.8	snp_07_22500080	J	-0.32	1.47E-06	7	A	T	upstream_gene_variant	LOC_Os07g37560	-	'31.1.1.3.8'_'cell.organisation.cytoskeleton.Myosin.Class_VII'
PGC7.8	snp_07_22501571	J	0.42	9.81E-07	7	G	A	upstream_gene_variant	LOC_Os07g37560	-	'31.1.1.3.8'_'cell.organisation.cytoskeleton.Myosin.Class_VII'
PGC7.8	snp_07_22502269	J	-0.32	1.29E-06	7	G	C	upstream_gene_variant	LOC_Os07g37560	-	'31.1.1.3.8'_'cell.organisation.cytoskeleton.Myosin.Class_VII'
PGC7.8	snp_07_22502462	J	-0.33	7.07E-07	7	A	G	upstream_gene_variant	LOC_Os07g37560	-	'31.1.1.3.8'_'cell.organisation.cytoskeleton.Myosin.Class_VII'
PGC8.1	snp_08_3648813	J	0.40	2.40E-06	8	A	G	intergenic_region	LOC_Os08g06474-LOC_Os08g06478	-	NA
PGC8.2	snp_08_4173699	J	0.35	1.76E-07	8	G	A	splice_region_variant&intron	LOC_Os08g07430	-	'35.2'_'not_assigned.unknown'
PGC8.3	snp_08_6071731	J	-0.47	2.10E-07	8	C	T	missense_variant	LOC_Os08g10360	p.Pro62Ser	'35.2'_'not_assigned.unknown'
PGC8.4	snp_08_24527577	J	0.42	1.32E-06	8	C	T	upstream_gene_variant	LOC_Os08g38790	-	'35.2'_'not_assigned.unknown'
PGC8.5	snp_08_26380470	J	-0.92	2.83E-06	8	G	A	missense_variant	LOC_Os08g41780	p.Arg283Cys	'11.9.2.1'_'lipid_metabolism.lipid_degradation.lipases.triacylglycerol_lipase'
PGC8.6	snp_08_27238987	J	-1.00	7.30E-07	8	G	C	intron_variant	LOC_Os08g43090	-	'27.3.35'_'RNA.regulation_of_transcription.bZIP_transcription_factor_family'
PGC8.7	snp_08_28221949	J	-0.65	1.66E-07	8	C	T	upstream_gene_variant	LOC_Os08g44950	-	'35.2'_'not_assigned.unknown'
PGC8.7	snp_08_28260958	J	-0.65	1.66E-07	8	G	A	upstream_gene_variant	LOC_Os08g45030	-	'34.16'_'transport.ABC_transporters_and_multidrug_resistance_systems'
PGC9.1	snp_09_3432724	J	-0.82	2.98E-06	9	G	A	downstream_gene_variant	LOC_Os09g07070	-	'35.2'_'not_assigned.unknown'
PGC9.2	snp_09_6549110	J	-0.50	4.18E-07	9	C	T	synonymous_variant	LOC_Os09g11730	p.Ala2Ala	'35.2'_'not_assigned.unknown'
PGC9.2	snp_09_6686339	J	-0.71	3.44E-07	9	A	T	downstream_gene_variant	LOC_Os09g11900	-	'35.2'_'not_assigned.unknown'
PGC9.2	snp_09_6723543	J	-0.66	6.30E-07	9	C	T	synonymous_variant	LOC_Os09g11940	p.Phe593Phe	'35.2'_'not_assigned.unknown'
PGC9.2	snp_09_6733776	J	-0.65	1.39E-06	9	G	A	upstream_gene_variant	LOC_Os09g11950	-	'35.2'_'not_assigned.unknown'
PGC9.2	snp_09_6894700	J	-0.58	5.01E-07	9	T	C	upstream_gene_variant	LOC_Os09g12190	-	'35.2'_'not_assigned.unknown'
PGC9.2	snp_09_6895540	J	-0.62	5.91E-07	9	G	A	upstream_gene_variant	LOC_Os09g12190	-	'35.2'_'not_assigned.unknown'
PGC9.2	snp_09_6900752	J	-0.57	1.15E-07	9	A	G	downstream_gene_variant	LOC_Os09g12200	-	'35.2'_'not_assigned.unknown'
PGC9.2	snp_09_6905233	J	-0.57	1.15E-07	9	C	G	intergenic_region	LOC_Os09g12200-LOC_Os09g12210	-	NA
PGC9.3	snp_09_17170796	J	-0.85	2.59E-06	9	C	T	upstream_gene_variant	LOC_Os09g28290	-	'35.2'_'not_assigned.unknown'
PGC9.4	snp_09_19571667	J	0.45	1.81E-06	9	A	G	synonymous_variant	LOC_Os09g32820	p.Gln151Gln	'23.3.1.3'_'nucleotide_metabolism.salvage.phosphoribosyltransferases.upp'
PGC10.1	snp_10_447233	J	0.73	1.24E-06	10	G	A	upstream_gene_variant	LOC_Os10g01670	-	'35.2'_'not_assigned.unknown'
PGC10.2	snp_10_20874591	J	-0.55	6.06E-07	10	C	T	downstream_gene_variant	LOC_Os10g39130	-	'27.3.24'_'RNA.regulation_of_transcription.MADS_box_transcription_factor_family'

QTL ID	SNP ID	Allele†	Beta	P-value	Chr	Ref	Alt	Region	Gene	Amino acid change	GoMAPMAN
PGC10.3	snp_10_21798319	J	-0.87	1.72E-06	10	T	C	upstream_gene_variant	LOC_Os10g40640	-	'35.1'_'not_assigned.no_ontology'
PGC11.1	snp_11_6246057	J	-0.89	1.34E-06	11	C	T	upstream_gene_variant	LOC_Os11g11280	-	'35.2'_'not_assigned.unknown'
PGC11.2	snp_11_10259061	J	-0.58	1.19E-06	11	G	A	intron_variant	LOC_Os11g18194	-	'17.3.1.2.99'_'hormone_metabolism.brassinosteroid.synthesis-degradation.sterols.other'
PGC11.3	snp_11_11126781	J	-0.65	4.13E-07	11	C	T	upstream_gene_variant	LOC_Os11g19350	-	'35.2'_'not_assigned.unknown'
PGC11.3	snp_11_11150956	J	-0.63	2.93E-06	11	G	A	synonymous_variant	LOC_Os11g19380	p.Ala76Ala	'35.2'_'not_assigned.unknown'
PGC11.3	snp_11_11154123	J	-0.65	4.21E-07	11	C	T	upstream_gene_variant	LOC_Os11g19390	-	'35.2'_'not_assigned.unknown'
PGC11.3	snp_11_11184639	J	-0.65	3.98E-07	11	C	T	downstream_gene_variant	LOC_Os11g19440	-	'35.2'_'not_assigned.unknown'
PGC11.3	snp_11_11197785	J	-0.70	1.26E-07	11	C	T	upstream_gene_variant	LOC_Os11g19450	-	'35.2'_'not_assigned.unknown'
PGC11.3	snp_11_11198781	J	-0.65	4.21E-07	11	C	T	upstream_gene_variant	LOC_Os11g19450	-	'35.2'_'not_assigned.unknown'
PGC11.4	snp_11_14301203	J	-0.70	2.36E-07	11	C	T	upstream_gene_variant	LOC_Os11g25080	-	'35.2'_'not_assigned.unknown'
PGC11.4	snp_11_14370139	J	-0.70	2.39E-07	11	C	T	upstream_gene_variant	LOC_Os11g25230	-	'26.8'_'misc.nitrilases,_*nitrile_lyases,_berberine_bridge_enzymes,_reticuline_oxidases,_troponine_reductases'
PGC11.4	snp_11_14374189	J	-0.76	5.92E-08	11	C	T	downstream_gene_variant	LOC_Os11g25230	-	'26.8'_'misc.nitrilases,_*nitrile_lyases,_berberine_bridge_enzymes,_reticuline_oxidases,_troponine_reductases'
PGC11.4	snp_11_14385117	J	-0.70	2.35E-07	11	G	A	upstream_gene_variant	LOC_Os11g25240	-	'35.2'_'not_assigned.unknown'
PGC11.4	snp_11_14435883	J	-0.70	2.34E-07	11	G	A	upstream_gene_variant	LOC_Os11g25330	-	'23.2'_'nucleotide_metabolism.degradation'
PGC11.4	snp_11_14511763	J	-0.70	2.39E-07	11	C	G	intron_variant	LOC_Os11g25454	-	'26.2'_'misc.UDP_glucosyl_and_glucoronyl_transferases'
PGC11.4	snp_11_14530506	J	-0.70	2.39E-07	11	T	C	downstream_gene_variant	LOC_Os11g25510	-	'30.2.24'_'signalling.receptor_kinases.S-locus_glycoprotein_like'
PGC11.4	snp_11_14565502	J	0.42	1.62E-06	11	G	T	upstream_gene_variant	LOC_Os11g25550	-	'35.2'_'not_assigned.unknown'
PGC11.4	snp_11_14632460	J	-0.69	1.20E-07	11	G	A	upstream_gene_variant	LOC_Os11g25650	-	'35.2'_'not_assigned.unknown'
PGC11.4	snp_11_14637639	J	-0.70	2.57E-07	11	C	T	synonymous_variant	LOC_Os11g25650	p.His124His	'35.2'_'not_assigned.unknown'
PGC11.4	snp_11_14651854	J	-0.64	5.26E-07	11	C	T	upstream_gene_variant	LOC_Os11g25670	-	'35.2'_'not_assigned.unknown'
PGC11.4	snp_11_14657761	J	0.40	7.45E-08	11	G	A	upstream_gene_variant	LOC_Os11g25700	-	'26.8'_'misc.nitrilases,_*nitrile_lyases,_berberine_bridge_enzymes,_reticuline_oxidases,_troponine_reductases'
PGC11.4	snp_11_14684806	J	-0.64	5.24E-07	11	C	T	synonymous_variant	LOC_Os11g25750	p.Ser263Ser	'35.2'_'not_assigned.unknown'
PGC11.4	snp_11_14691120	J	0.37	5.62E-07	11	A	G	upstream_gene_variant	LOC_Os11g25750	-	'35.2'_'not_assigned.unknown'
PGC11.4	snp_11_14710222	J	0.35	1.78E-06	11	G	A	intron_variant	LOC_Os11g25780	-	'35.1'_'not_assigned.no_ontology'
PGC11.4	snp_11_14716008	J	-0.64	5.27E-07	11	T	C	downstream_gene_variant	LOC_Os11g25780	-	'35.1'_'not_assigned.no_ontology'
PGC11.4	snp_11_14735906	J	-0.64	5.27E-07	11	C	T	upstream_gene_variant	LOC_Os11g25850	-	'35.2'_'not_assigned.unknown'
PGC11.4	snp_11_14880355	J	-0.70	2.39E-07	11	G	A	upstream_gene_variant	LOC_Os11g26020	-	'35.2'_'not_assigned.unknown'
PGC11.4	snp_11_14895621	J	-0.70	2.37E-07	11	C	T	missense_variant	LOC_Os11g26040	p.Ala167Val	'35.2'_'not_assigned.unknown'
PGC11.4	snp_11_14971461	J	-0.70	2.34E-07	11	G	T	upstream_gene_variant	LOC_Os11g26160	-	'27.3.85'_'RNA.regulation_of_transcription.sigma_like_plant'
PGC11.4	snp_11_15058869	J	-0.70	2.33E-07	11	C	T	upstream_gene_variant	LOC_Os11g26290	-	'35.2'_'not_assigned.unknown'
PGC11.4	snp_11_15114369	J	-0.70	2.41E-07	11	T	G	missense_variant	LOC_Os11g26390	p.Asn314His	'26.25'_'misc.sulfotransferase'
PGC11.4	snp_11_15131879	J	0.34	1.76E-06	11	A	G	upstream_gene_variant	LOC_Os11g26420	-	'35.2'_'not_assigned.unknown'

QTL ID	SNP ID	Allele†	Beta	P-value	Chr	Ref	Alt	Region	Gene	Amino acid change	GoMAPMAN
PGC11.4	snp_11_15164790	J	-0.70	2.20E-07	11	C	T	intergenic_region	LOC_Os11g26460-LOC_Os11g26470	-	NA
PGC11.4	snp_11_15170171	J	-0.70	2.35E-07	11	C	T	upstream_gene_variant	LOC_Os11g26470	-	'35.2'_'not_assigned.unknown'
PGC11.4	snp_11_15443889	J	-0.71	5.12E-07	11	C	T	missense_variant	LOC_Os11g26870	p.Ala55Thr	'35.2'_'not_assigned.unknown'
PGC11.5	snp_11_28872156	J	-0.97	2.59E-07	11	A	G	missense_variant	LOC_Os11g47870	p.Asn665Asp	'27.3.21'_'RNA.regulation_of_transcription.GRAS_transcription_factor_family'
PGC11.5	snp_11_28889703	J	-1.02	1.11E-07	11	T	G	upstream_gene_variant	LOC_Os11g47900	-	'27.3.21'_'RNA.regulation_of_transcription.GRAS_transcription_factor_family'
PGC11.5	snp_11_28924197	J	-0.80	2.78E-06	11	C	T	upstream_gene_variant	LOC_Os11g47944	-	'20.1'_'stress.biotic'
PGC12.1	snp_12_2419472	J	-1.17	1.19E-10	12	G	A	downstream_gene_variant	LOC_Os12g05360	-	'35.2'_'not_assigned.unknown'
PGC12.2	snp_12_3384075	J	-0.89	3.01E-07	12	C	T	downstream_gene_variant	LOC_Os12g06920	-	'20.1'_'stress.biotic'
PGC12.2	snp_12_3415749	J	-0.89	3.02E-07	12	G	A	upstream_gene_variant	LOC_Os12g06980	-	'35.2'_'not_assigned.unknown'
PGC12.2	snp_12_3423375	J	-0.89	3.03E-07	12	C	A	upstream_gene_variant	LOC_Os12g06990	-	'35.2'_'not_assigned.unknown'
PGC12.2	snp_12_3429125	J	-0.89	3.09E-07	12	A	T	upstream_gene_variant	LOC_Os12g07000	-	'35.2'_'not_assigned.unknown'
PGC12.2	snp_12_3588777	J	-0.75	1.02E-06	12	G	T	upstream_gene_variant	LOC_Os12g07300	-	'35.2'_'not_assigned.unknown'
PGC12.3	snp_12_10819584	J	-0.68	1.14E-06	12	C	A	upstream_gene_variant	LOC_Os12g18729	-	'35.2'_'not_assigned.unknown'
PGC12.3	snp_12_10819586	J	-0.69	1.05E-06	12	T	C	upstream_gene_variant	LOC_Os12g18729	-	'35.2'_'not_assigned.unknown'
PGC12.4	snp_12_21592589	J	-0.50	3.46E-07	12	C	T	downstream_gene_variant	LOC_Os12g35510	-	'35.2'_'not_assigned.unknown'
PGC12.4	snp_12_21599823	J	-0.50	1.86E-06	12	G	A	upstream_gene_variant	LOC_Os12g35510	-	'35.2'_'not_assigned.unknown'
PGC12.4	snp_12_21603304	J	-0.48	6.93E-07	12	A	G	upstream_gene_variant	LOC_Os12g35520	-	'35.2'_'not_assigned.unknown'
PGC12.4	snp_12_21709876	J	-0.50	1.55E-06	12	C	T	downstream_gene_variant	LOC_Os12g35710	-	'10.5.3'_'cell_wall.cell_wall_proteins.LRR'
PGC12.4	snp_12_21734529	J	-0.45	2.82E-06	12	G	A	downstream_gene_variant	LOC_Os12g35720	-	'35.2'_'not_assigned.unknown'
PGC12.4	snp_12_21825842	J	-0.40	1.88E-06	12	A	G	missense_variant	LOC_Os12g35810	p.Gln104Arg	'30.2.17'_'signalling.receptor_kinases.DUF_26'
PGC12.4	snp_12_21829957	J	-0.31	2.93E-06	12	G	A	upstream_gene_variant	LOC_Os12g35820	-	'35.2'_'not_assigned.unknown'
PGC12.4	snp_12_21831804	J	-0.29	2.93E-06	12	A	T	upstream_gene_variant	LOC_Os12g35820	-	'35.2'_'not_assigned.unknown'
PGC12.4	snp_12_21832632	J	-0.30	2.83E-06	12	C	T	upstream_gene_variant	LOC_Os12g35820	-	'35.2'_'not_assigned.unknown'
PGC12.4	snp_12_21832960	J	-0.30	5.63E-07	12	C	T	upstream_gene_variant	LOC_Os12g35820	-	'35.2'_'not_assigned.unknown'
PGC12.4	snp_12_21852966	J	-0.32	1.74E-06	12	G	A	downstream_gene_variant	LOC_Os12g35860	-	'35.2'_'not_assigned.unknown'
PGC12.4	snp_12_21852971	J	-0.31	2.47E-06	12	C	T	downstream_gene_variant	LOC_Os12g35860	-	'35.2'_'not_assigned.unknown'
PGC12.4	snp_12_21853367	J	-0.31	2.65E-06	12	C	A	downstream_gene_variant	LOC_Os12g35860	-	'35.2'_'not_assigned.unknown'
PGC12.4	snp_12_21880082	J	-0.50	5.33E-07	12	G	T	downstream_gene_variant	LOC_Os12g35895	-	'35.2'_'not_assigned.unknown'
PGC12.4	snp_12_21889863	J	-0.50	3.79E-07	12	C	T	missense_variant	LOC_Os12g35920	p.Glu94Lys	'35.2'_'not_assigned.unknown'

†represent the source subspecies showing the major occurrence of alternate allele, where J and I indicate *Japonica* , and *Indica* subspecies, respectively.

Table S3: Genome wide epistasis interaction targets identified from the GWAS genetic variants associated with grain chalkiness.

Region 1				Region 2				p-value of interaction
SNP1	QTL1	Locus ID	Functional categories	SNP2	QTL2	Locus ID	Functional categories	
snp_01_1548861	pgc1.1	LOC_Os01g03720	27.3.25'_'RNA.regulation_of_transcription.MYB_domain_transcription_factor_family'	snp_04_31033053	pgc4.4	LOC_Os04g52240	'17.6.1.2'_'hormone_metabolism.gibberelin.synthesis-degradation.ent-kaurene_synthase'	4.69E-09
snp_01_1548874	pgc1.1	LOC_Os01g03720	'27.3.25'_'RNA.regulation_of_transcription.MYB_domain_transcription_factor_family'	snp_04_31033053	pgc4.4	LOC_Os04g52240	'17.6.1.2'_'hormone_metabolism.gibberelin.synthesis-degradation.ent-kaurene_synthase'	1.46E-08
snp_01_1548861	pgc1.1	LOC_Os01g03720	'27.3.25'_'RNA.regulation_of_transcription.MYB_domain_transcription_factor_family'	snp_04_31035500	pgc4.4	LOC_Os04g52230	'17.6.1.2'_'hormone_metabolism.gibberelin.synthesis-degradation.ent-kaurene_synthase'	1.95E-08
snp_01_1548876	pgc1.1	LOC_Os01g03720	'27.3.25'_'RNA.regulation_of_transcription.MYB_domain_transcription_factor_family'	snp_04_31033053	pgc4.4	LOC_Os04g52240	'17.6.1.2'_'hormone_metabolism.gibberelin.synthesis-degradation.ent-kaurene_synthase'	2.14E-08
snp_01_1548861	pgc1.1	LOC_Os01g03720	'27.3.25'_'RNA.regulation_of_transcription.MYB_domain_transcription_factor_family'	snp_04_31033185	pgc4.4	LOC_Os04g52240	'17.6.1.2'_'hormone_metabolism.gibberelin.synthesis-degradation.ent-kaurene_synthase'	2.62E-08
snp_01_1548861	pgc1.1	LOC_Os01g03720	'27.3.25'_'RNA.regulation_of_transcription.MYB_domain_transcription_factor_family'	snp_04_31024611	pgc4.4	LOC_Os04g52220	'35.2'_'not_assigned.unknown'	3.27E-08
snp_01_1548861	pgc1.1	LOC_Os01g03720	'27.3.25'_'RNA.regulation_of_transcription.MYB_domain_transcription_factor_family'	snp_04_30942194	pgc4.4	LOC_Os04g52090	'35.2'_'not_assigned.unknown'	3.59E-08
snp_01_1548861	pgc1.1	LOC_Os01g03720	'27.3.25'_'RNA.regulation_of_transcription.MYB_domain_transcription_factor_family'	snp_04_31024925	pgc4.4	LOC_Os04g52220	'35.2'_'not_assigned.unknown'	3.6E-08
snp_01_1548861	pgc1.1	LOC_Os01g03720	'27.3.25'_'RNA.regulation_of_transcription.MYB_domain_transcription_factor_family'	snp_04_31035704	pgc4.4	LOC_Os04g52230	'17.6.1.2'_'hormone_metabolism.gibberelin.synthesis-degradation.ent-kaurene_synthase'	3.68E-08
snp_01_1548861	pgc1.1	LOC_Os01g03720	'27.3.25'_'RNA.regulation_of_transcription.MYB_domain_transcription_factor_family'	snp_04_31025209	pgc4.4	LOC_Os04g52230	'17.6.1.2'_'hormone_metabolism.gibberelin.synthesis-degradation.ent-kaurene_synthase'	3.85E-08
snp_01_1548861	pgc1.1	LOC_Os01g03720	'27.3.25'_'RNA.regulation_of_transcription.MYB_domain_transcription_factor_family'	snp_04_31029025	pgc4.4	LOC_Os04g52230	'17.6.1.2'_'hormone_metabolism.gibberelin.synthesis-degradation.ent-kaurene_synthase'	3.9E-08
snp_01_1548861	pgc1.1	LOC_Os01g03720	'27.3.25'_'RNA.regulation_of_transcription.MYB_domain_transcription_factor_family'	snp_04_31029425	pgc4.4	LOC_Os04g52220	'35.2'_'not_assigned.unknown'	3.94E-08
snp_01_1548861	pgc1.1	LOC_Os01g03720	'27.3.25'_'RNA.regulation_of_transcription.MYB_domain_transcription_factor_family'	snp_04_31025026	pgc4.4	LOC_Os04g52220	'35.2'_'not_assigned.unknown'	4.14E-08
snp_01_1548861	pgc1.1	LOC_Os01g03720	'27.3.25'_'RNA.regulation_of_transcription.MYB_domain_transcription_factor_family'	snp_04_31032879	pgc4.4	LOC_Os04g52240	'17.6.1.2'_'hormone_metabolism.gibberelin.synthesis-degradation.ent-kaurene_synthase'	4.39E-08
snp_01_1548861	pgc1.1	LOC_Os01g03720	'27.3.25'_'RNA.regulation_of_transcription.MYB_domain_transcription_factor_family'	snp_04_30942215	pgc4.4	LOC_Os04g52090	'35.2'_'not_assigned.unknown'	4.42E-08
snp_01_1548861	pgc1.1	LOC_Os01g03720	'27.3.25'_'RNA.regulation_of_transcription.MYB_domain_transcription_factor_family'	snp_04_31029056	pgc4.4	LOC_Os04g52230	'17.6.1.2'_'hormone_metabolism.gibberelin.synthesis-degradation.ent-kaurene_synthase'	4.54E-08
snp_01_1548861	pgc1.1	LOC_Os01g03720	'27.3.25'_'RNA.regulation_of_transcription.MYB_domain_transcription_factor_family'	snp_04_31035595	pgc4.4	LOC_Os04g52230	'17.6.1.2'_'hormone_metabolism.gibberelin.synthesis-degradation.ent-kaurene_synthase'	4.75E-08
snp_01_1548861	pgc1.1	LOC_Os01g03720	'27.3.25'_'RNA.regulation_of_transcription.MYB_domain_transcription_factor_family'	snp_04_31025109	pgc4.4	LOC_Os04g52230	'17.6.1.2'_'hormone_metabolism.gibberelin.synthesis-degradation.ent-kaurene_synthase'	4.78E-08
snp_01_1548861	pgc1.1	LOC_Os01g03720	'27.3.25'_'RNA.regulation_of_transcription.MYB_domain_transcription_factor_family'	snp_04_31032724	pgc4.4	LOC_Os04g52240	'17.6.1.2'_'hormone_metabolism.gibberelin.synthesis-degradation.ent-kaurene_synthase'	4.83E-08
snp_01_1548861	pgc1.1	LOC_Os01g03720	'27.3.25'_'RNA.regulation_of_transcription.MYB_domain_transcription_factor_family'	snp_04_31024760	pgc4.4	LOC_Os04g52220	'35.2'_'not_assigned.unknown'	5.21E-08
snp_01_1548861	pgc1.1	LOC_Os01g03720	'27.3.25'_'RNA.regulation_of_transcription.MYB_domain_transcription_factor_family'	snp_04_31033239	pgc4.4	LOC_Os04g52240	'17.6.1.2'_'hormone_metabolism.gibberelin.synthesis-degradation.ent-kaurene_synthase'	5.7E-08
snp_01_1548874	pgc1.1	LOC_Os01g03720	'27.3.25'_'RNA.regulation_of_transcription.MYB_domain_transcription_factor_family'	snp_04_31035500	pgc4.4	LOC_Os04g52230	'17.6.1.2'_'hormone_metabolism.gibberelin.synthesis-degradation.ent-kaurene_synthase'	5.71E-08
snp_01_1548861	pgc1.1	LOC_Os01g03720	'27.3.25'_'RNA.regulation_of_transcription.MYB_domain_transcription_factor_family'	snp_04_31030123	pgc4.4	LOC_Os04g52240	'17.6.1.2'_'hormone_metabolism.gibberelin.synthesis-degradation.ent-kaurene_synthase'	5.84E-08
snp_01_1548861	pgc1.1	LOC_Os01g03720	'27.3.25'_'RNA.regulation_of_transcription.MYB_domain_transcription_factor_family'	snp_04_31030699	pgc4.4	LOC_Os04g52230	'17.6.1.2'_'hormone_metabolism.gibberelin.synthesis-degradation.ent-kaurene_synthase'	6.31E-08

Region 1				Region 2				p-value of interaction
SNP1	QTL1	Locus ID	Functional categories	SNP2	QTL2	Locus ID	Functional categories	
snp_01_1548874	pgc1.1	LOC_Os01g03720	'27.3.25'_'RNA.regulation_of_transcription.MYB_domain_transcription_factor_family'	snp_04_31033185	pgc4.4	LOC_Os04g52240	'17.6.1.2'_'hormone_metabolism.gibberelin.synthesis-degradation.ent-kaurene_synthase'	7.72E-08
snp_01_1548861	pgc1.1	LOC_Os01g03720	'27.3.25'_'RNA.regulation_of_transcription.MYB_domain_transcription_factor_family'	snp_04_31032653	pgc4.4	LOC_Os04g52240	'17.6.1.2'_'hormone_metabolism.gibberelin.synthesis-degradation.ent-kaurene_synthase'	8.03E-08
snp_01_1548876	pgc1.1	LOC_Os01g03720	'27.3.25'_'RNA.regulation_of_transcription.MYB_domain_transcription_factor_family'	snp_04_31035500	pgc4.4	LOC_Os04g52230	'17.6.1.2'_'hormone_metabolism.gibberelin.synthesis-degradation.ent-kaurene_synthase'	8.07E-08
snp_01_1548861	pgc1.1	LOC_Os01g03720	'27.3.25'_'RNA.regulation_of_transcription.MYB_domain_transcription_factor_family'	snp_04_31027329	pgc4.4	LOC_Os04g52210	'17.6.1.2'_'hormone_metabolism.gibberelin.synthesis-degradation.ent-kaurene_synthase'	8.4E-08
snp_01_1548874	pgc1.1	LOC_Os01g03720	'27.3.25'_'RNA.regulation_of_transcription.MYB_domain_transcription_factor_family'	snp_04_31024611	pgc4.4	LOC_Os04g52220	'35.2'_'not_assigned.unknown'	9.39E-08
snp_01_1548861	pgc1.1	LOC_Os01g03720	'27.3.25'_'RNA.regulation_of_transcription.MYB_domain_transcription_factor_family'	snp_04_31212801	pgc4.4	LOC_Os04g52479	'35.2'_'not_assigned.unknown'	1.01E-07
snp_01_1548874	pgc1.1	LOC_Os01g03720	'27.3.25'_'RNA.regulation_of_transcription.MYB_domain_transcription_factor_family'	snp_04_31024925	pgc4.4	LOC_Os04g52220	'35.2'_'not_assigned.unknown'	1.04E-07
snp_01_1548874	pgc1.1	LOC_Os01g03720	'27.3.25'_'RNA.regulation_of_transcription.MYB_domain_transcription_factor_family'	snp_04_31035704	pgc4.4	LOC_Os04g52230	'17.6.1.2'_'hormone_metabolism.gibberelin.synthesis-degradation.ent-kaurene_synthase'	1.06E-07
snp_01_1548876	pgc1.1	LOC_Os01g03720	'27.3.25'_'RNA.regulation_of_transcription.MYB_domain_transcription_factor_family'	snp_04_31033185	pgc4.4	LOC_Os04g52240	'17.6.1.2'_'hormone_metabolism.gibberelin.synthesis-degradation.ent-kaurene_synthase'	1.09E-07
snp_01_1548874	pgc1.1	LOC_Os01g03720	'27.3.25'_'RNA.regulation_of_transcription.MYB_domain_transcription_factor_family'	snp_04_31025209	pgc4.4	LOC_Os04g52230	'17.6.1.2'_'hormone_metabolism.gibberelin.synthesis-degradation.ent-kaurene_synthase'	1.11E-07
snp_01_1548874	pgc1.1	LOC_Os01g03720	'27.3.25'_'RNA.regulation_of_transcription.MYB_domain_transcription_factor_family'	snp_04_31029025	pgc4.4	LOC_Os04g52230	'17.6.1.2'_'hormone_metabolism.gibberelin.synthesis-degradation.ent-kaurene_synthase'	1.12E-07
snp_01_1548874	pgc1.1	LOC_Os01g03720	'27.3.25'_'RNA.regulation_of_transcription.MYB_domain_transcription_factor_family'	snp_04_31029425	pgc4.4	LOC_Os04g52220	'35.2'_'not_assigned.unknown'	1.13E-07
snp_01_1548874	pgc1.1	LOC_Os01g03720	'27.3.25'_'RNA.regulation_of_transcription.MYB_domain_transcription_factor_family'	snp_04_31025026	pgc4.4	LOC_Os04g52220	'35.2'_'not_assigned.unknown'	1.19E-07
snp_01_1548874	pgc1.1	LOC_Os01g03720	'27.3.25'_'RNA.regulation_of_transcription.MYB_domain_transcription_factor_family'	snp_04_31032879	pgc4.4	LOC_Os04g52240	'17.6.1.2'_'hormone_metabolism.gibberelin.synthesis-degradation.ent-kaurene_synthase'	1.28E-07
snp_01_1548874	pgc1.1	LOC_Os01g03720	'27.3.25'_'RNA.regulation_of_transcription.MYB_domain_transcription_factor_family'	snp_04_31029056	pgc4.4	LOC_Os04g52230	'17.6.1.2'_'hormone_metabolism.gibberelin.synthesis-degradation.ent-kaurene_synthase'	1.31E-07
snp_01_1548876	pgc1.1	LOC_Os01g03720	'27.3.25'_'RNA.regulation_of_transcription.MYB_domain_transcription_factor_family'	snp_04_31024611	pgc4.4	LOC_Os04g52220	'35.2'_'not_assigned.unknown'	1.31E-07
snp_01_1548874	pgc1.1	LOC_Os01g03720	'27.3.25'_'RNA.regulation_of_transcription.MYB_domain_transcription_factor_family'	snp_04_31032724	pgc4.4	LOC_Os04g52240	'17.6.1.2'_'hormone_metabolism.gibberelin.synthesis-degradation.ent-kaurene_synthase'	1.37E-07
snp_01_1548874	pgc1.1	LOC_Os01g03720	'27.3.25'_'RNA.regulation_of_transcription.MYB_domain_transcription_factor_family'	snp_04_31025109	pgc4.4	LOC_Os04g52230	'17.6.1.2'_'hormone_metabolism.gibberelin.synthesis-degradation.ent-kaurene_synthase'	1.37E-07
snp_01_1548874	pgc1.1	LOC_Os01g03720	'27.3.25'_'RNA.regulation_of_transcription.MYB_domain_transcription_factor_family'	snp_04_31035595	pgc4.4	LOC_Os04g52230	'17.6.1.2'_'hormone_metabolism.gibberelin.synthesis-degradation.ent-kaurene_synthase'	1.38E-07
snp_01_1548876	pgc1.1	LOC_Os01g03720	'27.3.25'_'RNA.regulation_of_transcription.MYB_domain_transcription_factor_family'	snp_04_30942194	pgc4.4	LOC_Os04g52090	'35.2'_'not_assigned.unknown'	1.38E-07
snp_01_1548876	pgc1.1	LOC_Os01g03720	'27.3.25'_'RNA.regulation_of_transcription.MYB_domain_transcription_factor_family'	snp_04_31024925	pgc4.4	LOC_Os04g52220	'35.2'_'not_assigned.unknown'	1.44E-07
snp_01_1548876	pgc1.1	LOC_Os01g03720	'27.3.25'_'RNA.regulation_of_transcription.MYB_domain_transcription_factor_family'	snp_04_31035704	pgc4.4	LOC_Os04g52230	'17.6.1.2'_'hormone_metabolism.gibberelin.synthesis-degradation.ent-kaurene_synthase'	1.48E-07
snp_01_1548874	pgc1.1	LOC_Os01g03720	'27.3.25'_'RNA.regulation_of_transcription.MYB_domain_transcription_factor_family'	snp_04_31024760	pgc4.4	LOC_Os04g52220	'35.2'_'not_assigned.unknown'	0.00000015
snp_01_1548876	pgc1.1	LOC_Os01g03720	'27.3.25'_'RNA.regulation_of_transcription.MYB_domain_transcription_factor_family'	snp_04_31025209	pgc4.4	LOC_Os04g52230	'17.6.1.2'_'hormone_metabolism.gibberelin.synthesis-degradation.ent-kaurene_synthase'	1.54E-07
snp_01_1548876	pgc1.1	LOC_Os01g03720	'27.3.25'_'RNA.regulation_of_transcription.MYB_domain_transcription_factor_family'	snp_04_31029025	pgc4.4	LOC_Os04g52230	'17.6.1.2'_'hormone_metabolism.gibberelin.synthesis-degradation.ent-kaurene_synthase'	1.56E-07

Region 1				Region 2				p-value of interaction
SNP1	QTL1	Locus ID	Functional categories	SNP2	QTL2	Locus ID	Functional categories	
snp_01_1548876	pgc1.1	LOC_Os01g03720	'27.3.25'_'RNA.regulation_of_transcription.MYB_domain_transcription_factor_family'	snp_04_31029425	pgc4.4	LOC_Os04g52220	'35.2'_'not_assigned.unknown'	1.56E-07
snp_01_1548861	pgc1.1	LOC_Os01g03720	'27.3.25'_'RNA.regulation_of_transcription.MYB_domain_transcription_factor_family'	snp_04_31009177	pgc4.4	LOC_Os04g52190	'29.3.4.3'_'protein.targeting.secretory_pathway.vacuole'	1.58E-07
snp_01_1548876	pgc1.1	LOC_Os01g03720	'27.3.25'_'RNA.regulation_of_transcription.MYB_domain_transcription_factor_family'	snp_04_31025026	pgc4.4	LOC_Os04g52220	'35.2'_'not_assigned.unknown'	1.65E-07
snp_01_1548874	pgc1.1	LOC_Os01g03720	'27.3.25'_'RNA.regulation_of_transcription.MYB_domain_transcription_factor_family'	snp_04_31033239	pgc4.4	LOC_Os04g52240	'17.6.1.2'_'hormone_metabolism.gibberelin.synthesis-degradation.ent-kaurene_synthase'	1.66E-07
snp_01_1548874	pgc1.1	LOC_Os01g03720	'27.3.25'_'RNA.regulation_of_transcription.MYB_domain_transcription_factor_family'	snp_04_31030123	pgc4.4	LOC_Os04g52240	'17.6.1.2'_'hormone_metabolism.gibberelin.synthesis-degradation.ent-kaurene_synthase'	1.67E-07
snp_01_1548876	pgc1.1	LOC_Os01g03720	'27.3.25'_'RNA.regulation_of_transcription.MYB_domain_transcription_factor_family'	snp_04_30942215	pgc4.4	LOC_Os04g52090	'35.2'_'not_assigned.unknown'	1.74E-07
snp_01_1548876	pgc1.1	LOC_Os01g03720	'27.3.25'_'RNA.regulation_of_transcription.MYB_domain_transcription_factor_family'	snp_04_31032879	pgc4.4	LOC_Os04g52240	'17.6.1.2'_'hormone_metabolism.gibberelin.synthesis-degradation.ent-kaurene_synthase'	1.79E-07
snp_01_7687212	pgc1.2	LOC_Os01g13740	'27.3.20'_'RNA.regulation_of_transcription.G2-like_transcription_factor_family,_GARP'	snp_04_31035500	pgc4.4	LOC_Os04g52230	'17.6.1.2'_'hormone_metabolism.gibberelin.synthesis-degradation.ent-kaurene_synthase'	1.46E-15
snp_01_7687212	pgc1.2	LOC_Os01g13740	'27.3.20'_'RNA.regulation_of_transcription.G2-like_transcription_factor_family,_GARP'	snp_04_31030123	pgc4.4	LOC_Os04g52240	'17.6.1.2'_'hormone_metabolism.gibberelin.synthesis-degradation.ent-kaurene_synthase'	1.84E-14
snp_01_7687212	pgc1.2	LOC_Os01g13740	'27.3.20'_'RNA.regulation_of_transcription.G2-like_transcription_factor_family,_GARP'	snp_04_31024925	pgc4.4	LOC_Os04g52220	'35.2'_'not_assigned.unknown'	1.89E-14
snp_01_7687212	pgc1.2	LOC_Os01g13740	'27.3.20'_'RNA.regulation_of_transcription.G2-like_transcription_factor_family,_GARP'	snp_04_31035704	pgc4.4	LOC_Os04g52230	'17.6.1.2'_'hormone_metabolism.gibberelin.synthesis-degradation.ent-kaurene_synthase'	1.92E-14
snp_01_7687212	pgc1.2	LOC_Os01g13740	'27.3.20'_'RNA.regulation_of_transcription.G2-like_transcription_factor_family,_GARP'	snp_04_31029025	pgc4.4	LOC_Os04g52230	'17.6.1.2'_'hormone_metabolism.gibberelin.synthesis-degradation.ent-kaurene_synthase'	1.94E-14
snp_01_7687212	pgc1.2	LOC_Os01g13740	'27.3.20'_'RNA.regulation_of_transcription.G2-like_transcription_factor_family,_GARP'	snp_04_31029425	pgc4.4	LOC_Os04g52220	'35.2'_'not_assigned.unknown'	2.1E-14
snp_01_7687212	pgc1.2	LOC_Os01g13740	'27.3.20'_'RNA.regulation_of_transcription.G2-like_transcription_factor_family,_GARP'	snp_04_31025209	pgc4.4	LOC_Os04g52230	'17.6.1.2'_'hormone_metabolism.gibberelin.synthesis-degradation.ent-kaurene_synthase'	2.18E-14
snp_01_7687212	pgc1.2	LOC_Os01g13740	'27.3.20'_'RNA.regulation_of_transcription.G2-like_transcription_factor_family,_GARP'	snp_04_31029056	pgc4.4	LOC_Os04g52230	'17.6.1.2'_'hormone_metabolism.gibberelin.synthesis-degradation.ent-kaurene_synthase'	2.24E-14
snp_01_7687212	pgc1.2	LOC_Os01g13740	'27.3.20'_'RNA.regulation_of_transcription.G2-like_transcription_factor_family,_GARP'	snp_04_31035595	pgc4.4	LOC_Os04g52230	'17.6.1.2'_'hormone_metabolism.gibberelin.synthesis-degradation.ent-kaurene_synthase'	2.34E-14
snp_01_7687212	pgc1.2	LOC_Os01g13740	'27.3.20'_'RNA.regulation_of_transcription.G2-like_transcription_factor_family,_GARP'	snp_04_31025026	pgc4.4	LOC_Os04g52220	'35.2'_'not_assigned.unknown'	2.44E-14
snp_01_7687212	pgc1.2	LOC_Os01g13740	'27.3.20'_'RNA.regulation_of_transcription.G2-like_transcription_factor_family,_GARP'	snp_04_31032879	pgc4.4	LOC_Os04g52240	'17.6.1.2'_'hormone_metabolism.gibberelin.synthesis-degradation.ent-kaurene_synthase'	2.49E-14
snp_01_7687212	pgc1.2	LOC_Os01g13740	'27.3.20'_'RNA.regulation_of_transcription.G2-like_transcription_factor_family,_GARP'	snp_04_31032724	pgc4.4	LOC_Os04g52240	'17.6.1.2'_'hormone_metabolism.gibberelin.synthesis-degradation.ent-kaurene_synthase'	2.68E-14
snp_01_7687212	pgc1.2	LOC_Os01g13740	'27.3.20'_'RNA.regulation_of_transcription.G2-like_transcription_factor_family,_GARP'	snp_04_31033185	pgc4.4	LOC_Os04g52240	'17.6.1.2'_'hormone_metabolism.gibberelin.synthesis-degradation.ent-kaurene_synthase'	2.78E-14
snp_01_7687212	pgc1.2	LOC_Os01g13740	'27.3.20'_'RNA.regulation_of_transcription.G2-like_transcription_factor_family,_GARP'	snp_04_31024611	pgc4.4	LOC_Os04g52220	'35.2'_'not_assigned.unknown'	2.97E-14
snp_01_7687212	pgc1.2	LOC_Os01g13740	'27.3.20'_'RNA.regulation_of_transcription.G2-like_transcription_factor_family,_GARP'	snp_04_31033239	pgc4.4	LOC_Os04g52240	'17.6.1.2'_'hormone_metabolism.gibberelin.synthesis-degradation.ent-kaurene_synthase'	4.08E-14
snp_01_7687212	pgc1.2	LOC_Os01g13740	'27.3.20'_'RNA.regulation_of_transcription.G2-like_transcription_factor_family,_GARP'	snp_04_31009177	pgc4.4	LOC_Os04g52190	'29.3.4.3'_'protein.targeting.secretory_pathway.vacuole'	4.15E-14
snp_01_7687212	pgc1.2	LOC_Os01g13740	'27.3.20'_'RNA.regulation_of_transcription.G2-like_transcription_factor_family,_GARP'	snp_03_6728014	pgc3.3	s03g12640-LOC_Os03g	NA	4.68E-14
snp_01_7687212	pgc1.2	LOC_Os01g13740	'27.3.20'_'RNA.regulation_of_transcription.G2-like_transcription_factor_family,_GARP'	snp_04_31025109	pgc4.4	LOC_Os04g52230	'17.6.1.2'_'hormone_metabolism.gibberelin.synthesis-degradation.ent-kaurene_synthase'	5.47E-14

Region 1				Region 2				p-value of interaction
SNP1	QTL1	Locus ID	Functional categories	SNP2	QTL2	Locus ID	Functional categories	
snp_01_7687212	pgc1.2	LOC_Os01g13740	'27.3.20'_'RNA.regulation_of_transcription.G2-like_transcription_factor_family,_GARP'	snp_04_31030699	pgc4.4	LOC_Os04g52230	'17.6.1.2'_'hormone_metabolism.gibberelin.synthesis-degradation.ent-kaurene_synthase'	9.16E-14
snp_01_7687212	pgc1.2	LOC_Os01g13740	'27.3.20'_'RNA.regulation_of_transcription.G2-like_transcription_factor_family,_GARP'	snp_04_31024760	pgc4.4	LOC_Os04g52220	'35.2'_'not_assigned.unknown'	1.02E-13
snp_01_7687212	pgc1.2	LOC_Os01g13740	'27.3.20'_'RNA.regulation_of_transcription.G2-like_transcription_factor_family,_GARP'	snp_04_31027329	pgc4.4	LOC_Os04g52210	'17.6.1.2'_'hormone_metabolism.gibberelin.synthesis-degradation.ent-kaurene_synthase'	1.78E-13
snp_01_7687212	pgc1.2	LOC_Os01g13740	'27.3.20'_'RNA.regulation_of_transcription.G2-like_transcription_factor_family,_GARP'	snp_04_31005695	pgc4.4	LOC_Os04g52180	'35.2'_'not_assigned.unknown'	2.29E-13
snp_01_7687212	pgc1.2	LOC_Os01g13740	'27.3.20'_'RNA.regulation_of_transcription.G2-like_transcription_factor_family,_GARP'	snp_04_31033053	pgc4.4	LOC_Os04g52240	'17.6.1.2'_'hormone_metabolism.gibberelin.synthesis-degradation.ent-kaurene_synthase'	3.73E-13
snp_01_7687212	pgc1.2	LOC_Os01g13740	'27.3.20'_'RNA.regulation_of_transcription.G2-like_transcription_factor_family,_GARP'	snp_03_6504253	pgc3.3	LOC_Os03g12340	'35.2'_'not_assigned.unknown'	1.06E-12
snp_01_7687212	pgc1.2	LOC_Os01g13740	'27.3.20'_'RNA.regulation_of_transcription.G2-like_transcription_factor_family,_GARP'	snp_03_6536294	pgc3.3	LOC_Os03g12370	'27.3.23'_'RNA.regulation_of_transcription.HSF,Heat-shock_transcription_factor_family'	2.77E-12
snp_01_7687212	pgc1.2	LOC_Os01g13740	'27.3.20'_'RNA.regulation_of_transcription.G2-like_transcription_factor_family,_GARP'	snp_03_6509240	pgc3.3	LOC_Os03g12330	'35.2'_'not_assigned.unknown'	2.9E-12
snp_01_7687212	pgc1.2	LOC_Os01g13740	'27.3.20'_'RNA.regulation_of_transcription.G2-like_transcription_factor_family,_GARP'	snp_04_30942194	pgc4.4	LOC_Os04g52090	'35.2'_'not_assigned.unknown'	8.65E-12
snp_01_7687212	pgc1.2	LOC_Os01g13740	'27.3.20'_'RNA.regulation_of_transcription.G2-like_transcription_factor_family,_GARP'	snp_03_6660602	pgc3.3	LOC_Os03g12570	'27.3.46'_'RNA.regulation_of_transcription.DNA_methyltransferases'	9.38E-12
snp_01_7687212	pgc1.2	LOC_Os01g13740	'27.3.20'_'RNA.regulation_of_transcription.G2-like_transcription_factor_family,_GARP'	snp_03_6619595	pgc3.3	s03g12500-LOC_Os03g	NA	1.5E-11
snp_01_7687212	pgc1.2	LOC_Os01g13740	'27.3.20'_'RNA.regulation_of_transcription.G2-like_transcription_factor_family,_GARP'	snp_04_30942215	pgc4.4	LOC_Os04g52090	'35.2'_'not_assigned.unknown'	1.85E-11
snp_01_7687212	pgc1.2	LOC_Os01g13740	'27.3.20'_'RNA.regulation_of_transcription.G2-like_transcription_factor_family,_GARP'	snp_04_31032653	pgc4.4	LOC_Os04g52240	'17.6.1.2'_'hormone_metabolism.gibberelin.synthesis-degradation.ent-kaurene_synthase'	5.4E-11
snp_01_7687212	pgc1.2	LOC_Os01g13740	'27.3.20'_'RNA.regulation_of_transcription.G2-like_transcription_factor_family,_GARP'	snp_03_6471552	pgc3.3	LOC_Os03g12300	'35.2'_'not_assigned.unknown'	4.83E-10
snp_01_7687212	pgc1.2	LOC_Os01g13740	'27.3.20'_'RNA.regulation_of_transcription.G2-like_transcription_factor_family,_GARP'	snp_03_6461443	pgc3.3	LOC_Os03g12280	'35.2'_'not_assigned.unknown'	1.07E-09
snp_01_7687212	pgc1.2	LOC_Os01g13740	'27.3.20'_'RNA.regulation_of_transcription.G2-like_transcription_factor_family,_GARP'	snp_06_30974876	pgc6.8	LOC_Os06g51200	'35.2'_'not_assigned.unknown'	1.01E-07
snp_01_34329540	pgc1.9	LOC_Os01g59360	'30.3'_'signalling.calcium'	snp_07_22442767	pgc7.8	LOC_Os07g37480	'35.2'_'not_assigned.unknown'	6.87E-09
snp_01_34329540	pgc1.9	LOC_Os01g59360	'30.3'_'signalling.calcium'	snp_07_22442796	pgc7.8	LOC_Os07g37480	'35.2'_'not_assigned.unknown'	2.46E-08
snp_01_34329540	pgc1.9	LOC_Os01g59360	'30.3'_'signalling.calcium'	snp_07_22443624	pgc7.8	LOC_Os07g37480	'35.2'_'not_assigned.unknown'	2.83E-08
snp_01_34329540	pgc1.9	LOC_Os01g59360	'30.3'_'signalling.calcium'	snp_07_22442623	pgc7.8	LOC_Os07g37480	'35.2'_'not_assigned.unknown'	2.87E-08
snp_01_34329540	pgc1.9	LOC_Os01g59360	'30.3'_'signalling.calcium'	snp_07_22484898	pgc7.8	LOC_Os07g37540	'35.2'_'not_assigned.unknown'	2.98E-08
snp_01_34329540	pgc1.9	LOC_Os01g59360	'30.3'_'signalling.calcium'	snp_07_22486583	pgc7.8	LOC_Os07g37550	'1.1.1.1'_'PS.lightreaction.photosystem_II.LHC-II'	3.06E-08
snp_01_34329540	pgc1.9	LOC_Os01g59360	'30.3'_'signalling.calcium'	snp_07_22443025	pgc7.8	LOC_Os07g37480	'35.2'_'not_assigned.unknown'	3.08E-08
snp_01_34329540	pgc1.9	LOC_Os01g59360	'30.3'_'signalling.calcium'	snp_07_22443235	pgc7.8	LOC_Os07g37480	'35.2'_'not_assigned.unknown'	9.86E-08
snp_03_26840782	pgc3.7	s03g47470-LOC_Os03	NA	snp_07_22486583	pgc7.8	LOC_Os07g37550	'1.1.1.1'_'PS.lightreaction.photosystem_II.LHC-II'	4.63E-11
snp_03_26840782	pgc3.7	s03g47470-LOC_Os03	NA	snp_07_22442767	pgc7.8	LOC_Os07g37480	'35.2'_'not_assigned.unknown'	7.07E-11
snp_03_26840782	pgc3.7	s03g47470-LOC_Os03	NA	snp_07_22442796	pgc7.8	LOC_Os07g37480	'35.2'_'not_assigned.unknown'	1.34E-10
snp_03_26840782	pgc3.7	s03g47470-LOC_Os03	NA	snp_07_22443235	pgc7.8	LOC_Os07g37480	'35.2'_'not_assigned.unknown'	1.4E-10
snp_03_26840782	pgc3.7	s03g47470-LOC_Os03	NA	snp_07_22443624	pgc7.8	LOC_Os07g37480	'35.2'_'not_assigned.unknown'	1.51E-10
snp_03_26840782	pgc3.7	s03g47470-LOC_Os03	NA	snp_07_22442623	pgc7.8	LOC_Os07g37480	'35.2'_'not_assigned.unknown'	1.54E-10
snp_03_26840782	pgc3.7	s03g47470-LOC_Os03	NA	snp_07_22443025	pgc7.8	LOC_Os07g37480	'35.2'_'not_assigned.unknown'	1.57E-10
snp_03_26840782	pgc3.7	s03g47470-LOC_Os03	NA	snp_07_22484898	pgc7.8	LOC_Os07g37540	'35.2'_'not_assigned.unknown'	1.83E-10

Region 1				Region 2				p-value of interaction
SNP1	QTL1	Locus ID	Functional categories	SNP2	QTL2	Locus ID	Functional categories	
snp_03_26840782	pgc3.7	s03g47470-LOC_Os03	NA	snp_07_22442651	pgc7.8	LOC_Os07g37480	'35.2'_'not_assigned.unknown'	4.02E-08
snp_04_31033053	pgc4.4	LOC_Os04g52240	'17.6.1.2'_'hormone_metabolism.gibberelin.synthesis-degradation.ent-kaurene_synthase'	snp_05_29310238	pgc5.8	LOC_Os05g51080	'35.2'_'not_assigned.unknown'	7.88E-09
snp_04_31033053	pgc4.4	LOC_Os04g52240	'17.6.1.2'_'hormone_metabolism.gibberelin.synthesis-degradation.ent-kaurene_synthase'	snp_05_29341831	pgc5.8	LOC_Os05g51150	'27.3.85'_'RNA.regulation_of_transcription.sigma_like_plant'	9.2E-09
snp_04_31033053	pgc4.4	LOC_Os04g52240	'17.6.1.2'_'hormone_metabolism.gibberelin.synthesis-degradation.ent-kaurene_synthase'	snp_05_29309709	pgc5.8	LOC_Os05g51080	'35.2'_'not_assigned.unknown'	2.19E-08
snp_04_31035500	pgc4.4	LOC_Os04g52230	'17.6.1.2'_'hormone_metabolism.gibberelin.synthesis-degradation.ent-kaurene_synthase'	snp_05_29310238	pgc5.8	LOC_Os05g51080	'35.2'_'not_assigned.unknown'	3.3E-08
snp_04_31035500	pgc4.4	LOC_Os04g52230	'17.6.1.2'_'hormone_metabolism.gibberelin.synthesis-degradation.ent-kaurene_synthase'	snp_05_29341831	pgc5.8	LOC_Os05g51150	'27.3.85'_'RNA.regulation_of_transcription.sigma_like_plant'	3.82E-08
snp_04_31212801	pgc4.4	LOC_Os04g52479	'35.2'_'not_assigned.unknown'	snp_06_30974876	pgc6.8	LOC_Os06g51200	'35.2'_'not_assigned.unknown'	4.08E-08
snp_04_31033053	pgc4.4	LOC_Os04g52240	'17.6.1.2'_'hormone_metabolism.gibberelin.synthesis-degradation.ent-kaurene_synthase'	snp_05_29337175	pgc5.8	LOC_Os05g51140	'35.2'_'not_assigned.unknown'	7.38E-08
snp_04_31025026	pgc4.4	LOC_Os04g52220	'35.2'_'not_assigned.unknown'	snp_05_29310238	pgc5.8	LOC_Os05g51080	'35.2'_'not_assigned.unknown'	7.8E-08
snp_04_31035500	pgc4.4	LOC_Os04g52230	'17.6.1.2'_'hormone_metabolism.gibberelin.synthesis-degradation.ent-kaurene_synthase'	snp_05_29309709	pgc5.8	LOC_Os05g51080	'35.2'_'not_assigned.unknown'	8.15E-08
snp_04_31035595	pgc4.4	LOC_Os04g52230	'17.6.1.2'_'hormone_metabolism.gibberelin.synthesis-degradation.ent-kaurene_synthase'	snp_05_29310238	pgc5.8	LOC_Os05g51080	'35.2'_'not_assigned.unknown'	8.67E-08
snp_04_31025026	pgc4.4	LOC_Os04g52220	'35.2'_'not_assigned.unknown'	snp_05_29341831	pgc5.8	LOC_Os05g51150	'27.3.85'_'RNA.regulation_of_transcription.sigma_like_plant'	8.96E-08
snp_04_31035595	pgc4.4	LOC_Os04g52230	'17.6.1.2'_'hormone_metabolism.gibberelin.synthesis-degradation.ent-kaurene_synthase'	snp_05_29341831	pgc5.8	LOC_Os05g51150	'27.3.85'_'RNA.regulation_of_transcription.sigma_like_plant'	9.94E-08
snp_04_31030699	pgc4.4	LOC_Os04g52230	'17.6.1.2'_'hormone_metabolism.gibberelin.synthesis-degradation.ent-kaurene_synthase'	snp_05_29310238	pgc5.8	LOC_Os05g51080	'35.2'_'not_assigned.unknown'	1.45E-07
snp_04_31030699	pgc4.4	LOC_Os04g52230	'17.6.1.2'_'hormone_metabolism.gibberelin.synthesis-degradation.ent-kaurene_synthase'	snp_05_29341831	pgc5.8	LOC_Os05g51150	'27.3.85'_'RNA.regulation_of_transcription.sigma_like_plant'	1.67E-07
snp_05_5376161	pgc5.2	s05g09520-LOC_Os05	NA	snp_06_30974876	pgc6.8	LOC_Os06g51200	'35.2'_'not_assigned.unknown'	3.42E-08
snp_05_29341831	pgc5.8	LOC_Os05g51150	'27.3.85'_'RNA.regulation_of_transcription.sigma_like_plant'	snp_06_1259614	pgc6.1	LOC_Os06g03310	'35.2'_'not_assigned.unknown'	5.79E-08
snp_05_29341831	pgc5.8	LOC_Os05g51150	'27.3.85'_'RNA.regulation_of_transcription.sigma_like_plant'	snp_06_1259666	pgc6.1	LOC_Os06g03310	'35.2'_'not_assigned.unknown'	6.41E-08
snp_05_29337175	pgc5.8	LOC_Os05g51140	'35.2'_'not_assigned.unknown'	snp_06_1259614	pgc6.1	LOC_Os06g03310	'35.2'_'not_assigned.unknown'	7.42E-08
snp_05_29337175	pgc5.8	LOC_Os05g51140	'35.2'_'not_assigned.unknown'	snp_06_1259666	pgc6.1	LOC_Os06g03310	'35.2'_'not_assigned.unknown'	8.2E-08
snp_05_29341831	pgc5.8	LOC_Os05g51150	'27.3.85'_'RNA.regulation_of_transcription.sigma_like_plant'	snp_06_1246170	pgc6.1	LOC_Os06g03280	'35.2'_'not_assigned.unknown'	1.19E-07
snp_05_29341831	pgc5.8	LOC_Os05g51150	'27.3.85'_'RNA.regulation_of_transcription.sigma_like_plant'	snp_06_1258271	pgc6.1	LOC_Os06g03310	'35.2'_'not_assigned.unknown'	1.32E-07
snp_05_29341831	pgc5.8	LOC_Os05g51150	'27.3.85'_'RNA.regulation_of_transcription.sigma_like_plant'	snp_06_1261311	pgc6.1	LOC_Os06g03310	'35.2'_'not_assigned.unknown'	1.33E-07
snp_05_29337175	pgc5.8	LOC_Os05g51140	'35.2'_'not_assigned.unknown'	snp_06_1246170	pgc6.1	LOC_Os06g03280	'35.2'_'not_assigned.unknown'	1.78E-07
snp_06_1347924	pgc6.1	LOC_Os06g03500	'20.1'_'stress.biotic'	snp_07_22443235	pgc7.8	LOC_Os07g37480	'35.2'_'not_assigned.unknown'	9.29E-09
snp_06_1347924	pgc6.1	LOC_Os06g03500	'20.1'_'stress.biotic'	snp_07_22442796	pgc7.8	LOC_Os07g37480	'35.2'_'not_assigned.unknown'	1.16E-07
snp_06_1347924	pgc6.1	LOC_Os06g03500	'20.1'_'stress.biotic'	snp_07_22443025	pgc7.8	LOC_Os07g37480	'35.2'_'not_assigned.unknown'	1.26E-07
snp_06_1347924	pgc6.1	LOC_Os06g03500	'20.1'_'stress.biotic'	snp_07_22443624	pgc7.8	LOC_Os07g37480	'35.2'_'not_assigned.unknown'	1.35E-07
snp_06_1347924	pgc6.1	LOC_Os06g03500	'20.1'_'stress.biotic'	snp_07_22442623	pgc7.8	LOC_Os07g37480	'35.2'_'not_assigned.unknown'	1.36E-07
snp_06_1347924	pgc6.1	LOC_Os06g03500	'20.1'_'stress.biotic'	snp_07_22484898	pgc7.8	LOC_Os07g37540	'35.2'_'not_assigned.unknown'	1.44E-07
snp_06_1347924	pgc6.1	LOC_Os06g03500	'20.1'_'stress.biotic'	snp_07_22486583	pgc7.8	LOC_Os07g37550	'1.1.1.1'_'PS.lightreaction.photosystem_II.LHC-II'	1.47E-07
snp_06_1347924	pgc6.1	LOC_Os06g03500	'20.1'_'stress.biotic'	snp_07_22442767	pgc7.8	LOC_Os07g37480	'35.2'_'not_assigned.unknown'	0.00000017

Region 1				Region 2				p-value of interaction
SNP1	QTL1	Locus ID	Functional categories	SNP2	QTL2	Locus ID	Functional categories	
snp_06_30826213	pgc6.8	LOC_Os06g50930	'33.99'_'development.unspecified'	snp_07_22486583	pgc7.8	LOC_Os07g37550	'1.1.1.1'_'PS.lightreaction.photosystem_II.LHC-II'	3.19E-11
snp_06_30826303	pgc6.8	LOC_Os06g50930	'33.99'_'development.unspecified'	snp_07_22486583	pgc7.8	LOC_Os07g37550	'1.1.1.1'_'PS.lightreaction.photosystem_II.LHC-II'	4.85E-11
snp_06_30490737	pgc6.8	LOC_Os06g50370	'29.5.11.4.2'_'protein.degradation.ubiquitin.E3.RING'	snp_07_22486583	pgc7.8	LOC_Os07g37550	'1.1.1.1'_'PS.lightreaction.photosystem_II.LHC-II'	7.17E-11
snp_06_30826231	pgc6.8	LOC_Os06g50930	'33.99'_'development.unspecified'	snp_07_22486583	pgc7.8	LOC_Os07g37550	'1.1.1.1'_'PS.lightreaction.photosystem_II.LHC-II'	9.22E-11
snp_06_30826213	pgc6.8	LOC_Os06g50930	'33.99'_'development.unspecified'	snp_07_22442623	pgc7.8	LOC_Os07g37480	'35.2'_'not_assigned.unknown'	9.83E-11
snp_06_30826213	pgc6.8	LOC_Os06g50930	'33.99'_'development.unspecified'	snp_07_22442767	pgc7.8	LOC_Os07g37480	'35.2'_'not_assigned.unknown'	1.18E-10
snp_06_30826303	pgc6.8	LOC_Os06g50930	'33.99'_'development.unspecified'	snp_07_22442623	pgc7.8	LOC_Os07g37480	'35.2'_'not_assigned.unknown'	1.49E-10
snp_06_30974876	pgc6.8	LOC_Os06g51200	'35.2'_'not_assigned.unknown'	snp_07_22442623	pgc7.8	LOC_Os07g37480	'35.2'_'not_assigned.unknown'	1.62E-10
snp_06_30826213	pgc6.8	LOC_Os06g50930	'33.99'_'development.unspecified'	snp_07_22442796	pgc7.8	LOC_Os07g37480	'35.2'_'not_assigned.unknown'	1.81E-10
snp_06_30826213	pgc6.8	LOC_Os06g50930	'33.99'_'development.unspecified'	snp_07_22443025	pgc7.8	LOC_Os07g37480	'35.2'_'not_assigned.unknown'	2.03E-10
snp_06_30488787	pgc6.8	LOC_Os06g50360	'29.1.30'_'protein.aa_activation.pseudouridylate_synthase'	snp_07_22486583	pgc7.8	LOC_Os07g37550	'1.1.1.1'_'PS.lightreaction.photosystem_II.LHC-II'	2.06E-10
snp_06_30826213	pgc6.8	LOC_Os06g50930	'33.99'_'development.unspecified'	snp_07_22443624	pgc7.8	LOC_Os07g37480	'35.2'_'not_assigned.unknown'	2.17E-10
snp_06_30826213	pgc6.8	LOC_Os06g50930	'33.99'_'development.unspecified'	snp_07_22484898	pgc7.8	LOC_Os07g37540	'35.2'_'not_assigned.unknown'	2.3E-10
snp_06_30490737	pgc6.8	LOC_Os06g50370	'29.5.11.4.2'_'protein.degradation.ubiquitin.E3.RING'	snp_07_22442623	pgc7.8	LOC_Os07g37480	'35.2'_'not_assigned.unknown'	2.42E-10
snp_06_30826231	pgc6.8	LOC_Os06g50930	'33.99'_'development.unspecified'	snp_07_22442623	pgc7.8	LOC_Os07g37480	'35.2'_'not_assigned.unknown'	2.71E-10
snp_06_30826303	pgc6.8	LOC_Os06g50930	'33.99'_'development.unspecified'	snp_07_22442796	pgc7.8	LOC_Os07g37480	'35.2'_'not_assigned.unknown'	2.78E-10
snp_06_30436367	pgc6.8	LOC_Os06g50270	'35.2'_'not_assigned.unknown'	snp_07_22486583	pgc7.8	LOC_Os07g37550	'1.1.1.1'_'PS.lightreaction.photosystem_II.LHC-II'	2.82E-10
snp_06_30826303	pgc6.8	LOC_Os06g50930	'33.99'_'development.unspecified'	snp_07_22442767	pgc7.8	LOC_Os07g37480	'35.2'_'not_assigned.unknown'	2.93E-10
snp_06_30826303	pgc6.8	LOC_Os06g50930	'33.99'_'development.unspecified'	snp_07_22443025	pgc7.8	LOC_Os07g37480	'35.2'_'not_assigned.unknown'	3.09E-10
snp_06_30826231	pgc6.8	LOC_Os06g50930	'33.99'_'development.unspecified'	snp_07_22442767	pgc7.8	LOC_Os07g37480	'35.2'_'not_assigned.unknown'	3.24E-10
snp_06_30974876	pgc6.8	LOC_Os06g51200	'35.2'_'not_assigned.unknown'	snp_07_22442796	pgc7.8	LOC_Os07g37480	'35.2'_'not_assigned.unknown'	3.28E-10
snp_06_30826303	pgc6.8	LOC_Os06g50930	'33.99'_'development.unspecified'	snp_07_22443624	pgc7.8	LOC_Os07g37480	'35.2'_'not_assigned.unknown'	3.32E-10
snp_06_30826303	pgc6.8	LOC_Os06g50930	'33.99'_'development.unspecified'	snp_07_22484898	pgc7.8	LOC_Os07g37540	'35.2'_'not_assigned.unknown'	3.52E-10
snp_06_30974876	pgc6.8	LOC_Os06g51200	'35.2'_'not_assigned.unknown'	snp_07_22443025	pgc7.8	LOC_Os07g37480	'35.2'_'not_assigned.unknown'	3.73E-10
snp_06_30974876	pgc6.8	LOC_Os06g51200	'35.2'_'not_assigned.unknown'	snp_07_22442767	pgc7.8	LOC_Os07g37480	'35.2'_'not_assigned.unknown'	3.8E-10
snp_06_30974876	pgc6.8	LOC_Os06g51200	'35.2'_'not_assigned.unknown'	snp_07_22443624	pgc7.8	LOC_Os07g37480	'35.2'_'not_assigned.unknown'	3.92E-10
snp_06_30974876	pgc6.8	LOC_Os06g51200	'35.2'_'not_assigned.unknown'	snp_07_22484898	pgc7.8	LOC_Os07g37540	'35.2'_'not_assigned.unknown'	4.06E-10
snp_06_30974876	pgc6.8	LOC_Os06g51200	'35.2'_'not_assigned.unknown'	snp_07_22486583	pgc7.8	LOC_Os07g37550	'1.1.1.1'_'PS.lightreaction.photosystem_II.LHC-II'	4.29E-10
snp_06_30826231	pgc6.8	LOC_Os06g50930	'33.99'_'development.unspecified'	snp_07_22442796	pgc7.8	LOC_Os07g37480	'35.2'_'not_assigned.unknown'	4.9E-10
snp_06_30490737	pgc6.8	LOC_Os06g50370	'29.5.11.4.2'_'protein.degradation.ubiquitin.E3.RING'	snp_07_22442796	pgc7.8	LOC_Os07g37480	'35.2'_'not_assigned.unknown'	4.96E-10
snp_06_30826231	pgc6.8	LOC_Os06g50930	'33.99'_'development.unspecified'	snp_07_22443025	pgc7.8	LOC_Os07g37480	'35.2'_'not_assigned.unknown'	5.42E-10
snp_06_30490737	pgc6.8	LOC_Os06g50370	'29.5.11.4.2'_'protein.degradation.ubiquitin.E3.RING'	snp_07_22443025	pgc7.8	LOC_Os07g37480	'35.2'_'not_assigned.unknown'	5.48E-10
snp_06_30490737	pgc6.8	LOC_Os06g50370	'29.5.11.4.2'_'protein.degradation.ubiquitin.E3.RING'	snp_07_22442767	pgc7.8	LOC_Os07g37480	'35.2'_'not_assigned.unknown'	5.56E-10
snp_06_30826231	pgc6.8	LOC_Os06g50930	'33.99'_'development.unspecified'	snp_07_22443624	pgc7.8	LOC_Os07g37480	'35.2'_'not_assigned.unknown'	5.83E-10
snp_06_30490737	pgc6.8	LOC_Os06g50370	'29.5.11.4.2'_'protein.degradation.ubiquitin.E3.RING'	snp_07_22443624	pgc7.8	LOC_Os07g37480	'35.2'_'not_assigned.unknown'	5.91E-10
snp_06_30826213	pgc6.8	LOC_Os06g50930	'33.99'_'development.unspecified'	snp_07_22443235	pgc7.8	LOC_Os07g37480	'35.2'_'not_assigned.unknown'	6.02E-10
snp_06_30490737	pgc6.8	LOC_Os06g50370	'29.5.11.4.2'_'protein.degradation.ubiquitin.E3.RING'	snp_07_22484898	pgc7.8	LOC_Os07g37540	'35.2'_'not_assigned.unknown'	6.13E-10

Region 1				Region 2				p-value of interaction
SNP1	QTL1	Locus ID	Functional categories	SNP2	QTL2	Locus ID	Functional categories	
snp_06_30826231	pgc6.8	LOC_Os06g50930	'33.99'_'development.unspecified'	snp_07_22484898	pgc7.8	LOC_Os07g37540	'35.2'_'not_assigned.unknown'	6.21E-10
snp_06_30974876	pgc6.8	LOC_Os06g51200	'35.2'_'not_assigned.unknown'	snp_07_22443235	pgc7.8	LOC_Os07g37480	'35.2'_'not_assigned.unknown'	6.45E-10
snp_06_30488787	pgc6.8	LOC_Os06g50360	'29.1.30'_'protein.aa_activation.pseudouridylate_synthase'	snp_07_22442623	pgc7.8	LOC_Os07g37480	'35.2'_'not_assigned.unknown'	6.81E-10
snp_06_30487804	pgc6.8	LOC_Os06g50360	'29.1.30'_'protein.aa_activation.pseudouridylate_synthase'	snp_07_22442623	pgc7.8	LOC_Os07g37480	'35.2'_'not_assigned.unknown'	8E-10
snp_06_30487804	pgc6.8	LOC_Os06g50360	'29.1.30'_'protein.aa_activation.pseudouridylate_synthase'	snp_07_22486583	pgc7.8	LOC_Os07g37550	'1.1.1.1'_'PS.lightreaction.photosystem_II.LHC-II'	8.34E-10
snp_06_30826303	pgc6.8	LOC_Os06g50930	'33.99'_'development.unspecified'	snp_07_22443235	pgc7.8	LOC_Os07g37480	'35.2'_'not_assigned.unknown'	9.08E-10
snp_06_30436367	pgc6.8	LOC_Os06g50270	'35.2'_'not_assigned.unknown'	snp_07_22442623	pgc7.8	LOC_Os07g37480	'35.2'_'not_assigned.unknown'	9.47E-10
snp_06_30469303	pgc6.8	LOC_Os06g50330	'33.99'_'development.unspecified'	snp_07_22486583	pgc7.8	LOC_Os07g37550	'1.1.1.1'_'PS.lightreaction.photosystem_II.LHC-II'	1.32E-09
snp_06_30473682	pgc6.8	LOC_Os06g50340	'30.2.11'_'signalling.receptor_kinases.leucine_rich_repeat_XI'	snp_07_22486583	pgc7.8	LOC_Os07g37550	'1.1.1.1'_'PS.lightreaction.photosystem_II.LHC-II'	1.37E-09
snp_06_30488787	pgc6.8	LOC_Os06g50360	'29.1.30'_'protein.aa_activation.pseudouridylate_synthase'	snp_07_22442796	pgc7.8	LOC_Os07g37480	'35.2'_'not_assigned.unknown'	1.39E-09
snp_06_30826231	pgc6.8	LOC_Os06g50930	'33.99'_'development.unspecified'	snp_07_22443235	pgc7.8	LOC_Os07g37480	'35.2'_'not_assigned.unknown'	1.5E-09
snp_06_30488787	pgc6.8	LOC_Os06g50360	'29.1.30'_'protein.aa_activation.pseudouridylate_synthase'	snp_07_22443025	pgc7.8	LOC_Os07g37480	'35.2'_'not_assigned.unknown'	1.52E-09
snp_06_30488787	pgc6.8	LOC_Os06g50360	'29.1.30'_'protein.aa_activation.pseudouridylate_synthase'	snp_07_22442767	pgc7.8	LOC_Os07g37480	'35.2'_'not_assigned.unknown'	1.54E-09
snp_06_30488787	pgc6.8	LOC_Os06g50360	'29.1.30'_'protein.aa_activation.pseudouridylate_synthase'	snp_07_22443624	pgc7.8	LOC_Os07g37480	'35.2'_'not_assigned.unknown'	1.64E-09
snp_06_30487804	pgc6.8	LOC_Os06g50360	'29.1.30'_'protein.aa_activation.pseudouridylate_synthase'	snp_07_22442796	pgc7.8	LOC_Os07g37480	'35.2'_'not_assigned.unknown'	1.67E-09
snp_06_30488787	pgc6.8	LOC_Os06g50360	'29.1.30'_'protein.aa_activation.pseudouridylate_synthase'	snp_07_22484898	pgc7.8	LOC_Os07g37540	'35.2'_'not_assigned.unknown'	1.7E-09
snp_06_30490737	pgc6.8	LOC_Os06g50370	'29.5.11.4.2'_'protein.degradation.ubiquitin.E3.RING'	snp_07_22443235	pgc7.8	LOC_Os07g37480	'35.2'_'not_assigned.unknown'	1.77E-09
snp_06_30487804	pgc6.8	LOC_Os06g50360	'29.1.30'_'protein.aa_activation.pseudouridylate_synthase'	snp_07_22442767	pgc7.8	LOC_Os07g37480	'35.2'_'not_assigned.unknown'	1.79E-09
snp_06_30487804	pgc6.8	LOC_Os06g50360	'29.1.30'_'protein.aa_activation.pseudouridylate_synthase'	snp_07_22443025	pgc7.8	LOC_Os07g37480	'35.2'_'not_assigned.unknown'	1.83E-09
snp_06_30487804	pgc6.8	LOC_Os06g50360	'29.1.30'_'protein.aa_activation.pseudouridylate_synthase'	snp_07_22443624	pgc7.8	LOC_Os07g37480	'35.2'_'not_assigned.unknown'	2E-09
snp_06_30487804	pgc6.8	LOC_Os06g50360	'29.1.30'_'protein.aa_activation.pseudouridylate_synthase'	snp_07_22484898	pgc7.8	LOC_Os07g37540	'35.2'_'not_assigned.unknown'	2.06E-09
snp_06_30436367	pgc6.8	LOC_Os06g50270	'35.2'_'not_assigned.unknown'	snp_07_22442796	pgc7.8	LOC_Os07g37480	'35.2'_'not_assigned.unknown'	2.07E-09
snp_06_30436367	pgc6.8	LOC_Os06g50270	'35.2'_'not_assigned.unknown'	snp_07_22442767	pgc7.8	LOC_Os07g37480	'35.2'_'not_assigned.unknown'	2.16E-09
snp_06_30451292	pgc6.8	LOC_Os06g50300	'20.2.1'_'stress.abiotic.heat'	snp_07_22486583	pgc7.8	LOC_Os07g37550	'1.1.1.1'_'PS.lightreaction.photosystem_II.LHC-II'	2.22E-09
snp_06_30436367	pgc6.8	LOC_Os06g50270	'35.2'_'not_assigned.unknown'	snp_07_22443025	pgc7.8	LOC_Os07g37480	'35.2'_'not_assigned.unknown'	2.34E-09
snp_06_30436367	pgc6.8	LOC_Os06g50270	'35.2'_'not_assigned.unknown'	snp_07_22443624	pgc7.8	LOC_Os07g37480	'35.2'_'not_assigned.unknown'	2.45E-09
snp_06_30436367	pgc6.8	LOC_Os06g50270	'35.2'_'not_assigned.unknown'	snp_07_22484898	pgc7.8	LOC_Os07g37540	'35.2'_'not_assigned.unknown'	2.56E-09
snp_06_30469303	pgc6.8	LOC_Os06g50330	'33.99'_'development.unspecified'	snp_07_22442623	pgc7.8	LOC_Os07g37480	'35.2'_'not_assigned.unknown'	4E-09
snp_06_30473682	pgc6.8	LOC_Os06g50340	'30.2.11'_'signalling.receptor_kinases.leucine_rich_repeat_XI'	snp_07_22442623	pgc7.8	LOC_Os07g37480	'35.2'_'not_assigned.unknown'	4.1E-09
snp_06_30488787	pgc6.8	LOC_Os06g50360	'29.1.30'_'protein.aa_activation.pseudouridylate_synthase'	snp_07_22443235	pgc7.8	LOC_Os07g37480	'35.2'_'not_assigned.unknown'	4.5E-09

Region 1				Region 2				p-value of interaction
SNP1	QTL1	Locus ID	Functional categories	SNP2	QTL2	Locus ID	Functional categories	
snp_06_30487804	pgc6.8	LOC_Os06g50360	'29.1.30'_'protein.aa_activation.pseudouridylate_synthase'	snp_07_22443235	pgc7.8	LOC_Os07g37480	'35.2'_'not_assigned.unknown'	5E-09
snp_06_30436367	pgc6.8	LOC_Os06g50270	'35.2'_'not_assigned.unknown'	snp_07_22443235	pgc7.8	LOC_Os07g37480	'35.2'_'not_assigned.unknown'	5.15E-09
snp_06_30436072	pgc6.8	LOC_Os06g50270	'35.2'_'not_assigned.unknown'	snp_07_22486583	pgc7.8	LOC_Os07g37550	'1.1.1.1'_'PS.lightreaction.photosystem_II.LHC-II'	5.5E-09
snp_06_30451292	pgc6.8	LOC_Os06g50300	'20.2.1'_'stress.abiotic.heat'	snp_07_22442623	pgc7.8	LOC_Os07g37480	'35.2'_'not_assigned.unknown'	6.29E-09
snp_06_30974876	pgc6.8	LOC_Os06g51200	'35.2'_'not_assigned.unknown'	snp_07_22442651	pgc7.8	LOC_Os07g37480	'35.2'_'not_assigned.unknown'	6.67E-09
snp_06_30469303	pgc6.8	LOC_Os06g50330	'33.99'_'development.unspecified'	snp_07_22442796	pgc7.8	LOC_Os07g37480	'35.2'_'not_assigned.unknown'	7.77E-09
snp_06_30473682	pgc6.8	LOC_Os06g50340	'30.2.11'_'signalling.receptor_kinases.leucine_rich_repeat_XI'	snp_07_22442796	pgc7.8	LOC_Os07g37480	'35.2'_'not_assigned.unknown'	8.06E-09
snp_06_30826213	pgc6.8	LOC_Os06g50930	'33.99'_'development.unspecified'	snp_07_22442651	pgc7.8	LOC_Os07g37480	'35.2'_'not_assigned.unknown'	8.3E-09
snp_06_30469303	pgc6.8	LOC_Os06g50330	'33.99'_'development.unspecified'	snp_07_22442767	pgc7.8	LOC_Os07g37480	'35.2'_'not_assigned.unknown'	8.44E-09
snp_06_30469303	pgc6.8	LOC_Os06g50330	'33.99'_'development.unspecified'	snp_07_22443025	pgc7.8	LOC_Os07g37480	'35.2'_'not_assigned.unknown'	8.48E-09
snp_06_30473682	pgc6.8	LOC_Os06g50340	'30.2.11'_'signalling.receptor_kinases.leucine_rich_repeat_XI'	snp_07_22442767	pgc7.8	LOC_Os07g37480	'35.2'_'not_assigned.unknown'	8.75E-09
snp_06_30473682	pgc6.8	LOC_Os06g50340	'30.2.11'_'signalling.receptor_kinases.leucine_rich_repeat_XI'	snp_07_22443025	pgc7.8	LOC_Os07g37480	'35.2'_'not_assigned.unknown'	8.79E-09
snp_06_30469303	pgc6.8	LOC_Os06g50330	'33.99'_'development.unspecified'	snp_07_22443624	pgc7.8	LOC_Os07g37480	'35.2'_'not_assigned.unknown'	9.08E-09
snp_06_30473682	pgc6.8	LOC_Os06g50340	'30.2.11'_'signalling.receptor_kinases.leucine_rich_repeat_XI'	snp_07_22443624	pgc7.8	LOC_Os07g37480	'35.2'_'not_assigned.unknown'	9.41E-09
snp_06_30469303	pgc6.8	LOC_Os06g50330	'33.99'_'development.unspecified'	snp_07_22484898	pgc7.8	LOC_Os07g37540	'35.2'_'not_assigned.unknown'	9.51E-09
snp_06_30473682	pgc6.8	LOC_Os06g50340	'30.2.11'_'signalling.receptor_kinases.leucine_rich_repeat_XI'	snp_07_22484898	pgc7.8	LOC_Os07g37540	'35.2'_'not_assigned.unknown'	9.86E-09
snp_06_30451292	pgc6.8	LOC_Os06g50300	'20.2.1'_'stress.abiotic.heat'	snp_07_22442796	pgc7.8	LOC_Os07g37480	'35.2'_'not_assigned.unknown'	1.26E-08
snp_06_30490737	pgc6.8	LOC_Os06g50370	'29.5.11.4.2'_'protein.degradation.ubiquitin.E3.RING'	snp_07_22442651	pgc7.8	LOC_Os07g37480	'35.2'_'not_assigned.unknown'	1.3E-08
snp_06_30451292	pgc6.8	LOC_Os06g50300	'20.2.1'_'stress.abiotic.heat'	snp_07_22442767	pgc7.8	LOC_Os07g37480	'35.2'_'not_assigned.unknown'	1.34E-08
snp_06_30451292	pgc6.8	LOC_Os06g50300	'20.2.1'_'stress.abiotic.heat'	snp_07_22443025	pgc7.8	LOC_Os07g37480	'35.2'_'not_assigned.unknown'	1.37E-08
snp_06_30451292	pgc6.8	LOC_Os06g50300	'20.2.1'_'stress.abiotic.heat'	snp_07_22443624	pgc7.8	LOC_Os07g37480	'35.2'_'not_assigned.unknown'	1.47E-08
snp_06_30436072	pgc6.8	LOC_Os06g50270	'35.2'_'not_assigned.unknown'	snp_07_22442623	pgc7.8	LOC_Os07g37480	'35.2'_'not_assigned.unknown'	1.52E-08
snp_06_30451292	pgc6.8	LOC_Os06g50300	'20.2.1'_'stress.abiotic.heat'	snp_07_22484898	pgc7.8	LOC_Os07g37540	'35.2'_'not_assigned.unknown'	1.55E-08
snp_06_30826231	pgc6.8	LOC_Os06g50930	'33.99'_'development.unspecified'	snp_07_22442651	pgc7.8	LOC_Os07g37480	'35.2'_'not_assigned.unknown'	1.72E-08
snp_06_30826303	pgc6.8	LOC_Os06g50930	'33.99'_'development.unspecified'	snp_07_22442651	pgc7.8	LOC_Os07g37480	'35.2'_'not_assigned.unknown'	1.84E-08
snp_06_30469303	pgc6.8	LOC_Os06g50330	'33.99'_'development.unspecified'	snp_07_22443235	pgc7.8	LOC_Os07g37480	'35.2'_'not_assigned.unknown'	2.2E-08
snp_06_30473682	pgc6.8	LOC_Os06g50340	'30.2.11'_'signalling.receptor_kinases.leucine_rich_repeat_XI'	snp_07_22443235	pgc7.8	LOC_Os07g37480	'35.2'_'not_assigned.unknown'	2.28E-08
snp_06_30487804	pgc6.8	LOC_Os06g50360	'29.1.30'_'protein.aa_activation.pseudouridylate_synthase'	snp_07_22442651	pgc7.8	LOC_Os07g37480	'35.2'_'not_assigned.unknown'	2.69E-08
snp_06_30436367	pgc6.8	LOC_Os06g50270	'35.2'_'not_assigned.unknown'	snp_07_22442651	pgc7.8	LOC_Os07g37480	'35.2'_'not_assigned.unknown'	2.89E-08
snp_06_30436072	pgc6.8	LOC_Os06g50270	'35.2'_'not_assigned.unknown'	snp_07_22442796	pgc7.8	LOC_Os07g37480	'35.2'_'not_assigned.unknown'	2.91E-08
snp_06_30451292	pgc6.8	LOC_Os06g50300	'20.2.1'_'stress.abiotic.heat'	snp_07_22443235	pgc7.8	LOC_Os07g37480	'35.2'_'not_assigned.unknown'	3.07E-08
snp_06_30436072	pgc6.8	LOC_Os06g50270	'35.2'_'not_assigned.unknown'	snp_07_22442767	pgc7.8	LOC_Os07g37480	'35.2'_'not_assigned.unknown'	3.13E-08
snp_06_30436072	pgc6.8	LOC_Os06g50270	'35.2'_'not_assigned.unknown'	snp_07_22443025	pgc7.8	LOC_Os07g37480	'35.2'_'not_assigned.unknown'	3.16E-08
snp_06_30436072	pgc6.8	LOC_Os06g50270	'35.2'_'not_assigned.unknown'	snp_07_22443624	pgc7.8	LOC_Os07g37480	'35.2'_'not_assigned.unknown'	3.37E-08
snp_06_30488787	pgc6.8	LOC_Os06g50360	'29.1.30'_'protein.aa_activation.pseudouridylate_synthase'	snp_07_22442651	pgc7.8	LOC_Os07g37480	'35.2'_'not_assigned.unknown'	3.41E-08
snp_06_30436072	pgc6.8	LOC_Os06g50270	'35.2'_'not_assigned.unknown'	snp_07_22484898	pgc7.8	LOC_Os07g37540	'35.2'_'not_assigned.unknown'	3.55E-08
snp_06_30436072	pgc6.8	LOC_Os06g50270	'35.2'_'not_assigned.unknown'	snp_07_22443235	pgc7.8	LOC_Os07g37480	'35.2'_'not_assigned.unknown'	7.28E-08
snp_06_30451292	pgc6.8	LOC_Os06g50300	'20.2.1'_'stress.abiotic.heat'	snp_07_22442651	pgc7.8	LOC_Os07g37480	'35.2'_'not_assigned.unknown'	1.06E-07
snp_06_30469303	pgc6.8	LOC_Os06g50330	'33.99'_'development.unspecified'	snp_07_22442651	pgc7.8	LOC_Os07g37480	'35.2'_'not_assigned.unknown'	1.18E-07

Region 1				Region 2				p-value of interaction
SNP1	QTL1	Locus ID	Functional categories	SNP2	QTL2	Locus ID	Functional categories	
snp_06_30473682	pgc6.8	LOC_Os06g50340	'30.2.11'_'signalling.receptor_kinases.leucine_rich_repeat_XI'	snp_07_22442651	pgc7.8	LOC_Os07g37480	'35.2'_'not_assigned.unknown'	1.22E-07

Table S4: Coexpressed gene regulatory networks of developing seeds derived from diversity lines containing contrasting chalk haplotypes. The interaction among target QTL containing epistasis genes in Module (M1) and Module (M2).

M1 (darkslateblue)				
**node1**	**Annotation**	**node2**	**Annotation**	**weight**
LOC_Os01g13740(IUNQ30149):G2-like transcription factor family,GARP				
IUNQ30149	LOC_Os01g13740,G2-like transcription factor family,GARP	IUNQ14991	LOC_Os01g13740,G2-like transcription factor family,GARP	0.24
IUNQ50071	LOC_Os01g03720,MYB domain transcription factor family	IUNQ30149	LOC_Os01g13740,G2-like transcription factor family,GARP	0.13
IUNQ49371	LOC_Os04g52240,hormone metabolism.gibberelin.synthesis	IUNQ30149	LOC_Os01g13740,G2-like transcription factor family,GARP	0.17
IUNQ30149	LOC_Os01g13740,G2-like transcription factor family,GARP	IUNQ50210	LOC_Os04g52240,hormone metabolism.gibberelin.synthesis	0.16
IUNQ52649	LOC_Os04g52230,hormone metabolism.gibberelin.synthesis	IUNQ30149	LOC_Os01g13740,G2-like transcription factor family,GARP	0.18
IUNQ30149	LOC_Os01g13740,G2-like transcription factor family,GARP	IUNQ08262	LOC_Os04g52230,hormone metabolism.gibberelin.synthesis	0.17
IUNQ15265	LOC_Os05g51150,RNA pol sigma factor	IUNQ30149	LOC_Os01g13740,G2-like transcription factor family,GARP	0.11
IMULT03545	LOC_Os04g52220,not assigned.unknown	IUNQ30149	LOC_Os01g13740,G2-like transcription factor family,GARP	0.08
IUNQ30149	LOC_Os01g13740,G2-like transcription factor family,GARP	IUNQ14991	LOC_Os01g13740,G2-like transcription factor family,GARP	0.24
LOC_Os01g13740(IUNQ14991):G2-like transcription factor family,GARP				
IUNQ50071	LOC_Os01g03720,MYB domain transcription factor family	IUNQ14991	LOC_Os01g13740,G2-like transcription factor family,GARP	0.05
IUNQ49371	LOC_Os04g52240,hormone metabolism.gibberelin.synthesis	IUNQ14991	LOC_Os01g13740,G2-like transcription factor family,GARP	0.08
IUNQ14991	LOC_Os01g13740,G2-like transcription factor family,GARP	IUNQ50210	LOC_Os04g52240,hormone metabolism.gibberelin.synthesis	0.08
IUNQ52649	LOC_Os04g52230,hormone metabolism.gibberelin.synthesis	IUNQ14991	LOC_Os01g13740,G2-like transcription factor family,GARP	0.08
IUNQ14991	LOC_Os01g13740,G2-like transcription factor family,GARP	IUNQ08262	LOC_Os04g52230,hormone metabolism.gibberelin.synthesis	0.07

node1	Annotation	node2	Annotation	weight
IUNQ15265	LOC_Os05g51150,RNA pol sigma factor	IUNQ14991	LOC_Os01g13740,G2-like transcription factor family,GARP	0.04
LOC_Os01g03720(IUNQ50071):MYB domain transcription factor family				
IUNQ50071	LOC_Os01g03720,MYB domain transcription factor family	IUNQ30149	LOC_Os01g13740,G2-like transcription factor family,GARP	0.13
IUNQ50071	LOC_Os01g03720,MYB domain transcription factor family	IUNQ14991	LOC_Os01g13740,G2-like transcription factor family,GARP	0.05
IUNQ50071	LOC_Os01g03720,MYB domain transcription factor family	IUNQ49371	LOC_Os04g52240,hormone metabolism.gibberelin.synthesis	0.02
IUNQ50071	LOC_Os01g03720,MYB domain transcription factor family	IUNQ52649	LOC_Os04g52230,hormone metabolism.gibberelin.synthesis	0.09
IUNQ50071	LOC_Os01g03720,MYB domain transcription factor family	IUNQ08262	LOC_Os04g52230,hormone metabolism.gibberelin.synthesis	0.05
LOC_Os04g52240(IUNQ49371):hormone metabolism.gibberelin.synthesis				
IUNQ49371	LOC_Os04g52240,hormone metabolism.gibberelin.synthesis	IUNQ30149	LOC_Os01g13740,G2-like transcription factor family,GARP	0.17
IUNQ49371	LOC_Os04g52240,hormone metabolism.gibberelin.synthesis	IUNQ14991	LOC_Os01g13740,G2-like transcription factor family,GARP	0.08
IUNQ50071	LOC_Os01g03720,MYB domain transcription factor family	IUNQ49371	LOC_Os04g52240,hormone metabolism.gibberelin.synthesis	0.02
IUNQ49371	LOC_Os04g52240,hormone metabolism.gibberelin.synthesis	IUNQ50210	LOC_Os04g52240,hormone metabolism.gibberelin.synthesis	0.06
IUNQ52649	LOC_Os04g52230,hormone metabolism.gibberelin.synthesis	IUNQ49371	LOC_Os04g52240,hormone metabolism.gibberelin.synthesis	0.07
IUNQ49371	LOC_Os04g52240,hormone metabolism.gibberelin.synthesis	IUNQ08262	LOC_Os04g52230,hormone metabolism.gibberelin.synthesis	0.04
IUNQ49371	LOC_Os04g52240,hormone metabolism.gibberelin.synthesis	IUNQ15265	LOC_Os05g51150,RNA pol sigma factor	0.03
LOC_Os04g52240(IUNQ50210):hormone metabolism.gibberelin.synthesis				
IUNQ30149	LOC_Os01g13740,G2-like transcription factor family,GARP	IUNQ50210	LOC_Os04g52240,hormone metabolism.gibberelin.synthesis	0.16
IUNQ14991	LOC_Os01g13740,G2-like transcription factor family,GARP	IUNQ50210	LOC_Os04g52240,hormone metabolism.gibberelin.synthesis	0.08

node1	Annotation	node2	Annotation	weight
IUNQ49371	LOC_Os04g52240,hormone metabolism.gibberelin.synthesis	IUNQ50210	LOC_Os04g52240,hormone metabolism.gibberelin.synthesis	0.06
IUNQ52649	LOC_Os04g52230,hormone metabolism.gibberelin.synthesis	IUNQ50210	LOC_Os04g52240,hormone metabolism.gibberelin.synthesis	0.08
IUNQ50210	LOC_Os04g52240,hormone metabolism.gibberelin.synthesis	IUNQ08262	LOC_Os04g52230,hormone metabolism.gibberelin.synthesis	0.04
LOC_Os04g52230(IUNQ52649):hormone metabolism.gibberelin.synthesis				
IUNQ52649	LOC_Os04g52230,hormone metabolism.gibberelin.synthesis	IUNQ30149	LOC_Os01g13740,G2-like transcription factor family,GARP	0.18
IUNQ52649	LOC_Os04g52230,hormone metabolism.gibberelin.synthesis	IUNQ14991	LOC_Os01g13740,G2-like transcription factor family,GARP	0.08
IUNQ50071	LOC_Os01g03720,MYB domain transcription factor family	IUNQ52649	LOC_Os04g52230,hormone metabolism.gibberelin.synthesis	0.09
IUNQ52649	LOC_Os04g52230,hormone metabolism.gibberelin.synthesis	IUNQ49371	LOC_Os04g52240,hormone metabolism.gibberelin.synthesis	0.07
IUNQ52649	LOC_Os04g52230,hormone metabolism.gibberelin.synthesis	IUNQ50210	LOC_Os04g52240,hormone metabolism.gibberelin.synthesis	0.08
IUNQ52649	LOC_Os04g52230,hormone metabolism.gibberelin.synthesis	IUNQ08262	LOC_Os04g52230,hormone metabolism.gibberelin.synthesis	0.11
IUNQ52649	LOC_Os04g52230,hormone metabolism.gibberelin.synthesis	IUNQ15265	LOC_Os05g51150,RNA pol sigma factor	0.04
IMULT03545	LOC_Os04g52220,not assigned.unknown	IUNQ52649	LOC_Os04g52230,hormone metabolism.gibberelin.synthesis	0.03
LOC_Os04g52230(IUNQ08262):hormone metabolism.gibberelin.synthesis				
IUNQ30149	LOC_Os01g13740,G2-like transcription factor family,GARP	IUNQ08262	LOC_Os04g52230,hormone metabolism.gibberelin.synthesis	0.17
IUNQ14991	LOC_Os01g13740,G2-like transcription factor family,GARP	IUNQ08262	LOC_Os04g52230,hormone metabolism.gibberelin.synthesis	0.07
IUNQ50071	LOC_Os01g03720,MYB domain transcription factor family	IUNQ08262	LOC_Os04g52230,hormone metabolism.gibberelin.synthesis	0.05
IUNQ49371	LOC_Os04g52240,hormone metabolism.gibberelin.synthesis	IUNQ08262	LOC_Os04g52230,hormone metabolism.gibberelin.synthesis	0.04
IUNQ50210	LOC_Os04g52240,hormone metabolism.gibberelin.synthesis	IUNQ08262	LOC_Os04g52230,hormone metabolism.gibberelin.synthesis	0.04

node1	Annotation	node2	Annotation	weight
IUNQ52649	LOC_Os04g52230,hormone metabolism.gibberelin.synthesis	IUNQ08262	LOC_Os04g52230,hormone metabolism.gibberelin.synthesis	0.11
IUNQ15265	LOC_Os05g51150,RNA pol sigma factor	IUNQ08262	LOC_Os04g52230,hormone metabolism.gibberelin.synthesis	0.02
LOC_Os04g52220(IMULT03545): not assigned.unknown				
IMULT03545	LOC_Os04g52220,not assigned.unknown	IUNQ30149	LOC_Os01g13740,G2-like transcription factor family,GARP	0.08
IMULT03545	LOC_Os04g52220,not assigned.unknown	IUNQ14991	LOC_Os01g13740,G2-like transcription factor family,GARP	0.03
IMULT03545	LOC_Os04g52220,not assigned.unknown	IUNQ52649	LOC_Os04g52230,hormone metabolism.gibberelin.synthesis	0.03
LOC_Os05g51150(IUNQ15265): RNA pol sigma factor				
IUNQ15265	LOC_Os05g51150,RNA pol sigma factor	IUNQ14991	LOC_Os01g13740,G2-like transcription factor family,GARP	0.04
IUNQ15265	LOC_Os05g51150,RNA pol sigma factor	IUNQ30149	LOC_Os01g13740,G2-like transcription factor family,GARP	0.11
IUNQ49371	LOC_Os04g52240,hormone metabolism.gibberelin.synthesis	IUNQ15265	LOC_Os05g51150,RNA pol sigma factor	0.03
IUNQ52649	LOC_Os04g52230,hormone metabolism.gibberelin.synthesis	IUNQ15265	LOC_Os05g51150,RNA pol sigma factor	0.04
IUNQ15265	LOC_Os05g51150,RNA pol sigma factor	IUNQ08262	LOC_Os04g52230,hormone metabolism.gibberelin.synthesis	0.02
**M2 (purple)**				
**node1**	**Annotation**	**node2**	**Annotation**	**weight**
LOC_Os04g52210(IMULT02870)				
IUNQ15182	LOC_Os01g13740,G2-like transcription factor family,GARP	IMULT02870	LOC_Os04g52210,hormone metabolism.gibberelin.synthesis	0.1352

Table S5: Detail of germplasm used for the study, with their phenotypic values for grain chalkiness and grain size parameters, along with their countries of origin.

Sl no	IRIS ID	GQNC_NO	Designations	GQNC collection	Chalkiness	Grain length	Grain width	Origin country
1	IRIS_313-9533	GQ01705	DUD KUNING::IRGC 44-1	Indica	2.32	7.06	2.37	Indonesia
2	IRIS_313-9705	GQ01709	TAITUNG WOO LI::IRGC 111-1	Indica	23.48	5.5875	2.6325	Taiwan
3	IRIS_313-9706	GQ01710	TAIPEI WOO CO::IRGC 112-1	Indica	17.02	5.205	2.665	Taiwan
4	IRIS_313-9758	GQ01711	I KUNG PAO::IRGC 114-1	Indica	18.66	4.8375	2.88	Taiwan
5	IRIS_313-9708	GQ01712	TSAI YUAN CHON::IRGC 126-1	Indica	7.34	5.1525	2.535	Taiwan
6	IRIS_313-9472	GQ01713	H 6::IRGC 157-1	Indica	10.72	6.015	2.655	Sri Lanka
7	IRIS_313-8400	GQ01715	RIKUTO NORIN MOCHI 20::IRGC 410-1	Tropical japonica	93.31	5.16	2.855	Japan
8	IRIS_313-10129	GQ01721	SAN SHIH TSI::IRGC 1038-1	Indica	30.51	5.2375	2.545	China
9	IRIS_313-9324	GQ01722	CHIH SHEN LI::IRGC 1306-1	Indica	52.29	5.6375	2.5875	China
10	IRIS_313-9469	GQ01723	TSAO SHENG LI 1::IRGC 1309-1	Indica	66.01	5.405	2.6575	China
11	IRIS_313-8645	GQ01724	PAI YI PING::IRGC 1368-1	Indica	35.26	5.83	2.41	China
12	IRIS_313-9017	GQ01726	PING SHAN TA TSWEN KU::IRGC 1563-1	Indica	45.47	6.22	2.4675	China
13	IRIS_313-8380	GQ01727	SAN CHIAO TSWEN::IRGC 1565-1	Indica	85.60	5.45	2.3125	China
14	IRIS_313-8745	GQ01728	SD 120 SELN (CI 12320)::IRGC 1667-1	Indica	3.21	6.2425	2.58	Haiti
15	IRIS_313-8778	GQ01729	SUP BLUE ROSE::IRGC 1739-1	Tropical japonica	11.62	6.35	2.68	United States
16	IRIS_313-8803	GQ01731	TEXAS PATNA::IRGC 1797-1	Tropical japonica	1.33	6.275	1.955	United States
17	IRIS_313-8960	GQ01732	BLUEBONNET::IRGC 1799-1	Tropical japonica	2.07	6.4	2.05	United States
18	IRIS_313-8323	GQ01733	REXARK ROGUE::IRGC 1972-1	Tropical japonica	2.12	6.18	1.925	United States
19	IRIS_313-9767	GQ01734	CI 9445::IRGC 2081-1	Indica	8.66	6.33	2.2075	United States
20	IRIS_313-9081	GQ01738	TAKAO MOCHI::IRGC 2564-1	Tropical japonica	86.66	4.82	2.82	Japan
21	IRIS_313-9267	GQ01742	CHIRGUA 1 I 22::IRGC 3408-1	Tropical japonica	1.35	6.48	2.635	Venezuela
22	IRIS_313-9372	GQ01743	WI BIR SHUN::IRGC 4602-1	Indica	22.24	5.155	2.63	China
23	IRIS_313-9204	GQ01744	CHI SHENG TAO::IRGC 4606-1	Indica	18.27	5.915	2.64	China
24	IRIS_313-8454	GQ01746	LAI YIP ZIM::IRGC 4955-1	Indica	8.67	5.6475	2.455	Taiwan
25	IRIS_313-9366	GQ01747	IMPROVED BLUE ROSE::IRGC 5022-1	Tropical japonica	1.28	6.015	2.91	United States
26	IRIS_313-9505	GQ01748	KORASISI::IRGC 5285-1	Indica	8.68	5.62	2.7125	Philippines
27	IRIS_313-8743	GQ01750	NIAO YAO::IRGC 5496-1	Indica	31.34	5.265	2.62	Taiwan
28	IRIS_313-9789	GQ01751	RIKUTO TAUKI MOCHI 27::IRGC 5726-1	Tropical japonica	89.46	5.475	2.705	Japan
29	IRIS_313-8876	GQ01752	DELITUS::IRGC 5769-1	Tropical japonica	3.17	6.095	2.435	France
30	IRIS_313-9791	GQ01754	LEAD::IRGC 5805-1	Indica	33.24	5.9375	2.6975	Malawi
31	IRIS_313-8450	GQ01756	498-2A BR 8::IRGC 5891-1	Indica	4.12	6.36	2.3	India
32	IRIS_313-9539	GQ01759	TEXAS PATNA 49::IRGC 6077-1	Tropical japonica	1.93	6.12	2.27	United States
33	IRIS_313-8846	GQ01760	BAIANG 6::IRGC 6129-1	Indica	37.66	5.8375	2.5675	Indonesia
34	IRIS_313-9795	GQ01762	HD 10::IRGC 6638-1	Indica	7.74	5.48	2.5825	Australia
35	IRIS_313-8622	GQ01763	XITTO::IRGC 6671-1	Indica	39.19	5.965	2.755	India

Sl no	IRIS ID	GQNC_NO	Designations	GQNC collection	Chalkiness	Grain length	Grain width	Origin country
36	IRIS_313-9452	GQ01764	B 505 A 1-28-7-1-2::IRGC 6741-1	Tropical japonica	1.41	6.265	1.94	United States
37	IRIS_313-9800	GQ01765	KAHEI::IRGC 7691-1	Tropical japonica	16.45	5.63	2.86	Japan
38	IRIS_313-9550	GQ01766	VEGOLD::IRGC 7756-1	Tropical japonica	2.44	6.62	1.98	United States
39	IRIS_313-9429	GQ01768	RTS 16::IRGC 8235-1	Indica	9.11	4.5775	2.495	Vietnam
40	IRIS_313-9156	GQ01769	DHOLA AMAN (LOWLAND AMAN)::IRGC 8341	Indica	6.80	6.0625	2.2475	Bangladesh
41	IRIS_313-8903	GQ01774	BPI 76 NON SENSITIVE (GREEN)::IRGC 9790-1	Indica	7.47	5.69	2.255	Philippines
42	IRIS_313-8530	GQ01775	DHANE BURWA::IRGC 10105-1	Indica	39.03	5.5675	2.8875	India
43	IRIS_313-9551	GQ01778	BENGALY MORIMO::IRGC 10976-1	Indica	7.02	6.0875	2.015	Madagascar
44	IRIS_313-8288	GQ01779	AVO::IRGC 11000-1	Indica	49.56	5.9275	2.54	Madagascar
45	IRIS_313-9825	GQ01780	MOCHICA::IRGC 11070-1	Indica	0.62	6.426666667	2.23	Peru
46	IRIS_313-8638	GQ01781	NA SOUAN::IRGC 11889-1	Indica	92.92	6.95	2.465	Laos
47	IRIS_313-9831	GQ01782	PODIWEE::IRGC 11938-1	Indica	32.01	5.8525	3	Sri Lanka
48	IRIS_313-9424	GQ01784	ARC 5840::IRGC 12144-1	Indica	15.88	5.4125	2.715	India
49	IRIS_313-9609	GQ01786	ARC 10594::IRGC 12524-1	Indica	9.13	5.6	2.43	India
50	IRIS_313-8986	GQ01787	ARC 10754::IRGC 12603-1	Indica	13.59	6.01	2.275	India
51	IRIS_313-9313	GQ01788	ARC 10799::IRGC 12631-1	Indica	17.88	5.595	2.49	India
52	IRIS_313-8595	GQ01789	MAKALIOKA STANDARD::IRGC 12768-1	Indica	75.84	6.37	2.3975	Madagascar
53	IRIS_313-8781	GQ01793	TAPANG::IRGC 13361-1	Indica	6.39	5.63	2.135	Malaysia
54	IRIS_313-9841	GQ01795	SML AWINI::IRGC 13391-1	Indica	3.09	7.425	2.15	Suriname
55	IRIS_313-8999	GQ01803	ARC 11430 B::IRGC 14567-1	Tropical japonica	52.71	5.145	3.175	India
56	IRIS_313-9590	GQ01804	KETAN SERANG::IRGC 14615-1	Indica	59.72	5.555	2.3125	Indonesia
57	IRIS_313-8812	GQ01805	RELLY::IRGC 14623-1	Indica	36.63	5.4725	2.6175	Indonesia
58	IRIS_313-9851	GQ01806	GOGOWIERIE::IRGC 14791-1	Tropical japonica	3.81	5.175	2.56	Suriname
59	IRIS_313-9862	GQ01808	MODDAI KARUPPAN::IRGC 15465-1	Indica	69.00	5.6325	3.1225	Sri Lanka
60	IRIS_313-9867	GQ01809	SIGARDIS::IRGC 15555-1	Indica	62.44	6.2975	2.5	Sri Lanka
61	IRIS_313-9023	GQ01810	CR 60-10::IRGC 15777-1	Indica	7.83	5.925	2.3225	India
62	IRIS_313-9461	GQ01812	KARANTABA 1::IRGC 15924-1	Indica	0.36	6.17	2.265	Senegal
63	IRIS_313-9005	GQ01814	TJERE SUGI::IRGC 16650-1	Indica	38.51	6.4175	2.31	Indonesia
64	IRIS_313-9882	GQ01817	O. SATIVA::IRGC 17083-1	Indica	87.26	5.63	2.4525	Taiwan
65	IRIS_313-9032	GQ01818	KHAO GRADOOK CHAHNG::IRGC 17111-1	Indica	2.38	6.3	2.3475	Thailand
66	IRIS_313-8643	GQ01820	DAJA::IRGC 17440-1	Indica	2.95	5.51	2.4	Indonesia
67	IRIS_313-8368	GQ01821	MENTIK TJERE BELUT::IRGC 18254-1	Indica	25.01	6.0825	2.16	Indonesia
68	IRIS_313-8436	GQ01822	PIKTO MAMAS::IRGC 18487-1	Tropical japonica	11.30	5.73	2.755	Indonesia
69	IRIS_313-8744	GQ01823	REMOL::IRGC 18624-1	Indica	52.80	5.435	2.655	Indonesia
70	IRIS_313-8956	GQ01831	RADEN KARAMUNTING::IRGC 20098-1	Indica	5.63	5.26	2.3625	Indonesia
71	IRIS_313-9320	GQ01832	SIPULUT HITAM PENDEK::IRGC 20154-1	Indica	85.87	6.005	2.6425	Indonesia
72	IRIS_313-8386	GQ01834	ARC 10812::IRGC 21074-1	Indica	7.76	5.99	2.385	India

Sl no	IRIS ID	GQNC_NO	Designations	GQNC collection	Chalkiness	Grain length	Grain width	Origin country
73	IRIS_313-9176	GQ01835	ARC 11359::IRGC 21348-1	Tropical japonica	31.66	5.155	2.845	India
74	IRIS_313-8585	GQ01837	ARC 11901::IRGC 21727-1	Indica	42.92	6.1875	2.485	India
75	IRIS_313-8967	GQ01838	ARC 12576::IRGC 22163-1	Indica	32.46	6.1425	2.245	India
76	IRIS_313-8603	GQ01839	ARC 12884::IRGC 22417-1	Indica	93.57	5.5275	3.0625	India
77	IRIS_313-9286	GQ01841	LEUANG TAWNG SOOK::IRGC 24394-1	Indica	3.91	6.7175	2.24	Thailand
78	IRIS_313-9119	GQ01842	LEUANG YAI 29-12-46::IRGC 24397-1	Indica	2.80	6.77	2.3775	Thailand
79	IRIS_313-8616	GQ01843	IR 5494 (BPH 1)::IRGC 24484-1	Indica	0.96	6.1925	2.1125	Philippines
80	IRIS_313-9129	GQ01845	LAYANDABU::IRGC 25214-1	Tropical japonica	4.26	6.325	2.47	Indonesia
81	IRIS_313-9897	GQ01849	BIKYAT::IRGC 26276-1	Tropical japonica	93.54	6.345	2.66	Philippines
82	IRIS_313-9139	GQ01850	GOJOL GORIA::IRGC 26629-1	Indica	3.06	5.545	2.36	Bangladesh
83	IRIS_313-9072	GQ01851	MUTA GANJE::IRGC 26744-1	Indica	5.57	5.6425	2.31	Bangladesh
84	IRIS_313-9271	GQ01852	PURA BINNI::IRGC 26772-1	Indica	6.15	5.4975	2.465	Bangladesh
85	IRIS_313-9314	GQ01853	KAAKARAN::IRGC 27138-1	Indica	29.31	6.55	2.2725	Indonesia
86	IRIS_313-8725	GQ01854	PULUT BARAYA::IRGC 27393-1	Indica	51.68	5.13	2.0925	Indonesia
87	IRIS_313-8850	GQ01855	ASHMBER::IRGC 27522-1	Indica	28.88	5.36	2.6175	Bangladesh
88	IRIS_313-8717	GQ01856	BAZAIL::IRGC 27526-1	Indica	38.26	5.28	2.945	Bangladesh
89	IRIS_313-9898	GQ01857	QUERO ASSAN::IRGC 28860-1	Indica	11.76	6.68	2.3925	Portugal
90	IRIS_313-9066	GQ01858	AUS 177::IRGC 29009-1	Indica	2.04	6.07	2.255	Bangladesh
91	IRIS_313-9249	GQ01860	KANU DAM::IRGC 29755-1	Indica	3.34	6.6575	2.4075	Cambodia
92	IRIS_313-9198	GQ01861	KHAO PONG AEV::IRGC 29820-1	Indica	83.85	5.975	2.15	Laos
93	IRIS_313-9297	GQ01863	KORR::IRGC 30751-1	Tropical japonica	13.42	5.865	2.7	Liberia
94	IRIS_313-9256	GQ01864	GALWAKA HANDERAN::IRGC 31381-1	Indica	36.52	5.67	2.8675	Sri Lanka
95	IRIS_313-9067	GQ01865	BHOJON KOLPO::IRGC 31727-1	Indica	31.67	5.9275	2.54	Bangladesh
96	IRIS_313-9617	GQ01866	HOLDI GIRA::IRGC 31801-1	Indica	62.88	5.4825	2.87	Bangladesh
97	IRIS_313-9262	GQ01867	JHODI BIRUN::IRGC 31812-1	Indica	6.60	5.8875	2.3375	Bangladesh
98	IRIS_313-8392	GQ01869	IR 2003-P7-7-4-2::IRGC 32671-1	Indica	13.62	6.0475	2.155	Philippines
99	IRIS_313-9148	GQ01870	BADAL 1163::IRGC 32796-1	Indica	20.01	5.4325	2.73	Bangladesh
100	IRIS_313-8751	GQ01871	HNANWA::IRGC 33118-1	Indica	1.95	6.395	2.295	Myanmar
101	IRIS_313-9285	GQ01872	PALEPYU::IRGC 33549-1	Indica	20.71	5.3725	2.62	Myanmar
102	IRIS_313-8697	GQ01873	YEBAWYIN::IRGC 33885-1	Indica	0.71	6.625	2.42	Myanmar
103	IRIS_313-9604	GQ01874	RPA 5929 (K 45)::IRGC 33963-1	Indica	2.78	6.055	2.345	India
104	IRIS_313-8658	GQ01876	CP 231::IRGC 34018-1	Tropical japonica	2.64	5.795	2.095	United States
105	IRIS_313-9065	GQ01877	PAI CHUEH CHIU LIU::IRGC 34259-1	Indica	24.90	5.415	2.6975	China
106	IRIS_313-9174	GQ01880	KUSHIARA::IRGC 34709-1	Indica	38.58	5.5675	2.85	Bangladesh
107	IRIS_313-8968	GQ01883	KALU ILANKALAYAN::IRGC 36270-1	Indica	30.27	5.455	2.825	Sri Lanka
108	IRIS_313-8925	GQ01884	KURULUTUDU::IRGC 36304-1	Indica	33.40	5.7725	2.7175	Sri Lanka
109	IRIS_313-9917	GQ01885	CHANDINA::IRGC 36420-1	Indica	9.11	5.4725	2.405	Sri Lanka

Sl no	IRIS ID	GQNC_NO	Designations	GQNC collection	Chalkiness	Grain length	Grain width	Origin country
110	IRIS_313-9227	GQ01886	WP 65::IRGC 36526-1	Indica	4.07	6.7975	2.235	Thailand
111	IRIS_313-8932	GQ01888	PATISAIL::IRGC 37562-1	Indica	3.27	5.25	2.2175	Bangladesh
112	IRIS_313-8737	GQ01891	SADAJIRA 19-287::IRGC 38320-1	Indica	21.06	5.7725	2.33	Bangladesh
113	IRIS_313-10147	GQ01896	RD 19::IRGC 39174-1	Indica	1.21	6.6225	2.26	Thailand
114	IRIS_313-9044	GQ01897	IR 2071-586-5-6-3::IRGC 39291-1	Indica	1.38	5.8475	2.2675	Philippines
115	IRIS_313-9566	GQ01898	RP 9-4::IRGC 39735-1	Indica	5.06	6.15	2.2925	India
116	IRIS_313-9560	GQ01901	ARC 11857::IRGC 40972-1	Indica	43.71	5.9	2.49	India
117	IRIS_313-8315	GQ01902	ARC 11867::IRGC 40978-1	Indica	42.86	6.775	2.5	India
118	IRIS_313-8844	GQ01906	ARC 14060::IRGC 41374-1	Indica	15.58	5.705	2.2625	India
119	IRIS_313-8994	GQ01907	ARC 14064::IRGC 41377-1	Indica	58.28	6.4775	2.1	India
120	IRIS_313-8900	GQ01908	ARC 14654::IRGC 41663-1	Indica	8.50	5.9925	2.3425	India
121	IRIS_313-9593	GQ01909	ARC 15088::IRGC 41934-1	Indica	29.39	6.035	2.3225	India
122	IRIS_313-9427	GQ01911	ARC 18092::IRGC 42256-1	Indica	37.88	6.04	2.395	India
123	IRIS_313-8982	GQ01912	ARC 18112::IRGC 42274-1	Indica	9.64	6.115	2.395	India
124	IRIS_313-8458	GQ01913	ARC 18175::IRGC 42316-1	Indica	6.37	5.9975	2.4575	India
125	IRIS_313-8414	GQ01914	ARC 18202::IRGC 42328-1	Indica	20.56	5.5175	2.3425	India
126	IRIS_313-8946	GQ01915	ARC 11524::IRGC 42672-1	Indica	76.57	5.785	2.6675	India
127	IRIS_313-8621	GQ01916	ARC 11663::IRGC 42695-1	Indica	18.65	5.81	1.82	India
128	IRIS_313-8332	GQ01917	ARC 15091::IRGC 43048-1	Indica	29.40	6.1975	2.1325	India
129	IRIS_313-9634	GQ01918	ARC 15873::IRGC 43250-1	Indica	74.36	5.9275	2.2375	India
130	IRIS_313-8453	GQ01919	ARC 18597::IRGC 43299-1	Indica	14.81	5.8125	2.425	India
131	IRIS_313-9405	GQ01921	INGSA BELANAK::IRGC 43402-1	Tropical japonica	26.28	5.785	2.65	Indonesia
132	IRIS_313-8536	GQ01922	NG KARAN DE::IRGC 43489-1	Indica	7.85	5.9475	2.2125	Indonesia
133	IRIS_313-8626	GQ01924	BANJANG::IRGC 44069-1	Tropical japonica	26.63	5.515	2.955	Malaysia
134	IRIS_313-8955	GQ01925	LASAT PUTIH::IRGC 44076-1	Tropical japonica	16.52	5.445	2.875	Malaysia
135	IRIS_313-8948	GQ01926	BUCAYAB::IRGC 44357-1	Indica	4.91	7.56	2.13	Philippines
136	IRIS_313-9348	GQ01927	BK 26::IRGC 45197-1	Indica	1.59	5.75	2.045	India
137	IRIS_313-10148	GQ01928	CAUVERY::IRGC 45255-1	Indica	9.66	5.6125	2.5525	India
138	IRIS_313-9433	GQ01929	GOKULGANJA::IRGC 45701-1	Indica	50.82	5.77	2.8225	India
139	IRIS_313-9259	GQ01930	G 25::IRGC 45733-1	Indica	7.25	5.555	2.6375	India
140	IRIS_313-9602	GQ01931	HTA 22::IRGC 45827-1	Indica	2.58	6.6975	2.3475	Thailand
141	IRIS_313-9287	GQ01932	SONAMUKHI::IRGC 46693-1	Indica	37.81	6.04	2.3525	India
142	IRIS_313-9190	GQ01934	CODE NO 31323::IRGC 46907-1	Indica	1.06	5.3875	1.8725	India
143	IRIS_313-9360	GQ01935	BHASAMANIK::IRGC 46954-1	Indica	2.26	6.1875	2.185	India
144	IRIS_313-9922	GQ01936	IRI 339::IRGC 46956-1	Indica	5.03	5.68	2.445	South Korea
145	IRIS_313-9924	GQ01937	KN 1 B 361-1-8-6-9::IRGC 46974-1	Indica	17.84	5.8175	2.63	South Korea
146	IRIS_313-9925	GQ01938	MILYANG 30::IRGC 46977-1	Indica	5.94	5.07	2.445	South Korea

Sl no	IRIS ID	GQNC_NO	Designations	GQNC collection	Chalkiness	Grain length	Grain width	Origin country
147	IRIS_313-9928	GQ01941	DEO KITAN::IRGC 47197-1	Tropical japonica	95.26	5.7	2.77	Philippines
148	IRIS_313-9929	GQ01942	GOBYERNO::IRGC 47216-1	Tropical japonica	8.15	5.455	2.71	Philippines
149	IRIS_313-8660	GQ01943	KOTTEYARAN::IRGC 47383-1	Indica	11.24	5.2975	2.7775	Sri Lanka
150	IRIS_313-8935	GQ01944	ARC 18061::IRGC 47650-1	Indica	2.60	5.4025	1.9	India
151	IRIS_313-10151	GQ01945	RD 15::IRGC 47705-1	Indica	0.99	6.6825	2.11	Thailand
152	IRIS_313-8980	GQ01946	E DAW HAWM::IRGC 47938-1	Indica	89.86	6.5775	2.51	Thailand
153	IRIS_313-8722	GQ01948	HAWM TOONG::IRGC 48007-1	Indica	88.52	6.465	2.5075	Thailand
154	IRIS_313-8785	GQ01950	LEUANG AWN::IRGC 48257-1	Indica	2.93	6.4925	2.295	Thailand
155	IRIS_313-8872	GQ01951	571::IRGC 48493-1	Tropical japonica	1.37	6.695	2.49	Thailand
156	IRIS_313-8831	GQ01953	KETAN KUNANG KUNENG::IRGC 48730-1	Indica	18.13	5.6775	2.4625	Indonesia
157	IRIS_313-8865	GQ01954	PADI SEPAH::IRGC 48803-1	Tropical japonica	3.21	5.47	2.75	Indonesia
158	IRIS_313-8437	GQ01955	IRRIBINI::IRGC 49094-1	Indica	84.96	5.4125	2.73	Bangladesh
159	IRIS_313-8559	GQ01959	KEERIPALA CHILL PADDY::IRGC 49790-1	Indica	10.44	5.8725	2.5825	India
160	IRIS_313-9351	GQ01961	PARA NELLU::IRGC 50009-1	Indica	20.57	5.81	2.715	India
161	IRIS_313-8431	GQ01964	BOCAO::IRGC 50384-1	Tropical japonica	54.90	5.675	2.875	Philippines
162	IRIS_313-9940	GQ01968	TIKAL 3::IRGC 50649-1	Indica	8.76	6.8925	2.2	Guatemala
163	IRIS_313-9522	GQ01970	RPW 9-4 (SS 1)::IRGC 50690-1	Indica	30.21	5.6875	2.5575	India
164	IRIS_313-9944	GQ01975	SOLOMON RED RICE::IRGC 50950-1	Indica	3.55	5.675	2.565	Solomon Islands
165	IRIS_313-9953	GQ01977	BAMOA A 75::IRGC 51101-1	Indica	9.15	6.875	2.2075	Mexico
166	IRIS_313-9723	GQ01983	AI ZI HUNG::IRGC 51255-1	Indica	29.28	5.445	2.66	China
167	IRIS_313-10154	GQ01984	HE GU TSAO::IRGC 51302-1	Indica	25.74	5.9175	2.5875	China
168	IRIS_313-9388	GQ01985	VARY MALADY MENA::IRGC 51555-1	Indica	39.83	5.5675	2.595	Madagascar
169	IRIS_313-9605	GQ01992	NCS 194::IRGC 51932-1	Indica	9.76	5.535	2.465	India
170	IRIS_313-8924	GQ01997	KUTTA::IRGC 52184-1	Indica	9.69	5.3425	2.62	India
171	IRIS_313-9611	GQ01998	WANGA BARUGULU::IRGC 52261-1	Indica	3.99	5.59	2.395	India
172	IRIS_313-8568	GQ02000	LARHA MUGAD::IRGC 52339-1	Indica	19.89	5.805	2.5325	India
173	IRIS_313-8631	GQ02006	DUDRE::IRGC 52523-1	Indica	13.06	5.8125	2.78	India
174	IRIS_313-8305	GQ02010	URAIBOOL::IRGC 52785-1	Indica	17.15	5.925	2.6875	India
175	IRIS_313-9384	GQ02012	BARIK KUDI::IRGC 52807-1	Indica	0.41	5.2775	1.79	India
176	IRIS_313-9966	GQ02018	CICA 9::IRGC 53079-1	Indica	6.65	6.735	2.22	Colombia
177	IRIS_313-9111	GQ02027	XIA ZHI BAI::IRGC 53437-1	Indica	1.24	5.5925	2.13	China
178	IRIS_313-8614	GQ02033	RAJHUSAI (ACR 12)::IRGC 53630-1	Indica	0.61	4.8225	2.3325	India
179	IRIS_313-9403	GQ02035	BADUIE::IRGC 53715-1	Indica	2.19	5.53	2.235	India
180	IRIS_313-9301	GQ02043	KETAN DONGGO::IRGC 54201-1	Tropical japonica	94.20	5.985	2.73	Indonesia
181	IRIS_313-8727	GQ02048	T 315::IRGC 54792-1	Indica	18.44	6.0125	2.7875	India
182	IRIS_313-8732	GQ02055	WP 36::IRGC 55278-1	Indica	4.11	6.9125	2.2325	Thailand
183	IRIS_313-9968	GQ02057	KULA KARUPPAN::IRGC 55328-1	Indica	29.75	5.44	2.9075	Sri Lanka

Sl no	IRIS ID	GQNC_NO	Designations	GQNC collection	Chalkiness	Grain length	Grain width	Origin country
184	IRIS_313-9970	GQ02059	RACE PERUMAL::IRGC 55347-1	Indica	17.82	5.605	2.81	Sri Lanka
185	IRIS_313-8572	GQ02061	ONOY::IRGC 55418-1	Tropical japonica	7.26	6.075	2.645	Philippines
186	IRIS_313-9976	GQ02065	SSANGDUJO::IRGC 55632-1	Indica	30.50	5.2425	2.5825	South Korea
187	IRIS_313-9980	GQ02069	FORTUNA INIA::IRGC 55865-1	Tropical japonica	4.07	6.915	2.525	Argentina
188	IRIS_313-9317	GQ02074	VEN THAP::IRGC 56138-1	Indica	7.56	5.3225	2.5825	Vietnam
189	IRIS_313-8571	GQ02076	ES 21::IRGC 56171-1	Indica	3.89	7.45	2.33	Tanzania
190	IRIS_313-8768	GQ02079	MOROFIN::IRGC 56698-1	Tropical japonica	4.10	6.195	2.47	Ivory Coast
191	IRIS_313-8687	GQ02081	KEREN FENHO::IRGC 56735-1	Tropical japonica	7.49	5.32	2.635	Guinea-Bissau
192	IRIS_313-8769	GQ02097	KOSAGI::IRGC 57692-1	Tropical japonica	16.02	6.12	2.56	Guinea
193	IRIS_313-8659	GQ02108	YA THAY SAN::IRGC 58245-1	Indica	38.68	6.19	2.1675	Myanmar
194	IRIS_313-9574	GQ02111	B 78-S81::IRGC 58415-1	Indica	3.21	6.575	2.33	Brunei Darussalam
195	IRIS_313-8405	GQ02134	JIN JUN DAO::IRGC 59710-1	Indica	28.30	5.3875	2.5425	China
196	IRIS_313-9727	GQ02137	NAN TE HAO::IRGC 59797-1	Indica	35.96	5.65	2.755	China
197	IRIS_313-9184	GQ02142	XI GAN JING REN::IRGC 60035-1	Indica	10.87	5.5525	2.655	China
198	IRIS_313-9140	GQ02145	81 A 32::IRGC 60162-1	Tropical japonica	14.59	5.335	3.105	China
199	IRIS_313-9568	GQ02148	PAMPANGON (H)::IRGC 60392-1	Tropical japonica	80.56	6.025	2.995	Malaysia
200	IRIS_313-8409	GQ02151	SOM NGHE AN::IRGC 60751-1	Indica	8.89	5.5575	2.665	Vietnam
201	IRIS_313-9182	GQ02152	KHAO MON::IRGC 60764-1	Indica	52.84	6.4	2.305	Myanmar
202	IRIS_313-8599	GQ02153	BUE GA WA::IRGC 60824-1	Tropical japonica	49.90	5.375	2.76	Thailand
203	IRIS_313-8757	GQ02165	NIRGUNI::IRGC 61127-1	Indica	33.13	5.57	2.83	India
204	IRIS_313-8920	GQ02166	PATALASAFED SUNGHAWADO::IRGC 61133-1	Indica	2.95	5.2825	2.015	India
205	IRIS_313-9451	GQ02169	PAMAH S 18::IRGC 61322-1	Indica	15.57	6.64	2.3575	Thailand
206	IRIS_313-8435	GQ02178	UPRH 233::IRGC 61667-1	Indica	50.09	5.335	2.6275	India
207	IRIS_313-10000	GQ02182	SUWEON 311::IRGC 61890-1	Indica	63.29	5.685	2.52	South Korea
208	IRIS_313-10001	GQ02191	C 662083::IRGC 62101-1	Indica	4.02	6.3775	2.13	Taiwan
209	IRIS_313-9572	GQ02193	ASU::IRGC 62154-1	Indica	14.59	5.8125	2.4	Bhutan
210	IRIS_313-8637	GQ02194	BALINGMI::IRGC 62156-1	Tropical japonica	47.36	5.55	2.82	Bhutan
211	IRIS_313-9492	GQ02197	NCS 237::IRGC 62202-1	Indica	40.14	5.53	2.59	India
212	IRIS_313-9400	GQ02212	NCS 964 C::IRGC 62604-1	Indica	14.09	5.8175	2.33	India
213	IRIS_313-8647	GQ02220	PERUNEL::IRGC 63113-1	Indica	7.94	5.8175	2.705	India
214	IRIS_313-10167	GQ02226	IR 13429-109-2-2-1::IRGC 63491-1	Indica	4.28	6.4075	2.1675	Philippines
215	IRIS_313-8433	GQ02229	CHANG LE SAN SHU ZAO::IRGC 63561-1	Indica	18.50	5.555	2.705	China
216	IRIS_313-8412	GQ02231	FEI GAI 122::IRGC 63599-1	Indica	8.95	6.33	2.3375	China
217	IRIS_313-8940	GQ02232	FU ZAO XIAN::IRGC 63619-1	Indica	26.41	5.4275	2.6	China
218	IRIS_313-10168	GQ02236	MEI FENG 9::IRGC 63735-1	Indica	55.04	5.9525	2.8075	China
219	IRIS_313-10170	GQ02237	MIN ZAO 6::IRGC 63772-1	Indica	35.90	5.6	2.6875	China
220	IRIS_313-9570	GQ02238	NX 3533::IRGC 63796-1	Indica	1.36	6.6575	2.2575	China

Sl no	IRIS ID	GQNC_NO	Designations	GQNC collection	Chalkiness	Grain length	Grain width	Origin country
221	IRIS_313-10171	GQ02241	YA NONG ZAO 4::IRGC 63908-1	Indica	36.09	6.12	2.4475	China
222	IRIS_313-10007	GQ02243	GARURA::IRGC 64111-1	Indica	93.90	5.87	2.64	Nepal
223	IRIS_313-8674	GQ02251	HAWM KRUA::IRGC 64333-1	Indica	0.61	6.2475	2.1675	Thailand
224	IRIS_313-8586	GQ02262	PLI KHAO::IRGC 64596-1	Indica	86.94	6.0875	2.8225	Thailand
225	IRIS_313-9529	GQ02268	DAGPA BARA::IRGC 64887-1	Tropical japonica	34.52	5.875	2.89	Bhutan
226	IRIS_313-8815	GQ02269	JANA (NAM)::IRGC 64897-1	Tropical japonica	16.95	5.65	2.685	Bhutan
227	IRIS_313-8857	GQ02270	KAMNAM::IRGC 64902-1	Tropical japonica	28.71	5.705	2.69	Bhutan
228	IRIS_313-8519	GQ02272	MANDASHERPO::IRGC 64913-1	Tropical japonica	27.56	6.13	2.56	Bhutan
229	IRIS_313-9288	GQ02282	TONG MAH::IRGC 65577-1	Indica	9.04	6.12	2.3975	Thailand
230	IRIS_313-10016	GQ02297	BOHOTO BALOOCHESTAN::IRGC 66237-1	Indica	7.78	6.39	2.105	Iran
231	IRIS_313-8699	GQ02303	BALASURIYA A::IRGC 66509-1	Indica	11.85	5.58	2.81	Sri Lanka
232	IRIS_313-9470	GQ02311	SAHULO FACHE SOYO::IRGC 66630-1	Tropical japonica	4.39	5.39	2.47	Indonesia
233	IRIS_313-9691	GQ02314	SIFARASI::IRGC 66644-1	Tropical japonica	0.95	6.35	2.165	Indonesia
234	IRIS_313-8339	GQ02323	BOND::IRGC 66755-1	Tropical japonica	0.46	6.655	2.08	United States
235	IRIS_313-8976	GQ02324	SKY BONNET::IRGC 66759-1	Tropical japonica	0.21	6.44	2.06	United States
236	IRIS_313-8434	GQ02325	TEBONNET::IRGC 66760-1	Tropical japonica	0.37	6.375	1.965	United States
237	IRIS_313-10177	GQ02331	DA GANG ZHAN::IRGC 67103-1	Indica	11.79	5.44	2.5975	China
238	IRIS_313-9503	GQ02343	BINUHANGIN::IRGC 67429-1	Indica	8.54	6.25	2.44	Philippines
239	IRIS_313-8383	GQ02344	GENE::IRGC 67434-1	Indica	37.29	5.9975	2.5925	Philippines
240	IRIS_313-9484	GQ02347	CHNNOR::IRGC 67485-1	Indica	5.83	6.31	2.42	India
241	IRIS_313-8514	GQ02350	DAA MANSA::IRGC 67559-1	Indica	20.65	6.07	2.4625	Ghana
242	IRIS_313-8796	GQ02354	DUDH KADAR::IRGC 67707-1	Indica	4.49	5.9425	2.45	India
243	IRIS_313-8988	GQ02356	BANIKAT::IRGC 67720-1	Indica	1.49	6.7825	2.3225	India
244	IRIS_313-8731	GQ02357	NIBARI::IRGC 67742-1	Indica	23.95	5.485	2.6775	India
245	IRIS_313-9730	GQ02363	BA SHI ZAO::IRGC 67903-1	Indica	28.02	5.835	2.6175	China
246	IRIS_313-9482	GQ02364	E 2024::IRGC 67958-1	Indica	2.52	5.9975	2.0775	China
247	IRIS_313-8914	GQ02365	E 2040::IRGC 67968-1	Indica	8.26	5.7775	2.4925	China
248	IRIS_313-10178	GQ02368	GAO JIAO BAI::IRGC 68047-1	Indica	8.02	5.7175	2.5925	China
249	IRIS_313-10179	GQ02369	GUI HUA ZAO::IRGC 68060-1	Indica	10.94	5.7525	2.535	China
250	IRIS_313-8889	GQ02370	LU MAO ZHAN::IRGC 68159-1	Indica	9.95	5.8075	2.3625	China
251	IRIS_313-10026	GQ02372	KITRANA 1007::IRGC 68517-1	Indica	25.92	5.475	2.2975	Madagascar
252	IRIS_313-8277	GQ02373	K 2 C 14::IRGC 68549-1	Tropical japonica	38.44	5.77	2.85	Ivory Coast
253	IRIS_313-9732	GQ02374	MAMORIAKA::IRGC 68672-1	Indica	7.54	5.895	2.3275	Madagascar
254	IRIS_313-10030	GQ02375	NS 252::IRGC 68878-1	Indica	84.26	5.84	2.63	Madagascar
255	IRIS_313-10034	GQ02377	RIZ INDETERMINE::IRGC 69014-1	Indica	3.33	6.65	2.275	Niger
256	IRIS_313-10040	GQ02381	MILYANG 77::IRGC 69340-1	Indica	3.19	4.865	2.5025	South Korea
257	IRIS_313-10041	GQ02382	BOTOHAVANA MENA::IRGC 69349-1	Indica	34.03	5.69	2.8	Madagascar

Sl no	IRIS ID	GQNC_NO	Designations	GQNC collection	Chalkiness	Grain length	Grain width	Origin country
258	IRIS_313-9740	GQ02383	ROJOFOTSY::IRGC 69402-1	Indica	44.43	5.725	2.7425	Madagascar
259	IRIS_313-10045	GQ02385	BANTA TIMA::IRGC 69474-1	Indica	5.89	6.2375	2.445	Gambia
260	IRIS_313-10046	GQ02386	CHAMA (DWARF)::IRGC 69487-1	Indica	4.01	5.595	2.21	Zambia
261	IRIS_313-10047	GQ02387	EX MARABA GURUKU::IRGC 69582-1	Indica	10.25	5.2475	2.6125	Nigeria
262	IRIS_313-10048	GQ02389	MOVE (MOKOLE)::IRGC 69739-1	Indica	10.18	6.56	2.5325	Benin
263	IRIS_313-9742	GQ02390	LOHAMBITRO::IRGC 69857-1	Tropical japonica	5.34	6.17	2.305	Madagascar
264	IRIS_313-10050	GQ02391	TSIMATAHOPAOSA::IRGC 69884-1	Indica	15.12	6.1875	2.225	Madagascar
265	IRIS_313-8387	GQ02394	FU LI HONG::IRGC 70250-1	Tropical japonica	87.74	4.83	2.68	China
266	IRIS_313-9253	GQ02395	YONG JIN ZAO 3::IRGC 70441-1	Indica	38.45	5.4025	2.7325	China
267	IRIS_313-10054	GQ02396	TOC 5430::IRGC 70487-1	Indica	3.93	6.3975	2.1675	Panama
268	IRIS_313-8884	GQ02398	E WAWNG::IRGC 71140-1	Tropical japonica	4.50	5.81	2.305	Thailand
269	IRIS_313-9123	GQ02399	LEUANG GLIANG::IRGC 71271-1	Indica	2.14	6.205	2.58	Thailand
270	IRIS_313-9050	GQ02401	ANGKARONG::IRGC 71493-1	Tropical japonica	10.05	5.51	2.78	Malaysia
271	IRIS_313-8894	GQ02407	LEKATAN::IRGC 71554-1	Tropical japonica	81.17	5.67	1.9	Malaysia
272	IRIS_313-9558	GQ02408	LIMBAYAN::IRGC 71556-1	Tropical japonica	19.61	5.85	2.675	Malaysia
273	IRIS_313-8883	GQ02409	LOMPUG::IRGC 71559-1	Tropical japonica	62.61	5.8	2.93	Malaysia
274	IRIS_313-9375	GQ02411	PULUTAN::IRGC 71596-1	Tropical japonica	86.98	6.56	2.345	Malaysia
275	IRIS_313-8381	GQ02412	PURAK SIRIBA::IRGC 71598-1	Tropical japonica	59.60	5.75	3.06	Malaysia
276	IRIS_313-8279	GQ02413	SAWAH::IRGC 71612-1	Tropical japonica	58.15	5.895	2.835	Malaysia
277	IRIS_313-8987	GQ02414	SERIBU::IRGC 71615-1	Tropical japonica	2.58	6.335	2.14	Malaysia
278	IRIS_313-9187	GQ02416	WANGKOD::IRGC 71646-1	Tropical japonica	22.56	6.21	2.585	Malaysia
279	IRIS_313-10191	GQ02417	MIN KE ZHAN::IRGC 72230-1	Indica	0.13	5.665	2.165	China
280	IRIS_313-10196	GQ02421	GUO DI HEI::IRGC 72697-1	Indica	36.20	5.3875	2.715	China
281	IRIS_313-10061	GQ02422	ESTRELA A::IRGC 72918-1	Tropical japonica	0.52	7.18	2.12	Portugal
282	IRIS_313-10062	GQ02423	REA::IRGC 73031-1	Tropical japonica	4.17	6.635	2.36	Greece
283	IRIS_313-10065	GQ02424	JAERAERYUKDO::IRGC 73053-1	Tropical japonica	25.93	5.705	2.885	South Korea
284	IRIS_313-10067	GQ02425	JEONBUKGUNGWEONNA::IRGC 73055-1	Tropical japonica	88.63	5.73	2.865	South Korea
285	IRIS_313-10071	GQ02428	CHIYODA WASE::IRGC 74467-1	Tropical japonica	1.14	5.605	2.795	Japan
286	IRIS_313-10073	GQ02429	OWARI MOCHI::IRGC 74513-1	Tropical japonica	94.84	5.49	2.62	Japan
287	IRIS_313-10074	GQ02430	RIKU AIKOKU::IRGC 74514-1	Tropical japonica	2.56	5.265	2.69	Japan
288	IRIS_313-10075	GQ02431	SANGOKU::IRGC 74518-1	Tropical japonica	13.65	5.445	2.78	Japan
289	IRIS_313-10076	GQ02432	SHINA MOCHI::IRGC 74523-1	Tropical japonica	93.98	5.35	2.905	Japan
290	IRIS_313-10077	GQ02433	TAMASHIRO HIKARI::IRGC 74534-1	Tropical japonica	7.99	5.32	2.88	Japan
291	IRIS_313-10078	GQ02434	YOSHINO MOCHI::IRGC 74550-1	Tropical japonica	93.72	5.72	2.86	Japan
292	IRIS_313-10079	GQ02435	DANGO MOCHI::IRGC 74551-1	Tropical japonica	88.99	5.83	2.835	Japan
293	IRIS_313-10080	GQ02436	HIRAKAWA OKUTE::IRGC 74554-1	Tropical japonica	92.84	5.41	2.71	Japan
294	IRIS_313-10082	GQ02437	KYUUSHUU::IRGC 74558-1	Tropical japonica	12.26	5.8	2.835	Japan

Sl no	IRIS ID	GQNC_NO	Designations	GQNC collection	Chalkiness	Grain length	Grain width	Origin country
295	IRIS_313-9389	GQ02440	KINANDANG BUSIKSIK::IRGC 74607-1	Tropical japonica	15.65	5.655	2.92	Philippines
296	IRIS_313-8594	GQ02441	MINATANDA::IRGC 74613-1	Tropical japonica	9.82	5.8	2.96	Philippines
297	IRIS_313-9547	GQ02442	LABRA::IRGC 74757-1	Indica	23.66	5.7925	2.51	India
298	IRIS_313-8704	GQ02444	ROPA::IRGC 74774-1	Indica	5.07	6.3825	2.3625	India
299	IRIS_313-8303	GQ02445	SURMATIYA::IRGC 74779-1	Indica	2.88	5.8925	2.5275	India
300	IRIS_313-8754	GQ02446	TYPE 50::IRGC 74782-1	Indica	35.33	5.565	2.7975	India
301	IRIS_313-9594	GQ02448	ASHI BINNI::IRGC 77216-1	Indica	90.62	5.36	2.7	Bangladesh
302	IRIS_313-8854	GQ02449	CHAKOL::IRGC 77226-1	Indica	21.15	5.8125	2.81	Bangladesh
303	IRIS_313-9218	GQ02451	KALO CHAKOL::IRGC 77258-1	Indica	20.54	5.6075	2.7875	Bangladesh
304	IRIS_313-8930	GQ02452	MUKKALA BAZAL::IRGC 77279-1	Indica	48.85	5.905	2.5625	Bangladesh
305	IRIS_313-9696	GQ02453	NAZIRA SAIL::IRGC 77284-1	Indica	1.20	5.6075	2.385	Bangladesh
306	IRIS_313-8733	GQ02454	NOLOS::IRGC 77285-1	Indica	19.63	5.235	2.87	Bangladesh
307	IRIS_313-8632	GQ02455	PATMADHAI 1::IRGC 77292-1	Indica	27.96	5.7	2.4975	Bangladesh
308	IRIS_313-9325	GQ02457	SADA RUPA::IRGC 77299-1	Indica	12.62	6.2275	2.475	Bangladesh
309	IRIS_313-9557	GQ02459	MULLIKURUVA::IRGC 77529-1	Indica	24.70	4.3425	2.515	India
310	IRIS_313-10096	GQ02464	JOKJEBICHAL::IRGC 77663-1	Tropical japonica	65.97	5.36	2.775	South Korea
311	IRIS_313-10099	GQ02466	MANONGAZATO::IRGC 77876-1	Tropical japonica	10.90	5.825	2.46	Madagascar
312	IRIS_313-9281	GQ02470	KHAO KAI::IRGC 78251-1	Indica	19.54	6.915	2.4525	Thailand
313	IRIS_313-8565	GQ02476	YAHNG LEU::IRGC 78279-1	Tropical japonica	3.71	6	2.7	Thailand
314	IRIS_313-8578	GQ02477	KHAU TRA BONG::IRGC 78342-1	Tropical japonica	97.53	5.955	2.96	Vietnam
315	IRIS_313-8735	GQ02478	NEP NGAU::IRGC 78369-1	Tropical japonica	95.01	5.59	3.1	Vietnam
316	IRIS_313-10102	GQ02479	KELIRENY::IRGC 78426-1	Indica	7.49	5.6225	2.205	Madagascar
317	IRIS_313-9363	GQ02480	MAYBELLE::IRGC 78629-1	Tropical japonica	3.12	6.355	2.055	United States
318	IRIS_313-10220	GQ02481	EPEAL 102::IRGC 78698-1	Indica	5.03	6.41	2.1775	Brazil
319	IRIS_313-9623	GQ02482	PESAGRO 102::IRGC 78703-1	Indica	3.35	6.2	2.1375	Brazil
320	IRIS_313-10103	GQ02483	DALSUNG 41::IRGC 79385-1	Indica	56.19	5.28	2.4125	South Korea
321	IRIS_313-10221	GQ02484	BA BAI GU::IRGC 79580-1	Indica	9.41	5.315	2.3825	China
322	IRIS_313-10109	GQ02485	EX EBOKOZURU::IRGC 79912-1	Indica	8.05	6.7475	2.4925	Nigeria
323	IRIS_313-10224	GQ02486	HUA LI ZAO::IRGC 80950-1	Indica	22.10	5.645	2.505	China
324	IRIS_313-10226	GQ02487	TONG GU HONG::IRGC 81026-1	Indica	32.92	5.9775	2.6775	China
325	IRIS_313-10113	GQ02489	EX FOILAEIN (NAPUTO)::IRGC 81675-1	Indica	2.48	6.4775	2.245	Mozambique
326	IRIS_313-10114	GQ02490	FACAGRO 64::IRGC 82059-1	Indica	39.29	6.08	2.455	Burundi
327	IRIS_313-10228	GQ02491	HAN NUO::IRGC 82350-1	Tropical japonica	87.72	4.94	2.665	China
328	IRIS_313-10124	GQ02493	GALSAEGSSAL SHAREI::IRGC 82881-1	Tropical japonica	51.91	4.85	2.37	South Korea
329	IRIS_313-10234	GQ02495	PSBRC 50::IRGC 99706-1	Indica	22.57	5.9325	2.5525	Philippines
330	IRIS_313-10235	GQ02496	PSBRC 68::IRGC 99711-1	Indica	3.98	6.9225	2.375	Philippines
331	IRIS_313-10237	GQ02497	PSBRC 86::IRGC 99716-1	Indica	2.17	5.705	2.31	Philippines

Sl no	IRIS ID	GQNC_NO	Designations	GQNC collection	Chalkiness	Grain length	Grain width	Origin country
332	IRIS_313-10238	GQ02498	PSBRC 88::IRGC 99717-1	Indica	1.10	6.0975	2.2	Philippines
333	IRIS_313-10298	GQ02499	3210::IRGC 116950-1	Indica	13.62	6.595	2.16	Sri Lanka
334	IRIS_313-10257	GQ02500	ALTAMIRA 9::IRGC 116953-1	Indica	6.23	6.665	2.2425	Nicaragua
335	IRIS_313-10247	GQ02501	AMISTAD 82::IRGC 116954-1	Indica	1.14	6.53	2.165	Cuba
336	IRIS_313-10289	GQ02502	ARAURE 1::IRGC 116956-1	Indica	3.39	6.39	2.2375	Venezuela
337	IRIS_313-10258	GQ02503	BULI INIA::IRGC 116961-1	Tropical japonica	0.09	6.85	2.19	Chile
338	IRIS_313-10325	GQ02504	CAMPONI::IRGC 116963-1	Indica	3.89	6.7125	2.13	Suriname
339	IRIS_313-10260	GQ02505	CEA 3::IRGC 116965-1	Indica	5.80	6.34	2.1875	Paraguay
340	IRIS_313-10300	GQ02506	CT 9506-18-7-1T-2::IRGC 116973-1	Indica	2.65	6.23	2.2775	Colombia
341	IRIS_313-10263	GQ02507	CUYAMEL 3820::IRGC 116975-1	Indica	3.22	6.565	2.1275	Mexico
342	IRIS_313-10293	GQ02508	ECIA 24-107-1::IRGC 116978-1	Indica	0.85	6.6625	2.1525	Cuba
343	IRIS_313-10268	GQ02509	FONAIAP 2::IRGC 116985-1	Indica	5.07	7.0375	2.21	Venezuela
344	IRIS_313-10271	GQ02511	IA CUBA 17::IRGC 116990-1	Indica	7.14	6.43	2.3075	Cuba
345	IRIS_313-10275	GQ02513	ICTA MOTAGUA::IRGC 116995-1	Indica	8.37	6.4225	2.2325	Guatemala
346	IRIS_313-10314	GQ02514	INIAP 10::IRGC 117000-1	Indica	1.99	6.4175	2.105	Ecuador
347	IRIS_313-10307	GQ02515	INIAP 6::IRGC 117002-1	Indica	5.97	5.8475	2.12	Ecuador
348	IRIS_313-10294	GQ02516	IR 21015-72-3-3-3-1::IRGC 117004-1	Indica	1.11	6.3325	2.1275	Philippines
349	IRIS_313-10301	GQ02517	IRGA 959-1-2-2F-4-1-4A-6-CA-6X::IRGC 11700	Indica	9.54	6.065	2.2	Brazil
350	IRIS_313-10279	GQ02518	JUMA 62::IRGC 117011-1	Indica	6.20	6.395	2.1525	ominican Republic
351	IRIS_313-10318	GQ02520	RUSTIC::IRGC 117026-1	Indica	3.92	6.82	2.05	Guyana
352	IRIS_313-10285	GQ02521	SICAN::IRGC 117029-1	Indica	4.97	6.5175	2.2925	Peru
353	IRIS_313-10287	GQ02522	UQUIHUA::IRGC 117037-1	Indica	4.82	6.7575	2.25	Peru
354	IRIS_313-10366	GQ02524	IR 65483-111-5-9-2-11::IRGC 117286-1	Indica	1.26	6.28	2.1775	Philippines
355	IRIS_313-10374	GQ02525	IR 69502-6-SRN-3-UBN-1-B::IRGC 117290-1	Indica	4.34	6.6075	2.2475	Philippines
356	IRIS_313-10385	GQ02526	IR 75870-5-8-5-B-1::IRGC 117297-1	Indica	1.09	6.45	1.9975	Philippines
357	IRIS_313-10392	GQ02527	IR 77390-1-6-4-19-1-B::IRGC 117303-1	Indica	2.95	6.46	2.2375	Philippines
358	IRIS_313-10394	GQ02528	IR 80310-12-B-1-3-B::IRGC 117307-1	Indica	10.56	5.93	2.5425	Philippines
359	IRIS_313-10396	GQ02529	IR 80340-23-B-12-6-B::IRGC 117309-1	Indica	2.22	6.6125	2.28	Philippines
360	IRIS_313-10332	GQ02530	B 4414 F-MR-6-3::IRGC 117310-1	Indica	1.70	5.8475	2.3425	Indonesia
361	IRIS_313-10334	GQ02531	B 6136 E-3-TB-0-1-5::IRGC 117311-1	Indica	12.13	5.6225	2.555	Indonesia
362	IRIS_313-10333	GQ02532	B 6136-3-TB-0-1-5::IRGC 117312-1	Indica	23.27	5.5775	2.6075	Indonesia
363	IRIS_313-10337	GQ02533	B 6149 F-MR-7::IRGC 117314-1	Indica	37.78	6.1025	2.725	Indonesia
364	IRIS_313-10340	GQ02534	BR 4973-34-6-6::IRGC 117317-1	Indica	9.99	6.165	2.3275	Bangladesh
365	IRIS_313-10349	GQ02537	CSR-90 IR-2::IRGC 117327-1	Indica	7.03	6.7525	2.375	India
366	IRIS_313-10352	GQ02538	CT 9737-6-1-1-2-2P-M::IRGC 117330-1	Indica	4.46	6.51	2.2	Colombia
367	IRIS_313-10353	GQ02539	HP 3319-2WX-6-4-1-B::IRGC 117331-1	Indica	92.20	5.75	2.6625	South Korea
368	IRIS_313-10357	GQ02540	IR 52718-B-B-6-B-B-1-1::IRGC 117334-1	Indica	2.94	6.8125	2.345	Philippines

Sl no	IRIS ID	GQNC_NO	Designations	GQNC collection	Chalkiness	Grain length	Grain width	Origin country
369	IRIS_313-10397	GQ02541	IRGA 318-11-6-9-2B::IRGC 117339-1	Indica	0.32	6.84	2.005	Colombia
370	IRIS_313-10398	GQ02542	IRGA 318-11-9-2A::IRGC 117340-1	Indica	0.64	6.8575	2.0375	Colombia
371	IRIS_313-10399	GQ02543	IRGA 346-111-2-1F::IRGC 117341-1	Indica	4.08	5.685	2.4425	Colombia
372	IRIS_313-10400	GQ02544	IRGA 370-38-1-1F-C4-2::IRGC 117342-1	Indica	0.58	6.5875	2.07	Colombia
373	IRIS_313-10401	GQ02545	IRGA 370-42-1-1F-C-1::IRGC 117343-1	Indica	2.89	6.5975	2.1275	Colombia
374	IRIS_313-10402	GQ02546	IRGA 411-1-6-1F-A::IRGC 117344-1	Indica	2.78	6.335	2.0225	Colombia
375	IRIS_313-10403	GQ02547	IRGA 659-1-2-2-2::IRGC 117345-1	Indica	0.12	6.4225	2.26	Colombia
376	IRIS_313-10404	GQ02548	K 479-2-3::IRGC 117346-1	Indica	11.42	5.755	2.5425	India
377	IRIS_313-10412	GQ02549	RR 272-17-829::IRGC 117354-1	Indica	13.39	5.45	2.4525	Indonesia
378	IRIS_313-10417	GQ02550	UPR 1201-1-20-1::IRGC 117357-1	Indica	23.31	6.17	2.47	India
379	IRIS_313-10421	GQ02551	WAR 72-2-1-1::IRGC 117361-1	Indica	10.96	6.0575	2.4825	Sierra Leone
380	IRIS_313-10423	GQ02553	YN 1353-3::IRGC 117363-1	Indica	24.79	5.965	2.5925	Myanmar
381	IRIS_313-10361	GQ02554	IR 63295-AC 209-7::IRGC 117365-1	Indica	33.79	5.775	2.8075	Philippines
382	IRIS_313-10336	GQ02555	B 6144-MR-6-0-0::IRGC 117371-1	Indica	29.52	5.9025	2.5825	Laos
383	IRIS_313-10360	GQ02556	IR 57514-PMI 5-B-1-2::IRGC 117372-1	Indica	1.55	6.7975	2.245	Philippines
384	IRIS_313-10355	GQ02557	IR 31142-14-1-1-3-1-1-2::IRGC 117378-1	Indica	9.39	5.685	2.01	Philippines
385	IRIS_313-10371	GQ02559	IR 67518-B-11-1-B::IRGC 117380-1	Indica	4.58	6.31	2.155	Philippines
386	IRIS_313-10375	GQ02561	IR 70027-8-2-2-3-2::IRGC 117382-1	Indica	29.79	6.33	2.45	Philippines
387	IRIS_313-9002	GQ03533	KOTOBUKI MOCHI::IRGC 2545-1	Temperate japonica	92.11	5.195	2.87	Japan
388	IRIS_313-9438	GQ03534	7507-137::IRGC 40081-1	Temperate japonica	2.91	5.375	2.805	Japan
389	IRIS_313-9346	GQ03535	SHINCHIKU IKU 103::IRGC 10430-1	Temperate japonica	1.47	5.32	2.985	Taiwan
390	IRIS_313-8890	GQ03536	WIR 1072::IRGC 57496-1	Temperate japonica	10.22	5.415	2.93	Belgium
391	IRIS_313-8204	GQ03537	S 102::GERVEX 1671-C1	Temperate japonica	3.60	5.46	3.145	United States
392	IRIS_313-8136	GQ03538	SAFARI::GERVEX 1256-C1	Temperate japonica	15.71	5.995	2.655	Portugal
393	IRIS_313-8062	GQ03539	80 UPLA::GERVEX 164-C1	Temperate japonica	2.65	6.62	1.93	Argentina
394	IRIS_313-8058	GQ03540	91 UPLA::GERVEX 155-C1	Temperate japonica	1.39	6.205	2.285	Argentina
395	IRIS_313-8173	GQ03541	A 201::GERVEX 1638-C1	Temperate japonica	2.23	6.905	2.025	United States
396	IRIS_313-8209	GQ03542	ALEXANDROS::GERVEX 1678-C1	Temperate japonica	4.06	6.54	2.28	Greece
397	IRIS_313-8069	GQ03543	ARIANA::GERVEX 396-C1	Temperate japonica	2.25	6.41	2.2	Romania
398	IRIS_313-8177	GQ03544	AUGUSTO::GERVEX 1643-C1	Temperate japonica	2.13	6.365	2.515	Italy
399	IRIS_313-8171	GQ03545	AUZGUSTA::GERVEX 1634-C1	Temperate japonica	2.86	7.33	2.405	Hungary
400	IRIS_313-8102	GQ03546	BLUE BONNET::GERVEX 669-C1	Temperate japonica	4.26	6.375	2.105	United States
401	IRIS_313-8166	GQ03547	CALENDAL::GERVEX 1516-C1	Temperate japonica	3.69	6.31	3.05	France
402	IRIS_313-8111	GQ03548	CARRICO::GERVEX 828-C1	Temperate japonica	29.23	5.6	3.085	Portugal
403	IRIS_313-8112	GQ03549	CHIPKA::GERVEX 837-C1	Temperate japonica	32.39	5.72	3.18	Bulgaria
404	IRIS_313-8165	GQ03550	CIGALON::GERVEX 1514-C1	Temperate japonica	24.62	5.22	3.025	France
405	IRIS_313-8085	GQ03551	CLOT::GERVEX 523-C1	Temperate japonica	18.63	5.51	2.9	Spain

Sl no	IRIS ID	GQNC_NO	Designations	GQNC collection	Chalkiness	Grain length	Grain width	Origin country
406	IRIS_313-8161	GQ03552	CNA 4081::GERVEX 1494-C1	Temperate japonica	12.78	6.445	2.11	Brazil
407	IRIS_313-8158	GQ03553	CT 23::GERVEX 1488-C1	Temperate japonica	0.05	6.76	2.2	Colombia
408	IRIS_313-8159	GQ03554	CT 36::GERVEX 1489-C1	Temperate japonica	0.04	6.805	2.19	Colombia
409	IRIS_313-8180	GQ03555	DELLMONT::GERVEX 1647-C1	Temperate japonica	1.31	6.51	2.17	United States
410	IRIS_313-8113	GQ03556	ESCARLATE::GERVEX 887-C1	Temperate japonica	100.00	5.855	3.14	Portugal
411	IRIS_313-8114	GQ03557	FAISCA::GERVEX 900-C1	Temperate japonica	5.66	6.255	2.475	Portugal
412	IRIS_313-8115	GQ03558	FAMILIA 181::GERVEX 901-C1	Temperate japonica	7.35	6.695	2.505	Portugal
413	IRIS_313-8099	GQ03559	FLIPPER::GERVEX 597-C1	Temperate japonica	9.27	5.79	2.715	Italy
414	IRIS_313-8087	GQ03560	FRANCES::GERVEX 527-C1	Temperate japonica	1.72	5.14	2.87	Spain
415	IRIS_313-8026	GQ03562	GIOVANNI MARCHETTI::GERVEX 62-C1	Temperate japonica	29.54	5.58	3.105	Italy
416	IRIS_313-8044	GQ03563	GOLFO::GERVEX 118-C1	Temperate japonica	1.08	6.435	2.14	Italy
417	IRIS_313-8074	GQ03564	GOOLARAH::GERVEX 500-C1	Temperate japonica	0.04	6.43	1.97	Australia
418	IRIS_313-8041	GQ03565	GRALDO::GERVEX 108-C1	Temperate japonica	1.29	6.315	2.025	Italy
419	IRIS_313-8116	GQ03566	HAREM::GERVEX 929-C1	Temperate japonica	3.08	7.12	2.455	Portugal
420	IRIS_313-8118	GQ03567	IBO 400::GERVEX 943-C1	Temperate japonica	14.35	6.58	2.885	Portugal
421	IRIS_313-7688	GQ03568	IR 22::IRGC 11356-C1	Temperate japonica	0.58	6.255	2.075	Philippines
422	IRIS_313-8025	GQ03569	ITALPATNA 48::GERVEX 60-C1	Temperate japonica	0.73	6.475	2.455	Italy
423	IRIS_313-8186	GQ03570	JACINTO::GERVEX 1653-C1	Temperate japonica	2.36	6.275	2.05	United States
424	IRIS_313-8119	GQ03571	JUBILIENI::GERVEX 992-C1	Temperate japonica	30.25	5.28	2.87	Bulgaria
425	IRIS_313-7719	GQ03572	KOGONI 91-1::C1	Temperate japonica	1.14	6.555	2.035	Mali
426	IRIS_313-8192	GQ03573	L 205::GERVEX 1659-C1	Temperate japonica	2.58	6.6	2.065	United States
427	IRIS_313-8033	GQ03574	LOMELLINO::GERVEX 83-C1	Temperate japonica	22.85	5.56	3.115	Italy
428	IRIS_313-8049	GQ03575	LORD::GERVEX 127-C1	Temperate japonica	4.14	6.39	2.435	Italy
429	IRIS_313-8039	GQ03576	LOTO::GERVEX 104-C1	Temperate japonica	7.44	5.915	2.725	Italy
430	IRIS_313-8208	GQ03577	LUSITO IRRADIADO 859-85-2::GERVEX 1676-C	Temperate japonica	12.13	6.015	2.74	Portugal
431	IRIS_313-8195	GQ03578	LUXOR::GERVEX 1662-C1	Temperate japonica	8.00	5.93	2.785	Italy
432	IRIS_313-8090	GQ03579	MARENY::GERVEX 535-C1	Temperate japonica	13.90	5.595	2.81	Spain
433	IRIS_313-8214	GQ03581	MELAS::GERVEX 1684-C1	Temperate japonica	0.19	6.565	2.2	Greece
434	IRIS_313-8121	GQ03582	MESTRE::GERVEX 1082-C1	Temperate japonica	20.82	6.305	2.82	Italy
435	IRIS_313-8123	GQ03583	MUGA::GERVEX 1099-C1	Temperate japonica	1.88	5.915	3.15	Portugal
436	IRIS_313-8200	GQ03584	OPALE::GERVEX 1667-C1	Temperate japonica	1.13	6.465	2.29	Italy
437	IRIS_313-8068	GQ03585	ORIONE::GERVEX 333-C1	Temperate japonica	6.34	6.255	2.43	Italy
438	IRIS_313-8076	GQ03586	PELDE::GERVEX 502-C1	Temperate japonica	0.46	6.76	1.875	Australia
439	IRIS_313-8032	GQ03587	PIEMONTE::GERVEX 81-C1	Temperate japonica	27.48	5.645	3.01	Italy
440	IRIS_313-8126	GQ03588	PLOVDIV 24::GERVEX 1168-C1	Temperate japonica	16.09	5.42	3.04	Bulgaria
441	IRIS_313-8046	GQ03589	PLUS::GERVEX 122-C1	Temperate japonica	2.71	6.21	2.025	Italy
442	IRIS_313-8127	GQ03590	POLIZESTI 28::GERVEX 1170-C1	Temperate japonica	22.29	5.665	3.05	Bulgaria

Sl no	IRIS ID	GQNC_NO	Designations	GQNC collection	Chalkiness	Grain length	Grain width	Origin country
443	IRIS_313-8202	GQ03591	REXMONT::GERVEX 1669-C1	Temperate japonica	0.42	6.475	2.035	United States
444	IRIS_313-8024	GQ03592	RIBE 253::GERVEX 54-C1	Temperate japonica	4.04	6.435	2.615	Italy
445	IRIS_313-8023	GQ03593	RIZZOTTO 51/1::GERVEX 50-C1	Temperate japonica	9.73	6.12	2.87	Italy
446	IRIS_313-8129	GQ03594	RODINA::GERVEX 1234-C1	Temperate japonica	27.28	5.79	3.165	Bulgaria
447	IRIS_313-8048	GQ03595	RONCOLO::GERVEX 124-C1	Temperate japonica	21.70	5.265	2.955	Italy
448	IRIS_313-8053	GQ03596	RUBIDIO::GERVEX 146-C1	Temperate japonica	19.44	5.955	2.45	Italy
449	IRIS_313-8132	GQ03597	RUBI::GERVEX 1247-C1	Temperate japonica	0.95	6.415	2.575	Portugal
450	IRIS_313-8138	GQ03598	SALOIO::GERVEX 1259-C1	Temperate japonica	28.96	6.02	3.135	Portugal
451	IRIS_313-8205	GQ03599	SALVO::GERVEX 1672-C1	Temperate japonica	0.25	6.615	1.965	Italy
452	IRIS_313-8065	GQ03600	SAMBA::GERVEX 186-C1	Temperate japonica	2.03	6.68	2.085	Italy
453	IRIS_313-8139	GQ03601	SANDOCA::GERVEX 1262-C1	Temperate japonica	3.60	7.325	2.345	Portugal
454	IRIS_313-8141	GQ03602	SELN 244 A 6-20::GERVEX 1273-C1	Temperate japonica	1.08	5.225	3.025	Australia
455	IRIS_313-8142	GQ03603	SETTANTUNO::GERVEX 1279-C1	Temperate japonica	6.62	5.61	3.125	Portugal
456	IRIS_313-8096	GQ03604	SR 113::GERVEX 553-C1	Temperate japonica	5.26	6.185	2.54	Spain
457	IRIS_313-8145	GQ03605	SUPER::GERVEX 1304-C1	Temperate japonica	31.36	7.225	2.49	Portugal
458	IRIS_313-8105	GQ03606	THAIPERLA::GERVEX 696-C1	Temperate japonica	5.40	5.35	3.09	United States
459	IRIS_313-8148	GQ03607	TIMICH 108::GERVEX 1325-C1	Temperate japonica	10.00	4.915	3.035	Romania
460	IRIS_313-8149	GQ03608	TOPAZIO::GERVEX 1332-C1	Temperate japonica	20.94	5.42	3.12	Portugal
461	IRIS_313-8097	GQ03609	ULLAL::GERVEX 556-C1	Temperate japonica	4.13	5.44	2.82	Spain
462	IRIS_313-8151	GQ03610	VALTEJO::GERVEX 1355-C1	Temperate japonica	35.80	5.675	3.18	Portugal
463	IRIS_313-8183	GQ03611	DREW::GERVEX 1650-C1	Temperate japonica	1.12	6.34	1.99	United States
464	IRIS_313-7725	GQ03612	MADINIKA 1329::GERVEX 8366-C1	Temperate japonica	3.55	6.97	2.25	Madagascar
465	IRIS_313-8669	GQ03613	CALROSE 76::IRGC 34275-1	Temperate japonica	0.19	5.515	2.74	United States
466	IRIS_313-9774	GQ03614	EGYPTIAN::IRGC 3217-1	Temperate japonica	6.74	5.45	2.905	Turkey
467	IRIS_313-10083	GQ03615	BETIS::IRGC 74581-1	Temperate japonica	0.48	6.02	2.615	Spain
468	IRIS_313-9491	GQ03616	BLUE BELLE::IRGC 51125-1	Temperate japonica	1.13	6.515	2.085	United States
469	IRIS_313-10092	GQ03617	CHALBYEO::IRGC 77639-1	Temperate japonica	91.74	5.005	2.925	South Korea
470	IRIS_313-10093	GQ03618	CHEONJUDO::IRGC 77644-1	Temperate japonica	0.00	4.93	2.92	South Korea
471	IRIS_313-9703	GQ03619	CHIANAN 8::IRGC 90-1	Temperate japonica	1.44	5.05	2.99	Taiwan
472	IRIS_313-9463	GQ03622	CI 1600::IRGC 16305-1	Temperate japonica	9.66	5.21	2.945	United States
473	IRIS_313-9410	GQ03623	CI 9498::IRGC 2134-1	Temperate japonica	6.68	5.445	3.035	United States
474	IRIS_313-10059	GQ03624	DACHEONGBYEO::IRGC 72533-1	Temperate japonica	0.42	5.085	2.605	South Korea
475	IRIS_313-9725	GQ03625	DA DAO TOU::IRGC 59499-1	Temperate japonica	3.85	5.745	3.04	China
476	IRIS_313-9193	GQ03626	EAN 4 C 1::IRGC 26016-1	Temperate japonica	6.98	6.645	2.825	Brazil
477	IRIS_313-9884	GQ03627	FUKUSHIMA MOCHI (GLUT)::IRGC 19296-1	Temperate japonica	95.00	4.98	3.01	Japan
478	IRIS_313-10094	GQ03628	GAWICHAL::IRGC 77653-1	Temperate japonica	95.81	5.745	2.91	South Korea
479	IRIS_313-10119	GQ03629	GITANO::IRGC 82424-1	Temperate japonica	0.34	6.86	2.48	Italy

Sl no	IRIS ID	GQNC_NO	Designations	GQNC collection	Chalkiness	Grain length	Grain width	Origin country
480	IRIS_313-9974	GQ03630	HEUKSANJO::IRGC 55536-1	Temperate japonica	4.82	4.97	2.845	South Korea
481	IRIS_313-10056	GQ03631	HOKURIKU 52::IRGC 72491-1	Temperate japonica	0.28	4.985	2.755	Japan
482	IRIS_313-9228	GQ03632	HOKUSETSU::IRGC 65705-1	Temperate japonica	6.35	4.67	2.935	Japan
483	IRIS_313-9886	GQ03633	HUK ZO::IRGC 19760-1	Temperate japonica	0.80	5.075	3.005	South Korea
484	IRIS_313-9379	GQ03634	HWANGJO::IRGC 55547-1	Temperate japonica	3.36	4.71	2.91	South Korea
485	IRIS_313-9790	GQ03635	JAPONES BALILLA::IRGC 5785-1	Temperate japonica	14.82	5.36	3.02	Uruguay
486	IRIS_313-9887	GQ03636	JEUK DO::IRGC 19775-1	Temperate japonica	18.25	4.935	2.85	South Korea
487	IRIS_313-9698	GQ03637	JO SANG DAE YA::IRGC 90852-1	Temperate japonica	0.37	4.85	2.97	North Korea
488	IRIS_313-10089	GQ03639	KALIN::IRGC 77312-1	Temperate japonica	30.21	5.315	3.315	Bulgaria
489	IRIS_313-10097	GQ03640	MAEKJO::IRGC 77666-1	Temperate japonica	2.58	4.965	2.98	South Korea
490	IRIS_313-10111	GQ03641	MOLO::IRGC 81603-1	Temperate japonica	0.97	6.07	2.515	Italy
491	IRIS_313-9769	GQ03642	MURASAHITSUTSURI::IRGC 2493-1	Temperate japonica	93.32	5.12	2.97	Japan
492	IRIS_313-9468	GQ03643	NONG KE::IRGC 59807-1	Temperate japonica	2.64	5.12	3.145	China
493	IRIS_313-9995	GQ03644	NONGLIMNA 1::IRGC 58347-1	Temperate japonica	89.92	5.32	2.74	South Korea
494	IRIS_313-9759	GQ03645	OEIRAS::IRGC 286-1	Temperate japonica	13.74	5.59	3.04	Portugal
495	IRIS_313-9839	GQ03646	O. SATIVA::IRGC 12876-1	Temperate japonica	30.49	5.715	3.105	Spain
496	IRIS_313-8856	GQ03648	S 201::IRGC 55230-1	Temperate japonica	10.89	5.38	3.095	United States
497	IRIS_313-10014	GQ03649	SANT ANDREA::IRGC 65732-1	Temperate japonica	8.46	6.25	3.04	Italy
498	IRIS_313-10084	GQ03650	SENIA::IRGC 74582-1	Temperate japonica	12.34	5.66	2.935	Spain
499	IRIS_313-9813	GQ03651	SZANISZLO 2::IRGC 9353-1	Temperate japonica	26.68	6.33	3.06	Hungary
500	IRIS_313-9702	GQ03652	TAICHUNG 179::IRGC 85-1	Temperate japonica	5.54	4.77	2.935	Taiwan
501	IRIS_313-9701	GQ03653	TAICHUNG 65::IRGC 79-1	Temperate japonica	2.05	5.235	3.005	Taiwan
502	IRIS_313-9838	GQ03654	TEPUKE::IRGC 12872-1	Temperate japonica	7.41	5.22	2.955	New Zealand
503	IRIS_313-9048	GQ03656	TSCHINANANGKA::IRGC 64935-1	Temperate japonica	38.24	5.44	2.92	Bhutan
504	IRIS_313-9890	GQ03657	WA BANG::IRGC 19880-1	Temperate japonica	3.24	5.105	3.095	South Korea
505	IRIS_313-9964	GQ03658	WIR 1951::IRGC 51643-1	Temperate japonica	1.78	6.355	2.67	Georgia
506	IRIS_313-9891	GQ03660	YONG AN HUK::IRGC 19891-1	Temperate japonica	2.82	5.155	2.975	South Korea
507	IRIS_313-10359	GQ03661	IR 53650-2B-10-1-2-1::IRGC 117379-1	Temperate japonica	16.02	6.235	2.35	Philippines
508	IRIS_313-10379	GQ03662	IR 73688-57-2::IRGC 117383-1	Temperate japonica	0.34	4.975	2.8	Philippines
509	IRIS_313-8399	GQ03664	68-2::IRGC 14546-1	Temperate japonica	1.48	5.655	2.64	France
510	IRIS_313-8755	GQ03667	NORIN 6::IRGC 2633-1	Temperate japonica	10.64	5.25	3.04	Japan
511	IRIS_313-8356	GQ03668	BLICCA::IRGC 47151-1	Temperate japonica	6.75	5.845	2.795	Philippines
512	IRIS_313-8481	GQ03669	DAN YAN NUO::IRGC 4860-1	Temperate japonica	4.90	4.915	2.935	China
513	IRIS_313-9197	GQ03670	GABAHSI RASAKI C 90::IRGC 49647-1	Temperate japonica	65.23	5.235	2.66	India
514	IRIS_313-8627	GQ03671	PRELUDE::IRGC 1790-1	Temperate japonica	1.91	6.5	2.555	United States
515	IRIS_313-9239	GQ03672	RIMBUN::IRGC 43741-1	Temperate japonica	14.32	6.55	2.39	Indonesia
516	IRIS_313-9969	GQ03673	PUTTU NELLU::IRGC 55346-1	Temperate japonica	90.56	5.725	2.485	Sri Lanka

Sl no	IRIS ID	GQNC_NO	Designations	GQNC collection	Chalkiness	Grain length	Grain width	Origin country
517	IRIS_313-9782	GQ03674	BENLLOK::IRGC 3404-1	Temperate japonica	2.68	5.18	3.035	Peru
518	IRIS_313-10327	GQ03675	CAROLINO BLANCO::IRGC 117249-1	Temperate japonica	14.61	6.125	2.81	Peru
519	IRIS_313-9616	GQ03676	INITLOG DALAG::IRGC 67436-1	Temperate japonica	45.31	4.105	2.515	Philippines
520	IRIS_313-8665	GQ03677	M 7::IRGC 34281-1	Temperate japonica	0.31	5.695	2.69	United States
521	IRIS_313-10242	GQ03678	H 305-84::IRGC 116988-1	Temperate japonica	6.97	6.81	2.33	Hungary
522	IRIS_313-11585	GQ03679	ACC 1286::IRGC 60116-1	Temperate japonica	25.96	5.195	2.85	China
523	IRIS_313-11437	GQ03680	CAMPECHE A 79::IRGC 51102-1	Temperate japonica	5.01	6.645	2.11	Mexico
524	IRIS_313-11671	GQ03681	DERAWA::IRGC 64106-1	Temperate japonica	4.68	5.65	2.36	Nepal
525	IRIS_313-11747	GQ03682	E 4197::IRGC 68004-1	Temperate japonica	1.19	6.195	2.475	China
526	IRIS_313-11573	GQ03683	GAO LIANG ZAO::IRGC 59563-1	Temperate japonica	22.91	5.31	2.545	China
527	IRIS_313-11570	GQ03684	GENG 77-4::IRGC 59321-1	Temperate japonica	50.43	5.71	2.495	China
528	IRIS_313-11802	GQ03685	JIE CAO ZHAN::IRGC 70305-1	Temperate japonica	29.04	5.54	2.63	China
529	IRIS_313-11724	GQ03686	KONYAN::IRGC 66931-1	Temperate japonica	22.66	5.645	2.665	Guinea
530	IRIS_313-11828	GQ03687	LALI GURMATIA::IRGC 70854-1	Temperate japonica	5.26	5.775	2.77	India
531	IRIS_313-11577	GQ03688	LANG QIAN CHE::IRGC 59730-1	Temperate japonica	19.46	5.7	2.545	China
532	IRIS_313-11478	GQ03689	LOCAL::IRGC 53300-1	Temperate japonica	18.58	5.9	2.4	India
533	IRIS_313-11609	GQ03690	MAE HAWM::IRGC 61283-1	Temperate japonica	9.21	5.285	2.455	Thailand
534	IRIS_313-10440	GQ03691	NAGKAYAT::IRGC 584-1	Temperate japonica	95.14	5.105	2.87	Philippines
535	IRIS_313-11650	GQ03692	NCS 901 A::IRGC 62568-1	Temperate japonica	5.33	5.865	2.405	India
536	IRIS_313-11508	GQ03693	PAWNG AEW 1::IRGC 55263-1	Temperate japonica	1.02	6.185	2.245	Thailand
537	IRIS_313-10631	GQ03694	SHINCHIKU IKU 97::IRGC 10429-1	Temperate japonica	1.98	5.215	3.01	Taiwan
538	IRIS_313-10429	GQ03695	TAICHUNG 150::IRGC 80-1	Temperate japonica	1.48	5.345	2.91	Taiwan
539	IRIS_313-11580	GQ03696	TAI ZHOU XIAN::IRGC 59912-1	Temperate japonica	0.08	5.635	2.2	China
540	IRIS_313-11202	GQ03697	4583::IRGC 36894-2	Temperate japonica	0.69	5.195	3	China
541	IRIS_313-10564	GQ03699	250 KUNGANI 1::IRGC 7370-1	Temperate japonica	2.96	5.07	2.945	Japan
542	IRIS_313-10567	GQ03700	AIKAWA 44::IRGC 7676-1	Temperate japonica	0.71	5.13	2.905	Japan
543	IRIS_313-10569	GQ03701	BEN KEI::IRGC 7769-1	Temperate japonica	5.74	5.305	2.985	Japan
544	IRIS_313-11890	GQ03702	C 722323::IRGC 73147-1	Temperate japonica	13.27	4.98	2.865	Taiwan
545	IRIS_313-10618	GQ03703	CHINES::IRGC 9316-1	Temperate japonica	12.59	5.265	3.03	
546	IRIS_313-11875	GQ03704	CHUBU 17::IRGC 72505-1	Temperate japonica	0.95	4.805	2.775	Japan
547	IRIS_313-10570	GQ03705	CHUSEI HONEN::IRGC 7777-1	Temperate japonica	0.10	4.975	2.895	Japan
548	IRIS_313-10677	GQ03706	DEWAMINORI::IRGC 12743-1	Temperate japonica	0.96	5.22	2.69	Japan
549	IRIS_313-11908	GQ03707	DUAN SHEN ZI::IRGC 73962-1	Temperate japonica	86.45	5.425	2.75	China
550	IRIS_313-11651	GQ03708	FEI ZHAO 12::IRGC 62683-1	Temperate japonica	0.78	5.01	2.89	China
551	IRIS_313-10430	GQ03709	GINMASARI::IRGC 242-1	Temperate japonica	0.29	4.965	2.985	Japan
552	IRIS_313-11652	GQ03710	GONG SHE 9::IRGC 62693-1	Temperate japonica	1.97	5.46	3.05	China
553	IRIS_313-12003	GQ03711	GYEONGSAN 1::IRGC 79404-1	Temperate japonica	50.90	5.165	2.695	South Korea

Sl no	IRIS ID	GQNC_NO	Designations	GQNC collection	Chalkiness	Grain length	Grain width	Origin country
554	IRIS_313-11574	GQ03712	HEI TOU HONG::IRGC 59595-1	Temperate japonica	2.83	5.52	3.03	China
555	IRIS_313-12054	GQ03713	HUA 24::IRGC 82127-1	Temperate japonica	0.96	5.26	2.925	China
556	IRIS_313-10967	GQ03714	IAS 22-8 PALMAR::IRGC 26058-1	Temperate japonica	27.24	5.87	2.535	Brazil
557	IRIS_313-11155	GQ03715	K 113::IRGC 34107-1	Temperate japonica	0.32	5.495	2.485	India
558	IRIS_313-10469	GQ03717	KINUGASAWASE::IRGC 2609-1	Temperate japonica	0.94	5.155	2.965	Japan
559	IRIS_313-11487	GQ03718	LALDIGHA::IRGC 53541-1	Temperate japonica	4.40	6.125	2.66	Bangladesh
560	IRIS_313-12059	GQ03719	LIGEN 2::IRGC 82398-1	Temperate japonica	59.21	5.18	2.715	China
561	IRIS_313-12060	GQ03720	LIJIAN 942::IRGC 82399-1	Temperate japonica	14.41	5.495	2.585	China
562	IRIS_313-10558	GQ03721	LITCHIKIANG::IRGC 7287-1	Temperate japonica	96.67	5.015	2.995	China
563	IRIS_313-10453	GQ03722	LU TAO 2::IRGC 1175-1	Temperate japonica	93.82	4.915	2.91	China
564	IRIS_313-11467	GQ03723	MACAPAGAL::IRGC 52927-1	Temperate japonica	1.17	6.535	2.24	Philippines
565	IRIS_313-11428	GQ03724	MARANHAO BRANCO::IRGC 50503-1	Temperate japonica	2.96	6.88	2.495	Brazil
566	IRIS_313-11653	GQ03725	MA SHE 8::IRGC 62750-1	Temperate japonica	7.29	5.065	3.165	China
567	IRIS_313-10437	GQ03726	NORIN 21::IRGC 493-1	Temperate japonica	0.64	5.12	2.84	Japan
568	IRIS_313-10568	GQ03727	OITA MII 120::IRGC 7696-1	Temperate japonica	1.20	5.005	2.86	Japan
569	IRIS_313-10642	GQ03728	SACHIKAZE::IRGC 10891-1	Temperate japonica	0.14	5.255	2.815	Japan
570	IRIS_313-10563	GQ03729	SHA TIAO TSAO::IRGC 7339-1	Temperate japonica	16.12	6.3	2.705	
571	IRIS_313-11582	GQ03730	WAN GENG BAI DAO TOU::IRGC 59948-1	Temperate japonica	10.81	4.985	2.915	China
572	IRIS_313-12217	GQ03731	WEONJU 8::IRGC 90845-1	Temperate japonica	0.93	5.21	2.955	South Korea
573	IRIS_313-11660	GQ03732	WULE DASIMA::IRGC 63450-1	Temperate japonica	29.01	5.965	2.71	Liberia
574	IRIS_313-10840	GQ03733	YE ZO::IRGC 19888-1	Temperate japonica	3.19	4.965	2.98	South Korea
575	IRIS_313-12061	GQ03734	YUNLEN 13::IRGC 82402-1	Temperate japonica	13.01	4.89	2.725	China
576	IRIS_313-11336	GQ03735	IREQUIN::IRGC 44476-2	Temperate japonica	93.51	5.065	2.91	Philippines
577	IRIS_313-10617	GQ03736	KARA SHALI::IRGC 9293-1	Temperate japonica	100.00	5.36	2.955	
578	IRIS_313-11973	GQ03738	NOINJO::IRGC 77669-1	Temperate japonica	5.70	4.73	2.96	South Korea
579	IRIS_313-11498	GQ03739	PADI HEDENG (REKET HEDENG)::IRGC 54266-	Temperate japonica	57.33	5.865	2.545	Indonesia
580	IRIS_313-11981	GQ03740	TAICHUNG 188::IRGC 78209-1	Temperate japonica	3.10	5.23	3.025	Taiwan
581	IRIS_313-10837	GQ03742	BACK KYUNG ZO::IRGC 19698-2	Temperate japonica	10.90	5.16	2.91	South Korea
582	IRIS_313-10838	GQ03743	SSAL BYEO::IRGC 19867-2	Temperate japonica	7.97	4.99	2.83	South Korea
583	IRIS_313-15904	GQ03744	JINBUBYEO::G1	Temperate japonica	0.04	5.23	2.93	South Korea
